# Classifying atopic dermatitis: a systematic review of phenotypes and associated characteristics

**DOI:** 10.1111/jdv.18008

**Published:** 2022-02-25

**Authors:** A.L. Bosma, A. Ascott, R. Iskandar, K. Farquhar, J. Matthewman, M.W. Langendam, A. Mulick, K. Abuabara, H.C. Williams, P.I. Spuls, S.M. Langan, M.A. Middelkamp‐Hup

**Affiliations:** ^1^ Department of Dermatology Amsterdam Public Health, Infection and Immunity Amsterdam UMC, location Academic Medical Center University of Amsterdam Amsterdam The Netherlands; ^2^ 8721 Department of Dermatology Worthing University Hospitals Sussex NHS Foundation Trust Worthing UK; ^3^ 4906 Faculty of Epidemiology and Population Health London School of Hygiene and Tropical Medicine London UK; ^4^ 3529 NHS Greater Glasgow and Clyde Glasgow UK; ^5^ 4906 Department of Non‐communicable Disease Epidemiology London School of Hygiene and Tropical Medicine London UK; ^6^ Department of Epidemiology and Data Science Amsterdam Public Health Research Institute Amsterdam UMC, location Academic Medical Center University of Amsterdam Amsterdam The Netherlands; ^7^ 1438 Department of Dermatology University of California San Francisco San Francisco CA USA; ^8^ Centre of Evidence‐Based Dermatology University of Nottingham Nottingham UK

## Abstract

Atopic dermatitis is a heterogeneous disease, accompanied by a wide variation in disease presentation and the potential to identify many phenotypes that may be relevant for prognosis and treatment. We aimed to systematically review previously reported phenotypes of atopic dermatitis and any characteristics associated with them. Ovid EMBASE, Ovid MEDLINE and Web of Science were searched from inception till 12 February 2021 for studies attempting to classify atopic dermatitis. Primary outcomes are atopic dermatitis phenotypes and characteristics associated with them in subsequent analyses. A secondary outcome is the methodological approach used to derive them. In total, 8511 records were found. By focussing only on certain clinical phenotypes, 186 studies were eligible for inclusion. The majority of studies were hospital‐based (59%, 109/186) and cross‐sectional (76%, 141/186). The number of included patients ranged from seven to 526 808. Data‐driven approaches to identify phenotypes were only used in a minority of studies (7%, 13/186). Ninety‐one studies (49%) investigated a phenotype based on disease severity. A phenotype based on disease trajectory, morphology and eczema herpeticum was investigated in 56 (30%), 22 (12%) and 11 (6%) studies respectively. Thirty‐six studies (19%) investigated morphological characteristics in other phenotypes. Investigated associated characteristics differed between studies. In conclusion, we present an overview of phenotype definitions used in literature for severity, trajectory, morphology and eczema herpeticum, including associated characteristics. There is a lack of uniform and consistent use of atopic dermatitis phenotypes across studies.

## Introduction

Atopic dermatitis (AD), also known as (atopic) eczema, is a common chronic inflammatory skin condition characterized by pruritus. It is a heterogeneous disease with a wide spectrum in clinical presentation, which may change over time. Besides a variety in clinical presentation (e.g. presence of the eczema in the flexures vs. nonflexural eczema), some have described distinct subtypes based on nonclinical features [e.g. presence of filaggrin (FLG) mutations or serum immunoglobulin E (IgE)]. AD is considered both an immunological and skin barrier disorder. The disease is influenced by endogenous factors, i.e. a genetic predisposition, as well as by exposure to environmental factors.^1^

In general, the term phenotype is a comprehensive concept and is used in numerous ways in the literature. There is a need for comparability between studies. A phenotype could be defined as a set of features of an individual resulting from the interplay between genetic and environmental factors. Due to its complexity in presentation and pathogenesis, various attempts have been made to classify AD into phenotypes.^2^ Phenotypes within AD can be distinguished based on various features, which could include any static or dynamic feature such as clinical presentation (i.e. morphology and course of disease), or nonclinical features (e.g. based on genetics or immunology).^3^ The identification of clinically meaningful phenotypes could be a first step to enable stratification of patients in the context of personalised medicine.

The primary objective of this systematic review was to report AD phenotypes, focussing on certain clinical phenotypes, that have been published in the literature and how these were defined, as well as to investigate which patient characteristics were associated with these phenotypes in subsequent analyses. Our secondary objective was to summarize the methodological approaches used to derive the phenotypes. To this point in time, no studies have been undertaken to systematically review the literature and summarize previously defined phenotypes in the field of AD.

## Methods

### Protocol and registration

The protocol for this systematic review has been published prior to the start of this study.^3^ In addition, the protocol was registered in the International Prospective Register of Systematic Reviews (PROSPERO; CRD42018087500).^4^ The changes to the protocol are summarized in Appendix S1 (Supporting Information). The study is reported in accordance with the Preferred Reporting Items for Systematic Reviews and Meta‐Analyses (PRISMA) guidelines.^5^

### Eligibility criteria

In the context of this systematic review, we have defined phenotype as any subtype or subgroup of AD patients in which associated characteristics were investigated.^3^ Subgroups of AD patients could be defined based on any feature, including both clinical and nonclinical features. We have included published studies that have a main aim to describe at least one of the following five phenotypic groupings:

1. The AD phenotype is defined by disease severity (e.g. mild, moderate‐to‐severe, severe).
2. The AD phenotype is defined by disease trajectory (e.g. early‐onset, late‐onset).
3. The AD phenotype is defined by morphological features (i.e. based on findings at physical examination [e.g. flexural eczema]); and
4. The AD phenotype is defined by (history of) eczema herpeticum.
  In these four phenotypic groupings, the associated characteristics (e.g. FLG mutations) are subsequently investigated per phenotype. For papers that did not define the phenotype by morphological features (see under 3), but instead first determined the phenotype (e.g. based on FLG mutations) in order to describe morphological characteristics in these subgroups, we included as a fifth phenotype:
5. The study defines the AD phenotype based on a certain feature (e.g. FLG mutations) in order to investigate morphological characteristics in these phenotypes.

We have excluded studies of localised eczema such as hand eczema, if not mentioned specifically in patients with AD, and other types of eczema such as contact dermatitis and seborrheic dermatitis; literature reviews, case reports and case series; conference abstracts, books and book chapters; and studies on other phenotype categories than defined above (including subgroups based only on age, gender, ethnic populations, presence of triggers, comorbidities, immunology and genetics). Ichthyosis vulgaris, prurigo nodularis and keratosis pilaris in AD patients were considered morphological features.

### Search strategy and information sources

A comprehensive literature search strategy was developed in consultation with a clinical librarian. We have searched Ovid EMBASE, Ovid MEDLINE and Web of Science from inception till 12 February 2021. No language restrictions or filters were applied. The Ovid MEDLINE search strategy can be found in Appendix S2 (Supporting Information). In addition, the reference lists from three major review articles were hand‐searched for relevant studies.^1^, ^2^, ^6^

### Study selection process

The results of the literature search were uploaded into Covidence online software. All titles and abstracts were screened independently by two reviewers, using a screening tool based on our eligibility criteria. Publications that both reviewers recorded as meeting the inclusion criteria were retrieved for full‐text review and excluded when not meeting the criteria. Disagreements were discussed with a second reviewer if necessary. Persistent conflicts were resolved with a senior author. Thereafter, full‐text publications were reviewed in duplicate by two separate reviewers. Disagreements were resolved after discussion between the reviewers and with a senior author if necessary.

### Data extraction process

Data from each full‐text publication were independently extracted by two reviewers (A.B., A.A., R.I., K.F. and J.M.), using a data extraction form designed for this purpose. Discrepancies in data extraction were resolved by discussion if necessary.

### Data items

We extracted the following data domains from the included publications using our predesigned data extraction form: study data, disease data and outcome data. The study data comprised the following items: year(s) conducted, study design, setting conducted in, country/countries conducted in, World Health Organization (WHO) region, and the number, age and gender of the participants with (atopic) eczema. The following disease data items were extracted: disease description, diagnostic criteria/codes and disease severity definition. The following outcome data items were extracted: qualitative description of the phenotype(s), proportion of individuals in each phenotype (if relevant), qualitative description of the characteristic(s) (of a priori interest) potentially associated with the phenotype(s), result of the statistical analyses on the association, methodological approach for deriving phenotype(s) and/or investigating the association (including a data‐driven approach using statistical techniques, rather than the predefinition of phenotypes, if applicable), and whether controls were included (including the number).

### Synthesis of results

The results are reported descriptively. We anticipated that both the phenotype definitions and potentially associated characteristics that are investigated would vary between studies. Therefore, we expected heterogeneity in all outcomes. We have grouped studies into categories where possible and composed evidence tables per phenotype category. If more than one phenotype category was applicable to one study, the publication was grouped into all relevant categories.

### Risk of bias assessment

Risk of bias was assessed per study using the critical appraisal checklists for analytical cross‐sectional studies, cohort studies and case‐control studies from the Joanna Briggs Institute (JBI), as appropriate.^7^ In the forms, we have treated the described phenotype as the outcome and the description of the potentially associated characteristics under investigation as the exposure. Traffic light tables were composed according to study design and phenotype category to visualize the qualitative results descriptively.

### Quality of the evidence

We aimed to use the Grading of Recommendations Assessment, Development and Evaluation (GRADE) approach for assessing the quality of evidence per phenotype category. As we anticipated that the phenotype definitions and potentially associated characteristics would vary between studies, an assessment was made whether the quality of evidence per phenotype category could be investigated.

## Results

### Search results

**Figure 1:**
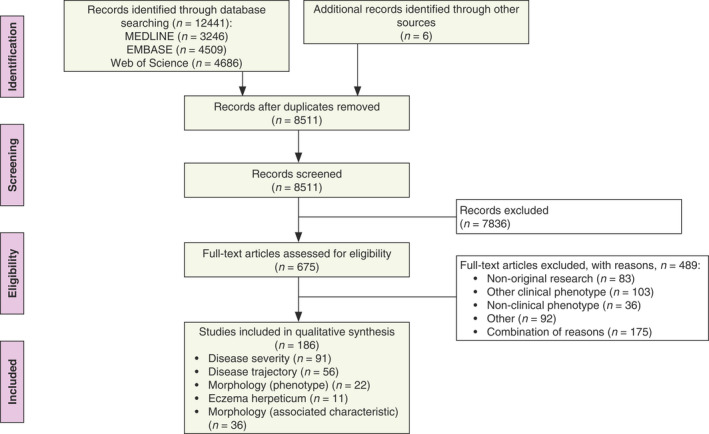
PRISMA flow diagram.

We have screened 8511 records and have assessed 675 full‐text publications. In total, 186 studies, published between 1966 and 2021, fulfilled the inclusion criteria. Reference searching has yielded 6 additional publications. Figure 1 gives an overview of the study selection process, including reasons for exclusion.

### Study overview

Of the included articles, 59% (109/186) was hospital‐based (medical specialist setting). Regarding study design, 76% (141/186) was cross‐sectional studies. In 7% of studies (13/186), a data‐driven approach was used to derive phenotypes, including two studies using existing data‐driven phenotypes. The number of included AD patients ranged from seven to 526 808. Ninety‐one (49%) publications investigated phenotypes based on disease severity (phenotype group 1). Phenotypes based on disease trajectory (phenotype group 2) were investigated in 56 (30%) studies. Thirty‐six (19%) studies investigated morphological characteristics in other phenotypes (phenotype group 5). A morphology‐based phenotype (phenotype group 3) and a phenotype of AD patients having eczema herpeticum (phenotype group 4) were investigated in 22 (12%) and 11 (6%) studies respectively. There was an overlap between phenotype categories in 26 studies, with two (*n* = 22) to three (*n* = 4) phenotype categories being investigated in one study. An overview of the study characteristics per study grouped per phenotype category can be found in Table S1a–e (Supporting Information).

### Risk of bias

The risk of bias of studies is reported in Table S2a–e (Supporting Information), demonstrating the qualitative results of the JBI critical appraisal checklists according to study design and phenotype category. We decided not to give an overall estimation of the risk of bias per paper but to descriptively report the checklist results per paper.

In various papers (30%, 27/91) within the disease severity category (phenotype group 1), no predefined scoring system or severity cut‐offs were reported, resulting in the score unclear for outcome in the risk of bias assessment.^8^, ^9^, ^10^ In many papers (27%, 15/56) on disease trajectories (phenotype group 2), age cut‐offs were unclear or it was unclear who assessed the age of onset (i.e. whether it concerned reports by patient, parent or physician).^11^, ^12^, ^13^ A lack of detail was identified regarding phenotypes based on morphological features and the investigation of morphological characteristics in other phenotypes (phenotype group 3 and 5). Often (in 69%, 41/59), it was unclear who performed the assessment or no criteria or further specifications for the assessment of morphological characteristics were reported (i.e. when characteristics were considered present or not).^14^, ^15^ Overall, in many cross‐sectional studies (60%, 84/140), the subjects and setting were not described in sufficient detail.^16^, ^17^, ^18^ In addition, the absence of inclusion of potentially confounding factors in the analyses of many studies (55%, 102/186) was noteworthy. A major source of bias across studies related to the two latter factors in the checklists.

### Quality of the evidence

We found heterogeneity in the phenotypes and investigated characteristics that were reported in studies and the results of this review are descriptive. Therefore, following discussions with author M.L., an international leading GRADE researcher, assessing the quality of the evidence with GRADE, was considered not relevant.

### Study results

**Figure 2:**
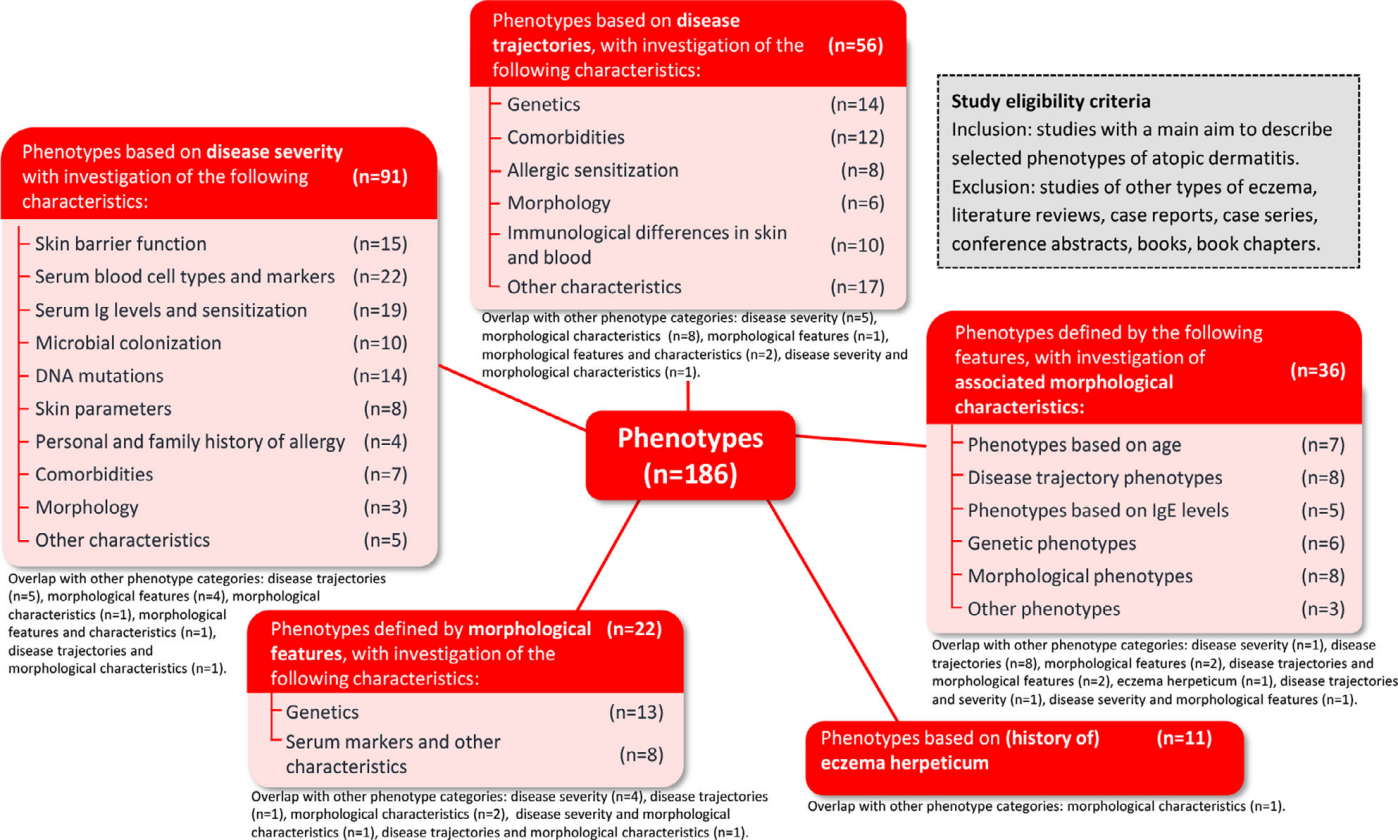
Phenotypes of atopic dermatitis and investigated associated characteristics (Graphical abstract).

An overview of all studies in alphabetical order per phenotype category and details of the results are found in Table S1a–e (Supporting Information). The results of the statistical analyses are summarized per phenotype category and per category of associated characteristics in Appendix S3 (Supporting Information). An overview of all phenotypic groupings and their investigated characteristics can be found in Fig. 2 (graphical abstract).

#### Phenotypes based on disease severity (phenotype group 1)

Within this phenotype category, 86% (78/91) of studies were cross‐sectional, 66% (60/91) was hospital‐based only, and in 2% (2/91), a data‐driven approach was used. In the studies, the number of included AD patients ranged from seven to 526 808. Regarding WHO region, the majority of studies were conducted in the European Region (*n* = 50, 55%), followed by the Western Pacific Region (*n* = 21, 23%), the Region of the Americas (*n* = 16, 18%) and the African Region and the Eastern Mediterranean Region (both *n* = 1, 1%). Investigated characteristics included the following categories: skin barrier function (*n* = 15),^19^, ^20^, ^21^, ^22^, ^23^, ^24^, ^25^, ^26^, ^27^, ^28^, ^29^, ^30^, ^31^, ^32^, ^33^ serum blood cell types and markers (*n* = 23),^9^, ^18^, ^23^, ^34^, ^35^, ^36^, ^37^, ^38^, ^39^, ^40^, ^41^, ^42^, ^43^, ^44^, ^45^, ^46^, ^47^, ^48^, ^49^, ^50^, ^51^, ^52^, ^53^ serum Ig levels and sensitization (*n* = 15),^20^, ^22^, ^25^, ^51^, ^54^, ^55^, ^56^, ^57^, ^58^, ^59^, ^60^, ^61^, ^62^, ^63^, ^64^ microbial colonization (*n* = 10),^15^, ^65^, ^66^, ^67^, ^68^, ^69^, ^70^, ^71^, ^72^, ^73^ DNA mutations (*n* = 14),^10^, ^51^, ^74^, ^75^, ^76^, ^77^, ^78^, ^79^, ^80^, ^81^, ^82^, ^83^, ^84^, ^85^ skin parameters (*n* = 8),^32^, ^86^, ^87^, ^88^, ^89^, ^90^, ^91^, ^92^ personal and family history of allergy (*n* = 4),^58^, ^93^, ^94^, ^95^ comorbidities (*n* = 7),^8^, ^51^, ^96^, ^97^, ^98^, ^99^, ^100^ morphology (*n* = 3)^51^, ^93^, ^101^ and other characteristics (*n* = 5)^51^, ^95^, ^102^, ^103^, ^104^ (see Table S1a and Appendix S3, Supporting Information).

The use of different scoring systems for determining disease severity were identified among the included studies (e.g. SCoring Atopic Dermatitis (SCORAD), Eczema Area and Severity Index (EASI)). The SCORAD was most frequently used (in 39 out of 91 studies). Strikingly, we found that even when the same scoring system was used, cut‐offs used to make a distinction between, for example, mild, moderate and severe AD differed between studies. For example, in studies, mild AD has been defined as SCORAD ranging from <15 to <37 points.^22^, ^33^, ^34^, ^66^ The threshold for severe AD ranged from >25 to >50 points.^25^, ^59^, ^79^ In addition, in many articles, no further specification of the basis of the severity definition was given.^10^, ^15^, ^38^, ^42^, ^44^, ^52^, ^68^, ^69^, ^89^, ^91^

#### Phenotypes based on disease trajectories (phenotype group 2)

Within this phenotype category, 50% (28/56) of studies was cross‐sectional, 57% (32/56) was hospital‐based only, and in 18% (10/56), a data‐driven approach was used. In the studies, the number of included AD patients ranged from nine to 108 703. Most studies were conducted in the European Region (*n* = 33/56, 59%). Thirteen studies (23%) were conducted in the Western Pacific Region, 9 (16%) in the Region of the Americas, and one (2%) in the South‐East Asian Region. Investigated characteristics included genetics (*n* = 14),^11^, ^17^, ^21^, ^51^, ^84^, ^105^, ^106^, ^107^, ^108^, ^109^, ^110^, ^111^, ^112^, ^113^ comorbidities (*n* = 12),^111^, ^112^, ^114^, ^115^, ^116^, ^117^, ^118^, ^119^, ^120^, ^121^, ^122^, ^123^ allergic sensitization (*n* = 8),^12^, ^63^, ^112^, ^124^, ^125^, ^126^, ^127^, ^128^ morphology (*n* = 6),^13^, ^114^, ^121^, ^129^, ^130^, ^131^ immunological differences in skin and blood (*n* = 10)^16^, ^21^, ^86^, ^91^, ^132^, ^133^, ^134^, ^135^, ^136^, ^137^ and other characteristics (*n* = 17)^111^, ^112^, ^114^, ^121^, ^124^, ^138^, ^139^, ^140^, ^141^, ^142^, ^143^, ^144^, ^145^, ^146^, ^147^, ^148^, ^149^ (see Table S1b and Appendix S3, Supporting Information).

Many studies which investigated phenotypes based on disease trajectories (including age of onset) and their associated characteristics were cross‐sectional studies (*n* = 28/56, 50%; with predefined phenotypes based on age cut‐offs), rather than longitudinal studies (*n* = 28/56, 50%; using for example statistical data‐driven approaches). Cross‐sectional studies investigating phenotypes based on disease trajectory have the potential of recall bias and a lack of information on temporality. However, in comparison with the other phenotype categories, longitudinal studies were predominantly seen within this category. Early‐onset disease was the most reported phenotype (*n* = 36/56, 64%). It predominantly concerned studies in adults retrospectively assessing self‐reported early onset of disease. These findings should be interpreted with caution as a previous study has reported that using the question ‘Have you had childhood eczema?’ to determine age of onset of AD leads to overestimation of the prevalence of childhood AD in adults.^195^ The age cut‐offs used diverged across studies. For example, early‐onset disease was defined using an age cut‐off that ranged from 3 months to 8 years. Therefore, use of the term early‐onset currently has limited informative value. Besides using age cut‐offs in the phenotype definition, inclusion of a maximum/minimum disease duration was added to the definition in five studies (e.g. early‐onset disease as within 6 months of disease onset).^86^, ^91^, ^114^, ^132^, ^137^

#### Phenotypes defined by morphological features, with subsequent investigation of associated characteristics (phenotype group 3)

Within this phenotype category, 95% (21/22) of studies was cross‐sectional, 73% (16/22) was hospital‐based only, and a data‐driven approach was used in none of the studies. In the studies, the number of included AD patients ranged from 21 to 2205. Regarding WHO region, 59% (13/22) of studies was performed in the Western Pacific Region and 41% (9/22) in Region of the Americas and/or the European Region. Thirteen studies investigated genetic characteristics,^105^, ^108^, ^150^, ^151^, ^152^, ^153^, ^154^, ^155^, ^156^, ^157^, ^158^, ^159^, ^160^ and eight studies investigated serum markers and other characteristics^14^, ^15^, ^22^, ^45^, ^46^, ^101^, ^161^, ^162^ (see Table S1c and Appendix S3, Supporting Information).

#### Phenotypes based on history of eczema herpeticum (phenotype group 4)

Within this phenotype category, 91% (10/11) of studies was cross‐sectional, 36% (4/11) was hospital‐based only (study setting was not reported in 6 studies). In addition, in none of the studies, a data‐driven approach was used. In the studies, the number of included AD patients ranged from 35 to 165 199. Nine (82%) of the studies were conducted in the Region of the Americas. One study (9%) was conducted in the European Region and one study (9%) in the Western Pacific Region. Various associated characteristics were investigated ^163^, ^164^, ^165^, ^166^, ^167^, ^168^, ^169^, ^170^, ^171^, ^172^, ^173^ (see Table S1d and Appendix S3, Supporting Information). Notably, in most studies, the diagnosis of (history of) eczema herpeticum was confirmed by either anti‐HSV antibody titer, PCR, Tzanck smear, immunofluorescence and/or culture test results. These are all objective assessments rather than a predefinition that could be subject to interpretation.

#### Phenotypes defined by any feature, with subsequent investigation of associated morphological characteristics (phenotype group 5)

Within this phenotype category 83% (30/36) of studies was cross‐sectional, 72% (26/36) was hospital‐based only, and in none of the studies, a data‐driven approach was used. Regarding WHO region, 47% (17/36) of studies was performed in the European Region, 28% (10/36) in the Western Pacific Region, 14% (5/36) in Region of the Americas, 3% (1/36) in the South‐East Asian Region and 3% (1/36) in the Eastern Mediterranean Region. In the studies, the number of included AD patients ranged from 31 to 6208. Various phenotype categories were investigated, including phenotypes based on age (*n* = 7),^93^, ^174^, ^175^, ^176^, ^177^, ^178^, ^179^ disease trajectory (*n* = 8),^12^, ^13^, ^114^, ^121^, ^129^, ^130^, ^131^, ^145^ IgE levels (*n* = 5),^126^, ^180^, ^181^, ^182^, ^183^ genetics (*n* = 6),^184^, ^185^, ^186^, ^187^, ^188^, ^189^ morphology (*n* = 8)^14^, ^101^, ^108^, ^131^, ^153^, ^163^, ^190^, ^191^ and other phenotypes (*n* = 3)^51^, ^192^, ^193^ (see Table S1e and Appendix S3, Supporting Information).

For phenotypes defined by morphological features and phenotypes defined by any feature with subsequent investigation of associated morphological characteristics, the study region may be relevant. Potential differences in AD morphology by study region have been reported.^196^ Therefore, the role of the region where the study took place should be considered. For studies investigating morphology including distribution of AD over the body surface, it became clear that, apart from the reporting of affected body parts, often no further specification was given at all. The reproducibility of these studies is questionable, as specific criteria are unclear. Research shows that variability exists in how people distinguish body parts.^197^ Further specification of how body parts are confined or when dermatitis was scored to be present (e.g. using size cut‐offs) would have contributed to the quality of these studies.

## Discussion

### Summary of evidence

We have undertaken a comprehensive analysis of the published literature on phenotype definitions used in literature and have described the characteristics associated with phenotypes. Phenotypes of patients with AD have been identified based on various features, including disease severity, disease trajectories, morphology and predisposition to eczema herpeticum. With this systematic review, we have gained insight on how these phenotype categories are reported in the literature, thereby contributing to developing a better understanding of AD. This systematic review highlights the heterogeneity that currently exists in the phenotyping of the AD population. In the literature, many phenotypes based on many features are described. At the present time, no consensus exists on how these phenotypes of AD should be defined, and the potential role of phenotypes in guiding both diagnostic and therapeutic management of patients is unknown.

Across the phenotype categories there were both differences and similarities in study characteristics. We identified mostly cross‐sectional studies (*n* = 141, 76%) in predominantly hospital‐based settings (*n* = 109, 59%). Hospital‐based studies could be subject to selection bias due to referral criteria, potentially leading to the identification of other phenotypes than when a population‐based approach was used. Therefore, hospital‐based studies need to be interpreted cautiously.^194^ Phenotypes based on disease severity were most frequently studied (*n* = 91, 49%). The methodological approach for investigating phenotypes differed between studies. Besides the predefinition of a subgroup of patients based on certain features (e.g. cut‐offs for age or severity), statistical data‐driven approaches were also used to identify phenotypes in the minority of studies (*n* = 13, 7%), for example, by using latent class analysis or cluster analysis. Though these data‐driven approaches are only used in a minority of studies aiming to investigate phenotypes, this can be considered a relatively unbiased way to identify phenotypes, in contrast to an approach using an investigator‐imposed predefinition. The most frequently used data‐driven approach is latent class analysis. Data‐driven approaches have the potential to identify patterns that are not obvious to clinical observation. Unfortunately, this only was performed in a small number of studies.

### Strengths and limitations

No previous systematic reviews were undertaken to map the current evidence on AD phenotypes in the literature. Librarians were involved in composing a comprehensive and broad search strategy. The protocol of this systematic review was published and preregistered. Moreover, we adhered to PRISMA guidelines in the reporting of this study.

Limitations include that since both the phenotype definitions and the a priori defined characteristics of interest differed between studies, we were unable to pool results and did not use GRADE to assess the quality of evidence. Accordingly, no meta‐analyses could be undertaken due to this heterogeneity in study outcomes, and therefore, we have reported on all studies separately in the evidence tables (Table S1a–e, Supporting Information) and Appendix S3 (Supporting Information). Meta‐bias resulting from publication bias or selective outcome reporting bias could not be assessed formally because of the qualitative nature of the study. However, both types of bias are deemed unlikely because of our rigorous search and descriptive nature of the studies. Studies were retrieved by our search when the term phenotype or synonyms of phenotype were specifically mentioned. In other words, studies that have used other terminologies (i.e. studies that describe phenotypes, but do not use the terminology phenotype or synonyms of phenotype) could have been missed. A bias for recent studies may have been introduced by the absence of these terminologies at inception of the used databases. Case reports and case series, for example, describing morphological phenotypes were excluded. Although we report associations between phenotypes and characteristics, these do not prove any causal relationship, and many are based on small sample sizes in hospital‐based populations and hence should be interpreted with caution due to the possibility of referral and selection bias. In context of the scoping nature of this systematic review, we did not restrict to a specific study setting, size or confounder adjustment. Lastly, because the term phenotype is used in numerous ways in the literature, we had to define phenotype for consistency, and in the context of this systematic review, we have defined phenotype as a subtype or subgroup of patients with AD. In the context of precision medicine, a semantic distinction with endo(pheno)types would be of interest. It was not feasible to include all potential phenotypic groupings in this study. Therefore, we were forced to make choices on which phenotypes to focus, which resulted in focussing only on the most clinically relevant phenotypes. Excluded phenotype categories include subgroups based only on age, gender, ethnic populations, presence of triggers, (allergic) comorbidities, immunology and genetics.

### Implications and recommendations for future research

At the moment, the therapeutic management of AD is generally not based on phenotypes that could reflect potentially relevant differences in characteristics between patients, with the exception of severity. In theory, these differences in phenotypes could be associated with variations in treatment outcome. In the context of personalized medicine, stratification according to phenotype would be of interest to enable investigation of which patients are likely to respond best to certain therapies. In order to facilitate comparative or pooled analyses across studies in the future, phenotypes should be uniformly defined and consistently used. Ideally, researchers should use the same definitions for AD phenotypes in research, similarly to using the same core outcome set for outcome measurements in clinical trials and clinical practice (www.homeforeczema.org/). This core outcome set already includes the recommendation of using the EASI to measure disease severity. A previous study has determined and recommended the following severity strata for EASI: 0: clear, 0.1–5.9: mild, 6.0–22.9: moderate and 23.0–72: severe.^198^ We should preferably use the same outcome measurements and cut‐offs to describe disease severity phenotypes. Regarding phenotypes based on disease trajectories, we ideally should use the same definitions, e.g. early‐onset disease, by using uniform age cut‐offs, when using non‐data‐driven approaches. However, first, we should get a clearer picture of the predictive ability of such cut‐offs. As for morphology, it would be desirable to develop (diagnostic) criteria for morphological phenotypes, as current diagnostic criteria for AD do not facilitate the identification of these or phenotypes in general.^199^, ^200^ The current heterogeneity in phenotyping of AD has demonstrated a need for international harmonization. More research using unbiased data‐driven approaches in well‐defined, population‐based settings should be considered to allow for the identification of phenotypes that are not obvious to clinical observation. Selection of appropriate data‐driven techniques should be guided by the nature of the dataset, e.g. whether it is cross‐sectional or longitudinal, and by the types of input available (disease activity, severity, clinical presentation etc.). To date, most cross‐sectional data‐driven techniques have been from the family of cluster analysis, and longitudinal data techniques have been from the family of mixture models such as latent class analysis. Phenotypes identified by a wide range of cross‐sectional data may be more richly characterized than phenotypes identified by a smaller range of fewer but longitudinally collected data, but their interpretation may be different. For example, cross‐sectional phenotypes may describe clinical AD presentation well but may be less suitable to track the persistence or resolution characteristics that longitudinal phenotypes characterize and vice versa. Whatever the method, the resulting phenotypes should be interpreted in context of the demographic characteristics (e.g. age, sex, ethnicity and geographical region) of the population represented by the sample used to derive them, i.e. not assumed to be applicable to populations not included in the sample. Phenotype studies should also be replicated in independent populations to investigate the stability of the identified phenotypes. In addition, it would be of interest to investigate phenotypes based on allergic comorbidities, since we apprehend AD as part of a larger group of diseases with TH2 inflammation skewing. Lastly, the identification of clinically meaningful phenotypes in the context of treatment outcome should be pursued, by investigating therapeutic effectiveness and safety in patients stratified according to phenotype.

## Conclusions

This systematic review has identified a lack in the uniform and consistent use of phenotypes of AD across studies. We have presented an overview of the phenotype definitions used in literature for disease severity, disease trajectory, morphology and eczema herpeticum. In addition, we describe characteristics reported to be associated with these phenotypes, and other phenotypes with subsequent investigation of associated morphological characteristics. Heterogeneity was observed in phenotype definitions used and in associated characteristics investigated within the same phenotypic grouping. Further research applying a consistent and uniform use of phenotype definitions and data‐driven data approaches are recommended. The identification of clinically meaningful phenotypes and insights into underlying endotypes has the potential to improve therapeutic strategies, by working towards personalized medicine and ultimately leading to the improvement of care for this condition.

## Supporting information


**Table S1**. Evidence tables per predefined phenotype category.

**Table S2**. Qualitative outcomes by the JBI critical appraisal checklists.

**Appendix S1**. Changes to the original protocol.

**Appendix S2**. Ovid MEDLINE search strategy.

**Appendix S3**. Summary of main results per phenotype category and per category of associated characteristics.

## Data Availability

The authors confirm that the data supporting the findings of this study are available within the article and its supplementary materials.
