# Supplementary material for: Classifying atopic dermatitis: a systematic review of phenotypes and associated characteristics
Source: J Eur Acad Dermatol Venereol. 2022 Feb 25;36(6):807–19. doi: 10.1111/jdv.18008 (PMC9307020; doi:10.1111/jdv.18008)
Supplement: Supplementary file 1 — Table S1. Evidence tables per predefined phenotype category. [file JDV-36-807-s005.zip › jdv18008-sup-0001-Table1a.docx]

**Table S1a. Evidence table for phenotypes based on disease severity**

| Study | Study design | Year | Setting | Country | WHO region | No. | Age -  Mean ± SD (range) | Sex – M/F, no. (%) | Phenotype description | No. (%) per phenotype | Potential associated characteristic(s) (of a priori interest) | Methodological approach | Inclusion of controls (Y (no.)/ N) |
| --- | --- | --- | --- | --- | --- | --- | --- | --- | --- | --- | --- | --- | --- |
| Addor 2012 | Cross-sectional | 2006-2007 | Hos | Brazil | Region of the Americas | 120 | NR (>0) | 54 (45) M / 66 (55) F | Mild, moderate, severe AD: based on Rajka and Langeland scale | Mild: n=34 (28.3), moderate: n=45 (37.5), severe: n=41 (34.2) | Corneometry, TEWL and IgE levels | Variance analyses, Tukey test | N |
|  | **Results of the analysis →** | Corneometry measurements ranged from 49.8 ± 9.3 (mean ± SD, mild), 37.3 ± 10.4 (moderate), to 29.7 ± 11.7 (severe). TEWL ranged from 5.8 ± 2.3 (mild), 10.2 ± 4.1 (moderate), to 15.2 ± 3.5 (severe). Significant difference between mean of corneometry, TEWL, and clinical severity (both P < 0.001). Means of IgE serum levels ranged from 142.4 UI/ml (mild), 310.48 (moderate), to 3644.22 (severe). Significant difference for IgE levels between mild and severe AD and moderate and severe AD (P < 0.001). No statistical difference for IgE levels between mild and moderate AD. | | | | | | | | | | | |
| Arima 2005 | Cross-sectional | NR | Hos | Japan | Western Pacific Region | 51 | 28.1 (21-36) | 13 (25) M / 38 (75) F | Mild, moderate, severe AD: severity classification described by Yoshiike et al. In addition, the grade of itching was evaluated, and patients with a total score of 1 to 4 were regarded as having mild, those with 5 to 8 as having moderate, and those with 9 to 12 as having severe AD. | NR | Beck Depression Inventory (BDI), Self-rating anxiety scale (SAS), Temperament and Character Inventory (TCI), Parental Bonding Instrument (PBI) | T-test, Bonferroni/Dunn’s multiple comparison | Y (51) |
|  | **Results of the analysis →** | Means (SD) of BDI were 8.44 (8.73) in mild AD, 6.55 (5.91) in moderate AD, and 19.40 (14.69) in severe AD. Significant difference between mild AD and severe AD, and between moderate AD and severe AD (P<0.05). Means (SD) of SAS were 33.68 (8.30) in mild AD, 33.00 (3.80) in moderate AD, and 41.50 (11.98) in severe AD. No significant differences between the groups. Neither PBI nor TCI showed any significant differences related to severity. | | | | | | | | | | | |
| Batmaz 2018 | Cross-sectional | 2015 | Hos | Turkey | European Region | 252 | 49.10 ± 46.56 mo (1 mo -17 y) | 133 (53) M / 119 (47) F | Mild, moderate, severe AD: based on SCORAD <25 mild, 25-50 moderate, >50 severe | Mild: n=120 (49.2), moderate: n=78 (32.0), severe: n=46 (18.8)  (severity missing n=8) | Neutrophil-lymphocyte ratio (NLR), platelet-lymphocyte ratio (PLR), mean platelet volume (MPV) | Student's t-test, Mann–Whitney U test | Y (75) |
|  | **Results of the analysis →** | No significant differences between the mean/median NLR, PLR, and MPV values among the groups: NLR: 0.94±0.73 for mild, 0.97±0.53 for moderate, 1.08±0.81 for severe; p=0.478. PLR: 80.12 (60.46-106.89, 85.54 (57.70-113.53), 79.38 (56.98-106.59); p=0.822. MPV: 9.05 (8.50-9.68), 8.80 (8.20-9.50), 9.00 (8.50-9.73). | | | | | | | | | | | |
| Benito 2016 | Cross-sectional | 2012 | Hos | Spain | European Region | 35 | NR | NR | Severe, moderate, mild AD in children: based on SCORAD severe ≥ 40, moderate 15–39 or mild <15 | Severe: n=13 (37), moderate: n=20 (57), mild: n=2 (6) | S. Aureus strain types isolated from nares/skin | No formal statistical test | N |
|  | **Results of the analysis →** | Methicillin-susceptible S. aureus strains of lineages CC45 and CC5 were detected in almost all cases in AD patients with severe AD, and lineages CC8 and CC30 in those with mild or moderate AD. | | | | | | | | | | | |
| Bergallo 2020 | Cross-sectional | NR | Hos | Italy | European Region | 23 | (2 mo-10 y) | (55) M / (45) F | Children with moderate-to-severe atopic dermatitis based on SCORAD (no further specification) | 100% | Expression of miRNA 155, FOXP3 and RORγ and Th17 / Treg ratio in blood (using PCR real time) | Student’s t test, Spearman’s test | Y (23) |
|  | **Results of the analysis →** | The over-expression of miR-155 is statistically significant (P=0.0040) in the group of patients with (moderate-to-severe) AD compared to the healthy control group. FOXP3 mRNA expression was statistically higher in AD (P=0.0057). The Th17 / Treg ratio is significantly smaller in AD (P=0.0012). Also the ratio miR-155/Th17/Treg is larger in AD (P=0.0002). No statistically difference between the two groups for RORγ mRNA. | | | | | | | | | | | |
| Biagini Myers 2020 | Cohort | 2016-2018 | Pop, hos | U.S.A. | Region of the Americas | 400 | Median (IQR): 2.3 (1.7-2.5) | 205 (51.3) M / 195 (48.7) F | Moderate-to-severe AD defined as SCORAD score of 25 or more | Moderate-to-severe: n=136 (34.1), mild: n=263 (65.9) | Lesional and nonlesional skin TEWL, host keratinocyte FLG expression, staphylococcal colonization, host keratinocyte S100A8 and S100A9 (alarmin) expression. Patterns of aeroallergen and food sensitization. | Wilcoxon or Kruskal-Wallis rank sum test, Chi-squared tests, multivariable logistic regression | N |
|  | **Results of the analysis →** | Moderate-to-severe AD is characterized by a significant decrease in nonlesional FLG expression (OR, 0.89; 95% CI, 0.81-0.97; P = .004) and significant increases in lesional TEWL (OR, 1.03; 95% CI, 1.01-1.04; P = .0002) and lesional S100A8 expression (OR, 1.25; 95% CI, 1.10-1.43; P =.0003). | | | | | | | | | | | |
| Bradley (also in trajectory category) 2002 | Cross-sectional | 1995-1997 | Hos | Sweden | European Region | 470 | Median 32 | 174 (37) M / 296 (63) F | Extreme AD (more severe phenotype): defined as having an early age of onset (at 2 years or younger), combined with a severity score of 3 or more. Severity scoring: Age at onset <2 years: 1, Hospitalization for AD: 1, Number of sites manifesting AD at examination: 0: 0, 1–3: 1, >3: 2, Raised total and/or allergen-specific serum IgE: 1. | Extreme AD: n=74 (16) | Genomic DNA markers | Genome-wide linkage analysis, linear model | N |
|  | **Results of the analysis →** | Linkage was found for the extreme phenotype and a region on chromosome 18p close to marker D18S542 (logarithm of the odds =1.88, P<0.005), to chromosome region 18q21 (D18S858; logarithm of the odds=1.67, and P<0.005), chromosome regions 21q21 (logarithm of the odds=1.69, P<0.005), 7p14 (logarithm of the odds=1.48, P<0.005) and chromosome region Xp11 close to marker DXS6800 (maximum logarithm of the odds score=2.64, P<0.005). | | | | | | | | | | | |
| Brandwein 2018 | Cross-sectional | 2015 | Hos | Israel | European Region | 35 | NR | NR | Severe AD, moderate AD using SCORAD (not further specified) | NR | 16S sequences (ribotypes) of AD-associated S. aureus on both lesional and nonlesional skin | NR | Y (10) |
|  | **Results of the analysis →** | Four unique full-length 16S rRNA S. aureus sequences, or ribotypes, were identified. Staphylococcus aureus ribotype 3 (i.e. 3a and 3b) was the only differentially abundant staphylococcal ribotype between moderate and severe AD skin on both the antecubital fossa and popliteal fossa. This ribotype was significantly more abundant in individuals with severe AD on both lesional and nonlesional skin (P<0.05). Comparison of the relative abundance of S. aureus ribotypes between healthy individuals and patients with moderate AD and severe AD on lesional and nonlesional skin, showed significant differences between severe (both lesional and nonlesional) and healthy skin and between moderate and severe nonlesional skin (P<0.05). | | | | | | | | | | | |
| Brown 2008 | Cross-sectional | 1996-2003 | Pop | U.K. | European Region | 195 | (7-9) | NR | Mild-to-moderate atopic eczema in childhood: based on Three Item Severity score (1-2 = mild eczema, 3-5 = moderate eczema, 6-9 = severe eczema) | Mild: n=70 (58), moderate: n=45 (38), severe: n=5 (4)  (severity missing n=75) | 5 FLG null mutations: R501X, 282del4, R2447X, S3247X, 3702delG | Fisher exact test, logistic regression analysis | Y (611) |
|  | **Results of the analysis →** | The combined null genotype (carriage of ≥1 FLG mutations) was significantly associated with atopic eczema (18.4% AD vs 12.9% controls, P = 1.2 x 10^-4). The odds ratio (OR) for individuals carrying 2 null mutations was 26.9 (95% CI, 3.3- 217.1), but heterozygote carriers showed no significant increase in risk (OR, 1.2; 95% CI, 0.7-1.9). | | | | | | | | | | | |
| Brunner (also in trajectory category) 2018 | Cross-sectional | NR | Hos | U.S.A. | Region of the Americas | C: 19 (A: 20) | C: 1.3 ± 1.2 (3-5) (A: 47.6 ± 14.5) | C: 11 (58) M / 8 (42) F (A: 12 (60) M / 8 (40) F) | Early-onset pediatric moderate-to-severe AD: defined as patients <5 years old with <6 months disease duration and based on SCORAD (mean 57.8 and range 33-84) | 100% of children | Transcriptional and barrier differences in skin | Mixed-effect models | Y (29) |
|  | **Results of the analysis →** | Pediatric early-onset moderate-to-severe AD also showed significant TH17/TH22 skewing but lacked the TH1 upregulation that characterizes adult AD. Pediatric AD exhibited relatively normal expression of epidermal differentiation and cornification products, which is downregulated in adults with AD. Some lipid-associated mediators (e.g., fatty acyl-CoA reductase 2 and fatty acid 2-hydroxylase) showed preferential downregulation in pediatric AD, and lipid barrier genes (FA2H and DGAT2) showed inverse correlations with TEWL. | | | | | | | | | | | |
| Chan 2018 | Cross-sectional | NR | NR | Taiwan | Western Pacific Region | 21 | 31.6 ± 3.3 (20-87) | 17 (81) M / 4 (19) F | Moderate-to-severe AD patients of Han Chinese descent | 100% | Molecular and cellular profiling of skin, skin biomarkers | Linear mixed-effect models | Y (21) |
|  | **Results of the analysis →** | Prominent hyperplasia (defined by epidermal thickness/ Ki671 cells/keratin 16 [K16] expression) was observed in lesional versus nonlesional AD and psoriasis and control skin (P <.01). Parakeratosis (a feature typifying European American psoriasis) was observed not only in 12 of 12 (100%) Chinese lesional psoriasis biopsies, but also in 21 of 21 (100%) lesional AD biopsies, versus none of the healthy control specimens. FLG showed interrupted expression in AD and psoriasis lesions without significant reduction of FLG mRNA. Loricrin (another differentiation protein) also showed skipped expression in lesional AD and psoriasis, and its mRNA expression was significantly downregulated in AD and psoriasis versus nonlesional skin (P < .01). CD3+ T cells and CD11c+ myeloid dendritic cells (DCs) characterized were increased in AD and psoriasis lesions versus controls. FcεR1+ cell infiltration was increased in both diseases but greater in AD versus psoriasis lesions (P < .001). Neutrophils were increased in both AD and psoriasis (P < .001), with greater increases in psoriasis (P < .1). Marked eosinophil infiltration characterized AD lesions versus psoriasis (P < .001) and uninvolved skin (P < .001). | | | | | | | | | | | |
| Clausen 2017 | Cross-sectional | 2013-2015 | Hos, other | Denmark | European Region | 101 | 35.7 ± 13.8 (19-77) | 43 (43) M / 58 (57) F | Mild, moderate, severe AD: based on SCORAD mild < 25, moderate 25–50, severe > 50 | Mild: n=31 (31), moderate: n=56 (55), severe: n=12 (12)  (severity missing n=2) | S. Aureus colonization: present/absent, stratified by site (lesional skin, non-lesional skin and nasal swab) | Fisher’s exact test | N |
|  | **Results of the analysis →** | SCORAD correlated with S. aureus colonization: significantly higher colonization prevalence in patients with moderate and severe disease than in those with mild disease, in lesional skin (66% vs 40%, P = 0.006), nonlesional skin (40% vs 3%, P < 0.001) and the nose (67% vs 31%, P < 0.001). | | | | | | | | | | | |
| Czarnowicki 2015 | Cross-sectional | NR | NR | U.S.A | Region of the Americas | 42 | 42 ± 2.1 (18-74) | 24 (57) M / 18 (43) F | Severe AD patients: based on a mean SCORAD of 65 | 100% | TH1, TH2, TH9, TH17, and TH22 T-cells and corresponding CD81 T-cell subsets in both cutaneous lymphocyte antigen (CLA)–positive and CLA- T-cell subsets | ANOVA, multivariate U-statistics | Y (25) |
|  | **Results of the analysis →** | Increased TH2/TC2/IL-13+ and TH22/TC22/IL-22+ populations (P <.1) were measured in patients with severe AD versus control subjects, with significant differences in CLA+T-cell numbers (P < .01). A significantly lower frequency of CLA+ IFN-g–producing cells was observed in patients with AD, with no significant differences in CLA-T-cell numbers. The CLA+TH1/TH2 and TC1/TC2 ratio was highly imbalanced in patients with AD (10 vs 3 [P =.005] and 19 vs 7 [P < .001], respectively). | | | | | | | | | | | |
| de Bruin-Weller 2020 | Cross-sectional | NR | Hos, GP | Canada, France, Germany, Italy, Spain and the United Kingdom | Region of the Americas, European Region | 1,467 | (18–65) | 656 (45) M / 811 (55) F | Mild, moderate, severe AD based on Investigator’s Global Assessment | Mild: n=547 (37.3), moderate: n=520 (35.4), severe: n=400 (27.3). | Patient-reported burden: questionnaire that included pruritus and pain numerical rating scales, Patient-Oriented-Scoring of Atopic Dermatitis (PO-SCORAD) itch and sleep visual analogue scales, Dermatology Life Quality Index (DLQI), the Hospital Anxiety and Depression Scale (HADS). Comorbidities. | Z-tests, paired-sample t-tests | N |
|  | **Results of the analysis →** | Compared with moderate and mild, participants with severe AD had more comorbidities, higher itch and pain severity, worse sleep and higher levels of anxiety and depression (all P < 0.001). Mean ± SD DLQI score among participants with severe AD (16.2 ± 6.9) showed a large effect on quality of life that was higher than those with moderate (10.2 ± 6.3) and mild (5.5 ± 4.9) (both P < 0.001). | | | | | | | | | | | |
| deOliveiraTitz 2016 | Cross-sectional | NR | NR | Brazil | Region of the Americas | 41 | 30.57 ± 11.23 (19-62) | 25 (61) M / 16 (39) F | Mild, moderate, severe AD in adults: according to EASI (no further details) | Mild: n=12 (29), moderate: n=19 (46), severe: n=10 (24) | IgE levels, eosinophils (LIN1-CCR3+) | Mann–Whitney or Kruskal–Wallis with Dunn’s post-test | Y (45) |
|  | **Results of the analysis →** | Increasing IgE levels were significantly correlated with increasing AD severity (P ≤ 0.0001). Significant higher frequency of eosinophils (LIN1-CCR3+) in AD, related to disease severity (P ≤ 0.05). | | | | | | | | | | | |
| Dworzak 1999 | Cross-sectional | NR | Hos | Austria | European Region | 36 | Mild AD: median: 5.8 (2.5-31.4)  Severe AD: 10.7 (0.5-27) | NR | Severe vs mild AD, according to extent of skin involvement (estimated using rule of nines) and total clinical severity grading according to Rajka, considering 6 parameters (erythema, edema/ papulation/ induration, pruritus, excoriations/ erosions, scaling/ dryness, and lichenification) on a 0.5 point incremental scale from 0 to 3: 1, mild; 2, moderate; and 3, severe. Mild: score ≤6 (median: 3.5), involving at most 20% of the skin. Severe AD: score > 7 (median: 10), involving at least 20% of skin. | Mild: n=21 (58), severe: n=15 (42) | Peripheral blood T cell subpopulations | Wilcoxon 2-sample test | Y (23) |
|  | **Results of the analysis →** | No differences among the groups with respect to the general proportions of T cells (CD3+), CD4+ T cells, CD8+ T cells, B cells (CD19+), NK cells (CD16+CD3–), CD103+ T cells, and CD25+ T cells among total circulating lymphocytes (CD3+ plus CD19+ plus CD16+). Slightly more CD4+ memory cells and clearly more HLA-DR+ T cells in patients with severe AD (P<0.05) in compared to controls. Severe AD had a significantly expanded proportion of  CLA+ T cells (P = .024) and CLA+/CD4+ T cells (P = .006) but similar proportions of CLA+/CD8+ T cells compared with control subjects. Severe AD also had distinctly more HLA-DR+/CLA+ T cells than control subjects (P = .005). Similar alterations were seen in patients with mild AD, but these were not statistically significant. After correction for age, all differences were significant only in probands less than 10 years of age. | | | | | | | | | | | |
| Dyjack 2018 | Cross-sectional | NR | Hos | U.S.A. | Region of the Americas | 30 | 36.3 ± 2.2 | 18 (60) M / 12 (40) F | Adult white patients with active AD and mild, moderate and severe disease based on IGA: mild: IGA= 2, moderate: IGA = 3, severe: IGA = 4) | Mild: n=7 (23), moderate: n=17 (57), severe: n=6 (20) | Gene expression profiling for a type 2 inflammatory signature (656 genes) in skin tape strips and biopsies | Cochran-Armitage trend test | Y (25) |
|  | **Results of the analysis →** | Principle components 2 values (type 2 inflammatory signature (type 2-high endotype)) were positively correlated with severity, with a median value of -6.94 for mild, -2.00 for moderate, and 5.19 for severe disease (P = 5.12 x 10^-2). | | | | | | | | | | | |
| Ekelund 2008 | Cross-sectional | 1995-1997 | Hos | Sweden | European Region | 921 | 29 | 368 (40) M / 553 (60) F | Severe eczema phenotype: based on arbitrary severity score ≥ 4 in a Swedish population. Severity score: Age at onset ≤ 2 years (1 point), Hospitalization for eczema (1 point), Affected sites on examination (0 = 0 points, 1-3 = 1 point, >3 = 2 points), Raised total and/or allergen-specific IgE (1 point). | Severe eczema: n=139 (15) | FLG mutations R501X and 2282del4 | Pedigree Disequilibrium Test | N |
|  | **Results of the analysis →** | OR for R501X of 5.29 (3.32–8.84) p=1.3×10–12, OR for 2282del4 of 6.34 (1.90–21.2) p=1.5×10–7, OR for the combined allele of 4.73 (1.98–11.29), p = 3.6×10–8, was found for the subgroup with a severe eczema phenotype. As to FLG null carriers, 67% of homozygote carriers had severe eczema, compared to 42% of heterozygotes and 16% of wild type carriers. | | | | | | | | | | | |
| Ercan 2013 | Cross-sectional | 2010 | Hos | Turkey | European Region | 49 | 4.9 ± 3.6 | 26 (53.1) M / 23 (46.9) F | Mild, moderate, severe AD in Turkish children: based on SCORAD: mild <15, moderate 15-40, severe >40. | Mild: n=21 (43), moderate: n=14 (29), severe: n=14 (29) | Serum immunoglobulin (Ig) levels: IgA and IgG z scores | Mann-Whitney U test, Kruskal-Wallis test | Y (50) |
|  | **Results of the analysis →** | The IgG z scores were statistically significantly higher, whereas IgA z scores were statistically significantly lower in patients with severe AD compared to those with mild and moderate AD. IgA z score: mild vs moderate: p=.19, moderate vs severe: p=.015, mild vs severe: p<0.001; all groups: p<.001. IgG z score: mild vs moderate: p=.47, moderate vs severe p=.027, mild vs severe p=.001; all groups: p=0003. | | | | | | | | | | | |
| Flohr (also in trajectory category) 2010 | Cross-sectional | 2009-2010 | Pop | U.K. | European Region | 29 | 3 mo | NR | Early-onset eczema at 3 months of age and with mild or moderately severe disease: based on SCORAD < 15 or ≥ 15 | SCORAD < 15: n=23 (79), SCORAD ≥ 15: n=6 (21) | TEWL, FLG mutations: R501X, 2282del4, R2447X and S3247X | Mann–Whitney U-test | Y (59) |
|  | **Results of the analysis →** | TEWL was higher in children with early-onset eczema compared with unaffected infants (median TEWL 14.24 vs. 11.24, P < 0.001). Higher TEWL was associated with more severe disease (median TEWL, SCORAD < 15, 13.1 vs. 29.6, SCORAD ≥ 15, P = 0.029). FLG mutation carriers were more likely to have early-onset eczema (OR 4.26, 95% CI 1.34–13.57, P = 0.014). | | | | | | | | | | | |
| Flohr (also in morphology phenotype) 2014 | Cross-sectional | 2009-2012 | Pop | U.K. | European Region | 154 | 3 mo | NR | Exclusively breastfed 3-month–old infants with mild (SCORAD<20) or moderate-to-severe (SCORAD≥20) AD and flexural (either around the eyes, neck, antecubital, and popliteal fossae or ankles) or non-flexural distribution | Mild: 132 (85.7),  moderate-to-severe:  n=22 (14.3).  Flexural AD only: n=44 (29), non-flexural AD only:  n=36 (23), flexural and non-flexural: n=74 (48). | TEWL, skin prick testing results to six study foods (cow’s milk, egg, cod, wheat, sesame, peanut). | Mann–Whitney U-test | Y (465) |
|  | **Results of the analysis →** | Higher TEWL was associated with more severe disease (median TEWL ‘SCORAD <20’ 14.7 (IQR, 12.2–18.8) versus ‘SCORAD≥20’ 26.4, (IQR, 15.4–39.7, P=0.001). There was also a strong association between food sensitization and AD severity (adjusted OR_SCORAD_<_20_=3.91, 95% CI: 1.70–9.00, P=0.001 vs. adjusted OR_SCORAD≥20_=25.60, 95% CI: 9.03–72.57, P<0.001). An association was also found for egg sensitization (adjusted OR_SCORAD_<_20_=5.59, 95% CI: 2.00–15.61, P=0.001 vs. adjusted OR_SCORAD≥20_=41.38, 95% CI: 12.75–134.35, P<0.001), cow’s milk sensitization (adjusted OR_SCORAD_<_20_=6.04, 95% CI: 1.30–28.08, P=0.02 vs. adjusted OR_SCORAD≥20_=26.55, 95% CI: 4.84–145.61, P<0.001) and peanut sensitization (adjusted OR_SCORAD_<_20_=2.39, 95% CI: 0.43–13.36, P=0.32 vs. adjusted OR_SCORAD≥20_=13.65, 95% CI: 2.28–81.62, P=0.004) separately. Regarding food sensitization risk (skin prick test ≥1mm), no significant difference was found for AD phenotype (no AD vs. flexural vs. non-flexural vs. both flexural and non-flexural) after adjusting for FLG, sex and AD severity. | | | | | | | | | | | |
| Foley (also in morphology characteristics category) 2001 | Cross-sectional | 1998-1999 | Pop, Hos | Australia | Western Pacific Region | 346 | (0-5) | 178 (51) M / 168 (49) F | Minimal, mild, moderate, severe AD severity in preschool-age Australian children: severity was graded as minimal (disease that the parent may not have been aware of and that would require no treatment or would respond well to simple emollients available without prescription), mild (disease that might require attention from a medical practitioner with the use of minimal prescription-only treatments), moderate (disease that would require attention from a medical practitioner, plus the use of more potent prescription-only topical steroids as well as emollients), and severe (disease that requires management by a dermatologist) | Minimal: (4.9), mild: (58.8), moderate (34.5), severe (1.7) | (Number of) affected sites, personal history of asthma or hay fever or a family history of atopy | Unclear: no (reporting of) formal statistical test | Y (770; unclear if included in analyses) |
|  | **Results of the analysis →** | With increasing severity, more sites were likely to be affected. The severity did not correlate with a personal history of asthma or hay fever or a family history of atopy. Flexures were affected in 72.5% of children with AD. Face: 60.1%, trunk: 38.2%, limbs: 37.6%, diaper area: 7.2%, all other sites: 4.9%. Only 20 (5.8%) of all children with AD did not have face or flexural involvement. | | | | | | | | | | | |
| Furue 2012 | Cross-sectional | 2010 | Other | Japan | Western Pacific Region | 66 | 35.7 (20–57) | 18 (27) M / 48 (73) F | Mild AD: defined ad serum TARC levels below 700 pg/mL | 100% | Serum TARC levels and epidermal barrier function by TEWL and stratum corneum hydration (SCH) | Mann–Whitney U-test, chi-squared test | Y (121) |
|  | **Results of the analysis →** | Significantly elevated serum TARC levels (median 214.5 (95% CI 186-250), p=0.032) and TEWL values (median 21.9 (95% CI 19.2–26.4), p<0.001) and significantly decreased SCH values (median 64.5 (95% CI 48.7–81.9), p<0.001) were detected in the mild AD group compared to those in the healthy group (median (95% CI): 177 (165–198), 16.9 (15.5–18.1) and 106.4 (94.0–122.7), respectively. | | | | | | | | | | | |
| Galli 2015 | Cross-sectional | 2012-2013 | Hos | Italy | European Region | 89 | 68 mo (6-195 mo) | 48 (53.9) M / 41 (46.1) F | Moderate (SCORAD 25-50), severe (SCORAD>50), mild (SCORAD<25) chronic eczema in children | Mild: n=66 (74.2), moderate: n=17 (19.1), severe: n=6 (6.7). | 25-Hydroxyvitamin D serum levels | Student’s T test | N |
|  | **Results of the analysis →** | Mean vitamin D concentrations in patients with moderate eczema (34.9 ng/ml ± 20,0) and severe eczema (78.3 ng/ml ± 71,0) were not statistically different from concentrations detected in patients with mild eczema (49.06 ng/ml ± 40,2) (p<0.05). | | | | | | | | | | | |
| Galli 2020 | Cross-sectional | 2015-2016 | Hos | Italy | European Region | 371 | 5.95 ± 3.72 | (57) M / (43) F | Five AD phenotypes in preschoolers (aged ≤5 years) (“moderate-severe AD, high comorbidity”; “moderate-severe AD, low comorbidity”; “mild AD, low comorbidity”; “mild AD, respiratory comorbidity”; “mild AD, food-induced comorbidity) and four AD phenotypes in schoolchildren (aged 6-14 years) (“moderate-severe AD, high comorbidity”; “moderate-severe AD, low comorbidity”; “mild AD, low comorbidity”; “mild AD, respiratory comorbidity”). | Preschoolers: “moderate-severe AD, high comorbidity”: n=16 (8%); “moderate-severe AD, low comorbidity”: n=73 (35%); “mild AD, low comorbidity”: n=42 (20%); “mild AD, respiratory comorbidity”: n=65 (32%); “mild AD, food-induced comorbidity”: n=10 (5%);  Schoolchildren: “moderate-severe AD, high comorbidity”: n=40 (24%); “moderate-severe AD, low comorbidity”: n=17 (10%); “mild AD, low comorbidity”: n=27 (16%); “mild AD, respiratory comorbidity”: n=81 (49%). | Sociodemograpic characteristics and individual and environmental factors | Data-driven approach to identify phenotypes by using latent class analysis.  Analysis of variance, Fisher exact test. | N |
|  | **Results of the analysis →** | Parental history of asthma and eczema, early day-care attendance, and exposure to molds were significantly associated with the “moderate-severe AD, high comorbidity” phenotype in preschool children (P<0.05). The “moderate-severe AD” phenotypes were also associated with the highest burden in terms of medication use and limitations in daily activities. No significant associations were observed in schoolchildren. | | | | | | | | | | | |
| Gayret 2019 | Cross-sectional | 2015-2016 | Hos | Turkey | European Region | 79 | 15.04 ± 11.90 mo (3-27 mo) | 49 (62) M / 30 (38) F | Children with AD and SCORAD:< 25 was classified as mild, 25 - 50 was classified as moderate, and> 50 was classified as severe | Mild: n=46 (58), moderate n=25 (32), severe: n=8 (10). | Platelet parameters (mean platelet volume (MPV), platelet distribution width (PDW)], neutrophil-lymphocyte ratio (NLR), platelet-lymphocyte ratio (PLR). | Mann-Whitney U test, Chi-squared test, Fisher’s exact test, Kruskal-Wallis test, logistic regressions. | Y (75) |
|  | **Results of the analysis →** | In the severe AD group, MPV was higher (7.62±1.81 vs 6.64±1.16, p=0.035) and PDW was lower than in the mild AD group (16.52±1.49 vs 17.93±1.44, p=0.0001). No significant differences for the other parameters. To determine the factors that most affected by SCORAD scores, logistic regression analysis was performed with MPV and PDW. This showed that the PDW level was the factor that most affected SCORAD in AD patients | | | | | | | | | | | |
| Hallau 2016 | Case-control | NR | Hos | Germany | European Region | 281 | Median (IQR): 37 (27-48) | 121 (43) M / 160 (57) F | Severe or mild/moderate AD in adults: based on SCORAD: severe: >40, mild/moderate: 9-40 | Mild/moderate: n=126 (45), severe: n=155 (55) | Frequencies of 6 SNPs in genes encoding vitamin D synthesizing enzyme Cyp27b1 or inactivating enzyme Cyp24a1 | Chi-squared test, Cochrane-Armitage trend test, Whitney-Mann U test, Kruskal–Wallis test | Y (278) |
|  | **Results of the analysis →** | Cyp24a1 rs2248359-major C allele significantly over-represented in patients with AD compared with healthy controls (OR 2.10, 95% CI 1.1-4.1, p=0.03), which was more pronounced in patients with severe AD (OR 3.5, 95% CI 1.4-8.8, p=0.008). Haplotype rs2248359T, rs2296241A (Cyp24a1-TA) was more frequent in healthy individuals than in severe AD patients (p = 0.005). Haplotype Cyp24a1-CA was associated with severe AD (p=0.003) in comparison to healthy controls. Haplotype TCGC (rs703842T, rs10877012C, rs3782130G, rs4646536C) was found more in healthy controls than in patients with severe AD (p = 0.029 ). | | | | | | | | | | | |
| Holm (in trajectory, severity and morphological characteristics group) 2019 | Cross-sectional | 2012-2017 | Hos | Denmark | European Region | 470 | 18.7 ± 16.5 | 214 (45.5) M / 256 (54.5) F | AD subgroups based on SCORAD: mild (< 25), moderate (25–50) and severe (> 50); small children (< 4 years of age), children/adolescents (age 4–15 years) and adults (> 15 years of age); early-onset (< 1 year of age), late-onset (>1 year of age) | Small children: n=122 (26), children/adolescents: n=103 (22) and adults: n=245 (52).  Mild: n= 166 (35.3), moderate: n= 218 (46.4), severe: n= 86 (18.3).  Early-onset: n=141 (47.8), late-onset: n=154 (52.2). | FLG mutations (R2447X, R501X and 2282del4), serum total IgE, blood eosinophil count; self-rated health, eczema distribution in the past month | ANOVA, chi-squared test, independent t-test | N |
|  | **Results of the analysis →** | A significant difference between severity groups in small children was observed for FLG mutation carrier status (16.7 vs. 30.2 vs. 60.0% mutation carriers among patients with mild, moderate and severe AD, respectively, p = 0.012) and self-rated health (3.2 vs. 2.7 vs. 2.8 with 4 being excellent health, p = 0.022).  In the subgroup of children/adolescents, disease severity was statistically significantly associated with CDLQI (5.6 vs. 7.3 vs. 8.8 among patients with mild, moderate and severe AD, respectively, p = 0.048). A significant difference between severity groups in adults was observed for male sex (24.4 vs. 39.8 vs. 52.9%, p = 0.003), serum total IgE (577 vs. 1269 vs. 2379 × 103 IU/L, p < 0.001), blood eosinophil count (0.28 vs. 0.39 vs. 0.61 × 109/L, p < 0.001) and asthma (42.9 vs. 38.8 vs. 72.0%, p < 0.001), hand eczema (66.2 vs. 81.6 vs. 88.0%, p = 0.007), flexural eczema (67.5 vs. 74.7 vs. 90%, p = 0.015), and DLQI (6.9 vs. 9.9 vs. 13.8, p < 0.001). Compared to no early onset of AD and no FLG mutations, early onset of AD and FLG mutation was associated with more severe disease (higher mean SCORAD (33.3 vs. 41.6, p = 0.012)) and high serum total IgE levels (903 vs. 1961 × 103 IU/L, p = 0.047). | | | | | | | | | | | |
| Ibrahim 2012 | Cross-sectional | NR | Hos | Egypt | Eastern Mediterranean Region | 25 | 6.9 ± 3.5 (3-14) | 13 (52) M / 12 (48) F | Severe, moderate, mild AD in Egyptian patients: according to SCORAD: mild ≤15, moderate = 15-40, severe ≥ 40 | Mild: n=4 (16), moderate: n=16 (64), severe: n=5 (20) | SNPs in the interleukin (IL)-18 gene: -137 G/C [rs187238], and -140 C/G [rs360721] | Pearson chi-squared-test, Fisher’s exact test | Y (50) |
|  | **Results of the analysis →** | The -140 GG genotype and the -140 G allele were more often associated with severe AD compared with mild and moderate phenotypes (p = 0.01 and 0.001, respectively; odds ratios [ORs], 14.25 [95% CI, 1.48 –143.2] and 16 [95% CI, 2.8 –90.46], respectively) and were more frequent in severe AD compared with controls (p = 0.02; OR, 11 [95% CI, 1.27–95.2] and p = 0.006; OR, 10.2 [95% CI, 1.94–54.5], respectively). Neither the -137 G allele nor the GG genotype showed any significant difference among AD severity subgroups (p = 0.069 and 0.09, respectively) or when comparing each phenotype separately with the control group (p>0.05). | | | | | | | | | | | |
| Jenerowicz 2007 | Cross-sectional | NR | Hos | Poland | European Region | 30 | 24.5 (8-60) | 8 (27) M / 22 (73) F | Mild-to-moderate and severe-to-very-severe: based on the W-AZS index as proposed by Silny: W-AZS value < 50 points: mild and moderate AD and W-AZS value ≥ 50 points: severe and very severe AD | Mild-to-moderate: n=17 (57), severe-to-very-severe: n=13 (43) | Absolute eosinophil count and mean eosinophil percentage (of total white cell count) | ANOVA analysis with post-hoc Newman-Keuls tests | Y (60) |
|  | **Results of the analysis →** | No statistically significant difference between mean eosinophil numbers in mild and moderate AD (288 ± 196.4), and severe AD (292.3 ± 225.3 eosinophils/mm³). No statistically significant difference between the mean eosinophil percentage in the differential white cell count: 5.3 ± 4.4% in mild and moderate AD , and 7.7 ± 6.9% in severe AD. | | | | | | | | | | | |
| Johnson 1974 | Cross-sectional | NR | Hos | U.S.A. | Region of the Americas | 58 | 3 or younger: n=15; 4 to 13: n=20; 14 to 18: n=9; 19 or older: n=14 | NR | Mild and severe AD: defined as mild: minimal and limited in distribution; severe: moderate or severe dermatitis of limited distribution and generalized dermatitis or pruritus. | Mild: n=26 (45), severe: n=32 (55) | Total serum IgE concentration elevation (defined as > 1,000 ng. per milliliter) | Chi-squared test | N |
|  | **Results of the analysis →** | Elevated IgE levels were more frequent in severe AD (21/32, 65.6%), in comparison with mild AD (4/26, 15.4%) (x2 = 14.7640, p < 0.0005) | | | | | | | | | | | |
| Jung 2014 | Cross-sectional | NR | NR | Korea | Western Pacific Region | 42 | 19.1 ± 11. | 21 (50) M / 21 (50) F | Mild AD, moderate-to-severe AD: based on EASI score: mild AD (EASI score <15) and moderate-to-severe AD (EASI score ≥15) | Mild: n=21 (50), moderate-to-severe: n=21 (50) | Skin barrier function (basal TEWL, SC hydration and skin surface pH), amount of pyrrolidone carboxylic acid (PCA) and caspase-14 in corneocytes | Two-tail and unpaired Student’s t-tests, Mann–Whitney test | Y (31) |
|  | **Results of the analysis →** | In both mild AD patients (p=-.0008) and moderate-to-severe AD (p<.0001) patients, the basal TEWL was significantly increased in lesional skin compared to nonlesional skin. The basal TEWL in the non-lesional skin of the mild AD patients was higher than that of the normal healthy controls (p=0.0031). Both mild AD patients (p<.0001) and moderate-to-severe AD patients (p<.0001) showed decreased SC hydration in lesional skin compared to non-lesional skin. The moderate-to-severe AD patients also had significantly decreased SC hydration in non-lesional skin compared to that of the normal healthy controls (p=0.0207). Skin surface pH on the lesional skin of the moderate-to-severe AD patients (p=.0149), but not of mild AD patients, was significantly higher than that on non-lesional skin. Lesional skin of the mild AD patients (p=0.007) as well as the moderate-to-severe AD patients (p=0.007) had significantly decreased amounts of PCA compared to non-lesional skin. The amount of PCA in the non-lesional skin of the moderate- to- severe AD patients, unlike that of the mild AD patients, was significantly less than that of the normal healthy controls (p=0.0172). The amount of caspase-14 in non-lesional skin of AD  patients was significantly lower in the moderate-to-severe AD patients than that of the normal healthy controls (p=0.0066). | | | | | | | | | | | |
| Kaga (also in morphology phenotype category) 2011 | Cross-sectional | NR | Hos | Japan | Western Pacific Region | 56 | (21-56) | 31 (55) M / 25 (45) F | Adult patients with head and neck AD of different severities: mild, moderate and severe (not further defined) | Mild: n=21 (38), moderate: n=18 (32), severe: n=17 (30) | Extent and diversity of Malassezia colonisation: nine human-associated Malassezia species. | Kruskal-Wallis test | Y (32) |
|  | **Results of the analysis →** | A significant difference was found among all combination groups for the extent of total Malassezia colonisation in comparison to controls (P < 0.05). In severe AD, Malassezia colonisation was approximately two- to fivefold that in mild and moderate AD patients and healthy individuals. The two major microbiota, M. globosa and M. restricta, accounted for more than 80% of all Malassezia colonisation in AD patients of all severities, but their proportions differed with severity. In the mild and moderate patients, M. restricta was predominant over M. globosa (P < 0.05), whereas the proportions of M. globosa and M. restricta were almost identical (P > 0.05) in the severe patients. No difference in species diversity related to the severity of AD in the patients was found. The number detected was similar (3.5–4.2 species per case) among the members of all severity groups. | | | | | | | | | | | |
| Kayserova 2012 | Cross-sectional | NR | Hos | Czech Republic | European Region | 94 | 3.12 mo (0.5-27 mo) | 62 (66) M / 32 (34) F | Severe AD in children: based on SCORAD 50-80 | 100%  (data from 74 patients were available) | Genotype and haplotype frequencies of SNPs of 13 selected cytokine/receptor genes | Chi-squared test | Y (103) |
|  | **Results of the analysis →** | Genotypes of 7 polymorphisms—IL-4 -1098G/T and -590C/T, IL-6 -174C/G and nt565A/G, and IL-10 -1082A/G, -819C/T, and -592A/C were significantly associated with severe AD in comparison with controls (P<.05). The haplotype IL-4 GC was higher in controls (12.8%) than in patients (5.62%) (OR=0.41, P=.01). At the same time, the haplotype TNF– AA was lower in patients (0%) than in controls (3.47%) (P=.01). | | | | | | | | | | | |
| Kezic 2011 | Cross-sectional | NR | Hos | Ireland | European Region | 96 | 8.5 ± 4.2 | 54 (59) M / 42 (41) F | Moderate-to-severe AD patients with known FLG mutations (ADFLG: FLG+/- or -/-) and those wild type for FLG mutations (ADNON-FLG: (+/+). Severity based on Nottingham Eczema Severity Score (NESS): median (range): 12 (6–15); SCORAD: median (range): 13 (0–36 | FLG+/+: n= 40 (42), FLG+/-: n=37 (39), FLG-/-: n=19 (20) | FLG degradation products: pyrrolidone carboxylic acid (PCA), urocanic acid (UCA), histidine (HIS) and tyrosine. NMF in SC, defined as the sum of PCA and UCA. | Shapiro–Wilk’s  Test, analysis of variance  (anova), post hoc Tukey analysis, Student’s one-tailed test, multiple regression analysis | Y (24) |
|  | **Results of the analysis →** | Levels of PCA, UCA and HIS correlated with FLG genotype, with the lowest values for the FLG-/-group (p<0.001). Levels of all NMF components tended to be higher in the control group when compared to patients without FLG mutations (NON-FLG: FLG+/+). For UCA, PCA + UCA and PCA + UCA + HIS; this difference was statistically significant (p<0.05). The lowest values were found for the FLG-/-group (p<0.001, for comparison with FLG+/+ group). Multiple regression analysis showed that NMF levels were independently associated with FLG genotype and severity of disease. | | | | | | | | | | | |
| Kou (also in morphology phenotype category) 2014 | Cross-sectional | NR | Hos | Japan | Western Pacific Region | 257 | Median: 33, IQR: 17 | 149 (58) M / 108 (42) F | Adult patients with mild, moderate, severe or very severe AD, according to Japanese guidelines: skin involvement with mild  eruption alone (mild); eruption with severe inflammation of  < 10% of the skin surface area (moderate); severe eruption, with 10–30% skin involvement (severe); severe eruption with > 30% skin surface area involvement (very severe). Further classification into: erythroderma type; widespread combi-nations of various types; prurigo type; limbs type; and head/face/neck/chest/back type. | Mild: n=42 (16), moderate: n=95 (37), severe: n=74 (29) and very severe: n=46 (18).  Erythroderma-type: n=44 (17), widespread-type: n=147 (57), prurigo-type: n=33 (13), limbs-type: n=11 (4) and head/face/neck/chest/back-type: n=22 (9). | Serum periostin levels | Mann–Whitney U-test, Wilcoxon t-test | Y (91) |
|  | **Results of the analysis →** | Patients with severe or very severe AD, had considerably higher levels of periostin than those with mild or moderate disease [mild: 104.0 (82.3–157.8) ng mL-1; moderate: 122.0 (90.0–179.0) ng mL-1; severe: 157.5 (120.3– 236.8) ng mL-1; very severe: 241.0 (163.8–371.3) ng mL-1; (p<0.01). No significant difference in periostin level between patients with AD with mild and moderate disease. Patients with erythroderma-type AD, followed by widespread-type AD, had significantly higher levels of periostin compared with the other groups (P < 0.01). | | | | | | | | | | | |
| Lacy 2009 | Cross-sectional | NR | Hos | U.K. | European Region | 46 | 39.43 ± 12.93 (20–69) | 18 (39) M / 28 (61) F | Mild vs moderate/severe AD (no further specification) | Mild: n=25 (54), moderate: n=9 (20), severe: n=12 (26)  (Moderate/severe: N=21 (46)) | Haplotype sets in the IL10 promoter region. | Fisher’s exact test | Y (40) |
|  | **Results of the analysis →** | The AGAC haplotype was significantly less frequent in patients with moderate/severe disease (haplotype frequency = 0/40) than in those with mild disease (haplotype frequency = 7/52; P = 0.032, Fisher’s exact test). | | | | | | | | | | | |
| Lammintausta (in morphology characteristic, morphology phenotype and severity category) 1993 | Cohort | 1983-1989 | Hos | Finland | European Region | 1008 | (19-41) | NR | Four subgroups according to disease severity and other atopic symptoms: 1: severe AD, history of periods of hospitalization (n=241); 2: Moderate AD, five or more ambulatory visits to the department of dermatology (n=399); 3: Mild dermatitis, one to four outpatient clinic visits (n=161); 4: Patients with allergic rhinitis, allergic conjunctivitis or asthma, but no dermatitis in childhood (n=207).  Hand dermatitis | 1: n=241 (24); 2: n=399 (40); 3: n=161 (16); 4: n=207 (21). | Occupational exposures; the occurrence, extent and distribution of papules, vesicles, erythema and lichenification | Chi-squared test | Y (626) |
|  | **Results of the analysis →** | In moderate-severe AD patients, facial dermatitis was seen in 63% and hand dermatitis in 54%, flexural dermatitis in 50% and in 52% dermatitis occurred on the body. Hand dermatitis showed an evident correlation with occupational exposure. Of the patients exposed to wet work or mechanically skin—irritating factors for 2h or more daily, 90% had hand dermatitis, compared to 50% of those who had little or no corresponding exposure. Dermatitis in other locations was not associated with occupational exposure. | | | | | | | | | | | |
| Laske 2004 | Cross-sectional | NR | Hos | Germany | European Region | 345 | 2.9 (0.2-24.3) | 193 (56) M / 152 (44) F | 4 subgroups based on SCORAD index quartiles: 0-25, 26-49, 50-74, 75-100 | SCORAD 75–100: n=41 (11.9); SCORAD 50–74: n=72 (20.8); SCORAD 26–49: n=41 (11.9); SCORAD 0–25: n=191 (55.4) | Serum IgE levels, allergic sensitization (IgE ≥ 0.35 kU/l) to food and aeroallergens, (family history of) (other) atopic diseases (e.g. bronchial asthma, allergic rhinoconjunctivitis) | Chi-squared  test, Yates test, Mann–Whitney U-test | N |
|  | **Results of the analysis →** | The mean serum IgE level in children with the high SCORAD quartile was significantly higher than in the low SCORAD quartile (5443 kU/l vs. 488 kU/l, p < 0.001). A sensitization to aeroallergens only was found in 60% of the high SCORAD group (high quartile) vs. 38% in the low SCORAD group (low quartile) (p < 0.02), to food allergens in 78% vs. 82% (n.s.) and to both aeroallergens and food allergens in 48% vs. 32% (n.s.). No significant difference for positive family history of atopic diseases or other atopic manifestations (bronchial asthma and allergic rhinoconjunctivitis). | | | | | | | | | | | |
| Lee 2018 | Cross-sectional | NR | Hos | Taiwan | Western Pacific Region | 22 | (6-18) | NR | Three groups according to disease severity: mild, moderate or severe AD: according to SCORAD (no further specification) | NR | Third-harmonic generation signals and the gray level co-occurrence matrix signatures of second-harmonic generation signals | Wilcoxon-signed rank test | N |
|  | **Results of the analysis →** | Third-harmonic generation (THG) signal intensity of lesional skin in AD were significantly positively correlated with AD severity (overall mean THG intensity and THG at different depths of the severe group was higher than that of the mild and moderate groups (P < 0.0001, for both analyses). Characteristic gray level co-occurrence matrix values were higher in severe AD, compared to mild and moderate groups. | | | | | | | | | | | |
| Liu 2020 | Cross-sectional | 2016-2017 | Hos | China | Western Pacific Region | 51 | (2-12) | NR | Moderate-to-severe AD (SCORAD > 25); further stratified into S. aureus-predominant group (AD.S) and S. aureus-non-dominant (AD.ND) group | Moderate-to-severe AD: 100%;  AD.S: n=11 (22), AD.ND: n=39 (76) | Characteristics of the cutaneous microbiota, disease severity | Wilcoxon rank-sum test | Y (31) |
|  | **Results of the analysis →** | The number of operational taxonomic units (OTUs) in the (moderate-to-severe) AD group was significantly lower than in the control group (P < 0.001). The AD group contained significantly fewer annotated genera than the control group (P < 0.001). Compared to controls, AD patients exhibited significant enrichments of Pseudomonas, Prevotella, Acinetobacter, Chryseobacterium and Desulfovibrio as well as obvious reductions of Corynebacterium, Streptococcus, Rotina, Clostridium XIVa, Gemmatimonas, Bacillus and Parabacteroides (p<0.01-0.001). The AD.S group exhibited lower skin microbial diversity (p<0.001) and higher SCORAD index (57.7 ± 6.7 vs 46.7 ± 13.1, p=0.002), than the AD.ND group. | | | | | | | | | | | |
| Lopes 2016 | Cross-sectional | NR | NR | Portugal | European Region | 73 | 30 | (39) M / (61) F | Patients with AD and severity classified based on the SCORAD score as mild (≤15), moderate (16-40), and severe (≥41) | Mild: n=7 (10), moderate: n=29 (37), severe: n=37 (51) | FLG mutations: p.Pro478Ser, p.Arg501Terc and c.2282del4 | Mann- Whitney test or Fisher exact test | N |
|  | **Results of the analysis →** | The presence of p.Pro478Ser was associated with moderate (p=0.02) and severe (p=0.01) disease, in contrast with p.Arg501Terc and c.2282del4. | | | | | | | | | | | |
| Lowe 2020 | Cohort | 1998-2016 | Pop | U.K. | European Region | 526,808 | (≥ 18) | 218,737 (41.5) M / 308,071 (58.5) F | Mild, moderate, or severe atopic eczema. By default, all people with atopic eczema were classified as having mild disease, unless they were (1) prescribed potent topical steroids or calcineurin inhibitors when they were classified as having moderate eczema, or (2) referred to a dermatologist, prescribed a systemic drug, or had a record for phototherapy when they were classified as having severe disease. | Mild: n=487,988 - 392,846, moderate: n=181,742 – 186,657, severe: n=33,190 – 34,577. | Major osteoporotic (hip, pelvis, spine, wrist, and proximal humerus) fractures | Stratified Cox regression | Y (2,569,030) |
|  | **Results of the analysis →** | Fracture risk is increased with increasing eczema severity, with the strongest associations in people with severe eczema (compared with those without atopic eczema) for spinal (HR, 2.09; 99% CI, 1.66-2.65), pelvic (HR, 1.66; 99% CI, 1.26-2.20), and hip (HR, 1.50; 99% CI, 1.30-1.74) fractures. In severe patients, an increased risk was also found for hip (HR, 1.50; 99% CI, 1.30-2.74), wrist (HR, 1.18; 99% CI, 1.00-1.39), and proximal humeral fractures HR, 1.27; 99% CI, 0.93-1.73), compared to controls. There was a 6% increase in the risk of spinal fracture in people with mild eczema (HR, 1.06; 99% CI, 0.96-1.17) and a 22% increase in those with moderate eczema (HR, 1.22; 99% CI, 1.10-1.36), compared to those without atopic eczema. The HRs with regards to other fractures for mild and moderate eczema ranged from 0.90 to 1.10. | | | | | | | | | | | |
| Martel 2016 | Cross-sectional | NR | NR | Denmark | European Region | 14 | 20-47 | 3 (21) M / 11 (79) F | Mild (extrinsic (IgE >200 kU/l) or intrinsic) AD: based on eczema area and severity index (EASI) <10 | 100% | Differentially expressed genes involved in corneocyte lipid envelope formation and inflammation. | One-way ANOVA with Tukey’s multiple comparison test, Wilcoxon signed rank test was applied, Grubbs’ test | Y (17) |
|  | **Results of the analysis →** | Expression of the majority of genes associated with skin barrier formation was unchanged or upregulated in patients with mild AD compared to normal healthy skin. No significant differences in the expression of FLG and loricrin at both mRNA and protein level were found in lesional skin from patients with mild AD. Several inflammation associated genes such as S100A9, MMP12, CXCL10 and CCL18 were highly expressed in lesional skin from patients with mild psoriasis and were also increased in patients with mild extrinsic and intrinsic AD. | | | | | | | | | | | |
| McPherson 2010 | Cohort | NR | NR | U.K. | European Region | 33 | NR | NR | Presence/absence of carriage of 2282del4 and R501X FLG null mutations in adults with moderate–severe AD: classed as > 25 SCORAD | 100% | Peripheral blood allergen-specific CD4+ T-helper 2 cell responses. | Non-parametric t-test | N |
|  | **Results of the analysis →** | FLG null mutations associated with significantly (P < 0.005) higher frequencies of allergen-specific CD4+ T-helper 2 cell responses in patients with moderate-severe AD. | | | | | | | | | | | |
| Miadonna 1985 | Cross-sectional | NR | NR | Italy | European Region | 19 | 5 ± 3.6 (6 mo- 13 y) | 10 (53) M / 9 (47) F | Children with severe atopic eczema (at least 60% of body affected) and children with lesions ranging 20-40% of body surface | children with severe atopic eczema: n=4 (21), children with lesions ranging 20-40% of body surface: n-15 (79) | Distribution of T cell subsets by means of OKT monoclonal antibodies | Unclear | Unclear |
|  | **Results of the analysis →** | An increased OKT4+/OKT8+ ratio has been detected only in three children with a particularly severe and extensive (>60% of the body surface) atopic eczema. In the other 16 children a normal ratio between lymphocyte subpopulations was found. | | | | | | | | | | | |
| Mittermann 2016 | Cross-sectional | NR | Hos | Sweden | European Region | 179 | 28 (18–65) | 78 (44) M / 101 (56) F | Adult patients with AD grouped according to the SCORAD index, into severe and moderate AD: severe AD defined as SCORAD ≥41 | Severe: n=53 (30), moderate: n=126 (70). | Specific IgE reactivity to 11 common aeroallergen sources (Phadiatop), 120 allergen components (MeDALL allergen chip), M. sympodialis, S. aureus and human epithelial cell extracts | Chi-squared test | Y (43) |
|  | **Results of the analysis →** | IgE reactivity was detected in 92% of patients with severe and 83% with moderate AD. The IgE reactivity profile in severe AD was more spread towards several different allergen molecules as compared to moderate AD. Severe AD showed a significantly higher frequency of IgE reactivity to allergens like cat (rFel d 1) and house dust mite (rDer p 4 and 10), to Staphylococcus aureus, M. sympodialis, and to human antigens. There were no significant differences in the frequencies of IgE reactivity to the grass pollen allergens rPhl p 1, 2, 5b, and 6 between the two AD groups. 34% in the severe AD group compared to 16% in the moderate AD group, p<0.01, showed reactivity to antigens in the S. aureus extract. The percentage of reactivity to the human cell extract was significantly higher (i.e., 30%) in severe than in moderate AD (i.e., 13%) (p<0.01). IgE reactivity against the M. sympodialis extract was detected more often in severe (i.e., 60%) compared to moderate AD (i.e., 39%) (p<0.01). IgE-reactivity to rMala s 7 was significantly more frequently detected in severe (38%) compared to moderate (27%) AD (p<0.01). For certain allergens (e.g. rFel d 1, rDer p 4, and rDer p 10) a significantly (p<0.05) more frequent IgE recognition was found for severe AD. The cross-reactive carbohydrate marker allergen from grass pollen (i.e., nPhl p 4) was more frequently recognized by severe AD (p<0.05) Significant difference (p<0.05) was reached for a higher IgE reactivity to rPhl p 2 in the moderate AD compared to severe AD. | | | | | | | | | | | |
| Mocsai 2014 | Cross-sectional | NR | NR | Hungary | European Region | 49 | 19 (5-36) | 22 (45) M / 27 (55) F | Three groups of patients with AD: mild-to-moderate FLG wild-type (OSCORAD ≤ 25, A), severe wild-type (OSCORAD > 25, B) and severe mutant (OSCORAD > 25, C) | A: n=10 (20), B: n=22 (45), C: n=17 (35) | Serum thymic stromal lymphopoietin (TSLP) levels, total and specific IgE levels (house dust mites, ragweed and cat dander), TEWL, atopic history ( asthma and rhinitis), FLG content and epidermal thickness. | Kruskal–Wallis test, Mann–Whitney test,  Fisher’s exact test | Y (10) |
|  | **Results of the analysis →** | Significantly increased TEWL was observed in the severe groups (groups B and C) relative to the mild-to-moderate group (group A) for both nonlesional skin (P = 0.0100 and P = 0.0262, respectively) and lesional skin (P = 0.0234 and P = 0.0464, respectively). No difference was detected between the two severe groups (groups B and C) with respect to TEWL for either lesional or nonlesional skin. When determining serum TSLP levels in the three AD groups,  no significant difference in serum TSLP levels was detected between the mild-to-moderate group and the severe groups. When combining all patients with severe AD into one group, significant elevation of serum TSLP levels was observed in patients with severe AD compared with healthy controls (P = 0.0236).  The occurrence of allergic asthma and rhinitis in the personal medical history of the patients was detected significantly more frequently in the FLG mutant group (P = 0.0166 and P = 0.0154) than in the wild-type groups (groups A and B, respectively). Prominent differences were found between the severe groups (B and C) and between the wild-type groups with respect to total serum IgE (P = 0.0181). In addition, a significant difference was found between groups A and C (P = 0.0229). These distinctions appeared even stronger when measuring specific IgE levels for ragweed and cat dander in patients with AD; indeed, significant differences were observed between the mutant and the wild-type groups (P = 0.0090 and P = 0.0472 for ragweed and P = 0.0338 and P = 0.0021 for cat dander in groups A and B and groups B and C, respectively). No significant differences were found between groups B and C with respect to the specific IgE levels for house dust mites. The two severe groups (B and C) showed significantly increased acanthosis compared with group A (P < 0.0001).  A significantly lower FLG level was observed in AD skin biopsies relative to samples from normal controls (P = 0.0001 for mildto- moderate, and P < 0.0001 for severe groups). In addition, there were significant differences between group A and groups B and C (P = 0.0010 and P = 0.0036, respectively). No differences were detected between the severe groups. | | | | | | | | | | | |
| Montero-Vilchez 2021 | Cross-sectional | 2019-2020 | Hos | Spain | European Region | 65 | 28.14±19.59 | 23 (35.4%) M / 42 (64.6%) F | Mild AD ( SCORAD < 37) versus moderate/severe AD (SCORAD ≥ 37) (mean SCORAD as cut-off) | SCORAD < 37: n=26 (40), SCORAD ≥ 37: n=34 (52) | Homeostasis parameters related to epidermal barrier function (stratum corneum hydration (SCH), TEWL, pH, erythema and melanin index, skin temperature, elasticity parameters) | Student’s t-test, receiver operating characteristic curves (ROC) curves | N |
|  | **Results of the analysis →** | SCH was significantly lower in patients with SCORAD ≥ 37 than in patients with SCORAD < 37 both at uninvolved AD skin (34.78 vs. 47.10 AU, p = 0.003) and AD eczematous lesion (19.90 vs. 30.68 AU, p = 0.044). Temperature was higher in patients with SCORAD ≥ 37 than in patients with SCORAD < 37 at AD eczematous lesion (32.45 vs. 31.74, p = 0.015). Patients with SCORAD ≥ 37 had nearly significantly higher erythema at uninvolved skin than patients with SCORAD < 37 (254.93 vs. 201.05 AU, p = 0.004). Elasticity was significantly lower in patients with SCORAD ≥ 37 than in patients with SCORAD < 37, both at uninvolved AD skin (67% vs. 79%, p = 0.003) and AD eczematous lesion (63% vs. 75%, p = 0.01). No differences were found for the other parameters. Patients with moderate/severe AD (SCORAD ≥ 37) presented a temperature > 31.75 °C a with a sensitivity of 81.8% and a specificity of 57.7% (area under the curve = 0.71, p = 0.006 ). | | | | | | | | | | | |
| Nousbeck 2020 | Cross-sectional | NR | Hos | Ireland | European Region | 28 | (3-10 mo) | 18 (64.3) M / 10 (35.7) F | Infants under 12 months of age with moderate-to-severe AD (no further details) | 100% | miRNAs in peripheral blood mononuclear cells (PBMCs) and plasma | Logistic regression models with area under the receiving operating characteristic estimation | Y (19) |
|  | **Results of the analysis →** | 10 differentially expressed miRNAs were identified in PBMCs and eight dysregulated miRNAs in plasma of infants with (moderate-to-severe) AD compared with controls. Upregulated miRNAs in PBMCs included miRNAs known to be involved in inflammation: miR-223-3p, miR-126-5p and miR-143-3p. Differential expression of only one miRNA, miR-451a, was observed in both PBMCs and plasma of children with AD. Dysregulation of three miRNAs (miR-451a, miR-143-3p and miR-223- 3p) was validated in larger numbers of samples and miR-451a was identified as a predictive biomarker for the early diagnosis of the disease. Experimentally verified targets of miR-451a, interleukin 6 receptor (IL6R) and proteasome subunit beta type-8 (PSMB8), were increased in patients with AD, negatively correlated with miR-451a levels and upregulated following inhibition of miR-451a in PBMCs. | | | | | | | | | | | |
| Oh 2009 | Cross-sectional | NR | Hos | Germany | European Region | 136 | 34 (IQR: 25–38) | 54 (40.0) M / 82 (60.0) F | Mild to moderate (SCORAD 15–50) and Severe AD (SCORAD >50) in German adults | Mild to moderate: n=99 (74), severe: n=35 (26) | Nine different SNPs in five genes:TLR1, -2, -4, -9 and TIRAP (encoding for microbial pattern recognition-related molecule by the innate immune system) | Chi-squared tests | Y (129) |
|  | **Results of the analysis →** | In severe AD, a significantly increased representation of the A-allele in position –16934 of the tlr2 gene was present (48.6 vs 20.2%; P = 0.004) in comparison to patients with mild-to-moderate disease. | | | | | | | | | | | |
| Okano-Mitani 1996 | Cross-sectional | NR | NR | Japan | Western Pacific Region | 35 | 19.9 ± 0.7 (16-35) | 11 (31) M / 24 (69) F | Severe, moderate and mild AD, based on Rajka and Langeland criteria | Severe: n=12 (34), moderate: n=15 (43), mild: n=8 (23) | LTA4 hydrolase activity in the supernatant fraction of peripheral blood polymorphonuclear leukocytes (PMN) and peripheral blood mononuclear cells (PBMC) | Student’s t-test | Y (13) |
|  | **Results of the analysis →** | The LTA4 hydrolase activities were significantly higher in preparations of cells from severe AD patients (123.94 ± 16.61 pmol/106 cells per min) than in those from moderate (49.03 ± 9.43 pmol/106 cells per min; P < 0.01) and mild (28.75 ± 11.42 pmol/106 cells per min; P < 0.01) AD patients and normal controls (15.14 ± 1.74 pmol/106 cells per min; P < 0.01). LTA4 hydrolase activities were also higher in peripheral blood mononuclear cells (PBMC) from severe AD patients (27.81 ± 8.28 pmol/106 cells per min) than in those from moderate (11.31 ± 2.11 pmol/106 cells per min; P < 0.05) and mild (6.16 ± 2.62 pmol/106 cells per min; P < 0.05) AD patients and normal controls (11.17 ± 0.83 pmol/106 cells per min; P < 0.05). LTA4 hydrolase activities in PMN were reduced after improvement of the disease in eight patients with severe or moderate AD. | | | | | | | | | | | |
| Okawa (also in morphology phenotype) 2018 | Cross-sectional | NR | Hos | Japan | Western Pacific Region | 240 | 32 ± 16.3 | 137 (57) M / 103 (43) F | Adult AD patients with mild, moderate, severe, very severe AD (according to the Japanese guidelines for AD proposed by the Research Group established by the Ministry of Health, Labor and Welfare of Japan: mild: skin involvement limited to mild eruption, moderate: <10% surface area involvement by eruption with severe inflammation, severe: 10% but <30% skin involvement by severe eruption, very severe: ≥30% of body involvement by severe eruption) and subgrouping based on morphology (erythroderma type, widespread combinations of various types, prurigo type, limb type, or head/ face/neck/chest/back type based on the individual clinical characteristics according to the modified criterion published by the JDA) | Mild: n=36 (15), moderate: n=88 (37), severe: n=72 (30),  very severe: n=44 (18)  Erythroderma type: n=42 (18), widespread type: n=137 (57),  prurigo type: n=33 (14), limb type: n=9 (3), head/face/neck/chest/back type: n=19 (8) | Serum levels of squamous cell carcinoma antigen (SCCA) 2 | Mann-Whitney U test, Kruskal-Wallis test, Dunn's multiple comparison test | Y (25) |
|  | **Results of the analysis →** | SCCA2 levels in mild, moderate, severe and very subgroups AD were significantly higher than those of healthy controls (p<0.001). Though there was no significant difference in SCCA2 levels between AD patients with mild and moderate, severe and very severe disease, higher SCCA2 levels were observed in parallel with progression of disease severity (median (IQR); mild 4.25 (1.92e5.96) ng/mL, moderate 5.35 (2.70e12.26) ng/mL, severe 11.16 (4.53e25.29) ng/mL, very severe 17.45 (5.07e36.09) ng/mL), indicating that the SCCA2 level reflects disease severity in AD patients (p<0.01 both for severe and moderate and for very severe and moderate). AD patients with erythroderma type showed significantly higher levels of SCCA2 compared to the others (p < 0.001), followed by the widespread type. On the other hand, AD patients whose lesions were not distributed systemically, such as those with limb type, had lower levels of SCCA2. In the patients with severe and very severe conditions, the ratio of the prurigo type in AD patients with lower levels of serum SCCA2 (≤2.8 ng/ml; 25% percentile) was significantly higher than the ratio in patients with higher levels of serum SCCA2 (>2.8 ng/ml), 25% (8 of 32 patients) and 7% (6 of 84 patients) respectively (p < 0.001). | | | | | | | | | | | |
| Ong 2008 | Cross-sectional | NR | Hos | U.S.A. | Region of the Americas | 50 | (1-6) | NR | Young children with mild and moderate AD: according to objective SCORAD ≤15 (mild) and >15-<40 (moderate) | Mild: n=34 (68), moderate: n=16 (32) | Allergic sensitization to staphylococcal superantigens: serum IgE to staphylococcal enterotoxin (SE) A, SEB, SEC, SED, and toxic shock syndrome toxin-1 | Logistic regressions | N |
|  | **Results of the analysis →** | The prevalence of allergic sensitization to staphylococcal superantigens in patients with mild and moderate AD was 38% and 63%, respectively. Allergic sensitization to staphylococcal superantigens, particularly SEA and SED, was found to be associated with moderate AD, compared with mild AD (P = .01 and .05, respectively). | | | | | | | | | | | |
| O'Regan 2010 | Cross-sectional | NR | Hos | Ireland | European Region | 132 | FLG+/+: 8.43 ± 3.76 | 79 (60) M / 53 (40) F | Moderate-to-severe AD according to the Nottingham Eczema Severity Score, further subgrouped by FLG genotype (FLG-/-: homozygous for null alleles, FLG+/- heterozygote null allele/wild-type, FLG+/+: homozygote wild-type) | 100% moderate-to-severe AD | NMF measured by using confocal Raman microspectroscopy and measurement of transepidermal water loss (TEWL) | ANOVA, Tukey post hoc analysis | N |
|  | **Results of the analysis →** | There was a statistically significant difference in NMF values between the 3 genotypes (P < .001, ANOVA, Tukey post hoc analysis). Raman-determined NMF discriminated between FLG-associated AD and non–FLG-associated AD (area under the curve, 0.94; 95% CI, 0.91-0.99). In addition, within the subset of FLG-associated AD, NMF distinguished between patients with 1 versus 2 mutations with an ROC AUC of 0.85 (95% CI, 0.77-0.93).  TEWL did not distinguish FLG genotype status in patients with moderate-to-severe AD (TEWL AUC, 0.583; 95% CI, 0.48-0.699; ROC curve not shown). | | | | | | | | | | | |
| Pavel (also in trajectory group) 2021 | Cross-sectional | NR | Hos | U.S.A. | Region of the Americas | 19 | 1.7 (0-5) | 14 (73.7) M / 5 (26.3) F | Early-onset (starting within the previous 6 months, age 0-5 years) moderate-to-severe (not further specified) AD | 100% | mRNA profile in lesional and nonlesional tape-stripped skin including differentially expressed genes (DEGs) and epidermal barrier alterations | Fold-changes, hypothesis testing, Spearman correlation coefficients | Y (17) |
|  | **Results of the analysis →** | 1829 DEGs were identified in lesional AD and 662 DEGs in nonlesional AD, compared to healthy skin (fold-change ≥2, FDR < −0.4, P < .05). Significant correlations were also identified between clinical measures (body surface area/BSA, pruritus ADQ, and transepidermal water loss/TEWL) with immune and barrier mRNAs in lesional and/or nonlesional AD (FLG/FLG2 with TEWL; r < −0.4, P < .05). | | | | | | | | | | | |
| Pigors 2018 | Cross-sectional | NR | Hos | U.K. | European Region | 43 | (3 mo – 22 y) | NR | Severe atopic eczema, determined by National Institute for Health and Care Excellence  guidelines | 100% | Filaggrin mutations and additional risk genes | Fisher’s exact test | Y (232) |
|  | **Results of the analysis →** | Filaggrin (FLG) harbored the highest number of enriched dominant (OR, 12.1; P < 0.0001) and recessive loss of function, that is, nonsense and frameshift mutations (OR, 43.4; P < 0.0001). FLG missense variants were not enriched in the Bangladeshi probands. In total, 13 loss-of-function FLG variants, of which 5 were unreported variants, were identified in 21 of the 43 Bangladeshi probands using whole-exome sequencing. | | | | | | | | | | | |
| Quah (also in disease trajectory category) 2015 | Cohort | 2004-2011 | Hos | Singapore | Western Pacific Region | 65 | (0-5) | NR | Early onset of eczema by 2 years + Severity of eczema up to 2 years: SCORAD quartiles: <22.56, 17.11-22.55, 11.075-17.10, <11.075 | Early onset of eczema by 2 years: n=57 (88)  SCORAD quartiles: <22.56: n=13, 17.11-22.55: n=14, 11.075-17.10: n=13, <11.075: n=13  (severity missing n=4) | Atopic disorders (manifestations of eczema, wheeze and rhinitis) and allergen sensitization at the age of 5 years | Chi-squared analysis, logistic regression analyses | Y (174) |
|  | **Results of the analysis →** | Eczema at year 2 increased the risk of eczema at year 5 (adjOR = 7.1; 95 % CI: 1.8–27.8) and this was further increased by the presence of allergen sensitization (adjOR = 25.4; 95 % CI: 4.7–138.5) and the concomitant presence of both wheeze and allergen sensitization (adjOR = 64.9; 95 % CI: 4.7–900.0). More severe eczema at the age of 2 years (SCORAD above median) was more strongly associated with eczema at year 5 than those with less severe eczema (SCORAD below median) (SCORAD above median: adjOR =15.2; 95 % CI = 4.3–53.7; SCORAD below median: adjOR = 5.7; 95 % CI = 1.4–22.7).  No significant association between eczema severity in the first 2 years of life and allergen sensitization at 2 years. Early onset of eczema at 2 years increased the risk of rhinitis (adjOR = 6.8; 95 % CI: 2.0–23.1). For HDM allergen sensitization at 5 years of age, early onset of eczema increased the risk (adjOR = 3.6; 95 % CI = 1.5–8.7). More severe eczema was significantly associated with the development of allergen sensitization at 5 years (SCORAD above median: adjOR = 2.9; 95 % CI = 1.0–8.5). Eczema severity at the age of 2 years did not affect outcomes for wheeze and rhinitis at 5 years (p > 0.05). | | | | | | | | | | | |
| Rafatpanah 2003 | Cross-sectional | NR | Hos | U.K. | European Region | 113 | Median: 4, IQR: 3-10; range: 0-16 | 64 (57) M / 49 (43) F | Children with AD were divided into 2 equal subgroups based on body surface area affected: half with the least severe AD (affecting 5%-27% of their total body surface area) and half with the most severe AD (affecting 27%-100% of their surface area). Median (IQR) percentage of body surface area affected: 27% (8%-46%) | Most severe: n=55 (49), least severe: n=58 (51) | Polymorphisms of the GM-CSF, TNF-α, and IL-1β genes | Logistic regression, chi-squared analysis | Y (114) |
|  | **Results of the analysis →** | Children with most severe AD (n = 55) had a very skewed distribution of –677 GM-CSF genotypes, with none of them inheriting the C/C genotype and 50% (n = 28) having an A/A genotype. This compared with the control group (n = 64) in which 16% (n = 10) had the C/C genotype and 16% (n = 10) had the A/A genotype (P < .001). The children with mild-to-moderate AD had genotype frequencies between these 2 extremes. No significant association was found between either IL-1β or TNF-α allele or genotype frequencies and AD. | | | | | | | | | | | |
| Sakurai 2002 | Cross-sectional | 1998-2000 | Hos | Japan | Western Pacific Region | 153 | 25 (4-46) | 78 (51) M / 75 (49) F | Patients with active AD stratified based on disease severity: mild (presence of skin lesions on less than 10% of the total body surface), moderate (involvement of 10-50%), severe (involvement of more than 50%) | Mild: n=71 (46), moderate: n=51 (33), severe: n=31 (20) | TEWL measured in normal-appearing skin, presence of patchy parakeratosis in normal-appearing skin | Student’s t -test | Y (69) |
|  | **Results of the analysis →** | TEWL with severe, moderate and mild symptoms were 10.5 ± 2.9, 8.3 ± 2.4 and 7.3 ± 2.1 g/m2 per h, respectively. Results in the severe and moderate cases were significantly (P< 0.001) different as compared with the normal controls (6.2±1.6 g/m2 per h), while TEWL in mild cases was only slightly increased (P>0.05). However, TEWL in completely healed AD for more than 5 years was 6.8±1.3 g/m2 per h, which was not significantly different than the normal controls. In the normal-appearing skin, patchy parakeratosis was found in 42% (10 of 24) of the severe cases, 29% (12 of 41) of the moderate cases, and 19% (12 of 64) of the mild cases. No evidence of patchy parakeratosis could be identified in the completely healed AD group or the normal controls. | | | | | | | | | | | |
| Salpietro 2011 | Cross-sectional | 2010-2011 | Hos | Italy | European Region | 187 | Median : 8 (0.5-12) | 96 (51.3) M / 91 (48.7) F | Mild to moderate and severe AD: according to SCORAD mild < 25, moderate 25-50, and severe > 50 | Mild to moderate: n=132 (70.6), severe: n=55 (29.4) | TLR2 (A-16934T and R753Q) and TLR4 (D299G and T399I) gene single nucleotide polymorphisms | Chi-squared test, Fisher exact test | Y (150) |
|  | **Results of the analysis →** | In two subgroups of AD children with mild to moderate and severe AD phenotypes, the occurrence of one risk allele of R753Q was associated with severe AD phenotype (P =0.002; OR 3.45; 95%CI 1.56-7.63; RR 2.14; 95%CI 1.35-3.10). The TLR2 A16934 allele was not found to be associated with the severity of AD, as well as TLR4 D299G and T399I SNPs. | | | | | | | | | | | |
| Sanchez 2017 | Cohort | 2011-2015 | Hos | Columbia | Region of the Americas | 433 | 8 (3-49) | 178 (48) M / 225 (52) F | Dermatitis/eczema ranked as severe (>40 SCORAD), moderate (16 to 39 SCORAD), or mild (<15 SCORAD) | Mild: n=102 (23%), moderate: n=214 (49%), severe: n=117 (27%) | Skin prick test results (skin sensitization), serological tests for immunoglobulins (including total IgE, specific IgE for certain allergens, IgG), maternal and paternal history of eczema | Chi-squared test, Fisher’s exact test, multivariate analyses | N |
|  | **Results of the analysis →** | Patients with severe SCORAD had higher specific IgE to Der p and Der f than the others groups (p=0.02). There were no significant differences in specific IgG and IgG4 among SCORAD groups. For the other allergen sources we did not observed that mono-sensitization were a risk factor for severe eczema.  Polysensitization to HDM, fungus, dogs, birds and cockroaches were a risk factor for severe eczema (p<0.05). Maternal and paternal eczema are important predictors of severe eczema (respectively, OR 5.45 (2.2-9.8), P<0.01 and OR 2.48 (1.9-9.8), P=0.01). | | | | | | | | | | | |
| Sandilands 2007 | Cross-sectional | NR | Hos | Ireland | European Region | 188 | 4.9 (<14) | 111 (59) M / 77 (41) F | Moderate-to-severe childhood Irish AD: according to Nottingham Eczema Severity (NESS) scale | 100% | 12 FLG mutation variants | Chi-squared test, Fisher’s exact test | Y (736) |
|  | **Results of the analysis →** | The five most common European mutations (R501X, 2282del4, R2447X, S3247X, 3702delG) showed a strong association with moderate-to-severe childhood eczema compared to controls (X2 test: P = 2.12 x 10-51; Fisher’s exact test: heterozygote odds ratio (OR) = 7.44 (95% confidence interval (c.i.) = 4.9–11.3), and homozygote OR = 151 (95% c.i. = 20–1,136)). | | | | | | | | | | | |
| Savolainen 1993 | Cross-sectional | 1983-1989 | Hos | Finland | European Region | 156 | (16-49) | 70 (45) M / 81 (52) F | Mild, moderate, severe AD in young adults: group I, mild AD: 10% or less of the body surface covered with eczema; group II, moderate AD: 10-50% of the body surface covered with eczema; group III, severe AD: more than 50% of the body surface covered with eczema | Mild: n=38 (24), moderate: n=26 (17), severe: n=11 (7) | Results of skin-prick tests, nasopharyngeal yeast cultures and immunoblotting (IgE, IgA and IgG) to allergens, including C. albicans | Kolmorogov-Smirnov goodness of fit test with continuity correction factor | Y (57) |
|  | **Results of the analysis →** | 84% of the patients in groups II and III (moderate and severe AD) developed histamine-equivalent skin test reactions whereas only 36% from groups I (mild AD) and 0,22% of AR patients and 5% of non-atopic controls appeared positive. The number of skin-prick test-positive patients is significantly greater in the combined group III/II as compared with the combined group I/O (no AD) (P < 0001), rhinitis group (P < 0.001) and non-atopic control group (P<0.001). Severe eczema was seldom seen in patients without saprophytic C. albicans growth. All C. albicans-specific IgE responses as well as the serum IgE values are mostly seen in individuals with severe forms of AD and are thus associated with the severity of the dermatitis. | | | | | | | | | | | |
| Schonmann 2020 | Cohort | 1998-2016 | Pop | England | European Region | 526,808 | Depression cohort: 43.9 ± 21.7, Anxiety cohort: 44.1 ± 21.43 | Depression cohort: 211,118 (53.8) F, Anxiety cohort: 237,527 (55.7) F | Mild, moderate, severe atopic eczema | Depression cohort: mild: 287,944, moderate: 135,485, severe: 24,777 Anxiety cohort: mild: 310,205, moderate: 147,261, severe: 27,538 | New diagnosis of depression/anxiety | Cox regression | Y (2,569,030) |
|  | **Results of the analysis →** | Stronger effect of atopic eczema on depression with increasing atopic eczema severity (HR [99% CI] compared with no atopic eczema: mild, 1.10 [1.08-1.13]; moderate, 1.19 [1.15- 1.23]; and severe, 1.26 [1.17-1.37]). A dose-response association was less apparent for new anxiety diagnosis (HR [99% CI] compared with no atopic eczema: mild, 1.14 [1.11-1.18]; moderate, 1.21 [1.17-1.26]; and severe, 1.15; [1.05-1.25]). | | | | | | | | | | | |
| SchultzLarsen 1985 | Cross-sectional | NR | Pop | Denmark | European Region | 47 | (6-19) | 22 (47) M / 25 (53) F | Mild AD, moderate AD (no further specifications) | Mild: n=10 (21), moderate: n=6 (13) (history of AD: n=31 (66)) | Lymphocyte transformations to phytohaemagglutinin, concanavalin A (Con A), pokeweed mitogen, and tuberculin. Lymphocyte subpopulations characterized by E_AET_ rosettes and monocloncal antibodies OKT3, OKT4, and OKT8. | Unpaired t test, Wilcoxon’s signed rank test, Mann-Whitney U test | Y (47) |
|  | **Results of the analysis →** | In moderate AD: statistically insignificant tendency (0.10-0.20>P>0.05) to a decreased lymphocyte transformation at suboptimal concentrations of concanavalin A (Con A) (12.5ug/ml) and a reduction of the OKT 8+ cells with an increased OKT4/OKT8 ratio. | | | | | | | | | | | |
| Semic-Jusufagic 2007 | Cohort | 1995-2002 | Pop | U.K. | European Region | 68 | (0-5) | NR | Childhood AD (5 years of age) with subgrouping based on eczema severity: mild, moderate/severe (no further specification) | Mild: n=61 (90), moderate/severe: n=7 (10) | Staphylococcus aureus–secreted enterotoxins (SEs): SE-mix IgE antibodies | T test, chi-squared test, logistic regression | Y (432) |
|  | **Results of the analysis →** | SE-mix sensitization rate increased significantly with increasing eczema severity (no eczema, mild, moderate/severe: 8.3%, 14.8%, 42.9%; P = .003) and remained independently associated with eczema in a multivariate model adjusting for total IgE (adjusted odds ratio, 2.19; 95% CI, 1.05-4.56; P = .04 | | | | | | | | | | | |
| Seneviratne 2006 | Cross-sectional | NR | Hos | U.K. | European Region | 22 | NR | NR | Severe vs mild AD: severe: Six Area, Six Sign Atopic Dermatitis (SASSAD) score of >54 (of a possible total of 108), mild: SASSAD scores of <10. | Mild AD: n=10 (45), severe AD: n=12 (55) | Production by CD4+ T cells of TNF-a, IL-4, IL-5, IL-13 and IL-10 in response to phorbol myristate acetate ⁄ ionomycin and Der p1 allergen | Student’s t-test | Y (10) |
|  | **Results of the analysis →** | There were significantly higher frequencies of allergen-specific circulating CD4+ T cells producing TNF-a - IL-4-, IL-5- and IL-13, and lower frequencies of these cells producing IL-10 in individuals with severe AD compared with mild AD and nonatopic controls (P < 0.01 for all comparisons). | | | | | | | | | | | |
| Shen 2018 | Cross-sectional | 2015-2016 | Hos | China | Western Pacific Region | 158 | 44.5 ± 34.0 months | 94 (59) M / 64 (41) F | Pediatric patients with mild (<25), moderate (25-50) and severe (>50) AD, based on SCORAD | Mild: n=49 (31.0), moderate: n=61 (38.6), severe: n=48 (30.4) | Skin ceramide subclass profiles: CER[NS] and CER[AS] (N: nonhydroxy fatty acids, A: α-hydroxy fatty acids, S: sphingosines) | Wilcoxon signed ranks tests | Y (55) |
|  | **Results of the analysis →** | No significant difference of the 2 CER subclasses (CER[NS] and CER[AS]) between mild, moderate, and severe patients. A trend that children with moderate to severe diseases have elevated levels of CER[NS] and CER [AS] compared with children with mild conditions. | | | | | | | | | | | |
| Silva 2010 | Cross-sectional | 2007 | Pop | Brazil | Region of the Americas | 296 | (2-10) | 150 (51) M / 146 (49) F | Mild and severe AD in 2 to 10-aged children from an area of low socioeconomic index in Brazil, based on ISAAC questionnaire: Mild AD was defined as eczema that appears and disappears in the last 12 months. Severe AD as eczema, flexural lesions in the last 12 months and night itching. | Mild: n=120 (40.8), severe: n=176 (58.2) | Presence of Ascaris lumbrocoides in stool | Univariate and multivariate logistic regression and descriptive analysis | Y (899) |
|  | **Results of the analysis →** | In the mild AD group of children, 11 (36.7%) were infected by Ascaris lumbricoides, while in the severe AD group, 40 (22.9%) had the same geohelinthosis (p=.01). A.Lumbricoides infection increased the frequency of mild AD (RR=1.7;P=.009), but not severe AD (RR=.86;P=.46), compared to controls. A.lumbrocoides decreased the frequency of severe AD (RR=1.46;P.016), compared to mild AD. In multivariate logistic regression, presence of the parasite had a strong association with the mild form of AD (OR=2.26; 95% CI 1.38-3.68; P<.001). | | | | | | | | | | | |
| Silverwood 2018 | Cohort | 1998-2015 | Pop | U.K. | European Region | 387 439 | Median: 43 (≥18) | 139 908 (36.1) M / 247 531 (63.9) F | Mild, moderate and severe atopic eczema in adults: patients with atopic eczema were considered to have mild conditions by default. They were classified as having moderate atopic eczema at the first of: a second potent topical corticosteroid treatment within one year or a first calcineurin inhibitor treatment. Patients were classified as having severe atopic eczema at the  first of: a systemic immunosuppressant treatment; a phototherapy code in CPRD or Hospital Episode Statistics; or a referral for atopic eczema. | Severe: n=19 700 (5.1%) | Cardiovascular outcomes: myocardial infarction, unstable angina, heart failure, atrial fibrillation, stroke, and cardiovascular death. | Cox regression model | Y (1 528 477) |
|  | **Results of the analysis →** | Patients with severe atopic eczema had a 20% increase in the risk of stroke (hazard ratio 1.22, 99% confidence interval 1.01 to 1.48 in the adjusted model), 40% to 50% increase in the risk of myocardial infarction (HR 1.41, 99% CI 1.15 to 1.71), unstable angina (HR 1.48, 99% CI 1.08 to 2.03), atrial fibrillation (HR 1.38, 99% CI 1.17 to 1.62), and cardiovascular death (HR 1.38, 99% CI 1.17 to 1.62), and 70% increase in the risk of heart failure (HR 1.69, 99% CI 1.38 to 2.06). | | | | | | | | | | | |
| Simpson 2018 | Cross-sectional | 2013-2014 | Pop, Hos | U.S.A. | Region of the Americas | 1519 | 45.7 ± 17.4 | 552 (34.3) M / 997 (65.7) F | Mild or moderate/severe AD based on the PO-SCORAD: mild disease < 25 and moderate/severe disease ≥ 25 | Mild: n=689 (45), moderate/severe: n=830 (55) | validated measures and stand-alone questions assessing itch (pruritus numerical rating scale; PO-SCORAD itch visual analog scale), pain (numerical rating scale), sleep (PO-SCORAD sleep visual analog scale; sleep interference with function), anxiety and depression (Hospital Anxiety and Depression Scale), and health-related quality of life (DLQI). | Chi-squared test, student t test | N |
|  | **Results of the analysis →** | Moderate/severe patients reported greater severity and duration of itch* and more pain, more sleep problems**, higher prevalence of anxiety and depression (50.2% vs 27.3%), and greater health-related quality-of-life impairment (DLQI 9.2 vs 2.9;), relative to mild AD (all p<.001). *Results for itch included more days per week with itchy skin (5.7 vs 2.7) and higher proportions with itch duration greater than half a day (22.8% vs 2.9%). **Results for sleep included trouble sleeping (3.9 vs 1.1 on the PO-SCORAD VAS), longer sleep latency (38.8 vs 21.6 minutes), more frequent sleep disturbances (2.6 vs 0.4 nights in past week), and greater need for over-the-counter sleep medications (39% vs 21%)(all p<.001). These sleep problems “much” or “very much” interfered with daily function in a higher proportion of patients with moderate/severe AD (24.3% vs 6.8%; P <.001). | | | | | | | | | | | |
| Sugawara 2012 | Cross-sectional | 2008-2009 | Hos | Japan | Western Pacific Region | 24 | 25 ± 4 (20-41) | 24 (100) M / 0 (0) F | Mild AD patients: based on low local SCORAD scores on the volar forearm | 100% | Levels of various NMF components in the SC | T-test, Dunnett’s test, repeated measures ANOVA | Y (18) |
|  | **Results of the analysis →** | The total NMF content of each tape stripped SC sample was decreased in AD patients (.2-.4 nmol/nmol Hydro SC p<.01). Free amino acids (FAAs) levels were not remarkably reduced, levels of pyrrolidone carboxylic acid (PCA), lactate, urea, sodium and potassium were significantly decreased in NMF from mild AD skin compared to controls. Unlike the distribution of FLG-derived FAAs and PCA, lactate, urea, potassium and sodium were abundant in the surface layer of the SC compared with the inner layer of the SC. Such findings strongly suggest that those components are supplied from outside the SC, i.e. they originate from sweat | | | | | | | | | | | |
| Takigawa 1991 | Cross-sectional | NR | NR | Japan | Western Pacific Region | 84 | (12-48) | 44 (52) M / 40 (48) F | Mild, moderate, severe AD: based on the extent of the dermatitis patients were classified as severe (> 50% skin surface involved), moderate (50-10%) and mild (< 10%) | Mild: n=25 (30), moderate: n=14 (17), severe: n=45 (54) | FcεRII+ peripheral blood mononuclear cells (PBMC) | Student's t-test | Y (55) |
|  | **Results of the analysis →** | Patients with severe and moderate AD had 5.9% and 5.7% FcεRII+ peripheral blood mononuclear cells (PBMC), respectively, that were significantly higher than percentages in mild AD patients (2.6%), severe to moderate eczematous dermatitis (2.3%), mild eczematous dermatitis (2.2%) and normal individuals (1.7%) (0.05 > P). The intensity of FceRII expression on PBMC was significantly higher in the severe AD group, compared with the five other groups, where no difference in the intensity was noted. In severe and moderate AD, 10% of FcεRII+ PBMC were T cells that preferentially expressed CD8, and the remainder B cells and monocytes. FcεRII+ T cells comprised 1% of peripheral T cells, while half or more of peripheral B cells expressed FcεRII+. In mild AD, eczematous dermatitis patients and normal subjects, FcεRII were expressed exclusively on 25-35% of peripheral B cells. | | | | | | | | | | | |
| Thijs 2017 | Cross-sectional | NR | Hos | The Netherlands | European Region | 193 | Moderate: 30.6, severe: 31.1 | 81 (42) M / 112 (58) F | Adult patients with moderate-to-severe AD: based on six area, six sign atopic dermatitis [SASSAD] score: moderate AS with SASSAD scores ranging from 15 to 20, 21 to 25, and 26 to 30 and patients with severe AD with SASSAD scores ranging from 31 to 35, 36 to 40, and greater than 40. See results* | 100% moderate-to-severe (moderate: n=95, severe: n=98) | 147 serum mediators, total IgE levels, and 130 allergen-specific IgE levels. SASSAD score, body surface area (BSA) involvement, serum TARC levels | Data-driven approach for further specification of phenotype, by using principal component analysis and  cluster analysis | Y (30) |
|  | **Results of the analysis →** | *4 distinct clusters of patients with AD were identified. Cluster 1 had high SASSAD scores and body surface areas with the highest levels of pulmonary and activation-regulated chemokine, tissue inhibitor of metalloproteinases 1, and soluble CD14 (23% of population, n=44). Cluster 2 had low SASSAD scores with the lowest levels of IFN-a, tissue inhibitor of metalloproteinases 1, and vascular endothelial growth factor (29%, n=54). Cluster 3 had high SASSAD scores with the lowest levels of IFN-b, IL-1, and epithelial cytokines (33%, n=62). Cluster 4 had low SASSAD scores but the highest levels of the inflammatory markers IL-1, IL-4, IL-13, and thymic stromal lymphopoietin (15%, n=28). Moderate AD had a lower SASSAD score and lower median body surface area (BSA) involvement than patients with severe AD (geometric mean SASSAD score, 22.3 [95% CI, 21.3-23.3] vs 39.1 [95% CI, 37.5-40.9], respectively; median BSA, 33% [Q1-Q3, 21% to 48%] vs 54% [Q1-Q3, 39% to 72%], respectively). All patients with moderate-to-severe AD had increased serum levels of thymus and activation-regulated chemokine (TARC) compared with control subjects (median, 3950 pg/mL [Q1-Q3, 2104-8944 pg/mL] in patients with severe AD,1505 pg/mL[Q1-Q3, 654-3200 pg/mL]in patients with moderate AD, and 97 pg/mL [Q1-Q3, 68-137 pg/mL] in control subjects). | | | | | | | | | | | |
| Thompson 1983 | Cross-sectional | NR | NR | U.S.A. | Region of the Americas | 7 | (17-40) | 5 (71) M / 2 (29) F | Patients with moderate to severe AD and substantially elevated serum IgE levels of 1339 to 24,261 lU/ml | 100% | Peripheral blood T lymphocytes analyzed for Fc receptors for IgE (Tε cells) and IgG (Tɣ cells) by rosette assays and characterized with monoclonal antibodies | Student t-test | Y (14) |
|  | **Results of the analysis →** | Six of seven patients with moderate to severe AD and IgE levels of 1339 to 24,261 IU/ml had less than 0.1% Tε cells and significantly fewer Tɣ cells (3.1% ± 2.7, p < 0.01) than the nonatopic control donors and the atopic donors in remission. The donors with moderate to severe atopic disease had higher percentages of IgE resetting PBL (3.6% ± 1.9) and lower percentages of IgG-rosetting PBL (8.2% ± 3.9, p < 0.01). Nonatopic control donors and atopic donors in remission had similar percentages of IgG rosetting T cells (10 .5% ± 4.1 and 7.2% ± 3.7, respectively), compared with the significantly reduced percentage of IgG rosetting T cells in the moderately to severely atopic donors (3.1%± 2.7, p< 0.01). | | | | | | | | | | | |
| Toncic 2020 | Cross-sectional | 2016-2017 | Hos | Croatia | European Region | 100 | 25 (17-43) | 31 (31) M / 69 (69) F | Moderate-to-severe AD, based on SCORAD > 15 | Mild: n=10 (10), moderate-to-severe: n=90 (90) | Presence of three FLG mutations (R501X, 2282del4 and R2447X); stratum corneum levels of filaggrin degradation pruducts (NMF), TEWL and pH in lesional and non-lesional skin | Mann–Whitney U test, Wilcoxon matched-pairs signed-rank test | Y (50) |
|  | **Results of the analysis →** | 3% of AD patients had FLG mutation 2282del4, 1% had R501X. We did not find R2447X in our population. R501X, 2282del4 and R2447X were not found in healthy individuals. Highest NMF levels were found in healthy skin, gradually decreasing from healthy to non-lesional and lesional skin, respectively. The differences between healthy and lesional AD skin and between lesional and non-lesional skin were statistically significant (P < 0.0001). TEWL was significantly higher in AD skin compared to healthy skin, and the TEWL increase was more prominent in lesional than in non-lesional skin. A similar pattern was observed for pH. | | | | | | | | | | | |
| Torsney 1966 | Case-control | 1951-1963 | Hos | U.S.A. | Region of the Americas | 72 | (3 mo-10 y) | 38 (53) M / 34 (47) F | Children hospitalized for severe AD | 100% | Congenital anomalies, family history of allergy, allergic history of hayfever, asthma, or hives, death | No formal statistical test | Y (144) |
|  | **Results of the analysis →** | There was a positive family history of allergy in 64 % of the AD patients and 43 % of the controls. There was past allergic history of hayfever, asthma, or hives at the time of the initial admission in 18 % of the AD patients and 5 % of the controls. The frequency of associated allergic manifestations increased with follow-up to 43 % in the AD group and 8.3 % in the controls. It was also found that 48 % of the index cases still had AD at the time of follow-up. There was an 18 % incidence of significant congenital anomalies in our AD group as compared to 9.7 % in our controls. Of perhaps greater significance: 23.6 % of our patients with active disease required additional hospitalization or prolonged medical care for other medical conditions as compared to 8 % of the controls. It was found that 3 of our dermatitis patients and 2 of the controls had died. | | | | | | | | | | | |
| Uehara 1989 | Cross-sectional | 1985-1987 | Hos | Japan | Western Pacific Region | 200 | (15-30) | NR | Mild vs severe AD: Mild: localization of active skin lesions to two or three anatomical areas for at least 6 months prior to examination. Severe: involvement of 70% or more of the total body surface for at least 6 months before the study. | Mild: n=100 (50), severe: n=100 (50) | Personal and family atopic respiratory disease (ARD) history: 3 groups: 1 those who had personal ARD history (n=98), 2 those who did not have personal ARD history, but had a family history of ARD (n=40), 3 AD patients who had neither personal nor a family history of ARD (n=62). Serum IgE levels. | No formal statistical test | N |
|  | **Results of the analysis →** | A very high serum IgE value was observed in 25% of mild cases, while a normal or moderately elevated serum IgE level was seen in 39% of the severe cases.  The distribution of the three groups of ARD history in mild AD cases was almost the same to that in severe AD cases: Group 1: mild n=49, severe n=50; Group 2: mild n=21, severe n=18; Group 3: mild n=30, severe n=32. In the group of patients with a personal history of ARD, serum IgE levels were elevated in the majority of mild cases, and in all of the severe cases. Very high serum IgE values were obtained in nearly all (47/49) of the severe cases, and in a considerable number (17/38) of the mild cases. Patients with severe AD and normal serum IgE mostly lacked personal and family ARD history. A striking difference in magnitude of serum IgE elevation was found between patients with severe AD with a personal history of ARD and without any personal or family history of ARD. Serum IgE levels were normal in 20 (63%) of the 32 severe cases in group 3. Most patients with severe AD and a normal serum IgE value belonged to the group 3. | | | | | | | | | | | |
| Ungar 2020 | Cross-sectional | NR | NR | NR | NR | 27 | 39.2 ± 14.3 (>18) | 19 (70.1) M / 8 (29.9) F | Moderate-to-severe AD, EASI ≤ 50  Very severe AD, EASI > 50 | Moderate-to-severe AD: n=20 (74)/100%, very severe AD: n=7 (26) | Aortic and carotid vascular inflammation | Student t-tests, chi-squared tests, fold change, Spearman correlation coefficient | Y (12) |
|  | **Results of the analysis →** | Patients with (moderate-to-severe) AD had elevated aortic max target-to-background ratio (TBR) (fold change [FCH] = 1.45, P = .057) versus healthy controls and significantly elevated mean TBR (FCH = 1.20; P < .05) in the right carotid (RC) arteries versus controls. When examining greatest focal inflammation (most diseased segment [MDS] TBR), patients with AD had higher aortic inflammation (FCH = 1.28; P = .052). AD clinical severity significantly correlated with C-reactive protein (ρ = 0.60, P < .01) and with RC mean TBR levels (ρ = 0.60, P = .04). Stratifying patients into moderate-to-severe and very severe AD showed greater RC mean TBR in patients with very severe AD versus controls (FCH = 1.31; P = .02) and versus patients with moderate/severe AD (FCH = 1.23, P = .05). Aortic inflammation was also significantly greater in patients with very severe AD versus controls (max TBR: FCH = 1.6, P = .04; MDS TBR: FCH = 1.73, P = .03). AD and control patients were also matched on total cholesterol in a subset analysis. There was a trend towards elevated TBR in AD vs controls. Total cholesterol levels in patients with AD did not correlate with TBR. | | | | | | | | | | | |
| Uysal  2018 | Case-control | NR | Hos | Turkey | European Region | 60 | Median: 18 mo, IQR:15.5–28 mo; range: 12-36 mo | 40 (66.7) M / 20 (33.3) F | Mild, moderate, severe AD in children: based on SCORAD and EASI (no further specification) | Mild: n=6 (10), moderate: n=28 (47), severe: n=26 (43) | Novel oxidative stress parameters: native thiol, total thiol and disulphide levels, and ratios as dynamic thiol/disulphide homeostasis (dTDH) | Student t-test, ANOVA, Mann-Whitney U-test | Y (60) |
|  | **Results of the analysis →** | There was no significant difference between mild, moderate and severe AD in terms of native thiol, total thiol or disulphide levels, or in terms of disulphide/ native thiol, disulphide/total thiol or native/total thiol ratios (p-values: 0.06-0.66). | | | | | | | | | | | |
| Valenzuela 2020 | Cross-sectional | 2019 | Hos | Chile | Region of the Americas | 23 | 30.52 ± 12.03 | (56.5) M / (43.5) F | Moderate/severe AD, based on SCORAD and EASI, Body Surface Area (BSA) index, and the Investigator Global Assessment scale for Atopic Dermatitis (IGA) | 100% | Gingival crevicular fluid (GCF) protease profile | Mann-Whitney U test, Fisher’s exact test, t-test, multiple linear regression model | Y (21) |
|  | **Results of the analysis →** | The GCF levels of zinc-binding ADAM8, ADAM9, MMP8, Neprilysin/CD10, aspartyl-binding Cathepsin E, serin-binding Protein convertase9, and uPA/Urokinase proteases were lower in moderate/severe AD patients compared to controls (p < 0.05). No inter-group differences in the levels of the other 28 proteases were found. MMP8, Cathepsin E, and ADAM9 were the biomarkers with the highest sensitivity and specificity regarding the detection of AD (p < 0.05). Lower levels of MMP8 in the GCF from the AD group versus healthy group (p = 0.029) were found. This difference remained significant after adjustment by periodontitis (p = 0.042). MMP8 revealed the diagnostic potential to identify AD patients versus healthy controls, (ROC area = 0.672, p < 0.05). | | | | | | | | | | | |
| Verzeaux 2018 | Cross-sectional | NR | NR | France | European Region | 10 | (18-50) | 0 (0) M / 10 (100) F | Mild-to-moderate atopic patients: based on mean SCORAD 37.5 | 100% | In vivo confocal Raman microspectroscopy data on the SC | Student’s t test, orthogonal PLS analysis | Y (10) |
|  | **Results of the analysis →** | Atopic skin revealed a significant modification of lipid organization and protein conformation in addition to the decrease of the lipid-to-protein ratio (<0.05). This study also highlighted a significant reduction of the bound water and an increase in protein organized secondary structure in atopic skin (p<0.05). | | | | | | | | | | | |
| Wang 2020 | Cross-sectional | NR | Hos | China | Western Pacific Region | 28 | (0-2) | NR | Moderate-to-severe AD, according to criteria of the Japanese dermatological association | 100% | Lipid markers in facial surface skin lipids | Orthogonal partial least  squares analysis | Y (32) |
|  | **Results of the analysis →** | 29 lipid components had differences in AD compared to healthy skin: Sphingolipids: Cer(d18:2/20:0), Cer(d16:1/23:0), Cer(d18:1/18:1(9Z)), Cer(d18:1/19:0), SM(d18:0/20:0); glyceroglycolipids: TG(13:0/14:0/20:2(11Z,14Z))[iso6], TG(14:0/14:0/15:1(9Z))[iso3], TG(13:0/15:1(9Z)/17:1(9Z))[iso6], TG(22:3(10Z,13Z,16Z)/22:6(4Z,7Z,10Z,13Z,16Z,19Z)/22:6(4Z,7Z,10Z,13Z,16Z,19Z))[iso3], TG(14:0/17:0/22:2(13Z,16Z))[iso6], TG(14:0/14:0/16:0)[iso3], TG(13:0/14:1(9Z)/16:1(9Z))[iso6], TG(13:0/14:1(9Z)/20:2(11Z,14Z))[iso6], TG(15:0/18:0/18:1(9Z))[iso6], DG(18:4(6Z,9Z,12Z,15Z)/22:1(11Z)/0:0)[iso2], DG(15:1(9Z)/17:0/0:0)[iso2], DGTS(16:0/18:2(9Z,12Z)); glycerophospholipids: PA(22:2(13Z,16Z)/17:0), PA(P‐16:0/21:0), PE(18:3(6Z,9Z,12Z)/20:0), PE(20:2(11Z,14Z)/19:0), 1‐(8‐[5]‐ladderane‐octanyl)‐2‐(8‐[3]‐ladderane‐octanyl)‐sn‐glycerophosphoethanolamine; fatty acyls: 21‐Methyl‐8Z‐pentatriacontene, 13‐Methyl‐1‐tritriacontene, 1‐Octacosene, Mayolene‐16, N′‐5Z,8Z,11Z,14Z‐eicosatetraenoyl‐N″diethyl‐ethylenediamine, Palmityllaurate, Type IV cyanolipideicosanoyl ester. | | | | | | | | | | | |
| Wehrmann 1990 | Cross-sectional | NR | NR | Germany | European Region | 41 | 28 ± 12 (16-40) | 18 (44) M / 23 (56) F | Severe AD, defined as an acute exacerbation stage of the disease with typical clinical features and a body involvement of at least 50% of the surface | 100% | Immunophenotypic characteristics of natural killer cell subsets | Student’s t tests | Y (40) |
|  | **Results of the analysis →** | Lower percentages of cells with expression of CD16, CD56 and CD57 surface antigens in severe AD compared to (healthy) controls (p<0.05). No essential difference in the percentage of CD56+ cells with CD3 antigen-coexpression was found between all groups (controls 2.3 ± 0.4%; allergic rhinitis 2.3± 0.4%, severe AD 2.0±0.4%). A reduction in cells with immunophenotype CD56+ CD3- seen in severe AD (4.8%, p<0.05). Lower percentages of CD56+ NK cells with CD16 antigen-coexpression in both groups severe AD and allergic rhinitis (2.3±0.4%, p<0.01; 3.0±0.6%, p<0.05). Lower percentage of CD8+ peripheral blood lymphocytes in patients with severe AD (12.4±1.0%) compared to allergic rhinitis and controls. The number of CD4+ peripheral blood lymphocytes was higher in severe AD (52.3±1.4%, p<0.001) than in the allergic rhinitis (39.8±2.1%, N.S.) and control group 43.7±014%). | | | | | | | | | | | |
| Wehrmann 1989 | Cross-sectional | NR | Hos | Germany | European Region | 15 | Median: 23 (15-35) | NR | Severe AD: based on criteria of Hanifin & Rajka (no further specification) | 100% | In vitro generation of IFN-gamma, in vivo IgG4 and IgE concentration and the number of FcRL/CD23 (low affinity Fc receptor for IgE) positive lymphocytes. | Student’s t-test | Y (10) |
|  | **Results of the analysis →** | Significantly impaired capacity to secrete IFN-gamma after phythaemagglutinin-stimulation compared to controls (mean 193 (SD 46) U/ml) in severe AD patients (mean 90 (SD 26) U/ml) (P<0.05). Higher IgG4 levels in patients IgG4 (mean 1.38 (SD 0.40) mg/ml) compared to controls (mean 0.59 (SD 0.11) mg/ml), n.s. Levels of IgG1, IgG2, and IgG3 did not differ significantly. A significant portion of lymphocytes bearing the low affinity Fc receptor for IgE (CD23) was observed in patients (7/8 patients) with a range 2-10% positive. Those of controls were completely negative or <2%. | | | | | | | | | | | |
| Wei 2019 | Cross-sectional | NR | Pop | U.S.A. | Region of the Americas | 801 | 45.2 | 226 (28) M / 575 (72) F | Moderate-to-severe AD, moderate AD (Rajka-Langeland score: 4.5-7.5), severe AD (Rajka-Langeland score ≥8) | Moderate-to-severe: n=801 (100),  moderate: n=590 (73.7)  severe: n=211 (26.3) | Patient-reported flares, AD symptoms (POEM, pruritus NRS, sleep disruption), Quality of life (DLQI), Treatment Satisfaction (TSQM score), workplace productivity | Chi-squared test, t-test, Spearman’s rank-order correlation | N |
|  | **Results of the analysis →** | In the 12 months before the baseline survey, 74.2% of patients reported no or less than 3 months of remission. The most common medications patients reported using within the past month were topical corticosteroids (63.6%), whereas 11.4% and 5.1% of patients used systemic corticosteroids and systemic immunosuppressants, respectively. Overall, 81.3% of patients had experienced at least 1 flare over the past month. The mean (SD) POEM score (measure of symptom frequency) over the past week was 10.3 (7.6); 57.6% of patients reported moderate-to-very-severe symptoms over the past week (POEM scores >8). The mean (SD) pruritus NRS score for worst itch during the previous 24 hours was 5.1 (3.2); 34.3% of patients experienced 1 to 4 days of sleep disruption over the past week. The mean DLQI score among all participants was 6.4; 43.9% of patients reported that AD had a moderate to extremely large effect on QoL (DLQI >=6). Among working patients (78.8%) that reported missing work, the mean (SD) number of hours missed because of AD was 7.1 (8.2) hours in the past 7 days.  Patients with severe AD had a higher mean POEM score (16.0 vs 8.2) and pruritus NRS score for itch at the worst moment (6.9 vs 4.5) than patients with moderate AD (P < .001 for all). For 3 to 7 days of the week, 54.0% of patients with severe AD had sleep disruption vs 12.2% of patients with moderate AD . For 5 to 7 days of the week, 77.3% of patients with severe AD had itchy skin vs 36.7% of patients with moderate AD (P < .001). Patients with severe AD had higher mean DLQI scores than patients with moderate AD (10.6 vs 5.0; P < .001). Patients with severe AD also reported significantly worse outcomes on all DLQI items compared with those with moderate AD (all P < .001). Patients with severe AD also experienced significantly lower (P < .001) TSQM scores across all domains than patients with moderate AD. Patients with severe AD experienced significantly more flares than patients with moderate AD (2.4 vs 1.6, P < .001). Patients with severe AD also had significantly greater impairment while working, as well as in usual activities and overall work impairment (all P < .001), than those with moderate AD. Except for percentage of work time missed because of AD and absenteeism, and number of hours missed from work, outcomes were significantly worse across all other WPAI domains/questions/categories (P < .001) among patients with severe vs moderate AD. | | | | | | | | | | | |
| Winge 2011 | Cross-sectional | NR | Hos | Ethiopia | African Region | 103 | 9.4 (0.3-34.0), median: 7.0 | 58 (56) M / 45 (44) F | Ethiopian patients with mild, moderate vs severe AD: cases were categorized into mild (< 15 points), moderate (15–40 points) or severe (> 40 points) AD according to objective SCORAD | Mild: n= 2 (2), moderate: n=67 (65), severe: n=34 (33) | Total serum IgE and allergen-specific serum IgE levels | Mann–Whitney U-test | N |
|  | **Results of the analysis →** | Patients with severe AD had significantly higher total IgE (400 ± 1349.7 vs 77.5 ± 809.9 in mild to moderate AD ) (P = 0.012) and more had elevated allergen-specific serum IgE levels against Der p (31%) than patients with mild to moderate AD (20%), although this did not reach statistical significance (P = 0.32). | | | | | | | | | | | |
| Yoshikawa 2000 | Cross-sectional | 1995-1996 | Hos | Japan | Western Pacific Region | 82 | (≥3) | 35 (43) M / 47 (57) F | Mild, moderate, severe AD based on the Kitasato University classification:  Mild: Mainly dry skin with localized lesions such as papules and erythema. Moderate: Papules, erythema, etc. are found diffuse, but lichenification and wet lesions are only localized. Severe: Frequent lesions such as papules and erythema are diffuse, and lichenification and oozing lesions are prominent. | Mild: n=15 (18), Moderate: n=53 (65), Severe: n=14 (17). | IgE level: low (≤375 IU / ml), moderate (between 375 and 2000 IU / ml), high (> 2000 IU / ml) | Kruskal Wallis test, post-hoc Tukey’s test | N |
|  | **Results of the analysis →** | Subgrouping based on IgE level was associated significantly with subgrouping based on AD severity (p=0.0457). As the severity increased, IgE also tended to increase. Severe AD had a higher IgE level (12460.1 ± 16775.4) than mild (1285.5 ± 1137.0, p<0.01) and moderate AD (4481.9 ± 7904.6, p<0.05). | | | | | | | | | | | |
| Zhang 2011 | Cross-sectional | NR | Hos | Japan | Western Pacific Region | 9 | 23-41 | 5 (56) M / 4 (44) F | Mild, moderate, or severe AD (no further specification) | Mild: n=3 (33), moderate: n=3 (33), severe: n=3 (33) | Skin fungal microbiota (species diversity of Malassezia microbiota, non-Malassezia yeast microbiota, filamentous fungal microbiota) in face scale samples | Principal coordinates analysis | Y (10) |
|  | **Results of the analysis →** | No remarkable correlations between the number of clones or detected species of Malassezzia and disease severity were found. The groups (mild, moderate, and severe) accounted for 67.8 ± 2.2, 70.7 ± 2.8, and 64.9 ± 1.8% of the clones, respectively, and 4.3±0.6, 4.0±1.0, and 5.3±0.6 species per case, respectively. M. globosa and M. restricta were the predominant species, regardless of disease severity; however, the ratio of M. globosa to M. restricta was different in each severity group. In patients with mild and moderate disease, the ratio of these species was nearly identical (M. restricta/M. globosa: 3.1–3.4 for mild and 2.1–4.1 for moderate vs. 1.1–1.4 for severe). The number of Non-Malassezia yeast microbiota species decreased with increasing severity of AD (i.e. 16.3 ± 1.2, 13.0 ± 0, and 9.7 ± 1.5 for mild, moderate, and severe, respectively. Regarding the sequence profiles for the predominant skin fungi (clustered by principal coordinates analysis): Patients with mild or moderate symptoms constituted a single cluster, whereas patients with severe disease and healthy individuals were in a separate cluster. | | | | | | | | | | | |
| Zheng 2019 | Cross-sectional | NR | Hos | China | Western Pacific Region | 48 | (0-1) | 26 (54) M / 22 (46) F | Mild AD: EASI <4, moderate AD: EASI 4-8, severe AD: EASI >8 | Mild: n=13 (27),  moderate: n=15 (31),  severe: n=20 (42) | Perioral skin bacterial diversity | Wilcoxon rank-sum test, Kruskal-Wallis H test | Y (20) |
|  | **Results of the analysis →** | The abundance of Bacteroidetes and Fusobacterium decreased significantly with an increase in disease severity (p< 0.01). The abundance of 6 genera, including Prevotella, decreased significantly with an increase in disease severity (p< 0.05). The abundance of Prevotella melaninogenica decreased gradually with an increase in disease severity. | | | | | | | | | | | |

Articles in alphabetical order. Column methodological approach: presents the methodological approach for investigating associations, unless further specified (i.e. in case of data-driven approach to identify phenotypes). AD, atopic dermatitis; Hos, hospital-based; Pop, population-based; No., number of participants with (atopic) dermatitis; NR, not reported; SD, standard deviation; Y, yes; N, no; U, unclear. Age in years unless specified otherwise. Mo, months; y, year(s). C/A: children/adults [(Brunner et al.: adults included as comparison group]. DLQI: Dermatology Life Quality Index. EASI: Eczema Area and Severity Index. FLG: filaggrin. Ig: immunoglobulin. IGA: Investigator Global Assessment. IL: interleukin. IQR: interquartile range. NMF: natural moisturizing factor. OR: odds ratio. SASSAD: six sign atopic dermatitis. SC: stratum corneum. SCORAD: Scoring Atopic Dermatitis. SNPs: single nucleotide polymorphisms. TARC: thymus and activation-regulated chemokine. TEWL: transepidermal water loss.
