# Supplementary material for: Classifying atopic dermatitis: a systematic review of phenotypes and associated characteristics
Source: J Eur Acad Dermatol Venereol. 2022 Feb 25;36(6):807–19. doi: 10.1111/jdv.18008 (PMC9307020; doi:10.1111/jdv.18008)
Supplement: Supplementary file 1 — Table S1. Evidence tables per predefined phenotype category. [file JDV-36-807-s005.zip › jdv18008-sup-0002-Table1b.docx]

**Supplementary Table 1b. Evidence table for phenotypes based on disease trajectories**

| Study | Study design | Year | Setting | Country | WHO region | No. | Age -  Mean ± SD (range) | Sex – M/F, no. (%) | Phenotype description | No. (%) per phenotype | Potential associated characteristic(s) (of a priori interest) | Methodological approach | Inclusion of controls (Y (no.)/ N) |
| --- | --- | --- | --- | --- | --- | --- | --- | --- | --- | --- | --- | --- | --- |
| Abuabara 2019 | Cohort | 1958-1970 | Pop | U.K. | European Region | 5,593 | (0-50) | 2509 (44.8) M / 3084 (55.2) F | childhood-onset (positive parental report during or before the last year at age 5-7 and/or 10-11 years) and adult-onset atopic eczema (first report of atopic eczema at age ≥23 years) | childhood-onset: n=3285 (58.7); adult-onset: n=2308 (41.3). | Sex, ethnic group, history of any breast-feeding, region of residence in childhood, region of residence in adulthood, childhood smoke exposure, smoking in adulthood, household size, in utero smoke exposure, birth weight, and the Registrar General’s designation of social class. Personal history of asthma or allergic rhinitis/hay fever; data on parental history of asthma and hay fever (parent’s report at age 5 years); any FLG–null mutation and a non-FLG genetic risk score, total IgE level, and allergen-specific IgE level. | Multivariable logistic regression, meta-analysis. | Y (19,436) |
|  | **Results of the analysis →** | When compared with subjects whose eczema started in childhood, those with adult-onset disease were more likely to be women (OR, 1.66 [95% CI, 1.44-1.92]; p <0.001), from Scotland or Northern England (1.31 [1.01-1.71]; p=0.045), of lower childhood socioeconomic group (1.23-1.38 [1.01-1.89]; p=0.009-0.044), smokers in adulthood (1.20 [1.04-1.38]; p=0.013), and less likely to have a history of asthma (0.79 (0.68-0.93); p=0.004). No significant differences were found for the other characteristics. A high non-FLG genetic risk score predicted childhood-onset atopic eczema, but there was little evidence for an association between the non-FLG genetic risk score and adult-onset disease (1.81 [1.37-2.40] and 1.18 [0.85-1.64], respectively). High allergen-specific IgE levels predicted childhood-onset atopic eczema, but there was little evidence for an association between the allergen-specific IgE and adult-onset disease (1.90 [1.32-2.74] and 0.86 [0.54-1.36], respectively). | | | | | | | | | | | |
| Amat 2015 | Cohort | 2002-2012 | Hos | France | European Region | 214 | 8.51 ± 2.79 mo | 124 (57.9) M / 90 (42.1) F | Three clusters of infants with early-onset AD (age younger than 12 months):  cluster 1 (AD with low sensitization),  cluster 2 (AD with multiple sensitizations),  cluster 3 (AD with familial history of asthma) | Cluster 1: n=94 (44), cluster 2: n=84 (39), cluster 3: n=36 (17). | Asthma at the age of 6 | Data-driven approach to identify phenotypes, by using cluster analysis: hierarchical bottom-up clustering method  Chi-squared test, Fischer's exact test | N |
|  | **Results of the analysis →** | Percentages of children suffering from asthma at the age of 6 were higher in clusters 2 and 3 (36.1% and 33.3% respectively versus 14.9% in cluster 1, p<0.01). [additional information on clusters: cluster 1: low to no sensitization to food (27.7%) or aeroallergens (10.6%) and moderate AD severity (SCORAD 25.29 +/- 14.6); cluster 2: characterized by a higher AD severity (SCORAD 32.66+/-16.6) and frequent sensitization to food (98.9%) or aeroallergens (26.2%), most likely multiple (96.4% for food allergens); cluster 3: parental history, moderate AD severity (SCORAD 24.46+/-15.7), moderate rate of sensitization to food allergens (38.9%) (exclusively single) with no sensitization to aeroallergens.] | | | | | | | | | | | |
| Barker 2007 | Cross-sectional | NR | Hos | U.K. | European Region | 163 | 36.4 (16–82) | NR | Adults with persistent AD, which had been present since early childhood (childhood onset AD persisting into adulthood) | 100% | FLG null mutations: R501X and 2282del4 | Pearson’s chi-squared test, Fisher’s exact tests, binary logistic regression | Y (1463) |
|  | **Results of the analysis →** | The association between the FLG null variants and AD was significant for each of the two common null variants: for R501X, the Chi2 P-value was 1.0x10^-27, for 2282del4 the P-value was 3x10^-26. The combined allele frequency of the two common FLG null variants was 0.270, in contrast to 0.046 in the ethnically matched population controls (OR of 7.7 with 95% CI of 5.3–10.9 and a Chi2 P-value of 1.7x10^-53). | | | | | | | | | | | |
| Bradley (also in severity category) 2002 | Cross-sectional | 1995-1997 | Hos | Sweden | European Region | 470 | Median 32 | 174 (37) M / 296 (63) F | Extreme AD(more severe phenotype): defined as having an early age of onset (at 2 years or younger ), combined with a severity score of 3 or more. Severity scoring: Age at onset <2 years: 1, Hospitalization for AD: 1, Number of sites manifesting AD at examination: 0: 0, 1–3: 1, >3: 2, Raised total and/or allergen-specific serum IgE: 1. | Extreme AD: n=74 (16) | Genomic DNA markers | Genome-wide linkage analysis, linear model | N |
|  | **Results of the analysis →** | Linkage was found for the extreme phenotype and a region on chromosome 18p close to marker D18S542 (logarithm of the odds =1.88, P<0.005), to chromosome region 18q21 (D18S858; logarithm of the odds=1.67, and P<0.005), chromosome regions 21q21 (logarithm of the odds=1.69, P<0.005), 7p14 (logarithm of the odds=1.48, P<0.005) and chromosome region Xp11 close to marker DXS6800 (maximum logarithm of the odds score=2.64, P<0.005). | | | | | | | | | | | |
| Brown 2008 | Cross-sectional | NR | Pop, Hos | U.K. | European Region | 186 | Adults | NR | Adults whose eczema had begun in early childhood (early-onset (atopic) eczema) | 100% | FLG mutations: R501X, 2282del4, R2447X, S3247X, 3702delG | Fisher’s exact test, logistic regression | Y (1035) |
|  | **Results of the analysis →** | Three FLG variants are strongly and independently associated with early-onset persistent eczema: R501X (P=3.2x10^-17, odds ratio (OR) 5.6, 95% CI 3.7–8.3), 2282del4 (P=7.1x10^-10, OR 4.1, 95% CI 2.7–6.4), and R2447X (P=7.8x10^-5, OR 7.3, 95% CI 2.8–18.7). A novel FLG null mutation was identified (3673delC) in one eczema case (not in any controls). Rarer variants S3247X, 3702delG, and 3673delC were individually not significantly associated. Combined null genotype of all six mutations was significantly associated with early-onset persistent eczema (P=1.3 x 10^-28, OR 5.6, (95% CI 4.1–7.8)) (allele model). Combined null genotype showed OR of 85.9 (95% CI 20.1–367.6) for homozygotes and compound heterozygotes, as opposed to 4.6 (3.1–6.8) for heterozygotes (genotype model). | | | | | | | | | | | |
| Brunner (also in severity category) 2018 | Cross-sectional | NR | Hos | U.S.A. | Region of the Americas | C: 19 (A: 20) | C: 1.3 ± 1.2 (3-5) (A: 47.6 ± 14.5) | C: 11 (58) M / 8 (42) F (A: 12 (60) M / 8 (40) F) | Early-onset pediatric moderate-to-severe AD: defined as patients <5 years old with <6 months disease duration and based on SCORAD (mean 57.8 and range 33-84) | 100% of children | Transcriptional and barrier differences in skin | Mixed-effect models | Y (29) |
|  | **Results of the analysis →** | Pediatric early-onset moderate-to-severe AD also showed significant TH17/TH22 skewing but lacked the TH1 upregulation that characterizes adult AD. Pediatric AD exhibited relatively normal expression of epidermal differentiation and cornification products, which is downregulated in adults with AD. Some lipid-associated mediators (e.g., fatty acyl-CoA reductase 2 and fatty acid 2-hydroxylase) showed preferential downregulation in pediatric AD, and lipid barrier genes (FA2H and DGAT2) showed inverse correlations with TEWL. | | | | | | | | | | | |
| Dezman 2017 | Cross-sectional | 2009-2012 | Hos | Slovenia | European Region | 241 | 23.5 ± 12.2 | 75 (31.1) M / 166 (68.9) F | Early-onset AD (≤8 years) and late-onset AD (>8 years) | Early onset: n=117 (49), late-onset: n=124 (51) | SNPs rs2303067 in SPINK5 and rs4950928 in CHI3L1  (see results) | Chi-squared  test for the goodness-of- fit model, Fisher’s exact test | Y (164) |
|  | **Results of the analysis →** | rs2303067 in SPINK5: Patients with early onset AD had higher frequencies of A allele compared with controls (56% vs. 47%; p = .033; OR = 1.44, 95% CI 1.03–2.02). There was lower frequency of the GG genotype in early onset AD (13%) compared with controls (27%) and late onset AD (24%). Early onset AD subjects had a higher frequency of combined AA+AG genotypes compared to controls (p = .003; OR = 2.57, 95% CI 1.35−4.88) and to late onset AD subjects (p = .031; OR = 2.17, 95% CI 1.10−4.23). No differences were observed between late onset AD subjects and controls. rs4950928 in CHI3L1: The frequency of combined GG+CG genotypes was higher in late onset AD (42%) than controls (30%) (p = 0.048; OR = 1.65, 95% CI 1.01−2.68). [Rs2303067 in SPINK5 is involved in skin barrier functioning (encoding a skin barrier protein) and rs4950928 in CHI3L1 is involved in immune response regulation.] | | | | | | | | | | | |
| Dezoteux (also in morphology characteristics group) 2019 | Cross-sectional | 2007-2016 | Hos | France | European Region | 533 | ≥ 18 years; AD≥45: range: 45-78 | AD≥45: 66 (53) M / 58 (47) F | Atopic dermatitis (AD) in adults over 45 years of age (AD≥45) and below 45 years (<45); Among AD≥45 patients, 20% (n=25) were categorized into Subgroup 1 (persistence of AD since childhood), 52% (n=64) into Subgroup 2 (recurrence of AD with a history of classic childhood AD), and 28% (n=35) into Subgroup 3 (adult-onset AD). | AD≥45: n=124 (23), AD<45: n=409 (77). | Age, gender, level of education, association with asthma and/or allergic rhinoconjunctivitis, family history of atopy, active smoking, and predominant skin region affected by AD lesions  (eczema, redness, oedema, lichenification, excoriations). | Chi-squared test, Fisher’s exact  Test, Kruskal-Wallis test | N |
|  | **Results of the analysis →** | Skin lesions predominated on the face and neck in AD≥45 patients with AD since childhood (30% in Subgroups 1 and 2) compared to those with adult-onset AD (14% in Subgroup 3) (no p-value reported). Gender (p=0.78), asthma and/or allergic rhinoconjunctivitis (73%, 76% and 75% 3 in Subgroups 1, 2 and 3, respectively; p = 0.94), a family history of atopy (83%, 84% and 79% in Subgroups 1, 2 and 3, respectively; p = 0.88), active smoking (24%, 21% and 23% in Subgroups 1, 2 and 3, respectively; p = 0.93), and level of education (17%, 36% and 29% in Subgroups 1, 2 and 3, respectively; p = 0.27) were similar in the three subgroups regarding AD onset. | | | | | | | | | | | |
| Esaki 2016 | Cross-sectional | NR | NR | U.S.A | Region of the Americas | 34 | (0.6 mo – 57 y) | 18 (52.9) M / 16 (47.1) F | New/early-onset pediatric AD: defined as children with AD younger than 5 years within 6 months of disease onset | New/early-onset pediatric AD: n=19 (56), adults: n=15 (44) | Immune and epidermal factors in skin biopsies | Linear models, 2-tailed t-test, | Y (32) |
|  | **Results of the analysis →** | In lesional skin children showed comparable or greater epidermal hyperplasia (thickness and keratin 16) and cellular infiltration (CD3+, CD11c+, and FcεRI+) than adults with AD. Similar to adults, strong activation of the TH2 (IL-13, IL-31, and CCL17) and TH22 (IL-22 and S100As) axes and some TH1  skewing (IFN-g and CXCL10) were present. Children showed significantly higher induction of TH17-related cytokines and antimicrobials (IL-17A, IL-19, CCL20, LL37, and peptidase inhibitor 3/elafin), TH9/IL-9, IL-33, and innate markers (IL-8) than adults (P < .02). Despite the characteristic downregulation in adult AD patients, FLG expression was similar in children with AD and healthy children. Nonlesional skin in pediatric AD patients showed higher levels of inflammation (particularly IL-17A and the related molecules IL-19 and LL37) and epidermal proliferation (keratin 16 and S100As) markers (P < .001 ). | | | | | | | | | | | |
| Esparza-Gordillo 2013 | Cohort | 1990-2005 | Hos, Pop | U.K., Germany | European Region | 1697 | NR | NR | Persistent vs transient AD in children with early-onset AD (<3 years): to determine the course of AD, the period between ages 4 and 14 years was divided into 3 time intervals: 4 to 6 years, 7 to 9 years, and 10 to 14 years. Subjects with early AD plus 2 or 3 positive scores at later age intervals were defined as having persistent AD. Transient AD was defined by early AD plus 0 or 1 positive intervals at later age. | Transient course: n=774 (46), persistent course: n=813 (48) | IL-6R 358Ala allele | Additive model with MQLS and PLINK | Y (2095) |
|  | **Results of the analysis →** | IL-6R 358Ala predisposes to the persistent form of AD (ORpersistent AD = 1.22, 95% CI 1.10-1.34, P = .0008; ORtransient AD = 1.04, 95% CI 0.91-1.16, P = .54). | | | | | | | | | | | |
| Ezzedine (both morphology groups, disease trajectory group) 2012 | Cross-sectional | 2007-2008 | Hos | France | European Region | 110 | 36.6 ± 16.2 (18–85) | 49 (45) M / 61 (55) F | Adult caucasian AD patients with or without ichthyosis vulgaris (IV) and with or without early onset (≤ 2 years vs > 2 years) | AD+IV: n=74 (67), AD-IV: n=36 (33)  Early onset: n=55 (50), late onset: n=28 (25), missing data for onset: n=23 (21) | Clinical signs of IV scored 0 (not present) to 3 (very severe) – diffuse xerosis, hyperlinearity of palms, scales on legs, scalp desquamation and keratosis pilaris (KP). Global IV clinical severity score (0–15). Age of onset of AD, SCORAD, family/personal history for AD, allergic rhinitis, allergic conjunctivitis or asthma, and total IgE. FLG mutations R501X, S2282del4, S3247X and R2447X. | Univariate and sub sequent multivariate  unconditional logistic regression analysis | N |
|  | **Results of the analysis →** | Univariate analysis: family history of atopy (OR 4.57, P = 0.01), xerosis (OR 4.67, P = 0.0003), PH (OR 10.71, P < 0.0001), scale on legs (OR 11.00, P < 0.0001), age (OR 2.15, P < 0.0001) and 2282del4 FLG (OR 5.41, P = 0.0010) mutation were positively correlated with the AD + IV phenotype. Multivariate analysis: only SCORAD for AD (OR 0.94, P = 0.01) and global clinical severity scoring for AD + IV (OR 2.62, P < 0.0001) were found to be independent factors. No significant differences were found for sex, R501X mutation, presence of other atopic symptoms, scalp desquamation, KP, age of onset, total IgE and presence of specific IgE between the groups. No S3247X or R2447X mutations were detected. 2282del4 mutation was significantly associated with early-age onset (OR 4.87, P = 0.04). No significant association between R501x mutation and age of onset | | | | | | | | | | | |
| Flohr (also in disease severity category) 2010 | Cross-sectional | 2009-2010 | Pop | U.K. | European Region | 29 | 3 mo | NR | Early-onset eczema at 3 months of age and with mild or moderately severe disease: based on SCORAD < 15 or ≥ 15 | SCORAD < 15: n=23 (79), SCORAD ≥ 15: n=6 (21) | TEWL, FLG mutations: R501X, 2282del4, R2447X and S3247X | Mann–Whitney U-test | Y (59) |
|  | **Results of the analysis →** | TEWL was higher in children with early-onset eczema compared with unaffected infants (median TEWL 14.24 vs. 11.24, P < 0.001). Higher TEWL was associated with more severe disease (median TEWL, SCORAD < 15, 13.1 vs. 29.6, SCORAD ≥ 15, P = 0.029). FLG mutation carriers were more likely to have early-onset eczema (OR 4.26, 95% CI 1.34–13.57, P = 0.014). | | | | | | | | | | | |
| Garmhausen (also in morphology characteristics category) 2013 | Cross-sectional | NR | Hos | Germany | European Region | 725 | 32.6 ± 14.0 (12–89) | 293 (40) M / 432 (60) F | Course type of AD based on age of onset and affection during the following phases (phases based on classification of Wuthrich): infantile phase 0-2 years, childhood phase 2-6 years, juvenile phase 6-14 years, adolescent phase 14-20 years, adult phase > 20 years.  Five main course types were identified: early onset of AD before the 2nd year of life and a chronic persisting course until adulthood (type 5); start of AD after the 20th year of life (type 31); start AD in childhood phase (2-6years) with a chronic persisting course until adulthood (type 20); start in adolescent phase (14-20 years) with a chronic persisting course until adulthood (type 30); start in juvenile phase (6-14 years) with a chronic persisting course until adulthood (type 27). | 607 patients could be classified into course types. Of these 607 patients 85.7% could be classified into five main different course types of all 31 course types recorded.  Type 5: n=189 (31.1). Type 31: n=112 (18.5). Type 20: n=84 (13.8), type 30: n=77 (12.7), type 27 n=58 (9.6). | Atopy signs and concomitant atopic diseases (criteria of Diepgen, including morphological features and serum IgE) | Logistic or linear  regression | N |
|  | **Results of the analysis →** | More food intolerance in course type 5 in comparison with the other four most frequent course types (p<0.05). Type 5 more often had flexural eczema (compared to course type 30; P = 0.0003 and course type 31 P < 0.0001). In comparison with course type 31, course type 5 showed a significantly higher occurrence of rhinoconjunctivitis (P < 0.0001), milk crust (P < 0.0001), Hertoghe sign (P = 0.0091), Dennie–Morgan infraorbital fold (P = 0.001), orbital darkening (P = 0.0007), white dermographism (P = 0.0031), predilection of skin lesions in the neck region (P = 0.0206), impact of psychic factors on the course of their disease (P = 0.0105) and asthma bronchiale (P < 0.0001). Higher total serum IgE levels in course type 5 in comparison to types 27, 30 and 31 (p<0.05). Allergen-specific IgE towards peanut, hazelnut and apple, cat dander, birch and grass pollen was more frequent in type 5 than the other four types (p<0.05) Non-allergic AD (low IgE serum levels (<150kU/l) and no sensitisation to aero or food allergens) was found in 25% of course type 31, significantly higher than course type 5 (6.4% P<0.0001). | | | | | | | | | | | |
| Greisenegger 2010 | Cross-sectional | NR | Hos | Austria, Germany | European Region | 462 | Median: 31 (quartiles: 23-41) | 171 (37) M / 291 (63) F | Austrian and German AD patients with early age of disease onset (≤ 2 years of age) and late onset (>2 years of age) | Early onset: n=178 (39), late onset: n=148 (32)  (Missing: n=136 (29)) | FLG mutations for R501X, R2247X, S3247X and 2282del4 | Logistic regression models | Y (402) |
|  | **Results of the analysis →** | Strong association of the combined genotype (defined as: presence of at least one of the four considered mutations) with an early age of disease onset (OR = 2.50, CI: 1.45–4.31, P = 0.001). | | | | | | | | | | | |
| Guglielmo (both morphology groups, disease trajectory group) 2020 | Cross-sectional | 2005-2020 | Hos | Italy | European Region | 31 | (11-62) | 16 (52) M / 15 (48) F | Adolescent-onset and adult-onset head and neck dermatitis (HND) (cut-off at 18 years) | Adolescent-onset HND: n=17 (55), adult-onset HND: n=14 (45), head and neck dermatitis: 100% | Age, sex, HND onset, AD distribution (exclusive HND vs diffuse AD including HND involvement), past medical history of AD or atopy (personal or family), IgE serum levels | Fisher exact test, t-test | N |
|  | **Results of the analysis →** | Adolescent-onset HND positively correlated with a past history of AD of the classic type and presented with exclusive head and neck involvement (100% and 59%, respectively) (P < .05). Adult-onset HND was associated with concomitant widespread atopic eczema, involving the flexural areas of the upper and lower limbs, trunk, nipples, or hands (68%) (P < .05). Increased serum IgE level (>100 IU/mL) was detected in 11/17 (65%) adolescents and 11/14 (78%) adult patients. No differences were observed between the two groups in terms of AD family history or personal atopy history, including asthma, food allergy, allergic rhinitis, or conjunctivitis. | | | | | | | | | | | |
| Hagendorens 2004 | Cross-sectional | NR | Hos | Belgium | European Region | 9 | (0-1) | NR | Occurrence of AD during the first year of life | 100% | Absolute number of lymphocyte and cytokine subtypes | Chi-squared, Mann–Whitney U-test | Y (19) |
|  | **Results of the analysis →** | An increased number of naive CD4+ CD45RA+ lymphocytes (median values and range (10^6 /l): 1373 (573–2401) vs. 880 (432–1674); p = 0.03) was found in children with AD occurrence during the first year, with a decreased basal IL-4 production by CD8+ lymphocytes (median values and range (%): 1.3 (0.7–2.0) vs. 0.7 (0.0–1.6); p = 0.02) and with a decreased percentage of IFN-γ producing stimulated CD4+ lymphocytes (median values and range (%): 2.6 (0.9–6.4) vs. 5.6 (1.3–12.4); p = 0.04). Absolute number of cord blood lymphocytes and other lymphocyte subsets analysed (CD19+ lymphocytes, CD3+ lymphocytes, CD3+CD4+ lymphocytes, CD3+CD8+lymphocytes, CD3+CD4+CD45RO+ lymphocytes) was comparable between children with and without occurrence of AD during the first year of life. | | | | | | | | | | | |
| Holm (in trajectory, severity and morphological characteristics group) 2019 | Cross-sectional | 2012-2017 | Hos | Denmark | European Region | 470 | 18.7 ± 16.5 | 214 (45.5) M / 256 (54.5) F | AD subgroups based on SCORAD: mild (< 25), moderate (25–50) and severe (> 50); small children (< 4 years of age), children/adolescents (age 4–15 years) and adults (> 15 years of age); early-onset (< 1 year of age), late-onset (>1 year of age) | Small children: n=122 (26), children/adolescents: n=103 (22) and adults: n=245 (52).  Mild: n= 166 (35.3), moderate: n= 218 (46.4), severe: n= 86 (18.3).  Early-onset: n=141 (47.8), late-onset: n=154 (52.2). | FLG mutations (R2447X, R501X and 2282del4), serum total IgE, blood eosinophil count; self-rated health, eczema distribution in the past month | ANOVA, chi-squared test, independent t-test | N |
|  | **Results of the analysis →** | A significant difference between severity groups in small children was observed for FLG mutation carrier status (16.7 vs. 30.2 vs. 60.0% mutation carriers among patients with mild, moderate and severe AD, respectively, p = 0.012) and self-rated health (3.2 vs. 2.7 vs. 2.8 with 4 being excellent health, p = 0.022).  In the subgroup of children/adolescents, disease severity was statistically significantly associated with CDLQI (5.6 vs. 7.3 vs. 8.8 among patients with mild, moderate and severe AD, respectively, p = 0.048). A significant difference between severity groups in adults was observed for male sex (24.4 vs. 39.8 vs. 52.9%, p = 0.003), serum total IgE (577 vs. 1269 vs. 2379 × 103 IU/L, p < 0.001), blood eosinophil count (0.28 vs. 0.39 vs. 0.61 × 109/L, p < 0.001) and asthma (42.9 vs. 38.8 vs. 72.0%, p < 0.001), hand eczema (66.2 vs. 81.6 vs. 88.0%, p = 0.007), flexural eczema (67.5 vs. 74.7 vs. 90%, p = 0.015), and DLQI (6.9 vs. 9.9 vs. 13.8, p < 0.001). Compared to no early onset of AD and no FLG mutations, early onset of AD and FLG mutation was associated with more severe disease (higher mean SCORAD (33.3 vs. 41.6, p = 0.012)) and high serum total IgE levels (903 vs. 1961 × 103 IU/L, p = 0.047). | | | | | | | | | | | |
| Hu 2019 | Cohort | 2002-2016 | Pop | The Netherlands | European Region | 1,279 | (6 mo-10 y) | Total population: 2632 (50) M / 2665 (50) F | Early transient, mid-transient, late transient and persistent eczema from age 6 months until 10 years | Early transient: n=439 (8), mid-transient: n=306 (6), late transient: n=412 (8), persistent: n=122 (2). | Socioeconomic and lifestyle exposures in early-life and genetic risk factors | Data-driven approach to identify phenotypes by using latent class growth analysis.  Weighted mutually adjusted multinomial regression models | Y (4,018) |
|  | **Results of the analysis →** | Early transient and persistent eczema were most common in first-born children, those with a parental history of eczema, allergy or asthma and those with persistent wheezing [range of OR: 1.37, 95% CI 1.07–1.74 and OR 3.38, 95%CI 1.95–5.85], compared with ‘never eczema’. Early transient eczema was most common in male children only (OR 1.49, 95% CI 1.18–1.89). Children with late transient or persistent eczema were more often of Asian ethnicity (OR 2.04, 95% CI 1.14–3.65 and OR 3.08, 95% CI 1.34–7.10, respectively), compared with ‘never eczema’. Children with early transient, late transient and persistent eczema more often had a filaggrin mutation or additional risk alleles (range OR: 1.07, 95%CI 1.02–1.12 and OR 2.21, 95%CI 1.39–3.50), compared to children with ‘never eczema’. Eczema phenotypes were not associated with maternal education, breastfeeding, day care attendance and pet exposure. | | | | | | | | | | | |
| Hu 2020 | Cohort | 2002-2016 | Pop | The Netherlands | European Region | 1,270 | (6 mo-10 y) | 659 (52) M / 611 (48) F | Early transient, mid-transient, late transient and persistent eczema phenotypes from age 6 months until 10 years | Early transient: n=434 (34), mid-transient: n=302 (24), late transient: n=412 (32), persistent eczema: n=122 (10). | Emotional (internalizing) and behavioural (externalizing) problems using the Child Behavior Checklist | Data-driven approach to identify phenotypes by using latent class growth model.  Chi-squared test, independent samples t-test, Mann–Whitney U-test, linear regression models,cross-lagged models. | Y (3,995) |
|  | **Results of the analysis →** | All eczema phenotypes were associated with more internalizing problems and attention problems at age 10 years, compared with never having eczema: range of Z-score differences 0.14 [95% CI 0.01–0.27] to 0.39 (95% CI 0.18–0.60). Children with early transient eczema had more externalizing problems (Z = 0.16, 95% CI 0.04–0.27), including aggressive behaviour symptoms at age 10 years (Z = 0.16, 95% CI 0.05–0.27). Bidirectional analysis showed that eczema at 0–2 years was associated with more internalizing and externalizing problems at ages 3–6 and 10 years, while only internalizing problems at 0–2 years were associated with an increased risk of eczema at age 10 years. | | | | | | | | | | | |
| Hu 2020 | Cohort | 2002-2016 | Pop | The Netherlands | European Region | 1,048 | (6 mo-10 y) | Total population: 2096 (49) / 2181 (51) F | Early transient, mid-transient, late transient and persistent eczema phenotypes from age 6 months until 10 years | Early transient: n=363 (9), mid-transient: n=259 (6), late transient: n=333 (8), persistent: n=93 (2). | Allergic sensitization, physician-diagnosed allergy and asthma and lung function at age 10 | Data-driven approach to identify phenotypes by using latent class growth model.  Adjusted linear, logistic and multinomial regression models. | Y (3,229) |
|  | **Results of the analysis →** | Compared with never eczema, all eczema phenotypes were associated with increased risks of asthma (OR range (95% CI): 2.68 (1.58, 4.57) to 11.53 (6.65, 20.01)), food and inhalant allergic sensitization (1.72 (1.25, 2.36) to 12.64 (7.20, 22.18)), and physician-diagnosed inhalant allergy (1.92 (1.34, 2.74) to 11.91 (7.52, 18.86)). Strongest effect estimates were observed of early transient and persistent eczema with the risk of physician-diagnosed food allergy (OR 6.95 (3.76, 12.84) and 35.05 (18.33, 70.00), respectively) and combined asthma and physician-diagnosed allergy (7.11 (4.33, 11.67) and 29.03 (15.27, 55.22), respectively). Eczema phenotypes were not associated with lung function measures. | | | | | | | | | | | |
| Just 2014 | Cohort | 2002-2012 | Hos | France | European Region | 229 | 6.5 ± 2.7 mo | 134 (59) M / 95 (41) F | Infants with early-onset (children aged younger than 12 months) active AD (without a history of wheezing) | 100% | Biological markers of atopy: allergic sensitization defined as the presence of positive specific IgEs to allergens and multiple sensitizations as being sensitized to ≥2 allergens. Elevated blood eosinophilia was defined as an eosinophil blood count ≥470 eosinophils/mm3 and elevated total IgE as a serum IgE level ≥45 kU/l. | Chi-squared test, Fisher’s exact test, Welsh’s student test, logistic  regression model | N |
|  | **Results of the analysis →** | Elevated blood eosinophilia was observed at baseline in 60 children (26.2%) and elevated total IgE in 85 (37.1%). Fifty-eighty per cent (132/229) of the children had sensitization to food allergens and 37% (86/229) multiple sensitizations. When elevated at baseline, eosinophilia and IgE levels remained significantly higher during the follow-up period compared to those with a low level at baseline (+705/mm3 vs. +76.1/mm3 over the follow-up period, p < 0.001 and +284.6 kU/l vs. +39.2 kU/l over the follow-up period, p < 0.001, respectively). Elevated total IgE at baseline emerged as a risk factor for developing sensitization to inhaled allergens at the end of follow-up (OR 2.94 [1.58–5.47] p < 0.001). Sensitization to food allergens decreased from 58% (132/229) to 34% (66/195), whereas sensitization to inhaled allergens increased over time from 17% (39/229) to 67% (130/195). One hundred infants (76.9%) sensitized to food allergens were sensitized to inhaled allergens at 6 yr (OR 3.32 [1.90–5.84] p < 0.001). Infants with multiple sensitizations to food allergens were more likely to be sensitized to inhaled allergens (OR 4.32 [2.22–8.40] p < 0.001) than infants with a single food sensitization (OR 2.20 [1.05–4.60] p = 0.035). Initial multiple sensitizations to food allergens were the most predictive factor for the risk of developing sensitization to inhaled allergens at 6 yr (OR 3.72 [1.68–8.30] p < 0.001). The risk was almost double than that for children with one food sensitization (OR 2.20 [1.01– 4.72] p = 0.05). | | | | | | | | | | | |
| Kulthanan (also in morphology characteristics category) 2011 | Cross-sectional | 2006-2008 | Hos | Thailand | South-East Asian Region | 56 | 34.1 ± 11.7 (18-72) | 10 (15) M / 46 (82) F | Extrinsic and intrinsic types of adult-onset AD in Thai patients, based on total serum IgE levels <200 kU/L, no specific serum IgE antibodies and/or negative SPT for common aeroallergens and/or food allergens, and the absence of associated respiratory atopic diseases = intrinsic form: iAD or atopiform dermatitis; other patients = extrinsic form; eAD. | Adult-onset: 100%  eAD: n=49 (87.5), iAD: n=7 (12.5) | “Clinical and diagnostic features”, including the Hanifin and Rajka criteria (including morphological features) | Pearson’s chi–squared test, Fisher exact test, unpaired t-test, Mann-Whitney U test | N |
|  | **Results of the analysis →** | eAD more commonly had typical lichenified/exudative eczematous lesions, especially on the antecubital and popliteal (flexural) areas, when compared with patients with iAD. Nummular and follicular lesions were more commonly seen in iAD group than the eAD group. Lesions on antecubital and popliteal area were detected more commonly in eAD (62.8% and 30.2% respectively). The most common area of involvement in iAD was non-flexural area (71.4%) especially trunk and legs (57.1% each), followed by flexural area (57.1%) and extensor area (42.9%). eAD frequently had flexural lichenification when compared with iAD (p=0.002), and had a statistically significant higher percentage of personal history of atopy (p<0.001), especially allergic rhinitis (p=0.001), than iAD. Ichthyosis, cutaneous infection, non-specific hand and foot dermatitis, Dennie-Morgan infraorbital fold, orbital darkening, facial pallor, anterior neck folds, itch when sweating, course influenced by environment or emotional factors, intolerance to wool and lipid solvent or any coarse fabric were commonly seen in iAD when compared with eAD (although not significant). | | | | | | | | | | | |
| Lee 2016 | Cohort | 2005-2006 | NR | Korea | Western Pacific Region | 242 | 7.54 ± 0.93 (6-8) | 125 (51.7) M / 117 (48.3) F | Four current AD phenotypes in school children based on latent class analysis: ‘early onset with low atopy’ (group 1), ‘early onset with high atopy and high eosinophil percentages’ (group 2), ‘late onset with low atopy’ (group 3), ‘late onset with high atopy and normal eosinophils’ (group 4) | Group 1: n=64 (26.4), group 2: n=117 (48.3), group 3: n= 24 (9.9), group 4: n=37 (15.3) | Asthma, serum IL-13 and thymic stromal lymphopoietin (TSLP). | Data driven approach to identify phenotypes, by using latent class analysis  ANOVA, Kruskal–Wallis, chi-square tests | Y (NR) |
|  | **Results of the analysis →** | Although groups 2 and 4 demonstrated high atopic burden, children in group 2 showed the persistence of AD (adjusted OR for AD remission = 0.310; 95% CI = 0.115–0.832) and eosinophilia (p<0.05) and was associated with a high prevalence of new cases of bronchial hyper-responsiveness (25.5%, p=0.017) and asthma (10.0%, n.s.) during follow-up. The serum IL-13 level was significantly increased in the early-onset AD groups (control group vs group 1, p = 0.035; control group vs group 2, p = 0.025), but there was no significant difference in the serum TSLP levels across all four groups. | | | | | | | | | | | |
| Loo 2015 | Cross-sectional | 2009-2010 | Pop | Singapore | Western Pacific Region | 187 | (0-18 mo) | (total population: 51.9% male) | Three phenotypes of AD based on the time of disease onset in an Asian mother-offspring cohort: early AD occurring within the first 6 months of life, AD occurring between 6 and 12 months and late-onset AD starting after the age of 12 months | Diagnosed before the age of 6 months: n=100 (53.5), diagnosis  between 6 and 12 months: n=43 (23.0), diagnosed after 12 months of age: n=47 (25.1) | Various characteristics related to family and exposure: maternal consumption of alcohol, maternal/paternal/sibling allergic history, maternal education level, ethnicity, vaginal delivery, antibiotic treatment in first 6 months of life, attended daycare, having a cat/dog, female gender, living near an expressway, having siblings, prenatal exposure to smoke, consumption of probiotics between 9 and 12 months, antibiotic treatment in the first 6 months, sum of skinfold thickness, birth weight/length, monthly household income | Logistic regression | Y (605) |
|  | **Results of the analysis →** | Maternal allergic history was associated with an increased risk of developing early-onset AD (adjusted odds ratio (aOR) 20.46, 95% confidence interval (CI) 2.73–153.15, p < 0.01). Maternal allergic history and attendance at a daycare centre increased the odds of the development of AD between 6 and 12 months (aOR 4.19, 95% CI 1.01–17.45, p = 0.049 and aOR 11.42, 95% CI 1.49–87.50, p = 0.02, respectively). Risk factors associated with increased odds of late-onset AD from 12 months were the consumption of probiotics between the age of 9 and 12 months and antibiotic treatment in the first 6 months of life (aOR 4.32, 95% CI 1.07–17.45, p = 0.04 and aOR 3.11, 95% CI 1.10–8.76, p = 0.03, respectively). Early-onset AD was associated with an increased risk of developing allergic sensitization (aOR 46.51, 95% CI 3.44–628.81, p < 0.01). | | | | | | | | | | | |
| Lou 2019 | Cohort | 2004-2025 | Hos | U.S.A. | Region of the Americas | PEER cohort: 741; GAD cohort: 337 | GAD cohort: mean: 33.6 years (SD: 21.7) (including persons without AD) | PEER cohort: (43.3) M / (56.7) F | Pediatric-onset AD, remission | 100% | Thymic stromal lymphopoietin (TSLP) variation | Chi-Square or logistic regression. | Y (238 (GAD cohort)) |
|  | **Results of the analysis →** | The minor variant frequency for rs1898671 was 23.5% (95% CI: 21.4, 25.8). White children with (pediatric-onset) AD were less likely to have rs1898671 variant (OR: 1.41 (1.20, 1.66)) than controls, but African-American children are equally likely (1.00 (0.73, 1.13) as AD African population. In the second cohort (N=585, GAD), the rs1898671 variant was less prevalent in those with (pediatric-onset) AD than those without. The protective effect was found to be greater in rs1898671 heterozygotes (OR: 1.91 (1.34, 2.75)) than homozygotes (OR: 1.28 (0.61, 2.70)). Children with the rs1898671 variant at any given survey were more likely to have a remission than children who were wildtype for rs1898671 (OR: 1.56; 95% CI: 1.26, 1.91). This effect was greater for African-American than white children and appears to be greater in those with a FLG loss of function variant. Neither of the haplotypes nor the individual SNPs associated with AD remission. | | | | | | | | | | | |
| Luukkonen (also in morphology phenotype) 2017 | Cross-sectional | 2000-2013 | Hos | Finland | European Region | 445 | 32.3 ± 14.9 | (37.2) M / (62.8) F | AD with and without early-onset (< 2 years), AD with and without PH, AD with and without KP | Early-onset AD: n=297 (74.8), AD with PH: n=143 (40.6), AD with KP: n=48 (14.2) | The 4 most prevalent European FLG null mutations (2282del4, R501X, R2447X, S3247X), 2 FLG mutations (S1020X, V603M)  enriched in the Finnish population, the 12-repeat allele (rs12730241) and 59 additional epidermal barrier gene variants) | Fisher’s exact test, logistic regression, linear model | Y (1710) |
|  | **Results of the analysis →** | Early-onset AD and PH showed significant associations with the combined FLG null genotype: The combined FLG null genotype was significantly associated with early-onset AD (< 2 years of age) (OR 4.15, p = 1.82 × 10–10) and PH (OR 4.67, p = 1.46 × 10–5), and suggestively associated with KP (OR 3.1, p = 0.0021). Regarding the individual mutations: Mutation 2822del4 was significantly associated with early-onset AD (OR 3.38, p = 8.38 × 10–6). R501X was associated with early-onset AD (OR 14.88, p = 0.00079), and suggestively with KP (OR 13.09, p = 0.0035). R2447X showed suggestive association with early-onset AD (OR 3.86, p = 0.0018). | | | | | | | | | | | |
| Martin 2015 | Cross-sectional | 2007-2011 | Pop | Australia | Western Pacific Region | 1806 | (11-15 mo) | 977 (54) M / 763 (42) F | Early eczema onset (<3 months) in one-year-old infants, further stratified into patients who did and did not required doctor-prescribed topical corticosteroid treatment | Early eczema onset: n=420 (23.2), patients who required doctor-prescribed topical corticosteroid treatment: n=167 (9.2 | Food allergy | Multivariate logistic regression | Y (2795) |
|  | **Results of the analysis →** | Infants with eczema diagnosed within the first 3 months after birth, and who required prescription topical corticosteroid treatments from a doctor, had the highest risk of food allergy (50.8%, 95% CI 42.8, 58.9). | | | | | | | | | | | |
| McAleer 2019 | Cross-sectional | 2012-2014 | Hos | Ireland | European Region | 100 | (0-11 mo) | Plasma: 34 (72) M / 13 (28) F;  stratum corneum: 49 (74) M / 17 (26) F | Infants (< 12 months of age) at first presentation with AD, for ≥ 6 weeks’ duration. | 100% | Multiple cytokines and chemokines and natural moisturizing factor measured in the stratum corneum and plasma. | Welch’s t-test, Mann–Whitney test | Y (20) |
|  | **Results of the analysis →** | Nineteen of 27 stratum corneum and 12 of 39 plasma biomarkers showed significant differences between healthy and (infants’ at first presentation of) AD skin.For example, CCL17 and CCL22 were elevated in AD in both stratum corneum and plasma. However, several inflammatory biomarkers were significantly different from healthy controls only in the stratum corneum; these included IL-1a, IL-1b, IL-15, IL-16, IL-18, IL- 12p40, SAA; chemokines CXCL8, CCL2 and CCL4; cell adhesion biomarkers sVCAM-1 and sICAM-1 and vascular factors VEGF-A and Flt-1. In contrast, VEGF-C, Tie-2 and CCL13 were significantly elevated in plasma and undetectable or unchanged in the stratum corneum. In the skin compartment the most significantly elevated  biomarkers in patients with AD were IL-18, CXCL8, Flt-1, CCL22, CCL17 and sICAM-1. The most significant decreases were observed in IL-15, GM-CSF, CCL4, IL-12p40, IL-1a, IL-13 and IL-5. In plasma, the biomarkers CCL17, IL-5, VEGF-C, GM-CSF and CCL26 had the most significant fold increases in  plasma of patients with AD compared with healthy controls, while IL-4 and CXCL10 had the most significant decreases. | | | | | | | | | | | |
| McKenzie 2019 | Cohort | 1998-2015 | Pop | U.S.A. | Region of the Americas | Range: 2,188-2,651 | (5-15) | Unclear | Atopic dermatitis onset was classified as early (age 5 years),  intermediate (age 9 years), and late (age 15 years) by the first year in which AD was reported. Transient AD was determined as AD reported in only a single year. Resolving AD was determined as AD reported at ages 5 and 9, but not 15 years. Early-onset, persistent AD was determined as AD present at all 3 years. | Early-onset, transient: (17.7%); early-onset, intermittent: (5.8%); early-onset, resolving: (14.2%); early-onset, persistent: (13.0%); intermediate onset, transient: (20.8%); intermediate onset, persistent: (6.5%); late onset: (22.0%). | Sociodemographics (sex, race/ethnicity, and household income), asthma history at age 15 years/lifetime, overall health status (poor/fair/good vs very good/excellent) | Logistic regression models | Y (unclear) |
|  | **Results of the analysis →** | A greater proportion of females (54.8% [40.2%-69.4%]) reported early AD onset. Alternatively, a greater proportion of boys (26.1% [9.3%-42.9%]) reported late-onset AD. Those with a household income less than 99% FPL had the greatest proportion of early (68.8% [51.2%-88.4%]) and the smallest proportion of late (18.2% [1.2%-35.2%]) AD onset. Female sex (multivariable repeated measures logistic regression; adjusted odds-ratio [95% CI]: 1.56 [1.02-2.37]) and black race (1.80 [1.07-3.01]) and multiracial/other (1.94 [1.00-3.78]) race, but not Hispanic ethnicity (0.99 [0.52-1.86]) or any household income groups (100%-199%: 0.56 [0.30-1.04]; >200%: 1.20 [0.63-2.29]) were associated with persistent AD across all 3 ages. Children with AD at ages 5 and 15 (2.63 [1.42-4.86]), 5, 8 and 15 (1.47 [1.02-2.12]) and 9 and 15 years (1.61 [1.00-2.60]) had higher odds of poor/fair/good overall health. Children with AD at ages 5 and 9 years had the highest odds of ever having asthma (adjusted odds ratio [95% confidence interval]: 6.05 [5.88-6.22]), followed by children with AD at ages 5, 9, and 15 years (3.17 [3.07%-3.27]). | | | | | | | | | | | |
| Megna (also in morphology characteristics category) 2017 | Cross-sectional | 2015-2016 | Hos | Italy | European Region | 253 | Persistent AD: 31.9 ± 11.6, adult-onset AD: 36.5 ± 12.4 | 140 (55) M / 113 (45) F | Persistent vs adult-onset AD in adults (persistent: onset < 18 years of age; adult-onset: onset ≥18 years of age; disease duration ≥6 months) | Persistent: n=151 (59.7), adult-onset: n=102 (40.3) | Clinical features, including involved body sites, morphology, past and present medical history, previous and ongoing AD treatments. | Fisher’s exact test | N |
|  | **Results of the analysis →** | Subgroup analysis between persistent vs adult-onset AD patients showed significant results only regarding AD severity (severe disease was more common in persistent group, p<0.05, 8/102, 7.8 vs 28/151, 18.5%), itch intensity (higher in adult-onset disease, 6.2 ± 2.3 vs 4.6 ± 2.5; p<0.05), and comorbidities (hypertension was more frequent in adult-onset group, 4/151, 2.6 vs 14/102, 13.7%; p<0.01). Significant higher subjects mean age was registered in AD adult-onset group respect to persistent AD one (36.5 ± 12.4 years vs 31.9 ± 11.6, p<0.05). No other significant differences were found between the groups.  As regards morphology of AD lesions, no differences were found: erythemato-desquamative pattern was the most common clinical presentation in both groups (118/151, 78.1% in persistent AD group and 70/102, 68.6% in adult-onset AD), followed by lichenified pattern (21/151, 13.9 vs 20/102, 19.6%). Exudative pattern was registered as the less frequently observed being reported in only 3.3% (5/152) and 3.9% (4/102) of persistent and adult-onset disease group, respectively. There were not any significant differences between persistent vs adult-onset AD except for narrow band (NB)-UVB which was more common in persistent AD past medical history (24/151, 15.9 vs 6/102, 5.9%, p<0.05). No statistically significant differences were found regarding AD lesion localization between persistent and adult-onset AD group. | | | | | | | | | | | |
| Mohrenschlager 2006 | Cross-sectional | 1994-2000 | Pop | Germany | European Region | 580 | (5-7) | 265 (45) M / 318 (55) F | Early onset eczema (< 2 years of age), late-onset eczema (≥ 2 years of age) in boys and girls | Early-onset: n=301 (52)  Late-onset: n=279 (48) | Atopy (by skin prick test and serum IgE measurements) at age 5-7 years | Breslow–Day test was used, logistic regression | N |
|  | **Results of the analysis →** | Early-onset eczema was reported for 5.8% of the girls and 5.2% of the boys without providing any significant statistical difference. The association between early-onset eczema and atopy at the age of 5–7 years was even stronger for girls than for boys and the association was stronger than the association between current eczema and atopy. Persistent eczema showed the highest OR for the associations with atopy, with no gender differences (OR 3.6 (95% CI 2.5–5.1)). In girls, early onset eczema (< 2 years of age) was strongly related to atopy at age 5–7 years [odds ratio (OR) 3.7; 95% confidence interval (CI) 2.7–5.1], whereas late-onset eczema (≥ 2 years of age) was not (OR 1.0; 95% CI 0.7–1.5). Boys were more often atopic at the age of 5–7 years than girls (28.3% vs. 20.6%), and early and late-onset eczema were related to atopy without such a difference (OR 2.8, 95% CI 2.0–4.0; OR 1.9, 95% CI 1.3–2.8, respectively). Late-onset eczema was not associated with atopy in girls. It was also not associated when defining atopy by skin prick test with inclusion of sensitization by egg and milk, which were not available for the serum measurements. Then the OR for the association between atopy (prick test) and late-onset eczema was 0.7 (95% CI 0.4–1.1) for the girls and 1.4 (95% CI 0.9–2.1) for the boys. | | | | | | | | | | | |
| Nettis (also in morphology characteristics group) 2020 | Cross-sectional | 2018-2020 | Hos | Italy | European Region | 550 | Median: 38.0 (IQR: 27.0) | 308 (56.0) M / 242 (44.0) F | Childhood-onset AD, adult-onset AD | Childhood-onset AD: n=348 (63.3), adult-onset AD: n= 202 (36.7) | Clinical characteristics: lichenified/ exudative flexural dermatitis alone and associated with portrait dermatitis, nummular eczema–like phenotype, PN-like pattern | Fisher exact test | N |
|  | **Results of the analysis →** | Lichenified/ exudative flexural dermatitis alone and associated with portrait dermatitis was more common in childhood-onset AD than in adult-onset AD (191/348 [54.9%] vs 76/202 [37.6%], P<0.01). Nummular eczema–like phenotype and PN-like pattern were associated with adult-onset, when compared to childhood-onset AD (15/202 [7.4%] vs 6/348 [1.7%], P<0.01 respectively). No statistically significant differences were found regarding the other phenotypes between childhood-onset AD and adult-onset AD. | | | | | | | | | | | |
| Nowrouzian 2019 | Cross-sectional | NR | Pop | Sweden | European Region | 12 | (0-36 mo) | NR | Early-onset eczema, by 18 months of age | 100% | Patterns and strains of nasal and gut colonisation of S. aureus | Fisher’s exact test | Y (52) |
|  | **Results of the analysis →** | Nasal and gut colonisation patterns: There were no differences in faecal counts between healthy and AD individuals. Nasal colonization by S. aureus during the first 2 months was slightly more common in the infants who remained healthy than in those who subsequently developed atopic eczema (not statistically significant). Gene carriage by gut commensal S. aureus: Strains from the infants who subsequently developed atopic eczema less frequently carried the ebp gene, encoding elastinbinding protein (71% vs. 22%, P = 0.01; OR developing eczema 0.2(95% CI 0.02 – 0.64)), and superantigen genes encoded by egc, compared with strains from children who remained healthy (71% vs. 11%; P = 0.001; OR of 0.05 (95% CI 0.006– 0.45). Results remained significant after exclusion of allergic infants, except for the negative association between ebp and atopic eczema. Gene carriage by nasal commensal S. aureus: egc superantigen gene with SEI encoding was significantly more commonly found among strains from subsequently healthy infants. The fib gene, encoding the adhesin fibrinogen-binding protein, was significantly more common in strains from subsequently eczematous infants. These associations were no longer significant after excluding children with other allergic manifestations. | | | | | | | | | | | |
| Pavel (also in severity group) 2021 | Cross-sectional | NR | Hos | U.S.A. | Region of the Americas | 19 | 1.7 (0-5) | 14 (73.7) M / 5 (26.3) F | Early-onset (starting within the previous 6 months, age 0-5 years) moderate-to-severe (not further specified) AD | 100% | mRNA profile in lesional and nonlesional tape-stripped skin including differentially expressed genes (DEGs) and epidermal barrier alterations | Fold-changes, hypothesis testing, Spearman correlation coefficients | Y (17) |
|  | **Results of the analysis →** | 1829 DEGs were identified in lesional AD and 662 DEGs in nonlesional AD, compared to healthy skin (fold-change ≥2, FDR < −0.4, P < .05). Significant correlations were also identified between clinical measures (body surface area/BSA, pruritus ADQ, and transepidermal water loss/TEWL) with immune and barrier mRNAs in lesional and/or nonlesional AD (FLG/FLG2 with TEWL; r < −0.4, P < .05). | | | | | | | | | | | |
| Paternoster 2018 | Cohort | 1991-2007 | Pop | U.K., the Netherlands | European Region | Differs over time (13.546 participants in total) | (1-16.5) | NR (6.564 (48) F, 6.982 (52) M in total) | Six latent classes identified based on course of AD over time: early-onset-early resolving, early-onset-persistent, early-onset-late-resolving, mid-onset-resolving, late-onset-resolving, unaffected/transient | Early-onset-early resolving: 12.9% in ALSPAC, 15.4% in PIAMA, early-onset-persistent: 7.3% in ALSPAC, 4.9% in PIAMA, early-onset-late-resolving: 7.0% in ALSPAC, 3.8% in PIAMA, mid-onset-resolving: 7.0% in ALSPAC, 6.5% in PIAMA, late-onset-resolving: 7.9% in ALSPAC, 6.5% in PIAMA, unaffected/transient: 58.0% in ALSPAC, 62.9% in PIAMA | Known AD risk factors, including FLG null mutations , 23 other established AD-genetic risk variants, (parental) atopic comorbidity (serum IgE levels (at ages 7, 8), comorbid Asthma (at ages 7, 11, 13)), sex, breastfeeding | Data-driven approach to identify phenotypes, by using latent class analysis  Multinomial regression, logistic regression | Y (58%/62.9%) |
|  | **Results of the analysis →** | Being female was a risk factor for the early-onset-persistent, mid-onset, and late-onset classes, the strongest association being with the late-onset class (ALPAC: odds ratio [OR], 1.90; 95% CI, 1.48-2.44; P = 4x 10^-7; PIAMA: OR, 1.87; 95% CI, 1.21- 2.90; P = .005). However, male sex was a risk factor for the early-onset-early-resolving class (ALSPAC: OR, 1.33; 95% CI, 1.10-1.61; P = .004). Maternal history of AD was associated with all classes in ALSPAC, with the strongest association in the persistent class (OR, 3.16; 95%CI, 2.60-3.83; P= 4x 10^-31). A similar pattern maternal history of asthma, where again the strongest association was with the persistent class (OR, 1.54; 95% CI, 1.22-1.95; P = 3 x 10^-4). Paternal asthma also showed association with the early-onset-late-resolving class (OR, 2.53; 95% CI, 1.30-4.91; P = .006). In PIAMA the associations with maternal and paternal history of asthma were similar. In ALSPAC, breast-feeding was associated with a higher risk of persistent and early-onset-late-resolving AD (OR, 1.42, 95% CI, 1.11-1.81, P =.006, and OR, 1.53, 95% CI, 1.12-2.08, P =.008, respectively). There was little evidence of association with mid- or late-onset classes. In PIAMA, there was little evidence for breast-feeding being associated with any class. Early-life exposure to a pet cat was not associated with any of the latent classes in the primary analyses for ALSPAC or PIAMA. In ALSPAC, all classes showed association with asthma at age 7 and 13 years. The associations were strongest for the persistent class (7 years: OR, 5.50, P = 5 x 10^-41; 13 years: OR, 7.19; P = 3 x 10^-46). In PIAMA, the persistent and early-onset-late-resolving group showed association with asthma at age 7 years (persistent OR, 14.27; P = 5 x 10^-15). At age 11 years, all but the mid-onset-resolving group were associated, again the strongest association being with the persistent group (OR, 15.35; P = 3 x 10^-11). In ALSPAC, only the mid-onset-resolving AD was not associated with FLG mutations. The strongest association was for the persistent group (OR, 4.31; 95% CI, 3.29-5.63; P = 2 x 10^-26); the other associated classes had ORs of about half this (2.14-2.30). In PIAMA, only the early-onset-late-resolving class was associated with FLG null mutations (OR, 5.63; 95% CI, 2.65-11.95; P = 7 x 10^-6). The combined genetic risk score encompassing all other AD variants was associated with all but the early-onset-early- resolving and the late-onset classes in the 6-class model in ALSPAC. The association was strongest with the persistent class (OR, 1.17; 95% CI, 1.12-1.22, for each additional risk allele; P = 2 x 10^-13). A similar pattern was observed in PIAMA, with the persistent class showing the strongest association and an almost identical effect size to that seen in ALSPAC (OR, 1.17; 95% CI, 1.07-1.28; P = 5 x 10^-4). | | | | | | | | | | | |
| Quah (also in disease severity category) 2015 | Cohort | 2004-2011 | Hos | Singapore | Western Pacific Region | 65 | (0-5) | NR | Early onset of eczema by 2 years + Severity of eczema up to 2 years: SCORAD quartiles: <22.56, 17.11-22.55, 11.075-17.10, <11.075 | Early onset of eczema by 2 years: n=57 (88)  SCORAD quartiles: <22.56: n=13, 17.11-22.55: n=14, 11.075-17.10: n=13, <11.075: n=13  (severity missing n=4) | Atopic disorders (manifestations of eczema, wheeze and rhinitis) and allergen sensitization at the age of 5 years | Chi-squared analysis, logistic regression analyses | Y (174) |
|  | **Results of the analysis →** | Eczema at year 2 increased the risk of eczema at year 5 (adjOR = 7.1; 95 % CI: 1.8–27.8) and this was further increased by the presence of allergen sensitization (adjOR = 25.4; 95 % CI: 4.7–138.5) and the concomitant presence of both wheeze and allergen sensitization (adjOR = 64.9; 95 % CI: 4.7–900.0). More severe eczema at the age of 2 years (SCORAD above median) was more strongly associated with eczema at year 5 than those with less severe eczema (SCORAD below median) (SCORAD above median: adjOR =15.2; 95 % CI = 4.3–53.7; SCORAD below median: adjOR = 5.7; 95 % CI = 1.4–22.7).  No significant association between eczema severity in the first 2 years of life and allergen sensitization at 2 years. Early onset of eczema at 2 years increased the risk of rhinitis (adjOR = 6.8; 95 % CI: 2.0–23.1). For HDM allergen sensitization at 5 years of age, early onset of eczema increased the risk (adjOR = 3.6; 95 % CI = 1.5–8.7). More severe eczema was significantly associated with the development of allergen sensitization at 5 years (SCORAD above median: adjOR = 2.9; 95 % CI = 1.0–8.5). Eczema severity at the age of 2 years did not affect outcomes for wheeze and rhinitis at 5 years (p > 0.05). | | | | | | | | | | | |
| Roduit 2012 | Cohort | 2002-2005 | Pop | Austria, Finland, France, Germany, Switzerland | European Region | 241 | (0-4) | NR | AD with onset within the first year of life, and after the first year of life | AD with onset within the first year of life: n=144 (60),  AD with onset after the first year of life: n=97 (40) | Early postnatal exposures to farm animals, feeding practices in the first year of life | Chi-squared test, generalized estimating equations, logistic regression | Y (665) |
|  | **Results of the analysis →** | No association between early postnatal contact with farm animals and AD with onset after the first year of life. Negative association between prenatal contact with farm animals and AD with onset during the first year of life (prenatal and postnatal OR 0.51[95% CI 0.26-1.00]; only prenatal OR 0.61 [95% CI 0.35-1.08]). Introduction of yogurt and shop milk within the first year of life showed an inverse association with the development of AD with onset after the first year of life compared with no introduction, indicating a protective effect (adjusted OR, 0.41; 95% CI, 0.23-0.73 and adjusted OR, 0.52; 95% CI, 0.30-0.92, respectively). The consumption of farm milk in the first year of life had a tendency to decrease the risk of having AD but only among children with no allergic parents (adjusted OR, 0.49; 95% CI, 0.21-1.18). The diversity of introduction of complementary food in the first year of life was associated with a reduction in the risk of having AD with onset after the first year of life (adjusted OR for AD with each additional major food item introduced, 0.76; 95% CI, 0.65-0.88). | | | | | | | | | | | |
| Roduit 2017 | Cohort | 2002-2005 | Pop | Austria, Finland, France, Germany, Switzerland | European Region | 213 | (0-6) | NR | Using latent class analysis, 4 phenotypes of AD  were identified depending on the onset and course of the disease: 2 early phenotypes with onset before age 2 years: early transient (no further symptoms after age 4 years), early persistent (persistence of symptoms until age 6 years); the late phenotype with onset at age 2 years or older; (the never/infrequent phenotype (no AD)) | Early transient:  n=96 (9.2), early persistent: n=67 (6.5), late phenotype: n = 50 (4.8), (never/infrequent phenotype: n=825 (79.5)) | Other allergic diseases (including food allergy, asthma, allergic rhinitis) | Data-driven approach to identify phenotypes, by using latent class analysis model  Multivariable logistic regressions | Y (825) |
|  | **Results of the analysis →** | Children with both parents with history of allergies were 5.35 (adjusted OR, 95% CI: 2.52-11.36) times more at risk to develop early-persistent AD compared with children with parents with no history of allergies. Both early phenotypes were strongly associated with food allergy (adjusted odds ratio, 3.69; 95% CI, 1.93-7.04 for early transient and adjusted odds ratio, 7.08; 95% CI, 3.59-13.98 for early persistent). Risk of developing asthma was significantly increased among the early-persistent phenotype (adjusted odds ratio, 2.87; 95% CI, 1.31-6.32). Late phenotype was only positively associated with allergic rhinitis (adjusted odds ratio, 3.23; 95% CI, 1.37-7.62). Early-persistent phenotype also had an increased risk of developing allergic rhinitis (adjusted odds ratio, 4.04; 95% CI, 1.82-8.96). Sensitization measured at 6 years showed positive associations between early-persistent AD and sensitization to inhalant allergens (adjusted OR 3.36 (1.78-6.355)) and between the late AD and sensitization to food allergen (adjusted OR 2.06 (1.05-4.035). | | | | | | | | | | | |
| Semic-Jusufagic 2010 | Cohort | NR | Pop | U.K. | European Region | 262 | (0-8) | NR | Transient eczema (eczema during the first 5 yr of life, no eczema after age 5), persistent (eczema at all time points (3, 5 and 8 yr)), intermittent (eczema at one time point during the first 5 yr, eczema at age 8) and late-onset (no eczema during the first 5 yr, eczema at age 8) | Transient eczema: n=91 (35), intermittent eczema: n=71 (27), persistent eczema: n=81 (31), late-onset eczema: n=19 (73) | Serum-soluble interleukin-5 receptor alpha levels (s-IL-5Ra) | Independent t test, multiple anova model | Y (203) |
|  | **Results of the analysis →** | No association between s-IL-5Ra and current eczema at age 5 or eczema ever in the first 5 yr of life, there was a significant association between eczema phenotypes and s-IL-5Ra (multiple anova model adjusted for gender and atopy, F = 2.56, p = 0.04), with children with late-onset eczema having significantly higher s-IL-5Ra levels compared to other groups. After adjustment for multiple comparisons, we found that children with late-onset eczema had significantly higher s-IL-5Ra compared to those who have never had eczema (mean difference [95% CI], 2.41 [1.03–5.62], p = 0.04) and those with intermittent eczema (2.63 [1.08–6.41], p = 0.02), with no difference between children who have never had eczema and other eczema phenotypes. | | | | | | | | | | | |
| Seo 2018 | Cross-sectional | 2012-2015 | Hos | Korea | Western Pacific Region | 572 | 13.29 mo ± 8.71 mo | 341 (59.6) M / 231 (40.4) F | Clinical phenotypes of AD in early childhood by cluster analysis: cluster A: early-onset AD with high eosinophil and food sensitization, cluster B: early-onset, non-allergic AD, cluster C: early-onset AD with high C-reactive protein, cluster D: middle-onset AD with inhalant sensitization. | Cluster A: n=141 (25), cluster B: n=218 (38), cluster C: n=53 (9.2), cluster D: n=160 (28) | SCORAD, TEWL | Data-driven approach to identify phenotypes, by using cluster analysis with a two-step approach based on 11 variables  ANOVA, chi-squared test | N |
|  | **Results of the analysis →** | Cluster A had the highest SCORAD (20 ± 10) and lesional TEWL values (59.2 ± 28.3), compared to cluster B (13 ± 7, 46.9 ± 23.0), cluster C (17 ± 9, 46.7 ± 19.8) and cluster D (15 ± 8.5, 47.7 ± 23.3) (p<0.001 and p=0.003 for the objective SCORAD and lesional TEWL respectively). Age at onset, age at diagnosis, white blood cell count, eosinophil count, C-reactive protein and serum total IgE level were the strongest predictors of cluster assignment (Wilks’s λ = 0.177, P < 0.001). These six variables resulted in correct classification of 95.5% of the original studied population. | | | | | | | | | | | |
| Shen 2013 | Cross-sectional | 2000-2010 | Pop | Taiwan | Western Pacific Region | 108.703 | (0-7) | NR | Persistent early eczema (age of onset before 36 months of age with a diagnose every year after 36 months), intermittent early eczema (diagnose after 36 months but not every year), transient early eczema (absent at age 3-6: only a diagnosis before 36 months of age), late-onset eczema (absent at age 0-2, present at 3-6: age of onset after 36 months of age) | Persistent early eczema: n= 7700 (2.6), intermittent early eczema: n= 11308 (3.8), transient early eczema: n=65948 (22.5), late-onset eczema: n=23747 (8.1) | Asthma and allergic rhinitis (AR) at age 7 years (85-96 months) (diagnostic codes ICD-9 493 and 477, respectively) | Chi-squared test | Y (185.019) |
|  | **Results of the analysis →** | Early eczema was associated with asthma at the age of 7 years (RR 1.63, 95% CI, 1.58-1.66). Those with persistent early eczema had a higher risk for asthma than those with transient eczema (RR 2.70, 95% CI, 2.57-2.83 and RR 1.63, 95% CI, 1.58-1.66), respectively). Late-onset eczema had a higher risk for asthma (RR 2.15, 95% CI, 2.08-2.23) than transient eczema. The RR for intermittent eczema was 2.60, 95% CI, 2.50-2.71. The RRs for AR at 7 years showed the same trends. Adjusted risk for different eczema groups showed similar results. | | | | | | | | | | | |
| Shoda 2016 | Cohort | 2003-2009 | Hos | Japan | Western Pacific Region | 371 | (0-3) | 200 (53.9) M / 171 (46.1) F | Five subgroups based on the timing of eczema onset: 1) less than 1 mo, 2) 1–2 mo, 3) 3– 4 mo, 4) 5–8 mo and 5) 9–12 mo. | < 1 mo: n=31 (3.4);  1-2 mo: n=64 (7.1); 3-4 mo: n=60 (6.6), 5-8 mo: n=55 (6.1); 9-12 mo: n=38 (4.2) | Food allergy at 3 years of age | Logistic regression analyses | Y (959) |
|  | **Results of the analysis →** | Eczema in the first year of life was a significant risk factor for development of food allergy at 3 years in multivariate analysis (aOR 3.90, 95% CI 2.34–6.52, p < 0.001). In each age (by month) subgroup the risk tented to be different. Infants with onset of eczema within the first 1–2 months after birth had the highest risk of food allergy at 3 years of age (aOR 6.61, 95% CI 3.27–13.34, p < 0.001), followed by onset of eczema between 3-4 months (aOR 4.69, 95% CI 2.17–10.13, p < 0.001). | | | | | | | | | | | |
| Silverberg (also in morphology characteristics category) 2018 | Cross-sectional | 2014-2016 | Hos | U.S.A. | Region of the Americas | 356 | 42.8 ± 16.7 (18-93) | 126 (35.4) M / 230 (64.6) F | Adult-onset (≥ 18 years) vs childhood-onset adults with AD | Adult-onset: n=149 (41.9), Childhood-onset: n=207 (58.1) | Surveys included questions about sociodemographics, birthplace, age of moving to the United States for foreign-born Americans. Medical history and skin examination by dermatologist (including Hanifin and Rajka major and minor criteria, EASI, SCORAD). | Chi-squared test, Fisher exact tests, Mann-Whitney U test, latent class analysis, multivariate logistic regression | N |
|  | **Results of the analysis →** | Adult- versus childhood-onset AD was associated with birthplace outside the United States (22.5% vs 11.5%; X2, P = .0008), but not sex, race/ethnicity, current smoking status, or alcohol consumption (P ≥ .11); and decreased personal history of asthma, hay fever, and food allergy and family history of AD, asthma and food allergy (P ≤ .0001 for all). There was no significant difference in the EASI, SCORAD, body surface area, numeric rating scale for itch and sleeplessness, or Patient-Oriented Eczema Measure between adult- and childhood-onset AD (Mann-Whitney U test, P ≥ .10). Adult-onset AD compared with childhood-onset AD was associated with significantly higher rates of nummular eczema lesions (p= .0097), but lower rates of 11 of 18 signs and symptoms of AD, including dermatitis affecting anterior neck fold, scalp, face, eyelids and conjunctivitis, Dennie-Morgan folds, hands or feet, nipples, cheilitis, pityriasis alba, KP/PH/ichthyosis, clinical course worsened by emotional or environmental factors, pruritus when sweating, and tendency toward cutaneous infections (p<.005). Furthermore, patients with adult- versus childhood-onset AD had a significantly lower number of combined AD signs and symptoms (median [IQR], 6 [2-8] vs 10 [6-13]; P< .0001). Latent class analysis identified 3 classes: (1) high probability of flexural dermatitis and xerosis with intermediate to high probabilities of head, neck, and hand dermatitis; (2) high probability of flexural dermatitis and xerosis, but low probabilities of head, neck, and hand dermatitis; and (3) lower probability of flexural dermatitis, but the highest probabilities of virtually all other signs and symptoms. Adult-onset AD was significantly associated with class 1 (multivariate logistic regression; adjusted odds ratio, 5.54; 95% CI, 1.59-19.28) and class 3 (adjusted odds ratio, 14.03; 95% CI, 2.33-85.50). | | | | | | | | | | | |
| Soomro 2018 | Cohort | NR | Hos | French | European Region | 184 | (0-5) | 184 (100) M / 0 (0) F | Early-onset (0–24 mo of age) eczema and late-onset (24–60 mo of age) eczema in male children | Early-onset: n=95 (52), late-onset: n=89 (48) | Early-life exposure to phthalates measured by maternal urinary concentrations of phthalate metabolites between the 24th and 28th week of gestation | Adjusted multiple logistic regression, Cox’s survival model | Y (420) |
|  | **Results of the analysis →** | Metabolites of di-isobutylphthalate(DiBP) and di-isononylphthalate(DiNP) were positively associated with early-onset (0–24 mo of age) eczema (15.7%) and late-onset (24–60 mo of age) eczema (14.7%). MiBP and MCOP were positively associated with early-onset eczema [OR=1:27 (95%CI: 1.00, 1.72), p<0:05 and OR=1:29 (95%CI:1.04,1.60), p<0:05, respectively] as well as late-onset eczema [OR=1:55 (95% CI:1.10,2.18), p<0:05 and OR=1:63 (95%CI:1.20,2.21), p<0:05, respectively] after adjusting for potential confounders. Other phthalate metabolites were not significantly associated with early-onset eczema. However in the case of late-onset eczema, MECPP and MCNP were positively associated [OR=1:34 (95%CI:1.00,1.81), p<0:05 andOR=1:29 (95% CI:1.02,1.64), p<0:05, respectively],whereas the association with MEHHP, MEOHP, and RDEHP were of borderline significance (p<0.10). | | | | | | | | | | | |
| Steiman 2020 | Cohort | 2013-2018 | Pop | U.S.A. | Region of the Americas | NR | (6-48 mo) | NR | Early-onset of AD before 2 years old | 100% | Farm exposures prenatally and in early life | Data-driven approach to identify phenotypes, by using latent class analysis | Y (NR) |
|  | **Results of the analysis →** | Among the latent class groups, children in families with diverse or more intense farm exposures (classes A and B) had reduced AD incidence, whereas low-exposure (class C) infants had AD incidence similar to that in nonfarm children. Among children in the farm group, cumulative prevalence of AD was positively related to delivery mode (vaginal delivery 16%, C-section 41%, P = .01) and inversely related to exclusive breast-feeding (12% vs 28%, P =.05). Children of farm families had reduced AD incidence (P = .03). Within farm families, exposures including poultry (3% vs 28%; P = .003), pig (4% vs 25%; P = .04), feed grain (13% vs 34%; P = .02), and number of animal species were inversely associated with AD incidence. | | | | | | | | | | | |
| Von Kobyletzki (also in morphology characteristics group) 2014 | Cohort | 2000, 2005 | Pop | Sweden | European Region | 829 | (1-8) | 419 (50.5) M / 410(49.5) F | AD with onset in or before the third year of life with remission in childhood (within 5 years of baseline/by age 6-8) | N=484 (52) reported no eczema during the 12 months preceding their follow-up interview in 2005;  n=345 participants without remission | Background, health, lifestyle, and environmental variables including: parental history, problems paying bills, house location, flexural eczema, awake at night, food allergy and rhinitis | Multivarablelogistic model | N |
|  | **Results of the analysis →** | Independent factors at baseline predicting remission were: milder eczema (adjusted OR (aOR), 1.43; 95% 1.16–1.77); later onset of eczema (aOR 1.40; 95% CI 1.08–1.80); non-flexural eczema (aOR 2.57; 95% CI 1.62–4.09); no food allergy (aOR 1.51; 95% CI 1.11–2.04), and rural living (aOR 1.48; 95% CI 1.07–2.05). Other factors were also associated with remission, such as having no or only one parent with a history of allergic disease. Factors related to the birth environment (birth order, sex, problems paying bills, or parental smoking) or family lifestyle (bedrooms with PVC flooring material, home construction, breastfeeding, antibiotic consumption and kindergarten attendance) were not associated with remission and neither was birth-weight. | | | | | | | | | | | |
| Wan 2017 | Cohort | 2004-2017 | NR | U.S.A. | Region of the Americas | 3966 | Median: 6.4, IQR: 4.0–10.2 (2-17) | (47) M / (53) F | Early (≤ 2 years), mid (3-7) and late onset (8–17 years) pediatric AD | Early: n=2913 (73), mid: n=725 (18), late: n=328 (8.2) | Seasonal allergies and asthma | Chi-squared test, Kruskal-Wallis test, logistic regression models | N |
|  | **Results of the analysis →** | At baseline, subjects with AD onset at ages 3–7 or 8–17 had significantly lower rates of seasonal allergies (74.6%, 69.9%, and 70.1% in the early-, mid-, and late-onset groups, respectively) and asthma (51.5%, 44.7% and 43.0% in the early-, mid-, and late-onset groups, respectively) than those with onset before age 2. After multivariable adjustment for sex, race, and age at registry enrollment, the relative risks for prevalent seasonal allergies were 0.91 (95% CI 0.88–0.95) and 0.82 (95% CI 0.74–0.89) in the mid- and late-onset groups, respectively. The risk of incident (i.e. new onset after enrollment) seasonal allergies significantly decreased with increasing age category of AD onset, with adjusted relative risks of 0.82 (95% CI 0.72–0.91) and 0.64 (95% CI 0.47–0.83) among the mid- and late-onset groups, respectively, compared to the early-onset group. The adjusted relative risk of prevalent asthma was significantly lower among the mid- and late-onset groups (RR 0.85, 95% CI 0.80–0.91 and RR 0.71, 95% CI 0.61–0.82, respectively) compared to the early-onset group. The adjusted risk of incident asthma was not significantly different between the older onset groups and the earliest onset group. After adjusting for participant sex, race, age at enrollment, and duration of follow-up, the risks of incident asthma in the older onset age groups were not significantly different from the early-onset group. | | | | | | | | | | | |
| Wan 2019 | Cohort | 2004-2018 | Hos | U.S.A. | Region of the Americas | 8,015 | Upon enrollment: median: 6.6 (IQR: 3.9–10.4) | 4,273 (53.3) F | Onset age was categorized into ≤2, 3–7, and 8–17 years old, i.e. early-onset, mid-onset, and late-onset AD, respectively | Early-onset: 5770 (72.0), mid-onset: 1492 (18.6), late-onset: 712 (8.9) | AD disease control and persistence | Generalized linear latent and mixed models | N |
|  | **Results of the analysis →** | The aORs for worse control were 0.71 (95% CI 0.64–0.80) and 0.51 (95% CI 0.43–0.60) in the mid- and late-onset groups, respectively, compared to the early-onset group. While all groups increasingly reported complete control with increasing age, the late-onset group was more likely to report complete control than the mid-onset and early-onset groups across all ages, and differences among the three groups were most distinct in the second and third decades of life. The odds of persistent AD were also lower for mid-onset (aOR 0.45 [95% CI 0.34–0.60]) and late-onset (aOR 0.19 [95% CI 0.12–0.30]) AD relative to early-onset AD. In all three groups, the proportion of subjects reporting persistent AD generally declined with older age, and differences among the three onset groups were most pronounced from early adolescence onward. | | | | | | | | | | | |
| Wang 2008 | Case-control | NR | NR | Sweden, U.K., Italy | European Region | 15 | (0-18 mo) | NR | Development of atopic eczema in the first 18 months of life | 100% | Infantile fecal microbiota (at 1 week of age) | Mann-Whitney rank sum tests, logistic regression modeling | Y (20) |
|  | **Results of the analysis →** | The median number of peaks, Shannon-Wiener index, and Simpson index of diversity were significantly less for infants with atopic eczema than for infants remaining healthy (p=0.01-0.05). The same was found when TTGE patterns were compared. The direction of the associations was unaltered by using logistic regression with adjustment for sex and study center, but the significance of the findings was generally enhanced. | | | | | | | | | | | |
| Wang 2020 | Cohort | 2015-2016 | Hos | Taiwan | Western Pacific Region | 50 | Upon enrollment: 2-4 mo | 25 (50) M / 25 (50) F | Exclusively breastfed infants with infantile AD status divided into 3 groups: no AD (never had AD), early-onset-early-resolving AD (infantile AD subsided in the first 2 years of life) and early-onset-persistent AD (infantile AD persisted after 2 years old) | Early-onset-early-resolving AD: n=26 (52), early-onset-persistent AD: n=24 (48) | Allergic disease: allergic rhinitis, asthma at age 3 | Logistic regression | Y (48) |
|  | **Results of the analysis →** | Patients with early-onset-early-resolving AD did not have an increased risk of allergic rhinitis and asthma development, and sensitization to allergens. However, the early-onset-persistent AD increased the risk of allergic rhinitis development and sensitization to inhalant allergens (adjusted OR 2.83, 7.07, respectively). | | | | | | | | | | | |
| West 2015 | Case-control | 2002-2004 | Hos | Australia | Western Pacific Region | 10 | (0-2.5) | 3 (30) M / 7 (70) F | Onset of (IgE-associated) eczema over the first 2.5 years in predisposed children due to maternal atopy | 100% | Microbial composition and diversity of the gut microbiome in stool samples | Nonparametric techniques  including Wilcoxon’s rank sum test, principal component analysis | Y (10) |
|  | **Results of the analysis →** | The relative abundance of Gram-positive Ruminococcaceae was lower at 1 week of age in infants developing IgE-associated eczema, compared with controls (P = 0.0047). At 1 year, alpha-diversity of Actinobacteria was lower in infants with IgE-associated eczema compared with controls (P = 0.002). The overall alpha-diversity was lower at 1 week of age in infants developing IgE-associated eczema, but this difference did not reach statistical significance. No significant differences were found for the alpha-diversity of Bacteroidetes, Proteobacteria and Firmicutes and for all for the time points 1 week and 1 month | | | | | | | | | | | |
| Yamamoto-Hanada 2019 | Cohort | 2003-2005 | Hos | Japan | Western Pacific Region | NR | (0.5-9) | (50.6) M / (49.4) F | 4 AD trajectory phenotypes: never/infrequent, early-onset, late-onset, persistent | Never/infrequent: (62.7),  early-onset (17.6), late-onset (9.5),  persistent (10.1) | Parental history of AD and asthma, sensitization to common allergens, persistent AD (at 9 years of age) | Data-driven approach to identify phenotypes, by using the group-based trajectory modeling approach | Y (758) |
|  | **Results of the analysis →** | Children with persistent AD had a higher proportion of parental history of AD (40.2% vs. 14.4%), higher sensitization to common allergens at age 9 years (96.1%vs. 67.5%), and AD more frequently diagnosed by physicians based on the UK Working Party’s criteria at 9 years old (91.0% vs. 9.5%) than children with never/infrequent AD. In particular, persistent AD was more highly associated with a family history of AD and asthma and a higher degree of sensitization. | | | | | | | | | | | |
| Yang 2020 | Cohort | 2007-2018 | Pop, Hos | Korea | Western Pacific Region | 355 | (0-3) | 610 (51.7) M / 570 (48.3) F | Early-onset transient, with onset within 2 years of age and no further symptoms after 3 years of age; early-onset persistent, with onset within 2 years of age and persistence of symptoms after 3 years of age; late onset, with onset of symptoms after 2 years of age | Prevalence of early-onset persistent: (12.0), early-onset transient: (21.6), late-onset: (3.0) | (Air pollutant) exposure to particulate matter with an aero-dynamic diameter ranging from 0.1mm to 2.5mm (PM2.5) during gestation, the influence of cord blood vitamin D on PM2.5 induced AD | Logistic regression analysis, Bayesian distributed lag interaction model | Y (616) |
|  | **Results of the analysis →** | PM2.5 exposure during the first trimester of pregnancy, especially during 6 to 7 weeks of gestation, was associated with early-onset persistent AD. This effect increased in children with low cord blood vitamin D, especially in those with PM2.5 exposure during 3 to 7 weeks of gestation. There were no significant associations between PM2.5 exposure during any trimester of pregnancy and other AD phenotypes. | | | | | | | | | | | |
| Yap 2014 | Cohort | 2004-2006 | Hos | Singapore | Western Pacific Region | Eczema: by 2 y: n=28; at 5 y: n=15 (atopic eczema: by 2 y: n=13; at 5 y: n=12) | 0-5 | Eczema: by 2 y: 14 (50) M / 14 (50) F; at 5 y:  6 (40) M / 9 (60) F (atopic eczema: NR) | Children who developed (atopic eczema) in the first 2 years and 5 years | Subjects who developed eczema in the first 2 years (n=28), and those with eczema at 5 years of age (n=15 [persistent from 2 years: n=11; new cases: n=4]) | Fecal microbiota composition | Linear mixed model | Y (51) |
|  | **Results of the analysis →** | Longitudinal analysis of fecal microbiota composition at three days, one and three months and one year of life revealed higher abundance of Enterobacteriaceae in patients with eczema by 2 years of age (coefficient (B): 1.081, 95% CI: 0.229- 1.933, adj p = 0.014) and specifically atopic eczema by 2 years of age (coefficient (B): 0.949, 95% CI: 0.214-1.683, adj p = 0.013) compared to controls, and Clostridium perfringens (coefficient (B): 0.521, 95% CI: 0.556-0.988, adj p=0.03) in eczema by 2 years of age compared to healthy controls. For atopic eczema at 5 years old, a lower relative abundance of Bifidobacterium was found in cases compared to healthy controls (coefficient (B): −27.635, 95% CI: −50.040 - -5.231, adj p = 0.018). | | | | | | | | | | | |
| Yazganoglu (also in morphology characteristics category) 2011 | Cross-sectional | 1996-2004 | Hos | Turkey | European Region | 321 | Median: 7; IQR: 12 (6 mo -21 y) | 175 (55) M / 146 (45) F | Turkish AD patients with onset before the age of 18 year (at infancy (1 month-2 years), at childhood (2-10 years), and at adolescence (10-18 years)) | 100% | Morphology: typical lichenified/exudative eczematous pattern or nummular, papular, prurigo-like, follicular, seborrheic dermatitis-like, mixed patterns, and erythroderma. Localization: Face/symmetrical cheek involvement, flexural sites (antecubital/popliteal/neck/wrist/ankle) with or without involvement of other parts, nonflexural involvement of the extremities, seborrheic areas (scalp, retroauricular region), anogenital/diaper area, and generalized involvement. Site of onset. | No formal statistical tests | N |
|  | **Results of the analysis →** | The main involved sites were flexures in 239 patients (74.5%), being most frequently antecubital/popliteal flexural areas in 217 patients (67.6%). Face n=189 (58.9%). Extremity n=174 (54.2%). Trunk n=151 (47.0%). Hand n=97 (30.2%; in 26.5% of infant, 31.6% of childhood and 41.3% of adolescent patients). Seborrheic areas (scalp/retroauricular) n=83 (25.9%). Foot n=36 (11.2%), Nipple n=17 (5.3%). Anogenital n=14 (4.4%; the majority being infants (64%)). Generalized n=17 (5.3%). Erythoderma n=2 (0.6%). 49.5% (n=159) of patients had nontypical localization of AD, the majority being infants or children who had flexural involvement rather than the typical cheek or extremity lesions. Lichenified/exudative eczematous pattern was the most frequent morphologic type (45.5%), followed by a mixed type (44.9%) comprising combinations of mainly lichenified/eczematous pattern with other patterns of dermatitis, mainly nummular pattern. A total of 175 patients (54.5%) had the nontypical morphologic variants such as nummular (21.2%), seborrheic dermatitis-like (21.2%), popular (18.7%), follicular (8.7%), and prurigo-like (4.6%) patterns mainly in combination with the lichenified/exsudative pattern. Among them, 32 patients (9.6%) had the following isolated morphologic variants: 17 patients (5.3%) had nummular pattern alone, whereas 7 patients (2.2%) had popular, 4 patients (1.2%) prurigo-like, and 3 patients (0.9%) follicular pattern alone. Regarding site of onset, flexural (mainly antecubital/popliteal) onset of AD was seen in the majority of cases (n = 130, 40.5%), followed by face (symmetrical cheek involvement) (n = 95, 29.6%), and extremity extensors (n = 33, 10.3%). | | | | | | | | | | | |
| Zhao 2012 | Cross-sectional | 2005-2006 | Hos | China | Western Pacific Region | 81 | Median: 15, IQR: 8.1–27.8 | 46 (51) M / 45 (49) F | Northeast Chinese AD patients with an onset before or after the age of 2 | Unclear: n=17-19 | Four non-synonymous polymorphisms in the coding region of SPINK5 (A1103A→G(Asn368Ser), G1156G→A(Asp386Asn), G1258G→A(Glu420Lys), G2475G→T(Glu825Asp)) | Spearman chi-squared test or Fisher’s exact test, single factor and Multiple logistic regression analysis | Y (250) |
|  | **Results of the analysis →** | No statistically significant difference was found when comparing frequencies of Wt (wild-type genotype) and variant (heterozygous, homozygous mutant of genotypes) between the groups, except for a significant difference in 2475G→T (P < 0.01) between the patients and the controls. | | | | | | | | | | | |

Articles in alphabetical order. Column methodological approach: presents the methodological approach for investigating associations, unless further specified (i.e. in case of data-driven approach to identify phenotypes). AD, atopic dermatitis; Hos, hospital-based; Pop, population-based; No., number of participants with (atopic) dermatitis; NR, not reported; SD, standard deviation; Y, yes; N, no; U, unclear. Age in years unless specified otherwise. Mo, months; y, year(s). C/A: children/adults [Brunner et al.: adults included as comparison group]. CI: confidence interval. FLG: filaggrin. IV: ichthyosis vulgaris. OR: odds ratio. PH: palmar hyperlinearity. SNPs: single nucleotide polymorphisms.
