# Supplementary material for: Classifying atopic dermatitis: a systematic review of phenotypes and associated characteristics
Source: J Eur Acad Dermatol Venereol. 2022 Feb 25;36(6):807–19. doi: 10.1111/jdv.18008 (PMC9307020; doi:10.1111/jdv.18008)
Supplement: Supplementary file 1 — Table S1. Evidence tables per predefined phenotype category. [file JDV-36-807-s005.zip › jdv18008-sup-0003-Table1c.docx]

**Supplementary Table 1c. Evidence table for phenotypes based on morphological features**

| Study | Study design | Year | Setting | Country | WHO region | No. | Age -  Mean ± SD (range) | Sex – M/F, no. (%) | Phenotype description | No. (%) per phenotype | Potential associated characteristic(s) (of a priori interest) | Methodological approach | Inclusion of controls (Y (no.)/ N) |
| --- | --- | --- | --- | --- | --- | --- | --- | --- | --- | --- | --- | --- | --- |

| Bremmer (also in morphology characteristics group) 2008 | Cross-sectional | 1978-2006 | Hos | U.S.A. | Region of the Americas | 1187 | 21.4 ± 17.9 (0-83) | NR | AD with (mild/moderate/severe) and without coexisting IV | AD+IV: n= 321 (32.0)  AD-IV: n=654 (68.0) (missing data: n=212) | Disease severity, age at onset, probability of having allergic respiratory disease (previous diagnosis or symptoms of ARD: asthma and allergic rhinoconjunctivitis), physical features (IV, HP, and KP, were graded on a 0-3 scale (0 = absent, 1 = mild, 2 = moderate, and 3 = severe).) | Chi-squared tests | N |
| --- | --- | --- | --- | --- | --- | --- | --- | --- | --- | --- | --- | --- | --- |
|  | **Results of the analysis →** | patients with IV than in those without (39.9% vs 32.9%, OR = 1.35, P = .050) and were most associated with severe IV (55.3% vs 32.9%. OR = 2.52, P = .002). Relationships between IV and asthma remained significant after adjusting for age, gender, season of examination, and AD severity (OR = 1.78, P= .019). A higher likelihood of concomitant allergic rhinoconjunctivitis symptoms was found if there was clinical evidence of IV (61.8% vs 53.2%, P = .018). No significant association with increased severity of IV. After adjustment for the severity of AD, age, gender, and season, the relationship between IV and allergic rhinoconjunctivitis remained significant (p = .002). A much stronger, significant association was seen between severe IV and allergic rhinoconjunctivitis symptoms (OR = 2.84, P =.048). Clinical IV was also associated with earlier onset of AD (71.1% vs 61.7% <2 y/o; p= .009), PH (81.3% vs 43.0%; P<.001) and KP (52.9% vs 28.4%; p<.001). | | | | | | | | | | | |
| Chen 2011 | Cross-sectional | NR | Hos | Singapore | Western Pacific Region | 448 | First study phase (adult group): NR; second study phase (children): 10 ± 5.18 (1–21) | First study phase (adult group): NR; second study phase (children): (68) M / (32) F | Singaporean Chinese childhood-adolescent AD patients with and without PH and AD patients with and without KP | Initial study phase (FLG mutation discovery cohort): 23 (adult patients); second study phase: 425 (paediatric patients). Of the paediatric AD cohort n=144 (33.9) had PH and n=30 (7.1) had KP | FLG null mutations | Fisher’s exact test,  logistic regression analysis | Y (536) |
|  | **Results of the analysis →** | The combined null FLG genotype of 17 mutations detected in cases and controls showed strong association with (AD [P = 5.3 · 10-9; OR 3.3],) PH (P = 9.0 · 10-15; OR 5.8), KP (Fisher’s exact test; P = 0.001; OR 4.7). 4.1% of patients with PH and 14.5% of patients without PH carried at least one FLG-null mutation. Association of FLG-null mutations with PH (P = 9.0 · 10-15; OR 5.8, 95% CI 3.6–9.3). FLG null mutations were also significantly associated with KP (P = 0.001; OR 4.7, 95% CI 2.1–10.7) even though this clinical marker was present in only 7.1% of patients. Predictive values of PH and KP for FLG-null mutations: The PPV of PH was 34.1% and the NPV was 85.5%; KP had comparable PPV of 31.6% and NPV of 79.3%; Indicating that patients with AD without PH and/or KP are unlikely to carry FLG-null mutations. | | | | | | | | | | | |
| Cheng 2016 | Cross-sectional | NR | Hos | China | Western Pacific Region | 2205 | 5.40 ± 7.7 | 1359 (61.6) M / 846 (38.4) F | AD with and without KP in the Chinese Han population | AD with KP: n=289 (13.6)  AD without KP: n=1835 (86.4)  (KP status recorded for n=2124) | Presence of eight SNPs rs12634229, rs6780220, rs4722404, rs7000782, rs7024096, rs7927894, rs878860, rs2164983 | PLINK 1.07 software | Y (2116) |
|  | **Results of the analysis →** | The A allele of rs6780220 was preferentially lower in patients with KP than those without KP (38.6% vs. 43.6%, P = 0.024, OR = 0.813) and controls (38.6% vs. 44.2%, p = 0.010, OR = 0.792), respectively, but these differences were not significant after the Bonferroni correction. | | | | | | | | | | | |
| Cheng 2012 | Cross-sectional | NR | Hos | China | Western Pacific Region | 100 | 2.87 ± 3.04 | 66 (66) M / 34 (34) F | AD with and without IV, AD with and without PH, AD with and without KP, AD with and without dyshidrosis, AD with and without cheilitis, AD with and without infra-auricular and retroauricular fissuring. All enrolled individuals were of Chinese Han ancestry. | AD+IV: n=13 (13), AD-IV: N=83 (83)  AD+KP: n=4 (4), AD-KP: N=96 (96)  AD+PH: n=31 (31), AD-PH: N=69 (69)  AD+ cheilitis: n=21 (21), AD- C: n=79 (79)  AD+ dyshidrosis: n =8 (8), AD-D: n=92 (92),  AD + infra/ retroauricular fissuring: n =23 (23), A-I: n=77 (77) | Three FLG null mutations: 3222del4, 3321delA and K4671X (as a compound genotype) | Pearson chi-squared test, Fisher’s exact test | Y (301) |
|  | **Results of the analysis →** | Compound genotype for common FLG mutations was associated with AD with IV: n=8(61.50%), p<0.001, OR 38.53, 95%CI 10.96–135.54; in comparison to controls: n=12 (3.99%). Compound genotypes for common FLG variants were associated with IV, KP and PH (p=0.001, p=0.002, p<0.001, respectively) in comparison with AD patients without IV, KP and PH, respectively. No differences were found for AD with and without dyshydrosis, cheilitis, and infra-auricular and retroauricular fissuring (p>0.05). | | | | | | | | | | | |
| Ezzedine (both morphology groups, disease trajectory group) 2012 | Cross-sectional | 2007-2008 | Hos | France | European Region | 110 | 36.6 ± 16.2 (18–85) | 49 (45) M / 61 (55) F | Adult caucasian AD patients with or without IV and with or without early onset (≤ 2 years vs > 2 years) | AD+IV: n=74 (67), AD-IV: n=36 (33)  Early onset: n=55 (50), late onset: n=28 (25), missing data for onset: n=23 (21) | Clinical signs of IV scored 0 (not present) to 3 (very severe) – diffuse xerosis, PH, scales on legs, scalp desquamation and KP. Global IV clinical severity score (0–15). Age of onset of AD, SCORAD, family/personal history for AD, allergic rhinitis, allergic conjunctivitis or asthma, and total IgE. FLG mutations R501X, S2282del4, S3247X and R2447X. | Univariate and sub sequent multivariate  unconditional logistic regression analysis | N |
|  | **Results of the analysis →** | Univariate analysis: family history of atopy (OR 4.57, P = 0.01), xerosis (OR 4.67, P = 0.0003), PH (OR 10.71, P < 0.0001), scale on legs (OR 11.00, P < 0.0001), age (OR 2.15, P < 0.0001) and 2282del4 FLG (OR 5.41, P = 0.0010) mutation were positively correlated with the AD + IV phenotype. Multivariate analysis: only SCORAD for AD (OR 0.94, P = 0.01) and global clinical severity scoring for AD + IV (OR 2.62, P < 0.0001) were found to be independent factors. No significant differences were found for sex, R501X mutation, presence of other atopic symptoms, scalp desquamation, KP, age of onset, total IgE and presence of specific IgE between the groups. No S3247X or R2447X mutations were detected. 2282del4 mutation was significantly associated with early-age onset (OR 4.87, P = 0.04). No significant association between R501x mutation and age of onset | | | | | | | | | | | |
| Flohr (also in severity group) 2014 | Cross-sectional | 2009-2012 | Pop | U.K. | European Region | 154 | 3 mo | NR | Exclusively breastfed 3-month–old infants with mild (SCORAD<20) or moderate-to-severe (SCORAD≥20) AD and flexural (either around the eyes, neck, antecubital, and popliteal fossae or ankles) or non-flexural distribution | Mild: 132 (85.7),  moderate-to-severe:  n=22 (14.3).  Flexural AD only: n=44 (29), non-flexural AD only:  n=36 (23), flexural and non-flexural: n=74 (48). | TEWL, skin prick testing results to six study foods (cow’s milk, egg, cod, wheat, sesame, peanut). | Mann–Whitney U-test | Y (465) |
|  | **Results of the analysis →** | Higher TEWL was associated with more severe disease (median TEWL ‘SCORAD <20’ 14.7 (IQR, 12.2–18.8) versus ‘SCORAD≥20’ 26.4, (IQR, 15.4–39.7, P=0.001). There was also a strong association between food sensitization and AD severity (adjusted OR_SCORAD_<_20_=3.91, 95% CI: 1.70–9.00, P=0.001 vs. adjusted OR_SCORAD≥20_=25.60, 95% CI: 9.03–72.57, P<0.001). An association was also found for egg sensitization (adjusted OR_SCORAD_<_20_=5.59, 95% CI: 2.00–15.61, P=0.001 vs. adjusted OR_SCORAD≥20_=41.38, 95% CI: 12.75–134.35, P<0.001), cow’s milk sensitization (adjusted OR_SCORAD_<_20_=6.04, 95% CI: 1.30–28.08, P=0.02 vs. adjusted OR_SCORAD≥20_=26.55, 95% CI: 4.84–145.61, P<0.001) and peanut sensitization (adjusted OR_SCORAD_<_20_=2.39, 95% CI: 0.43–13.36, P=0.32 vs. adjusted OR_SCORAD≥20_=13.65, 95% CI: 2.28–81.62, P=0.004) separately. Regarding food sensitization risk (skin prick test ≥1mm), no significant difference was found for AD phenotype (no AD vs. flexural vs. non-flexural vs. both flexural and non-flexural) after adjusting for FLG, sex and AD severity. | | | | | | | | | | | |
| Guglielmo (both morphology groups, disease trajectory group) 2020 | Cross-sectional | 2005-2020 | Hos | Italy | European Region | 31 | (11-62) | 16 (52) M / 15 (48) F | Adolescent-onset and adult-onset head and neck dermatitis (HND) (cut-off at 18 years) | Adolescent-onset HND: n=17 (55), adult-onset HND: n=14 (45), head and neck dermatitis: 100% | Age, sex, HND onset, AD distribution (exclusive HND vs diffuse AD including HND involvement), past medical history of AD or atopy (personal or family), IgE serum levels | Fisher exact test, t-test | N |
|  | **Results of the analysis →** | Adolescent-onset HND positively correlated with a past history of AD of the classic type and presented with exclusive head and neck involvement (100% and 59%, respectively) (P < .05). Adult-onset HND was associated with concomitant widespread atopic eczema, involving the flexural areas of the upper and lower limbs, trunk, nipples, or hands (68%) (P < .05). Increased serum IgE level (>100 IU/mL) was detected in 11/17 (65%) adolescents and 11/14 (78%) adult patients. No differences were observed between the two groups in terms of AD family history or personal atopy history, including asthma, food allergy, allergic rhinitis, or conjunctivitis. | | | | | | | | | | | |
| Heede (also in other morphology group) 2017 | Cross-sectional | 2006-2012 | Hos | Denmark | European Region | 233 | (18-83) | 50 (21) M / 182 (79) F | AD with and without hand eczema | AD + HE: n=131 (56), AD only: n=102 (44) | Gender, occupational dermatitis, facial dermatitis, age, positive patch test reactions, FLG mutation status (R501X, 2282del4, and R2447X) | Kruskal-Wallis test, chi-squared test | Y (287) |
|  | **Results of the analysis →** | Patients in the group with ‘hand eczema only’ were significantly older (p<0.001, Kruskal-Wallis test). Patients with only hand eczema had the highest prevalence of ≥1 positive patch test reaction (50.5%), followed by patients with both hand eczema and AD (40.5%) and patients with only AD (34.3%) (p=0.009, 𝜒2 test). Similar results were found for occupational dermatitis (61.0%, 38.9%, 2.0%, respectively). More facial dermatitis was found in patients with AD (64.7%) compared to patients with AD+HE (34.4%) or HE (12.2%). No statistical difference was found for gender. The prevalence of FLG mutations carriers was significantly higher in the group of patients diagnosed with both AD and hand eczema (33.1%) than in the group with AD only (18.8%) and the hand eczema group (8.9%) (p<0.001, 𝜒2 test). | | | | | | | | | | | |
| Kaga (also in severity phenotype group) 2011 | Cross-sectional | NR | Hos | Japan | Western Pacific Region | 56 | (21-56) | 31 (55) M / 25 (45) F | Adult patients with head and neck AD of different severities: mild, moderate and severe (not further defined) | Mild: n=21 (38), moderate: n=18 (32), severe: n=17 (30) | Extent and diversity of Malassezia colonisation: nine human-associated Malassezia species. | Kruskal-Wallis test | Y (32) |
|  | **Results of the analysis →** | A significant difference was found among all combination groups for the extent of total Malassezia colonisation in comparison to controls (P < 0.05). In severe AD, Malassezia colonisation was approximately two- to fivefold that in mild and moderate AD patients and healthy individuals. The two major microbiota, M. globosa and M. restricta, accounted for more than 80% of all Malassezia colonisation in AD patients of all severities, but their proportions differed with severity. In the mild and moderate patients, M. restricta was predominant over M. globosa (P < 0.05), whereas the proportions of M. globosa and M. restricta were almost identical (P > 0.05) in the severe patients. No difference in species diversity related to the severity of AD in the patients was found. The number detected was similar (3.5–4.2 species per case) among the members of all severity groups. | | | | | | | | | | | |
| Kou (also in severity phenotype category) 2014 | Cross-sectional | NR | Hos | Japan | Western Pacific Region | 257 | Median age (IQR): 33 (17) | 149 (58) M / 108 (42) F | Adult patients with mild, moderate, severe or very severe AD, according to Japanese guidelines: skin involvement with mild  eruption alone (mild); eruption with severe inflammation of  < 10% of the skin surface area (moderate); severe eruption, with 10–30% skin involvement (severe); severe eruption with > 30% skin surface area involvement (very severe). Further classification into: erythroderma type; widespread combi-nations of various types; prurigo type; limbs type; and head/face/neck/chest/back type. | Mild: n=42 (16), moderate: n=95 (37), severe: n=74 (29) and very severe: n=46 (18).  Erythroderma-type: n=44 (17), widespread-type: n=147 (57), prurigo-type: n=33 (13), limbs-type: n=11 (4) and head/face/neck/chest/back-type: n=22 (9). | Serum periostin levels | Mann–Whitney U-test, Wilcoxon t-test | Y (91) |
|  | **Results of the analysis →** | Patients with severe or very severe AD, had considerably higher levels of periostin than those with mild or moderate disease [mild: 104.0 (82.3–157.8) ng mL-1; moderate: 122.0 (90.0–179.0) ng mL-1; severe: 157.5 (120.3– 236.8) ng mL-1; very severe: 241.0 (163.8–371.3) ng mL-1; (p<0.01). No significant difference in periostin level between patients with AD with mild and moderate disease. Patients with erythroderma-type AD, followed by widespread-type AD, had significantly higher levels of periostin compared with the other groups (P < 0.01). | | | | | | | | | | | |
| Lammintausta (in morph charac, morphology phenotype en severity category) 1993 | Cohort | 1983-1989 | Hos | Finland | European Region | 1008 | (19-41) | NR | Four subgroups according to disease severity and other atopic symptoms: 1: severe AD, history of periods of hospitalization (n=241); 2: Moderate AD, five or more ambulatory visits to the department of dermatology (n=399); 3: Mild dermatitis, one to four outpatient clinic visits (n=161); 4: Patients with allergic rhinitis, allergic conjunctivitis or asthma, but no dermatitis in childhood (n=207).  Hand dermatitis | 1: n=241 (24); 2: n=399 (40); 3: n=161 (16); 4: n=207 (21). | Occupational exposures; the occurrence, extent and distribution of papules, vesicles, erythema and lichenification | Chi-squared test | Y (626) |
|  | **Results of the analysis →** | In moderate-severe AD patients, facial dermatitis was seen in 63% and hand dermatitis in 54%, flexural dermatitis in 50% and in 52% dermatitis occurred on the body. Hand dermatitis showed an evident correlation with occupational exposure. Of the patients exposed to wet work or mechanically skin—irritating factors for 2h or more daily, 90% had hand dermatitis, compared to 50% of those who had little or no corresponding exposure. Dermatitis in other locations was not associated with occupational exposure. | | | | | | | | | | | |
| Li 2013 | Cross-sectional | NR | NR | China | Western Pacific Region | 116 | NR | NR | AD-associated IV | 100% | FLG mutation frequency and FLG expression in the skin | Chi-squared test | Y (334) |
|  | **Results of the analysis →** | Eleven known FLG mutations were found in the 116 patients with AD-associated IV. The percentage of mutations in the FLG gene was 74% and 43% in patients with isolated IV and patients with AD-associated IV, respectively (OR 3.77, 95% CI 1.85–7.68, P < 0.001). The percentage of patients with isolated IV with the 3321delA mutation was significantly higher than the patients with AD-associated IV (48% vs. 20%, X2 = 14.4, P < 0.001). The compound genotypes for all FLG variants were significantly associated with IV [OR 60.21, 95% confidence interval (CI) 26.56–136.46, P < 0.001] and AD-associated IV (OR 101.22, 95% CI 9.20–36.71, P < 0.001). Immunohistochemical staining revealed that profilaggrin ⁄FLG peptides were remarkably reduced in the epidermis of all the patients. All the patients with either AD or IV showed lower FLG mRNA expression compared with the normal control. | | | | | | | | | | | |
| Li 2019 | Cross-sectional | NR | NR | China | Western Pacific Region | 487 | 7.1 ± 9.4 | 247 (51) M / 240 (49) F | Four dry skin phenotypes and a global clinical dry skin score as the sum of the scores for four dry skin phenotypes (0–4):  xerosis, ichthyosis vulgaris, palmar hyperlinearity and  keratosis pilaris | Global dry skin score ≥ 2: n=151 (81), global dry skin score < 2: n=35 (19) | Aryl hydrocarbon receptor (AHR) single-nucleotide polymorphisms (rs10249788 and rs2066853) | Chi-squared test, Fisher exact test | Y (436) |
|  | **Results of the analysis →** | In patients with AD, global clinical dry skin scores ≥ 2 were significantly associated with the rs10249788 combined (CT + TT) genotype (P = 0.02, OR = 3.06, 95% CI 1.12–8.30) and the rs2066853 combined (AG + AA) genotype (P = 0.01, OR = 2.68, 95% CI 1.24–5.78). Further stratification demonstrated that the AHR rs2066853 (AG + AA) and rs10249788 (CT + TT) genotypes could predict a higher risk of severe dry skin phenotypes in the male, early-onset (<2 years) and allergic rhinitis subgroups. Furthermore, the combined rs10249788 (CT + TT) and rs2066853 (AG + AA) genotypes led to a higher risk for severe dry skin (global clinical dry skin scores ≥ 2) in patients with AD, compared to other genotypes. There were no significant relationships between the rs10249788/rs2066853 polymorphisms and other AD-associated phenotypes (the dry skin phenotypes separately) (P > 0.05) | | | | | | | | | | | |
| Luukkonen (also in disease trajectory category) 2017 | Cross-sectional | 2000-2013 | Hos | Finland | European Region | 445 | 32.3 ± 14.9 | (37.2) M / (62.8) F | AD with and without early-onset (< 2 years), AD with and without PH, AD with and without KP | Early-onset AD: n=297 (74.8), AD with PH: n=143 (40.6), AD with KP: n=48 (14.2) | The 4 most prevalent European FLG null mutations (2282del4, R501X, R2447X, S3247X), 2 FLG mutations (S1020X, V603M)  enriched in the Finnish population, the 12-repeat allele (rs12730241) and 59 additional epidermal barrier gene variants) | Fisher’s exact test, logistic regression, linear model | Y (1710) |
|  | **Results of the analysis →** | Early-onset AD and PH showed significant associations with the combined FLG null genotype: The combined FLG null genotype was significantly associated with early-onset AD (< 2 years of age) (OR 4.15, p = 1.82 × 10–10) and PH (OR 4.67, p = 1.46 × 10–5), and suggestively associated with KP (OR 3.1, p = 0.0021). Regarding the individual mutations: Mutation 2822del4 was significantly associated with early-onset AD (OR 3.38, p = 8.38 × 10–6). R501X was associated with early-onset AD (OR 14.88, p = 0.00079), and suggestively with KP (OR 13.09, p = 0.0035). R2447X showed suggestive association with early-onset AD (OR 3.86, p = 0.0018). | | | | | | | | | | | |
| Okawa (also in severity phenotype) 2018 | Cross-sectional | NR | Hos | Japan | Western Pacific Region | 240 | 32 ± 16.3 | 137 (57) M / 103 (43) F | Adult AD patients with mild, moderate, severe, very severe AD (according to the Japanese guidelines for AD proposed by the Research Group established by the Ministry of Health, Labor and Welfare of Japan: mild: skin involvement limited to mild eruption, moderate: <10% surface area involvement by eruption with severe inflammation, severe: 10% but <30% skin involvement by severe eruption, very severe: ≥30% of body involvement by severe eruption) and subgrouping based on morphology (erythroderma type, widespread combinations of various types, prurigo type, limb type, or head/ face/neck/chest/back type based on the individual clinical characteristics according to the modified criterion published by the JDA) | Mild: n=36 (15), moderate: n=88 (37), severe: n=72 (30),  very severe: n=44 (18)  Erythroderma type: n=42 (18), widespread type: n=137 (57),  prurigo type: n=33 (14), limb type: n=9 (3), head/face/neck/chest/back type: n=19 (8) | Serum levels of squamous cell carcinoma antigen (SCCA) 2 | Mann-Whitney U test, Kruskal-Wallis test, Dunn's multiple comparison test | Y (25) |
|  | **Results of the analysis →** | SCCA2 levels in mild, moderate, severe and very subgroups AD were significantly higher than those of healthy controls (p<0.001). Though there was no significant difference in SCCA2 levels between AD patients with mild and moderate, severe and very severe disease, higher SCCA2 levels were observed in parallel with progression of disease severity (median (IQR); mild 4.25 (1.92e5.96) ng/mL, moderate 5.35 (2.70e12.26) ng/mL, severe 11.16 (4.53e25.29) ng/mL, very severe 17.45 (5.07e36.09) ng/mL), indicating that the SCCA2 level reflects disease severity in AD patients (p<0.01 both for severe and moderate and for very severe and moderate). AD patients with erythroderma type showed significantly higher levels of SCCA2 compared to the others (p < 0.001), followed by the widespread type. On the other hand, AD patients whose lesions were not distributed systemically, such as those with limb type, had lower levels of SCCA2. In the patients with severe and very severe conditions, the ratio of the prurigo type in AD patients with lower levels of serum SCCA2 (≤2.8 ng/ml; 25% percentile) was significantly higher than the ratio in patients with higher levels of serum SCCA2 (>2.8 ng/ml), 25% (8 of 32 patients) and 7% (6 of 84 patients) respectively (p < 0.001). | | | | | | | | | | | |
| Ono 2018 | Cross-sectional | NR | Hos | Japan | Western Pacific Region | 21 | 37.38 (17-66) | 11 (52) M / 10 (48) F | AD patients with morphology classified as eczema/exudative papules and lichenification/dermatitis | AD with eczema/exudative papules: n=11 (52), AD with dermatitis/lichenification: n=10 (48) | Properties of sweat: sweat glucose concentration, GLUT2 mRNA expression in sweat glands | Unpaired t-test | Y (16) |
|  | **Results of the analysis →** | Glucose concentration was significantly higher in sweat from patients with AD who had eczema/exudative papules, compared with those who had chronic dermatitis/lichenification and healthy subjects (p<0.05). Unlike the sweat glucose concentration, GLUT2 mRNA expression was significantly lower in sweat glands from AD patients with eczema/exudative papules compared with that from those with dermatitis/lichenification (p = 0.0007). | | | | | | | | | | | |
| Silverberg 2019 | Cross-sectional | NR | Pop | U.S.A. | Region of the Americas | 602 | 46.6, 95% CI: 45.1–48.1 | 349 (58) M / 253 (42) F | 5 classes of lesional distribution: 1. lower probabilities of lesions affecting any sites;  2. higher probability of lesions involving the anterior and posterior neck and trunk;  3. lesions involving the antecubital fossae and upper extremities;  4. lesions involving the arms, posterior hands, genitals and buttocks, and to a lesser extent face, palms and legs;  5. lesions affecting all sites. | Class 1: (35.3),  class 2: (26.9),  class 3: (19.0),  class 4: (9.7);  class 5 (9.1). | Quality of life as assessed using Dermatology Life Quality Index (DLQI) | Data-driven approach to identify phenotypes, by using latent class analysis  Multivariable logistic regression | N |
|  | **Results of the analysis →** | Class 2 (adjusted odds ratio [95% confidence interval]: 7.19 [3.21–16.07], class 3 (7.11 [3.20–15.80]), class 4 (6.90 [3.07–15.50]) and class 5 (7.92 [3.54– 17.71]) were all significantly associated with higher DLQI scores compared to class 1. | | | | | | | | | | | |
| Takahashi 2013 | Cross-sectional | NR | Hos | Japan | Western Pacific Region | 35 | 32.7 ± 8.95 (16-61) | 18 (51) M / 17 (49) F | AD subjects with lichenoid eczema and either prurigo or Papules over the cubital fossa | Lichenoid eczema: n=26 (74), patients with papules/prurigo: n=21 (55) | Axon reflex-mediated sweating volume (AXR) | Unpaired t test | Y (38) |
|  | **Results of the analysis →** | AD subjects with lichenoid eczema and either prurigo or papules over the cubital fossa showed decreased AXR (more below-average sweating), compared to patients with no eczematous change (p=.0001). | | | | | | | | | | | |
| Weidinger 2006 | Cross-sectional | NR | Hos | Germany | European Region | 476 | 22.12 ± 10.76 | 196 (41.2) M / 280 (58.8) F | AD with PH, AD without PH | AD with PH: n=113 (23.7) | FLG loss-of-function mutations R501X and 2282del4 | Transmission-disequilibrium test. Chi-squared goodness-of-fit test. | N |
|  | **Results of the analysis →** | FLG mutations are associated with PH in patients with AD (p = 5.4 x 10^-6 for combined genotype (heterozygous genotype for either R501X or 2282del4), p = 3.0 x10^-4 for R501X, p = .0004 for 2282del4). | | | | | | | | | | | |
| Yamamoto-Hanada 2020 | Cross-sectional | 2009-2010, 2015-2016 | Pop | Japan | Western Pacific Region | 35 | 6 | 82 (51.6) M / 77 (48.4) F | Flexural AD | Flexural AD: n=13 (37) | Plasma levels of 72 cytokines/chemokines | Principle component analysis | Y (146) |
|  | **Results of the analysis →** | The CCL22/MDC level in plasma was found to be significantly higher in children with visible flexural dermatitis than in those without visible flexural dermatitis. | | | | | | | | | | | |
| Yasuda-Sekiguchi 2020 | Cross-sectional | 2006-2016 | Hos | Japan | Western Pacific Region | 92 | Face and neck AD: 41.9 (29-57) | Face and neck AD: 11 (61.1) M / 7 (38.9) F | Face and neck AD (AD with persistent skin eruption areas on the face and neck) | Face and neck AD: n=18 (20), Non-face and neck AD: n=74 (80) | Enrichment of single nucleotide variations (SNVs) | Fisher’s exact test, generalised linear models | N |
|  | **Results of the analysis →** | Enrichment of 12 single nucleotide variations (SNVs) in patients with face and neck AD compared with the general Japanese population in the database. Subsequent allele frequency comparison between the face and neck AD and non - face and neck AD subgroups revealed enrichment of five SNVs. Multivariate analysis using genotype data revealed that three SNVs in theTLR1, TIRAP, and PSAPL1 genes, two of the three genes are involved in the Toll-like receptor pathway associated with the innate immunity, were significantly enriched in patients with face and neck AD. | | | | | | | | | | | |
| Zhong 2016 | Cross-sectional | NR | NR | China | Western Pacific Region | 547 | 13.0 ± 12.0 | 276 (50) M / 271 (50) F | Clinical phenotypes in the Chinese Han population, including the following four dry skin phenotypes: xerosis, IV, PH, and KP scored as 0 (not present) or 1 (present). A global clinical dry skin score was determined as the sum of the scores of each dry skin phenotype (0–4). Early age of onset (>2 years, ≤2 years). AD with and without: infraorbital fold, orbital darkening, white dermatographism, perifollicular accentuation. | Age of onset (≤2 years): n=288 (52.65), infraorbital fold: n=164 (29.98), orbital darkening: n=185 (33.82), white dermatographism: n=20 (3.66), perifollicular accentuation: n=130 (23.77), xerosis: n=492 (89.95), IV: n=177 (32.36), PH: n=182 (33.27), KP: n=78 (14.26), global score ≥2: n=221 (40.40) | FLG Gene Mutation c.3321delA | Chi‑squared exact test | Y (470) |
|  | **Results of the analysis →** | Significant associations were found between c.3321delA and the AD‑associated phenotypes IV (P = 0.006, OR = 2.393, 95% CI: 1.269–4.511), PH (P = 0.022, OR = 2.064, 95% CI: 1.096–3.887) and global clinical dry skin scores ≥2 (P = 0.001, OR = 2.848, 95% CI: 1.470–5.518). There was no significant difference between c.3321delA and the other AD‑associated phenotypes (P > 0.05). | | | | | | | | | | | |

Articles in alphabetical order. Column methodological approach: presents the methodological approach for investigating associations, unless further specified (i.e. in case of data-driven approach to identify phenotypes). AD, atopic dermatitis; Hos, hospital-based; Pop, population-based; No., number of participants with (atopic) dermatitis; NR, not reported; SD, standard deviation; Y, yes; N, no; U, unclear. DLQI: Dermatology Life Quality Index. TEWL, transepidermal water loss.
