# Supplementary material for: Classifying atopic dermatitis: a systematic review of phenotypes and associated characteristics
Source: J Eur Acad Dermatol Venereol. 2022 Feb 25;36(6):807–19. doi: 10.1111/jdv.18008 (PMC9307020; doi:10.1111/jdv.18008)
Supplement: Supplementary file 1 — Table S1. Evidence tables per predefined phenotype category. [file JDV-36-807-s005.zip › jdv18008-sup-0004-Table1d.docx]

**Supplementary Table 1d. Evidence table for phenotypes based on eczema herpeticum**

| Study | Study design | Year | Setting | Country | WHO region | No. | Age -  Mean ± SD (range) | Sex – M/F, no. (%) | Phenotype description | No. (%) per phenotype | Potential associated characteristic(s) (of a priori interest) | Methodological approach | Inclusion of controls (Y (no.)/ N) |
| --- | --- | --- | --- | --- | --- | --- | --- | --- | --- | --- | --- | --- | --- |
| Beck (also in morphology characteristics group) 2009 | Cross-sectional | 2006-2008 | Hos | U.S.A. | Region of the Americas | 553 | (1-80) | NR | AD with and without a history of EH (ADEH+ and ADEH-). ADEH+ was defined as AD subjects with at least one EH episode that had a diameter ≥ 5 cm documented by a physician and HSV infection was confirmed by either PCR, Tzanck smear, immunofluorescence and/or culture. | ADEH+ n=134 (24), ADEH- n=419 (76) | Various characteristics based on detailed history, physical examination, disease severity assessments and blood draw (including eosinophil counts, serum total IgE, TARC, CTACK, HSV-1 IgG and HSV-2 IgG antibody testing). | Fisher's Exact Test, two-sample t-test, McNemar's test | Y (348) |
|  | **Results of the analysis →** | ADEH+ subjects had more severe disease based on scoring systems (EASI and Rajka-Langeland), body surface area affected (ADEH+ had greater surface area of involvement with 32% having ≥ 35% BSA compared to only 9% of ADEH-subjects) and biomarkers (circulating eosinophil counts, serum IgE, TARC and CTACK) than ADEH- subjects (p<0.001). ADEH+ subjects were also more likely to have a history of food allergy (69 vs 40%; p<0.001) or asthma (64 vs 44%; p<0.001) and were more commonly sensitized to many common allergens (p<0.001). Cutaneous infections with S. aureus or molluscum contagiosum virus were more common in ADEH+ (78% and 8%, respectively) than in ADEH-subjects (29% and 2%; p<0.001). The majority (94%) of ADEH+ subjects developed AD before five years of age in contrast to only 59% of ADEH- subjects (p<0.001). More ADEH+ subjects (58%) said “Yes” in response to the question, “Do you have keratosis pilaris, hyperlinear palms or ichthyosis?” compared to the ADEH-group (42%, p=0.005). For overall HSV status, the ADEH+ group had higher proportion of seropositive results (94.7%) than either ADEH- (65.9%, p < 0.001) or CTL (66.4%, p < 0.001). | | | | | | | | | | | |
| Bin 2014 | Cross-sectional | NR | NR | U.S.A | Region of the Americas | 40 | AD without EH: 30 ± 14.6,  AD with EH: 28.2 ± 13.7, range total group: 6-65 | NR | AD patients with and without a history of EH: ADEH+/ADEH-. ADEH+: no acute HSV-1 infection at  the time of participation, which was confirmed by physical examination by the clinical investigator and the measurement of anti–HSV-1 IgM serum titers. All patients with ADEH+ had positive serological results for anti–HSV-1 IgG. | ADEH+: n=20 (50), ADEH-: n=20 (50). | Gene expression signatures of peripheral blood mononuclear cells (PBMCs) | ANOVA, independent-samples t test | Y (20) |
|  | **Results of the analysis →** | IFNA4 and IFNA5 gene transcripts were lower in HSV-1–stimulated PBMCs from patients with ADEH+ (p<0.05).IFN-a and IL-29 mRNA and protein levels were decreased in HSV-1–stimulated PBMCs from patients with ADEH+ compared with those from patients with ADEH– and healthy subjects (p<0.05). Gene expression of interferon regulatory factor (IRF) 3, IRF7 and IRF9 was decreased in HSV-1–stimulated PBMCs from patients with ADEH+ (p<0.05). | | | | | | | | | | | |
| Boorgula 2019 | Cross-sectional | NR | NR | U.S.A | Region of the Americas | Discovery study: 173; Replication study: 89 | Range mean age (range SD): 14.7-39.3 (11.4-21.4) | Discovery study: 103 (60) M / 70 (40) F;  Replication study: 68 (76) M / 21 (24) F | AD with and without eczema herpeticum (ADEH+ vs ADEH-). ADEH+ subjects were defined as patients with AD who had at least one previous EH episode as physician documented  in Beck et al. ADEH-subjects were defined as patients with AD with no history of EH. | Discovery study: ADEH+: n=90 (52), ADEH−: n=83 (48);  Replication study: ADEH+: n=36 (40), ADEH−: n=53 (60). | DNA methylation differences in whole blood cells, adjusting for six or seven cell types. | Linear models | Y (84) |
|  | **Results of the analysis →** | Genome-wide comparison: One CpG (cg18593727) showed genome-wide significantly differential methylation between the ADEH+ patients and the healthy control group in our discovery analysis and showed suggestive replication (discovery FDR adjusted q-value 0.0426, replication nominal p value 0.0345; adjusting for seven cell types, including eosinophils). Targeted gene comparison: Two CpGs, one in IL4 (cg23943829) and one in IL13 (cg04303330), showed significant differential methylation between ADEH+ and healthy controls in the discovery analysis (FDR adjusted q-values of 0.03 and 0.04, respectively) and suggestive significance, including similar effect sizes in the same direction, in replication (nominal p values of 0.051 and 0.094, respectively; adjusting for seven cell types, including eosinophils). Comparing ADEH+ with healthy controls, 490 CpGs were differentially methylated (FDR-adjusted q-value < 0.05; adjusting for six main cell types). Comparing ADEH− with controls, six CpGs were differentially methylated (FDR-adjusted q-value < 0.05). There were no CpGs that were significantly different between the ADEH− and ADEH+ phenotype groups. | | | | | | | | | | | |
| Broccardo 2011 | Cross-sectional | NR | Hos | U.S.A | Region of the Americas | 35 | AD without EH: 30.6 ± 18.4,  AD with EH: 22 ± 16.1, range total group: 1-80 | 19 (54) M / 16 (46) F | AD patients with and without history of EH | 18 AD-EH: n=18 (51.4), AD+EH: n=17 (48.6) | Proteins related to the skin barrier | Linear mixed model | Y (6) |
|  | **Results of the analysis →** | Proteins related to the skin barrier (FLG-2, corneodesmosin, desmoglein-1, desmocollin-1, and transglutaminase-3) and generation of NMF (arginase-1, caspase-14, and gamma-glutamyl cyclotransferase) were expressed at significantly lower levels in lesional versus nonlesional sites of patients with AD with and without history of EH. FLG-2 was expressed at significantly lower levels in EH-/EH+ nonlesional versus nonatopic skin. No statistical difference regarding lesional sites in EH- vs EH+ and non-lesional sites in EH- vs EH+. | | | | | | | | | | | |
| Gao 2015 | Cross-sectional | NR | NR | U.S.A | Region of the Americas | 457 | NR | NR | AD complicated by a history of EH (ADEH+) vs ADEH- in European American and African American patients. Patients with ADEH+ were defined as patients with AD who had a history of at least 1 EH episode, confirmed by either PCR, Tzanck smear, immunofluorescence and/or culture. | European American ADEH+: n=121,  ADEH-: n=336 (167 European American, 169 African American) | Variants in interferon pathway genes (IFNG, IFNGR1, IFNAR1, and IL12RB1) | Logistic regression | Y (323) |
|  | **Results of the analysis →** | 6 rare IFNGR1 missense variants, including 3 damaging variants (Val14Met [V14M], Val61Ile, and Tyr397Cys [Y397C]) were found conferring a higher risk for ADEH+ (P = .031). Variants V14M and Y397C were confirmed to be deleterious, leading to partial IFNGR1 deficiency. Seven common IFNGR1 SNPs (rs11914, rs17175127, rs1327475, rs10457655, rs7749390, rs2234711, rs28515059), along with common protective haplotypes (2-7 SNPs ), conferred a reduced risk of ADEH+ (P = .015-.002 and P = .0015-.0004, respectively), and both SNP and haplotype associations were replicated in an independent African American sample (P = .004-.0001 and P = .001-.0001, respectively). | | | | | | | | | | | |
| Gao 2010 | Cross-sectional | NR | NR | U.S.A | Region of the Americas | NR | NR | NR | AD with and without EH (ADEH+, ADEH-), defined as AD patients with at least one EH episode documented either by an investigator or diagnosis by another physician confirmed by HSV PCR, tissue immunofluorescence, Tzanck smear and/or culture in European American and African American patients. | NR | Thymic stromal lymphopoietin (TSLP) gene polymorphisms. 29 SNPs from TSLP, IL7R, and TSLPR (15, 11, and 3) were genotyped. | Cochran-Armitage trend test | N |
|  | **Results of the analysis →** | Significant associations were observed for ADEH+ in comparison to ADEH- subjects in the primary European American group and 2 TSLP SNPs (rs1898671, OR (95% CI): 0.6 (0.4-0.9), P = .001; rs2416259, OR (95% CI): 2.0 (1.1-3.6), P = .021) and 4 IL7R SNPs (rs12516866, OR (95% CI): 0.6 (0.3-1.0), P = .044; rs10213865, OR (95% CI): 0.6 (0.3-1.0), P =.027; rs1389832, OR (95% CI): 2.0 (1.1-3.7), P = .011; rs10058453, OR (95% CI): 1.9 (1.1-3.4), P = .022). The number of patients with ADEH in the African American sample was too small to perform meaningful tests for association. | | | | | | | | | | | |
| Gao 2009 | Cross-sectional | NR | Pop | U.S.A | Region of the Americas | 465 | Mean age (SD): range: 17.9-41.1 (10.3-22.1) | 295 (63) M / 170 (27) F | ADEH was defined as AD patients with at least one EH episode documented either by an investigator or diagnosis by another physician confirmed by HSV PCR, tissue immunofluorescence, Tzanck smear and/or culture in European American and African American patients. | ADEH: n=144 (31), ADEH-: N=321 (69) | Two common loss-of-function mutations (R501X, 2282del4) | Cochran–Armitage trend test, Fisher’s exact test | Y (309) |
|  | **Results of the analysis →** | The frequency of the R501X mutation was three times higher (25.4% vs 9%) for ADEH compared to AD without EH (odds ratio [OR]=3.4 (1.7–6.8), P=0.0002). No difference was found for 2282del4 (p=0.465). Associations with ADEH were stronger with the combined null mutations (OR=10.1 (4.7–22.1), P=1.99×10−11), compared to the risk for individuals carrying either the R501X mutation or the 2282del4 alone. Associations with the R501X mutation were replicated in the African American population; the R501X mutation conferred a greater risk of ADEH (OR=5.28 (1.0–27.4), P=0.0289) compared to AD patients without EH. No difference was found for 2282del4. | | | | | | | | | | | |
| Hinz 2011 | Cross-sectional | 2006-2009 | Hos | Germany | European Region | 174 | (15-60) | 96 (55) M / 78 (45) F | Four groups based on EH in AD: (i) AD patients with acute EH and recurrent HSV infections (ADEH+HSV+ acute), (ii) AD patients with history of EH and recurrent HSV infections (ADEH+HSV+ history), (iii) AD without EH but with recurrent HSV infections (ADEH-HSV+), (iv) AD without EH or HSV infections (ADEH-HSV-) | (i) ADEH+HSV+ acute: n=21 (12), (ii) ADEH+HSV+ history: n=31 (18), (iii) ADEH-HSV+: n=61 (35), (iv) ADEH-HSV-: n= 61(35) | Clinical and laboratory parameters (sex, heamatology cell counts, IgE serum levels, disease severity by EASI and SCORAD, concomitant asthma and allergic rhinitis) | Chi-squared test, Fisher’s exact test | Y (61) |
|  | **Results of the analysis →** | More male patients (71% and 77%) with AD were affected by EH (both acute and history) than female patients (29% and 23%). Acute episodes of EH are characterized by lower levels of lymphocytes and higher levels of monocytes, compared to patients with history of EH (p<0.05). AD patients with history of EH display higher total IgE serum levels (ADEH+HSV+ vs ADEH-HSV+, P < 0.001) and higher sensitization profiles and stronger severity of AD (EASI and SCORAD; ADEH+HSV+ vs ADEH-HSV+, P < 0.001). Patients with EH had significantly higher frequency of concomitant physician diagnosed asthma (64% vs 27%; P < 0.001) and allergic rhinitis (86% vs 58%; P < 0.001) than ADEH-HSV+ patients. | | | | | | | | | | | |
| Mathias 2013 | Cross-sectional | NR | NR | U.S.A. | Region of the Americas | 44 | NR | NR | AD with a history of EH (ADEH+), AD without a history of EH (ADEH-) | ADEH+: n=24 (55), ADEH–: n=20 (45) | Cytokine expression by of peripheral blood mononuclear cells: CD4, CD8, CD14, CD107a, CD3, CD16, CD56, interferon (IFN)-c, tumour necrosis factor (TFN)-a, tumour necrosis factor (TFN)-c, interleukin (IL)-10, IL-13 and IL-17. | Analysis of covariance (ANCOVA) models | Y (10) |
|  | **Results of the analysis →** | IFN-c expression after HSV treatment was lower in the CD8+ T cells and monocytes from patients with ADEH+ compared with patients who are ADEH– or nonatopic. Statistically significant difference in HSV cell mediated immunity responses (P < 0.05) between ADEH+ and nonatopic controls for CD8+ IFN-c+ T cells. Detailed dot plots show that ADEH+, but not ADEH–, subjects had lower percentages of HSV-specific CD8+ IFN-c+ T cells (P = 0.025) than nonatopic subjects. No differences were observed in the CD4+ IFN-c+ or the Natural killer cell (NK) expression of IFN-c after HSV stimulation. No significant differences were observed between ADEH+ and ADEH– subjects for the percentage of IL-10+, IL-13+ or IL-17+ CD4+ or CD8+ T cells after mock or HSV stimulation. However, the ratio of IFN-c– to IL-10-producing cells (P = 0.031) as well as the ratio of TNF-a to IL-10-producing cells (P = 0.017) were significantly lower in CD14+ cells after HSV-stimulation in the ADEH+ samples. | | | | | | | | | | | |
| Narla 2018 | Cross-sectional | 2002-2012 | Hos | U.S.A. | Region of the Americas | 165,199 | 58.6 (SEM: 0.2) | 440,151 (55.8%) F | Patients with AD hospitalized for EH | NR | Serious infections were defined as infections which lead to hospitalization, were life threatening, or required treatment in an inpatient setting | Logistic regression models | Y (341,915,592) |
|  | **Results of the analysis →** | Patients hospitalized with AD and eczema and no EH had significantly higher odds of cellulitis (1.79 [1.52–2.11]), erysipelas (21.38 [14.90–30.69]), acute sinusitis (6.63 [4.03–10.90]), and pyelonephritis (4.15 [2.52–6.83]), but not of fungal infection (1.16 [0.80-1.69]), compared to patients without AD and eczema or EH. However, patients with AD and eczema and EH had an even higher odds of cellulitis (3.56 [2.65–4.78]). | | | | | | | | | | | |
| Takahashi 2014 | Case-control | NR | NR | Japan | Western Pacific Region | 56 | ADEH+: 27.6 ± 2.2,  ADEH-: 32.8 ± 2.5 | NR | AD with (recurrent) EH (ADEH+) patients, AD patients without EH (ADEH-) | ADEH+: n=39 (70), ADEH−: n=17 (30) | Frequencies, phenotype and function of Tregs in the peripheral blood | Student t test, Dunnett test | Y (34) |
|  | **Results of the analysis →** | A significant decrease in the frequencies of γδ+ T cells and CD56+ NK cells was found in AD patients, irrespective of EH (p<0.001 for all groups), compared with healthy controls. In contrast, there were no significant differences in the frequencies of CD8+ T cells. AD patients with EH had a significantly higher frequency of CD4+CD25+ Foxp3+ Tregs in PBMCs at the onset of disease compared with after disease resolution: the mean frequencies of Tregs at the onset of EH were higher than those in ADEH- and in healthy controls. The frequencies of CLA+ and CCR4+ Tregs at the onset of disease were significantly higher than those after resolution of EH. The frequencies of Foxp3+ CTLA-4+, Foxp3+CD127dim/2, Foxp3+CD39+, Foxp3+HELIOS+, and Foxp3+Ki67+ Tregs were increased significantly at the onset of EH. Although rTregs and non-Tregs were slightly increased in the acute stage of ADEH, iTregs were dramatically increased in the acute stage of EH compared with those in ADEH- and those in healthy controls (p<0.05). After resolution of EH, the frequencies of Tregs decreased to values similar to those in healthy controls. Tregs obtained from AD patients with EH, either at the onset of disease or after its resolution, retained the suppressive capacity to inhibit proliferation of effector T cells, similar to healthy controls. IFN-g production by HSV-1–specific CD8+ and CD4+ T cells was restored upon depletion of Tregs from PBMCs obtained from AD patients with EH at onset of disease. Treg-suppressive capacity was retained in ADEH patients and, the expanded Tregs suppressed IFN-g production from HSV-1– specific CD8+ and CD4+ T cells. The increased frequency of CD14dimCD16+ proinflammatory monocytes (pMOs) was also observed in the blood and EH skin lesions. Thus, pMOs detected in ADEH patients at onset were characterized by an increased ability to produce IL-10 and a decreased ability to produce proinflammatory cytokines, unlike their normal counterparts. Our coculture study using Tregs and pMOs showed that the pMOs can promote the expansion of inducible Tregs. Tregs were detected frequently in the vicinity of HSV-expressing and varicella zoster virus–expressing CD16+ monocytes in the EH lesions. | | | | | | | | | | | |

Articles in alphabetical order. Column methodological approach: presents the methodological approach for investigating associations, unless further specified (i.e. in case of data-driven approach to identify phenotypes). AD, atopic dermatitis; Hos, hospital-based; Pop, population-based; No., number of participants with (atopic) dermatitis; NR, not reported; SD, standard deviation; Y, yes; N, no; U, unclear. TEWL, transepidermal water loss. EH: eczema herpeticum.
