# Supplementary material for: Classifying atopic dermatitis: a systematic review of phenotypes and associated characteristics
Source: J Eur Acad Dermatol Venereol. 2022 Feb 25;36(6):807–19. doi: 10.1111/jdv.18008 (PMC9307020; doi:10.1111/jdv.18008)
Supplement: Supplementary file 1 — Table S1. Evidence tables per predefined phenotype category. [file JDV-36-807-s005.zip › jdv18008-sup-0005-Table1e.docx]

**Supplementary Table 1e. Evidence table for phenotypes with associated morphological features**

| Study | Study design | Year | Setting | Country | WHO region | No. | Age -  Mean ± SD (range) | Sex – M/F, no. (%) | Phenotype description | No. (%) per phenotype | Potential associated characteristic(s) (of a priori interest) | Methodological approach | Inclusion of controls (Y (no.)/ N) |
| --- | --- | --- | --- | --- | --- | --- | --- | --- | --- | --- | --- | --- | --- |
| Beck (also in eczema herpeticum group) 2009 | Cross-sectional | 2006-2008 | Hos | U.S.A. | Region of the Americas | 553 | (1-80) | NR | AD with and without a history of EH (ADEH+ and ADEH-). ADEH+ was defined as AD subjects with at least one EH episode that had a diameter ≥ 5 cm documented by a physician and HSV infection was confirmed by either PCR, Tzanck smear, immunofluorescence and/or culture. | ADEH+ n=134 (24), ADEH- n=419 (76) | Various characteristics based on detailed history, physical examination, disease severity assessments and blood draw (including eosinophil counts, serum total IgE, TARC, CTACK, HSV-1 IgG and HSV-2 IgG antibody testing). | Fisher's Exact Test, two-sample t-test, McNemar's test | Y (348) |
|  | **Results of the analysis →** | ADEH+ subjects had more severe disease based on scoring systems (EASI and Rajka-Langeland), body surface area affected (ADEH+ had greater surface area of involvement with 32% having ≥ 35% BSA compared to only 9% of ADEH-subjects) and biomarkers (circulating eosinophil counts, serum IgE, TARC and CTACK) than ADEH- subjects (p<0.001). ADEH+ subjects were also more likely to have a history of food allergy (69 vs 40%; p<0.001) or asthma (64 vs 44%; p<0.001) and were more commonly sensitized to many common allergens (p<0.001). Cutaneous infections with S. aureus or molluscum contagiosum virus were more common in ADEH+ (78% and 8%, respectively) than in ADEH-subjects (29% and 2%; p<0.001). The majority (94%) of ADEH+ subjects developed AD before five years of age in contrast to only 59% of ADEH- subjects (p<0.001). More ADEH+ subjects (58%) said “Yes” in response to the question, “Do you have keratosis pilaris, hyperlinear palms or ichthyosis?” compared to the ADEH-group (42%, p=0.005). For overall HSV status, the ADEH+ group had higher proportion of seropositive results (94.7%) than either ADEH- (65.9%, p < 0.001) or CTL (66.4%, p < 0.001). | | | | | | | | | | | |
| Bohme 2001 | Case-control | 1995-1998 | Pop, Hos | Sweden | European Region | 221 | (0-29 mo) | 119 (54) M / 102 (46) F | Young children (2-year old children) with ongoing AD | Ongoing: n=157 (71) | Morphology: lesional distribution of eczema | No formal statistical test | Y (99) |
|  | **Results of the analysis →** | Of the children with ongoing AD 69% had eczema on the legs, 45% on the trunk, 36% on the arms, 28% on the hands and 26% on the head. A history of eczema in the nappy region was reported in 36%. | | | | | | | | | | | |
| Bohme 2000 | Case-control | 1995-1998 | Pop, Hos | Sweden | European Region | 221 | (0-29 mo) | 119 (54) M / 102 (46) F | 2-year-old children with ongoing and latent AD | Ongoing: n=157 (71), latent: n=64 (29) | The minor criteria of the Hanifin’s and Rajka’s AD criteria, including morphological characteristics | Chi-squared test, Fisher exact test | Y (99) |
|  | **Results of the analysis →** | Significantly more frequent than in controls (p<0.05): xerosis (100% in patients with ongoing AD, 97% in patients with latent AD, 40% in controls) and facial erythema (54% in patients with ongoing AD, 39% in patients with latent AD, 25% in controls). No significant differences were found for the other morphological characteristics. | | | | | | | | | | | |
| Bremmer (also in morphology phenotype group) 2008 | Cross-sectional | 1978-2006 | Hos | U.S.A. | Region of the Americas | 1187 | 21.4 ± 17.9 (0-83) | NR | AD with (mild/moderate/severe) and without coexisting IV | AD+IV: n= 321 (32.0)  AD-IV: n=654 (68.0) (missing data: n=212) | Disease severity, age at onset, probability of having allergic respiratory disease (previous diagnosis or symptoms of ARD: asthma and allergic rhinoconjunctivitis), physical features (IV, PH, and KP, were graded on a 0-3 scale (0 = absent, 1 = mild, 2 = moderate, and 3 = severe).) | Chi-squared test | N |
|  | **Results of the analysis →** | Patients with IV than in those without (39.9% vs 32.9%, OR = 1.35, P = .050) and were most associated with severe IV (55.3% vs 32.9%. OR = 2.52, P = .002). Relationships between IV and asthma remained significant after adjusting for age, gender, season of examination, and AD severity (OR = 1.78, P= .019). A higher likelihood of concomitant allergic rhinoconjunctivitis symptoms was found if there was clinical evidence of IV (61.8% vs 53.2%, P = .018). No significant association with increased severity of IV. After adjustment for the severity of AD, age, gender, and season, the relationship between IV and allergic rhinoconjunctivitis remained significant (p = .002). A much stronger, significant association was seen between severe IV and allergic rhinoconjunctivitis symptoms (OR = 2.84, P =.048). Clinical IV was also associated with earlier onset of AD (71.1% vs 61.7% <2 y/o; p= .009), HP (81.3% vs 43.0%; P<.001) and KP (52.9% vs 28.4%; p<.001). | | | | | | | | | | | |
| Brenninkmeijer 2008 | Case-control | 2000-2005 | Hos | The Netherlands | European Region | 156 | 15 | 44 (28) M / 112 (72) F | Paitients with a clinical diagnosis of AD who at least once had tested positive for allergen-specific IgE levels (true AD), atopiform dermatitis (AFD) defined as patients with a clinical diagnosis of AD with negative outcomes of both a skin prick test and Phadiatop test. | AD: n=122 (78), AFD: n=34 (22) | Diagnostic features (based on the Hanifin and Rajka, U.K and Millennium criteria.) | Chi-squared test, Fisher’s exact test, Mann-Whitney test | N |
|  | **Results of the analysis →** | The Dennie-Morgan fold was significantly more present in AFD patients compared with AD patients (38.2% vs 19.7%;OR = 2.76; 95% CI, 1.14-5.53). PH (OR = 0.16; 95% CI, 0.06-0.40), KP (OR = 0.27; 95% CI, 0.07-0.69), pityriasis alba (OR = 0.30, 95% CI, 0.12-0.65), and nonspecific hand or foot eczema (OR = 0.31; 95% CI, 0.69-0.75) were less frequently observed in AFD patients compared with AD patients. Many other features are tested, but were found not statistically significant. | | | | | | | | | | | |
| Carson 2012 | Cross-sectional | 1998-2008 | Hos | Denmark | European Region | 170 | NR | NR | Children with AD and with or without R501X and/or 2282del4 FLG null mutations | FLG null mutation carriers: n=16 (15.3) | Anatomical localization of dermatitis lesions (35 predefined areas) and morphology of single dermatitis lesions (including area (in percent of the total body surface area)) | Chi-squared test,  GEE-model, Partial Least Squares Discriminant Analysis (PLSDA), Principal Component Analysis (PCA). | Y (212) |
|  | **Results of the analysis →** | Involvement of the palm and back of the hands, the flexor and extensor extremities, the feet and the cheeks was statistically significant associated with the FLG null genotype (p values between <0.0001 and 0.002). Cheeks and back of the hands were significantly selected by the PLSDA. FLG mutations were associated with (more severe (moderate-severe SCORAD 44% vs. 31%; p = 0.14),) and widespread dermatitis (10% vs. 6% of the body area, p<0.001) compared to wild-type. | | | | | | | | | | | |
| Chu 2017 | Cross-sectional | 2007-2015 | Hos | Korea | FLG null mutation carriers | 5000 | 18.99 ± 10.22 (0-86) | 2590 (51.8) M / 2410 (48.2) F | AD in infancy ≤2 years old, childhood and adolescence >2 years old and ≤18 years old, adulthood >18 years old | Infancy: n=205 (4.1), childhood and adolescence: n=2240 (45), adulthood: n=2555 (51) | Anatomical involvement | No formal statistical test | N |
|  | **Results of the analysis →** | Infants and adults showed involvements in their head and neck area the most (25.3% and 26.7%, respectfully), whereas in patients in their childhood and adolescence, the upper extremities were the most involved body part (21.9%). | | | | | | | | | | | |
| Dezoteux (also in trajectory group) 2019 | Cross-sectional | 2007-2016 | Hos | France | European Region | 533 | ≥ 18 years; AD≥45: range: 45-78 | AD≥45: 66 (53) M / 58 (47) F | Atopic dermatitis (AD) in adults over 45 years of age (AD≥45) and below 45 years (<45); Among AD≥45 patients, 20% (n=25) were categorized into Subgroup 1 (persistence of AD since childhood), 52% (n=64) into Subgroup 2 (recurrence of AD with a history of classic childhood AD), and 28% (n=35) into Subgroup 3 (adult-onset AD). | AD≥45: n=124 (23), AD<45: n=409 (77). | Age, gender, level of education, association with asthma and/or allergic rhinoconjunctivitis, family history of atopy, active smoking, and predominant skin region affected by AD lesions  (eczema, redness, oedema, lichenification, excoriations). | Chi-squared test, Fisher’s exact  Test, Kruskal-Wallis test | N |
|  | **Results of the analysis →** | Skin lesions predominated on the face and neck in AD≥45 patients with AD since childhood (30% in Subgroups 1 and 2) compared to those with adult-onset AD (14% in Subgroup 3) (no p-value reported). Gender (p=0.78), asthma and/or allergic rhinoconjunctivitis (73%, 76% and 75% 3 in Subgroups 1, 2 and 3, respectively; p = 0.94), a family history of atopy (83%, 84% and 79% in Subgroups 1, 2 and 3, respectively; p = 0.88), active smoking (24%, 21% and 23% in Subgroups 1, 2 and 3, respectively; p = 0.93), and level of education (17%, 36% and 29% in Subgroups 1, 2 and 3, respectively; p = 0.27) were similar in the three subgroups regarding AD onset. | | | | | | | | | | | |
| Ezzedine (both morphology groups, disease trajectory group) 2012 | Cross-sectional | 2007-2008 | Hos | France | European Region | 110 | 36.6 ± 16.2 (18–85) | 49 (45) M / 61 (55) F | Adult caucasian AD patients with or without IV and with or without early onset (≤ 2 years vs > 2 years) | AD+IV: n=74 (67), AD-IV: n=36 (33)  Early onset: n=55 (50), late onset: n=28 (25), missing data for onset: n=23 (21) | Clinical signs of IV scored 0 (not present) to 3 (very severe) – diffuse xerosis, PH, scales on legs, scalp desquamation and KP. Global IV clinical severity score (0–15). Age of onset of AD, SCORAD, family/personal history for AD, allergic rhinitis, allergic conjunctivitis or asthma, and total IgE. FLG mutations R501X, S2282del4, S3247X and R2447X. | Univariate and sub sequent multivariate  unconditional logistic regression analysis | N |
|  | **Results of the analysis →** | Univariate analysis: family history of atopy (OR 4.57, P = 0.01), xerosis (OR 4.67, P = 0.0003), PH (OR 10.71, P < 0.0001), scale on legs (OR 11.00, P < 0.0001), age (OR 2.15, P < 0.0001) and 2282del4 FLG (OR 5.41, P = 0.0010) mutation were positively correlated with the AD + IV phenotype. Multivariate analysis: only SCORAD for AD (OR 0.94, P = 0.01) and global clinical severity scoring for AD + IV (OR 2.62, P < 0.0001) were found to be independent factors. No significant differences were found for sex, R501X mutation, presence of other atopic symptoms, scalp desquamation, KP, age of onset, total IgE and presence of specific IgE between the groups. No S3247X or R2447X mutations were detected. 2282del4 mutation was significantly associated with early-age onset (OR 4.87, P = 0.04). No significant association between R501x mutation and age of onset | | | | | | | | | | | |
| Foley (also in severity group) 2001 | Cross-sectional | 1998-1999 | Pop, Hos | Australia | Western Pacific Region | 346 | (0-5) | 178 (51) M / 168 (49) F | Minimal, mild, moderate, severe AD severity in preschool-age Australian children: severity was graded as minimal (disease that the parent may not have been aware of and that would require no treatment or would respond well to simple emollients available without prescription), mild (disease that might require attention from a medical practitioner with the use of minimal prescription-only treatments), moderate (disease that would require attention from a medical practitioner, plus the use of more potent prescription-only topical steroids as well as emollients), and severe (disease that requires management by a dermatologist) | Minimal: (4.9), mild: (58.8), moderate (34.5), severe (1.7) | (Number of) affected sites, personal history of asthma or hay fever or a family history of atopy | Unclear: no (reporting of) formal statistical test | Y (770; unclear if included in analyses) |
|  | **Results of the analysis →** | With increasing severity, more sites were likely to be affected. The severity did not correlate with a personal history of asthma or hay fever or a family history of atopy. Flexures were affected in 72.5% of children with AD. Face: 60.1%, trunk: 38.2%, limbs: 37.6%, diaper area: 7.2%, all other sites: 4.9%. Only 20 (5.8%) of all children with AD did not have face or flexural involvement. | | | | | | | | | | | |
| Garmhausen (also in trajectory group) 2013 | Cross-sectional | NR | Hos | Germany | European Region | 725 | 32.6 ± 14.0 (12–89) | 293 (40) M / 432 (60) F | Course type of AD based on age of onset and affection during the following phases (phases based on classification of Wuthrich): infantile phase 0-2 years, childhood phase 2-6 years, juvenile phase 6-14 years, adolescent phase 14-20 years, adult phase > 20 years.  Five main course types were identified: early onset of AD before the 2nd year of life and a chronic persisting course until adulthood (type 5); start of AD after the 20th year of life (type 31); start AD in childhood phase (2-6years) with a chronic persisting course until adulthood (type 20); start in adolescent phase (14-20 years) with a chronic persisting course until adulthood (type 30); start in juvenile phase (6-14 years) with a chronic persisting course until adulthood (type 27). | 607 patients could be classified into course types. Of these 607 patients 85.7% could be classified into five main different course types of all 31 course types recorded.  Type 5: n=189 (31.1). Type 31: n=112 (18.5). Type 20: n=84 (13.8), type 30: n=77 (12.7), type 27 n=58 (9.6). | Atopy signs and concomitant atopic diseases (criteria of Diepgen, including morphological features and serum IgE) | Logistic or linear  regression | N |
|  | **Results of the analysis →** | More food intolerance in course type 5 in comparison with the other four most frequent course types (p<0.05). Type 5 more often had flexural eczema (compared to course type 30; P = 0.0003 and course type 31 P < 0.0001). In comparison with course type 31, course type 5 showed a significantly higher occurrence of rhinoconjunctivitis (P < 0.0001), milk crust (P < 0.0001), Hertoghe sign (P = 0.0091), Dennie–Morgan infraorbital fold (P = 0.001), orbital darkening (P = 0.0007), white dermographism (P = 0.0031), predilection of skin lesions in the neck region (P = 0.0206), impact of psychic factors on the course of their disease (P = 0.0105) and asthma bronchiale (P < 0.0001). Higher total serum IgE levels in course type 5 in comparison to types 27, 30 and 31 (p<0.05). Allergen-specific IgE towards peanut, hazelnut and apple, cat dander, birch and grass pollen was more frequent in type 5 than the other four types (p<0.05) Non-allergic AD (low IgE serum levels (<150kU/l) and no sensitisation to aero or food allergens) was found in 25% of course type 31, significantly higher than course type 5 (6.4% P<0.0001). | | | | | | | | | | | |
| Guglielmo (both morphology groups, disease trajectory group) 2020 | Cross-sectional | 2005-2020 | Hos | Italy | European Region | 31 | (11-62) | 16 (52) M / 15 (48) F | Adolescent-onset and adult-onset head and neck dermatitis (HND) (cut-off at 18 years) | Adolescent-onset HND: n=17 (55), adult-onset HND: n=14 (45), head and neck dermatitis: 100% | Age, sex, HND onset, AD distribution (exclusive HND vs diffuse AD including HND involvement), past medical history of AD or atopy (personal or family), IgE serum levels | Fisher exact test, t-test | N |
|  | **Results of the analysis →** | Adolescent-onset HND positively correlated with a past history of AD of the classic type and presented with exclusive head and neck involvement (100% and 59%, respectively) (P < .05). Adult-onset HND was associated with concomitant widespread atopic eczema, involving the flexural areas of the upper and lower limbs, trunk, nipples, or hands (68%) (P < .05). Increased serum IgE level (>100 IU/mL) was detected in 11/17 (65%) adolescents and 11/14 (78%) adult patients. No differences were observed between the two groups in terms of AD family history or personal atopy history, including asthma, food allergy, allergic rhinitis, or conjunctivitis. | | | | | | | | | | | |
| Guo 2019 | Cross-sectional | 2013-2014 | Pop | China | Western Pacific Region | 1,819 | 6.36 ± 5.19 (1-12 mo) | 1074 (59) M / 745 (41) F | Infantile AD | 100% | Presence of skin lesions (facial, scalp, vulvar, extensor surfaces of the limbs, flexural, infra-auricular and retroauricular fissuring, eyelid, nummular, cheilitis, nipple, non-specific hand/foot, acrocheir, infranasal fissuring, prurigo type), atopy signs and minor signs related to AD (xerosis, palmar hyperlinerarity, keratosis pilaris, orbital darkening, dennie-morgan folds, hertoghe sign, perifollicular accentuation, ichthyosis vulgaris, pityriasis alba) | Chi-squared test, logistic regression analysis | Y (4,148) |
|  | **Results of the analysis →** | The most commonly seen manifestations in infantile AD were facial dermatitis (72.07%), xerosis (42.72%) and scalp dermatitis (27.93%). The percentages for the other skin lesions and atopy signs ranged from 0.11% to 12.20% and 0.22% to 4.73%, respectively. These included palmar hyperlinearity (4.73%), perifollicular accentuation (3.96%), ichthyosis vulgaris (2.86%), pityriasis alba (2.80%), keratosis pilaris (1.54%), orbital darkening (1.54%) and Dennie–Morgan folds (0.82%). | | | | | | | | | | | |
| Heede 2015 | Cohort | 2006-2013 | Pop | Denmark | European Region | 199 | NR | NR | Presence or absence of FLG mutations in adult patients with AD | FLGwt/+AD: n=161 (81): participants with no mutations with AD, FLGmut/+AD: n=38 (19): participants with both mutations and AD | Presence of dermatitis on the hands (persistent vs occasional hand dermatitis), feet, face, axillae, abdomen, chest, and/or back | Chi-squared test, Fisher exact test, Cochran-Armitage trend test, logistic regression models | Y (1944) |
|  | **Results of the analysis →** | FLG mutations increased the prevalence (OR) of foot dermatitis (OR 10.41; 95% confidence interval 5.27-20.60; p=0.005) and persistent hand dermatitis (OR 17.57; 95% confidence interval 8.60-35.89; p=0.038) in patients with AD, in comparison to controls with no FLG mutations and no history of AD. No statistical differences were found for occasional hand dermatitis, face dermatitis, axillae dermatitis and abdomen, chest, or back dermatitis. | | | | | | | | | | | |
| Heede (also in other morphology group) 2017 | Cross-sectional | 2006-2012 | Hos | Denmark | European Region | 233 | (18-83) | 50 (21) M / 182 (79) F | AD with and without hand eczema | AD + HE: n=131 (56), AD only: n=102 (44) | Gender, occupational dermatitis, facial dermatitis, age, positive patch test reactions, FLG mutation status (R501X, 2282del4, and R2447X) | Kruskal-Wallis test, Chi-squared test | Y (287) |
|  | **Results of the analysis →** | Patients in the group with ‘hand eczema only’ were significantly older (p<0.001, Kruskal-Wallis test). Patients with only hand eczema had the highest prevalence of ≥1 positive patch test reaction (50.5%), followed by patients with both hand eczema and AD (40.5%) and patients with only AD (34.3%) (p=0.009, 𝜒2 test). Similar results were found for occupational dermatitis (61.0%, 38.9%, 2.0%, respectively). More facial dermatitis was found in patients with AD (64.7%) compared to patients with AD+HE (34.4%) or HE (12.2%). No statistical difference was found for gender. The prevalence of FLG mutations carriers was significantly higher in the group of patients diagnosed with both AD and hand eczema (33.1%) than in the group with AD only (18.8%) and the hand eczema group (8.9%) (p<0.001, 𝜒2 test). | | | | | | | | | | | |
| Holm (in trajectory, severity and morphological characteristics group) 2019 | Cross-sectional | 2012-2017 | Hos | Denmark | European Region | 470 | 18.7 ± 16.5 | 214 (45.5) M / 256 (54.5) F | AD subgroups based on SCORAD: mild (< 25), moderate (25–50) and severe (> 50); small children (< 4 years of age), children/adolescents (age 4–15 years) and adults (> 15 years of age); early-onset (< 1 year of age), late-onset (>1 year of age) | Small children: n=122 (26), children/adolescents: n=103 (22) and adults: n=245 (52).  Mild: n= 166 (35.3), moderate: n= 218 (46.4), severe: n= 86 (18.3).  Early-onset: n=141 (47.8), late-onset: n=154 (52.2). | FLG mutations (R2447X, R501X and 2282del4), serum total IgE, blood eosinophil count; self-rated health, eczema distribution in the past month | ANOVA, chi-squared test, independent t-test | N |
|  | **Results of the analysis →** | A significant difference between severity groups in small children was observed for FLG mutation carrier status (16.7 vs. 30.2 vs. 60.0% mutation carriers among patients with mild, moderate and severe AD, respectively, p = 0.012) and self-rated health (3.2 vs. 2.7 vs. 2.8 with 4 being excellent health, p = 0.022).  In the subgroup of children/adolescents, disease severity was statistically significantly associated with CDLQI (5.6 vs. 7.3 vs. 8.8 among patients with mild, moderate and severe AD, respectively, p = 0.048). A significant difference between severity groups in adults was observed for male sex (24.4 vs. 39.8 vs. 52.9%, p = 0.003), serum total IgE (577 vs. 1269 vs. 2379 × 103 IU/L, p < 0.001), blood eosinophil count (0.28 vs. 0.39 vs. 0.61 × 109/L, p < 0.001) and asthma (42.9 vs. 38.8 vs. 72.0%, p < 0.001), hand eczema (66.2 vs. 81.6 vs. 88.0%, p = 0.007), flexural eczema (67.5 vs. 74.7 vs. 90%, p = 0.015), and DLQI (6.9 vs. 9.9 vs. 13.8, p < 0.001). Compared to no early onset of AD and no FLG mutations, early onset of AD and FLG mutation was associated with more severe disease (higher mean SCORAD (33.3 vs. 41.6, p = 0.012)) and high serum total IgE levels (903 vs. 1961 × 103 IU/L, p = 0.047). | | | | | | | | | | | |
| Imayama 1992 | Cross-sectional | 1990-1991 | Hos | Japan | Western Pacific Region | 172 | 20.4 (6 mo-63 y) | 77 (44.8) M / 95 (55.2) F | Four subgroups based on Dermatophagoides pteronyssinus patch test-positive (+ or ++) or negative (- or -? reactions; based on the ICDRG guidelines). AD and high (score 3 to 4) or low (score 0 to 2; not further defined) mite-specific IgE (both Dermatophagoides farinae and pteronyssinus): PT-positive with a low or no mite-specific lgE, PT-negative with an elevated mite-specific IgE, PT-positive with a high mite- specific IgE, PT-negative with a low level of mite-specific IgE. | PT-positive with low/no lgE: 19 (14.6)  PT-negative with elevated IgE: 42 (32.3)  PT-positive with high IgE: 32 (24.6)  PT-negative with low IgE: 37 (28.5) | Type of skin lesion (erythema, edema, vesicles, excoriations, crusts, scaling, and lichenification) and extent of affected areas | No formal statistical test | N |
|  | **Results of the analysis →** | Marked differences in the clinical morphology of the lesions were observed in the four different groups. In the group that was PT-positive with a low or no mite-specific lgE, skin lesions typically were discrete, papulovesicular, or crusted plaques, that were localized on the eyelids, neck, nipples, wrists, ankles, and the flexor areas of the elbows and knees. Diffuse, erythematous skin lesions were rare. In the group that was PT-negative with an elevated mite-specific IgE, skin lesions were ill defined, edematous or erythematous, and extended diffusely on the body surface. In patients with an elevated level of mite-specific lgE and positive PT, 89% manifested facial lesions. Clinical features in this group were lichenified and erythematous lesions which were frequently associated with papulovesicular lesions. Patients with a low level of mite-specific IgE and negative PT, showed no distinct morphological features. | | | | | | | | | | | |
| Julian-Gonzalez 2012 | Cross-sectional | NR | Hos | Mexico | Region of the Americas | 131 | (0-18) | 67 (51) M / 64 (49) F | AD in children during infancy (0-2), preschool to school age (2-12) and adolescence (12-18) | Infants: n=6 (35), preschool or school-aged children: n=49 (37), adolescent: n=39 (28) | Morphological characteristics, including sites affected and morphology of lesions (follicular, nummular, erythroderma, prurigo type, papular-lichenoid) | Chi-squared test | N |
|  | **Results of the analysis →** | Absolute frequency of some clinical variants of AD showed significant differences between age groups, specifically genital dermatitis (p= 0.01) and papular-lichenoid variant (p=0.02) were more common in infants; atopic feet (p=0.002), prurigo-like (p=0.04), nummular pattern (0.02) and erythroderma (p=0.02) in preschool and school-aged children; and eyelid eczema (p>0.99) and nipple dermatitis (0>0.99) in adolescents. No significant differences were found for follicular pattern, infra- auricular fissures, cheilitis, retro-auricular fissures, fingertip eczema and infranasal fissures. | | | | | | | | | | | |
| Kim 2017 | Cross-sectional | NR | Hos | Korea | Western Pacific Region | 81 | 15.25 ± 8.07 (7-23) | 46 (56.8) M / 35 (43.2) F | Genotype of patients based on FLG single nucleotide variations (SNVs) | NR | Phenotypic data, especially AD-associated minor clinical features, including xerosis, pityriasis alba, cheilitis, tendency towards non-specific hand or foot dermatitis, scalp scale, perifollicular accentuation, KP, PH, and ichthyosis | Chi-squared test | Y (100) |
|  | **Results of the analysis →** | The allele A of rs76413899 was only identified in patients with cheilitis (16.3%) (P = 0.033). Non-specific hand or foot dermatitis was associated with various FLG-SNVs including P478S (rs11584340). After Bonferroni correction, no significant associations were found between any single FLG-SNVs and AD-associated minor clinical features. Haplotype blocks were correlated with non-specific hand or foot dermatitis and scalp scale (p=0.0052-0.0384). | | | | | | | | | | | |
| Kulthanan (also in disease trajectory group) 2011 | Cross-sectional | 2006-2008 | Hos | Thailand | South-East Asian Region | 56 | 34.1 ± 11.7 (18-72) | 10 (15) M / 46 (82) F | Extrinsic and intrinsic types of adult-onset AD in Thai patients, based on total serum IgE levels <200 kU/L, no specific serum IgE antibodies and/or negative SPT for common aeroallergens and/or food allergens, and the absence of associated respiratory atopic diseases = intrinsic form: iAD or atopiform dermatitis; other patients = extrinsic form; eAD. | Adult-onset: 100%  eAD: n=49 (87.5), iAD: n=7 (12.5) | “Clinical and diagnostic features”, including the Hanifin and Rajka criteria (including morphological features) | Pearson’s chi–squared test, Fisher exact test, unpaired t-test, Mann-Whitney U test | N |
|  | **Results of the analysis →** | eAD more commonly had typical lichenified/exudative eczematous lesions, especially on the antecubital and popliteal (flexural) areas, when compared with patients with iAD. Nummular and follicular lesions were more commonly seen in iAD group than the eAD group. Lesions on antecubital and popliteal area were detected more commonly in eAD (62.8% and 30.2% respectively). The most common area of involvement in iAD was non-flexural area (71.4%) especially trunk and legs (57.1% each), followed by flexural area (57.1%) and extensor area (42.9%). eAD frequently had flexural lichenification when compared with iAD (p=0.002), and had a statistically significant higher percentage of personal history of atopy (p<0.001), especially allergic rhinitis (p=0.001), than iAD. Ichthyosis, cutaneous infection, non-specific hand and foot dermatitis, Dennie-Morgan infraorbital fold, orbital darkening, facial pallor, anterior neck folds, itch when sweating, course influenced by environment or emotional factors, intolerance to wool and lipid solvent or any coarse fabric were commonly seen in iAD when compared with eAD (although not significant). | | | | | | | | | | | |
| Lammintausta (in morph charac, morph phen en severity group) 1993 | Cohort | 1983-1989 | Hos | Finland | European Region | 1008 | (19-41) | NR | Four subgroups according to disease severity and other atopic symptoms: 1: severe AD, history of periods of hospitalization (n=241); 2: Moderate AD, five or more ambulatory visits to the department of dermatology (n=399); 3: Mild dermatitis, one to four outpatient clinic visits (n=161); 4: Patients with allergic rhinitis, allergic conjunctivitis or asthma, but no dermatitis in childhood (n=207).  Hand dermatitis | 1: n=241 (24); 2: n=399 (40); 3: n=161 (16); 4: n=207 (21). | Occupational exposures; the occurrence, extent and distribution of papules, vesicles, erythema and lichenification | Chi-squared test | Y (626) |
|  | **Results of the analysis →** | In moderate-severe AD patients, facial dermatitis was seen in 63% and hand dermatitis in 54%, flexural dermatitis in 50% and in 52% dermatitis occurred on the body. Hand dermatitis showed an evident correlation with occupational exposure. Of the patients exposed to wet work or mechanically skin—irritating factors for 2h or more daily, 90% had hand dermatitis, compared to 50% of those who had little or no corresponding exposure. Dermatitis in other locations was not associated with occupational exposure. | | | | | | | | | | | |
| Lee 2000 | Cross-sectional | 1993-1994 | Hos | Korea | Western Pacific Region | 130 | (2-42) | 62 (48) M / 68 (52) F | Childhood (2-12 years) and adolescent-adult (>12 years) AD in South Korea | Childhood: n=48 (37), adolescent-adult: n=82 (63). South korean: 100% | 39 morphological characteristics: 32 conventional and 7 minor features based on clinical experience of the authors. | Chi-squared test | Y (198) |
|  | **Results of the analysis →** | All features apart from geographic tongue and pitted nails were more likely to occur in South Korean AD patients (p<0.001 to <0.05), compared to controls. Nine features were of diagnostic importance only in the adolescent-adult AD group (cheilitis, nonspecific hand-foot dermatitis, IV, chronic dermatophytosis, pompholyx, nipple eczema, itchy hyperkeratotic papules on the dorsum of the hands, knuckle dermatitis of the hands, and prurigo nodularis), and three features were characteristic only in the childhood AD group (superficial folliculitis, fissured heel, and palmar erythema). | | | | | | | | | | | |
| Lee 2018 | Cross-sectional | 2015-2017 | Hos | Korea | Western Pacific Region | 281 | AD only: 13.6±13.9; AD with ACD: 27.4±18.2 | 166 (59) M / 115 (41) F | Concurrent allergic contact dermatitis (ACD) in AD patients and AD only based on patch testing | AD and ACD: n=71 (25), AD only: n=210 (75) | Presence of impetigo, prurigo nodularis, nummular eczema, and eczema herpeticum | Chi-square test, Fisher’s exact test; univariate and multivariate logistic regression analyses | N |
|  | **Results of the analysis →** | Presence of prurigo nodularis was associated with concurrent ACD in AD patients (OR: 2.69; 95% CI: 1.27-5.63; p = 0.009), but not after adjusting for age and sex (aOR: 1.67 (0.70-3.87); p=0.235). For the other characteristics no significant differences were found (p=0.103-0.788). | | | | | | | | | | | |
| Li 2018 | Cross-sectional | NR | Hos | China | Western Pacific Region | 487 | 7.1 ± 9.4 | 247 (50.7) M / 240 (49.3) F | Genotypes based on Aryl hydrocarbon receptor (AHR) gene polymorphisms (rs10249788 and rs2066853) in Han Chinese patients with AD | n=186 included in clinical (dry skin phenotype) analyses | “Dry skin phenotypes”: global clinical dry skin score was determined as the sum of the scores for four dry skin phenotypes (0–4): xerosis, IV, PH and KP. Each phenotype was scored as 0 (not present) or 1 (present). | Chi-squared test, Fisher exact test, Woolf test | Y (436) |
|  | **Results of the analysis →** | Patients with AD with the rs10249788 (CT + TT) (P = 0.02, OR = 3.06, 95% CI 1.12–8.30) or rs2066853 (AG + AA) genotype (P = 0.01, OR = 2.68, 95% CI 1.24–5.78) were more likely to have severe dry skin scores (global clinical dry skin scores ≥ 2). Further stratification analysis, the AHR rs2066853 (AG + AA) and rs10249788 (CT + TT) genotypes could predict a higher risk of severe dry skin phenotypes in the male, early-onset (before age of 2 years) and allergic rhinitis subgroups. Furthermore, the combined rs10249788 (CT + TT) and rs2066853 (AG + AA) genotypes led to a higher risk for severe dry skin in patients with AD (P < 0.01, OR = 3.58, 95% CI 1.26–10.20). | | | | | | | | | | | |
| Li 2020 | Cross-sectional | 2014 | Hos | China | Western Pacific Region | 6,208 | Bacterial infection: mean ± SD: 38:7 ± 17:5; no bacterial infection: mean ± SD: 36.9 ± 18.8. | 3183 (51) M / 3025 (49) F | Subgroups of AD based presence or absence of clinically suspected bacterial infection | Bacterial group: n=2918 (47); nonbacterial group: n=3290 (53) | Lesional distribution : cubital fossa, popliteal fossa, side neck, face, ear, shoulder and back, axillary, abdomen, foot, and pudendum. | Student’s t-test, chi-squared test; multivariate analysis, stepwise logistic regression | N |
|  | **Results of the analysis →** | In univariate analyses, significantly higher frequency of lesion distribution in the cubital fossa, popliteal fossa, side neck, ear, nape, shoulder and back, axillary, chest, abdomen, waist, upper limb, thigh, shank, foot, buttock, pudendum, and crissum was observed in patients with bacterial infection than those without bacterial infection (P < 0.04). On the contrary, significantly lower frequency of lesion distribution in the head and face was observed in patients with bacterial infection than those without bacterial infection (P < 0.02). No significant difference in lesion distribution in the eyelid and hand was found between the two groups (P > 0.42). In multivariate analyses, lesion distribution in the cubital fossa, popliteal fossa, ear, shoulder and back, axillary, foot, and pudendum was positively associated with bacterial infection (all OR > 1.0, P < 0.05). In contrast, the face and abdomen were reversely associated with bacterial infection (OR < 1.0, P < 0:005). No significant differences were found for side neck in the multivariate analysis (p=0.06). | | | | | | | | | | | |
| Majeed Al-Razzuqi 2011 | Cross-sectional | 2007-2008 | Hos | Iraq | Eastern Mediterranean Region | 36 | 9.66 ± 0.9 (8-11) | 21 (58) M / 15 (42) F | Pediatric patients with AD: slow and rapid acetylators | Slow acetylating status: n=26 (72), rapid acetylating status: n=10 (28) | Distribution eczematous lesions | Chi-squared test | Y (42) |
|  | **Results of the analysis →** | In the AD patients, eczematous lesions which presented in the limbs (elbows, knees, hands and feet) were found mostly in slow acetylators who represented 84.6% (22 out of 26 patients), whereas lesions that presented in the face and neck were found mainly in rapid acetylators who represented 60.0% (6 out of 10 patients) (p=0.029). | | | | | | | | | | | |
| Megna (also in trajectory group) 2017 | Cross-sectional | 2015-2016 | Hos | Italy | European Region | 253 | Persistent AD: 31.9 ± 11.6, adult-onset AD: 36.5 ± 12.4 | 140 (55) M / 113 (45) F | Persistent vs adult-onset AD in adults (persistent: onset < 18 years of age; adult-onset: onset ≥18 years of age; disease duration ≥6 months) | Persistent: n=151 (59.7), adult-onset: n=102 (40.3) | Clinical features, including involved body sites, morphology, past and present medical history, previous and ongoing AD treatments. | Fisher’s exact test | N |
|  | **Results of the analysis →** | Subgroup analysis between persistent vs adult-onset AD patients showed significant results only regarding AD severity (severe disease was more common in persistent group, p<0.05, 8/102, 7.8 vs 28/151, 18.5%), itch intensity (higher in adult-onset disease, 6.2 ± 2.3 vs 4.6 ± 2.5; p<0.05), and comorbidities (hypertension was more frequent in adult-onset group, 4/151, 2.6 vs 14/102, 13.7%; p<0.01). Significant higher subjects mean age was registered in AD adult-onset group respect to persistent AD one (36.5 ± 12.4 years vs 31.9 ± 11.6, p<0.05). No other significant differences were found between the groups.  As regards morphology of AD lesions, no differences were found: erythemato-desquamative pattern was the most common clinical presentation in both groups (118/151, 78.1% in persistent AD group and 70/102, 68.6% in adult-onset AD), followed by lichenified pattern (21/151, 13.9 vs 20/102, 19.6%). Exudative pattern was registered as the less frequently observed being reported in only 3.3% (5/152) and 3.9% (4/102) of persistent and adult-onset disease group, respectively. There were not any significant differences between persistent vs adult-onset AD except for narrow band (NB)-UVB which was more common in persistent AD past medical history (24/151, 15.9 vs 6/102, 5.9%, p<0.05). No statistically significant differences were found regarding AD lesion localization between persistent and adult-onset AD group. | | | | | | | | | | | |
| Meng 2014 | Cross-sectional | NR | Hos | China | Western Pacific Region | 1080 | 5.14 ± 6.42 (0.5-58) | 629 (58) M / 451 (42) F | AD patients with FLG gene mutation c.3321delA in the  Chinese Han Population | Genotype: AA: n=949 (88), Aa: n=118 (11), aa: n=13 (1) | “AD clinical phenotypes”: xerosis, PH, white dermatographism, KP, orbital darkening | Chi-squared test, Fisher’s exact test, ANOVA, nonparametric tests, logistic regression | Y (908) |
|  | **Results of the analysis →** | The c.3321delA allele frequency distribution is significantly associated with concomitant skin xerosis (P = 1.68E-03, OR = 2.13,95%CI = 1.32–3.46), PH (P = 3.64E-17, OR = 4.0,95%CI = 2.86–5.70), white dermatographism (P = 4.25E-03, OR = 1.82,95%CI = 1.22–2.71), IV (P = 2.17E-02, OR= 1.63, 95%CI = 1.07–2.49), KP (P =1.72E-02, OR= 1.70, 95%CI= 1.09–2.64), orbital darkening (P =3.62E-01, OR= 0.73, 95%CI= 0.36–1.45). | | | | | | | | | | | |
| Nettis (also in trajectory group) 2020 | Cross-sectional | 2018-2020 | Hos | Italy | European Region | 550 | Median: 38.0 (IQR: 27.0) | 308 (56.0) M / 242 (44.0) F | Childhood-onset AD, adult-onset AD | Childhood-onset AD: n=348 (63.3), adult-onset AD: n= 202 (36.7) | Clinical characteristics: lichenified/ exudative flexural dermatitis alone and associated with portrait dermatitis, nummular eczema–like phenotype, PN-like pattern | Fisher exact test | N |
|  | **Results of the analysis →** | Lichenified/ exudative flexural dermatitis alone and associated with portrait dermatitis was more common in childhood-onset AD than in adult-onset AD (191/348 [54.9%] vs 76/202 [37.6%], P<0.01). Nummular eczema–like phenotype and PN-like pattern were associated with adult-onset, when compared to childhood-onset AD (15/202 [7.4%] vs 6/348 [1.7%], P<0.01 respectively). No statistically significant differences were found regarding the other phenotypes between childhood-onset AD and adult-onset AD. | | | | | | | | | | | |
| On 2017 | Cross-sectional | NR | NR | Korea | Western Pacific Region | 70 | 19.3 (0–63) | 48 (68.6) M / 22 (31.4) F | Korean AD patients with and without FLG mutations (R501X, 3321delA, S1695X, Q1701X, Q1745X, Y1767X, Q1790X, S2554X, S2889X, S3296X, 3222del4, S1515X, Q2417X, and K4022X) | Patients with FLG mutations: n=11 (16), patients without FLG mutations: n=59 (84) | PH | Fisher’s exact test | N |
|  | **Results of the analysis →** | In the AD cohort, 24.3% of patients had PH. PH was present in eight patients (72.7%) with AD and FLG mutations. FLG mutations were significantly associated with PH (p<0.001). | | | | | | | | | | | |
| Reefer 2007 | Cross-sectional | NR | Hos | U.S.A. | Region of the Americas | 85 | High IgE: 34 ± 12. Low IgE group, divided into atopic and non atopic eczema groups: 29 ± 8.7, 33 ± 10 | 21 (25) M / 64 (75) F | AD patients with increased total IgE levels (>150 IU/mL); patients with IgE levels of less than 150 IU/mL, stratified into atopic eczema (AE) and non atopic eczema (NAE) according to sensitivity or no sensitivity to 11 common food allergens and aeroallergens. | High IgE: n=36 (42). Low IgE: AE: n=22 (26), NAE: n=27 (32) | Skin lesion distribution: head/neck, trunk, limbs, hands only | Chi-squared test, Fisher exact test | Y (15) |
|  | **Results of the analysis →** | A higher prevalence of lesions affecting only the hands was observed among the 2 groups with low total IgE levels (AE<150: 14%, NAE<150:11%), compared to the high total IgE level group (0%, p=0.05 for comparison with AE<150 group), whereas lesions affecting the head and neck were least common within the AE <150 group (18%), compared to the NAE<150 group: 48% (p=0.042) and AE>150 group: 53%). | | | | | | | | | | | |
| Silverberg (also in trajectory group) 2018 | Cross-sectional | 2014-2016 | Hos | U.S.A. | Region of the Americas | 356 | 42.8 ± 16.7 (18-93) | 126 (35.4) M / 230 (64.6) F | Adult-onset (≥ 18 years) vs childhood-onset adults with AD | Adult-onset: n=149 (41.9), Childhood-onset: n=207 (58.1) | Surveys included questions about sociodemographics, birthplace, age of moving to the United States for foreign-born Americans. Medical history and skin examination (including Hanifin and Rajka major and minor criteria, EASI, SCORAD) | Chi-squared test, Fisher exact tests, Mann-Whitney U test, latent class analysis, multivariate logistic regression | N |
|  | **Results of the analysis →** | Adult- versus childhood-onset AD was associated with birthplace outside the United States (22.5% vs 11.5%; X2, P = .0008), but not sex, race/ethnicity, current smoking status, or alcohol consumption (P ≥ .11); and decreased personal history of asthma, hay fever, and food allergy and family history of AD, asthma and food allergy (P ≤ .0001 for all). There was no significant difference in the EASI, SCORAD, body surface area, numeric rating scale for itch and sleeplessness, or Patient-Oriented Eczema Measure between adult- and childhood-onset AD (Mann-Whitney U test, P ≥ .10). Adult-onset AD compared with childhood-onset AD was associated with significantly higher rates of nummular eczema lesions (p= .0097), but lower rates of 11 of 18 signs and symptoms of AD, including dermatitis affecting anterior neck fold, scalp, face, eyelids and conjunctivitis, Dennie-Morgan folds, hands or feet, nipples, cheilitis, pityriasis alba, KP/PH/ichthyosis, clinical course worsened by emotional or environmental factors, pruritus when sweating, and tendency toward cutaneous infections (p<.005). Furthermore, patients with adult- versus childhood-onset AD had a significantly lower number of combined AD signs and symptoms (median [IQR], 6 [2-8] vs 10 [6-13]; P< .0001). Latent class analysis identified 3 classes: (1) high probability of flexural dermatitis and xerosis with intermediate to high probabilities of head, neck, and hand dermatitis; (2) high probability of flexural dermatitis and xerosis, but low probabilities of head, neck, and hand dermatitis; and (3) lower probability of flexural dermatitis, but the highest probabilities of virtually all other signs and symptoms. Adult-onset AD was significantly associated with class 1 (adjusted OR, 5.54; 95% CI, 1.59-19.28) and class 3 (adjusted OR, 14.03; 95% CI, 2.33-85.50). | | | | | | | | | | | |
| Tanei 2015 | Cross-sectional | 2000-2014 | Hos | Japan | Western Pacific Region | 60 | 77.1 ± 8.6 (≥60) | 41 (68) M / 19 (32) F | Elderly atopic eczema, categorized into three groups: IgE-allergic type; indeterminate-allergic type; and non-IgE allergic type. IgE-allergic: higher levels of serum total IgE (>400 IU/L) and positive specific IgE antibodies to common environmental allergens. Non-IgE-allergic: normal levels of serum total IgE (≤400 IU/L) and no detectable positive responses of specific IgE. Indeterminate-allergic: positive specific IgE responses with a total IgE level ≤400 IU/L or negative specific IgE responses with a total IgE level >400 IU/L. | IgE-allergic AE: n=38 (63), indeterminate-allergic AE: n=9 (15), and non-IgE-allergic AE: n=13 (22) | Morphological characteristics: including icthyosis, history of lichenified chronic eczema in the elbow and knee folds, lichenification in the antecubital areas (localized, diffuse, around the folds) | Mann-Whitney U test, Kruskal-Wallis test, Fisher’s exact test | N |
|  | **Results of the analysis →** | The incidence of ichthyosis is significantly lower in IgE-allergic AE as compared with indeterminate-allergic and non-IgE-allergic AE (p<0.05). Although various forms of skin manifestations were observed in elderly patients with AE, no significant difference was observed in the three types of elderly AE. On aggregate, eczematous dermatitis presented in 65.0% of cases involving the face and neck, 91.7% involving the trunk, 95.0% involving the upper extremities, and 83.3% involving the lower extremities in elderly AE patients. Hertoghe’s sign was recognized in 30% of elderly AE patients. Prurigo-forming papules and/or nodules on the trunk and extremities were observed in approximately 27% of patients. Exudative inflammatory erythema on the trunk and eczematous erythroderma were seen in 21.7% and 23.3% of patients, respectively. In regard to lichenified eczema (lichenification) in the extremities, lichenification was observed in 73.3% of extensor sites of the upper extremities and/or wrist, and 51.7% of extensor sites of the lower extremities. Lichenification in the elbow and knee folds showed relatively low positive rates as 23.3% (localized form, 10.0%; diffuse form, 13.3%) of the elbow folds and 18.3% (localized form, 8.3%; diffuse form, 10.0%) of the knee folds. Although 76.7% and 81.7% of elderly AE patients showed no lichenification in the elbow or knee folds, respectively, the reverse sign of lichenification around the folds was observed in 40.0% of patients in antecubital areas and 11.7% of patients in popliteal areas.10% (IgE-allergic AE, 2.6%; non-IgE-allergic AE, 38.5%) of elderly AE patients reported a history of lichenified chronic eczema in the elbow and knee folds when they lacked lichenification in those folds at the time of medical examination. Lichenification in the antecubital areas in the three types of the elderly AE patients was as follows: localized, 5.3%; diffuse, 18.4%; around the folds, 39.5% in IgE-allergic AE, localized, 11.1%; diffuse,11.1%; around the folds, 33.3% in indeterminate-allergic AE, and localized, 23.1%; diffuse, none; and around the folds, 46.2% in non-IgE-allergic AE. | | | | | | | | | | | |
| Thyssen 2010 | Cross-sectional | 2006-2008 | Pop | Denmark | European Region | 322 | (total population: 18-69) | NR | FLG genotyping for R501X and 2282del4 null mutations in Danish population: four different combinations of AD and FLG mutation status: (‘group 1’ had normal FLG mutation status and absence of AD; ‘group 2’ had presence of FLG null mutations and absence of AD); ‘group 3’ had normal FLG mutation status and presence of AD; ‘group 4’ had presence of FLG null mutations as well as AD. | Group 3: n=266 (83), group 4: n=56 (17) | Hand eczema (ever and in the past 12 months) | Chi-squared trend test, logistic regression model, long-likelihood ratio test | Y (2878) |
|  | **Results of the analysis →** | Participants with combined presence of AD and FLG null mutation status had a significantly higher prevalence of hand eczema ever (62.5%, 35/56) and hand eczema within past 12 months (71.4%, 25/35) compared with subjects with normal FLG status and absence of AD (11.8%, 500/2665 and 44.1%, 220/499, respectively). We found positive associations between hand eczema within the past 12 months and FLG null mutation status in participants with a history of AD (OR 2.98; 95% CI 1.27–7Æ01; P = 0.012), but not in subjects without AD (OR 0.82; 95% CI 0.41–1.67; P = 0.60) (adjusted for sex, age and contact allergy in logistic regression analyses). | | | | | | | | | | | |
| Von Kobyletzki (also in trajectory group) 2014 | Cohort | 2000, 2005 | Pop | Sweden | European Region | 829 | (1-8) | 419 (50.5) M / 410(49.5) F | AD with onset in or before the third year of life with remission in childhood (within 5 years of baseline/by age 6-8) | N=484 (52) reported no eczema during the 12 months preceding their follow-up interview in 2005;  n=345 participants without remission | Background, health, lifestyle, and environmental variables including: parental history, problems paying bills, house location, flexural eczema, awake at night, food allergy and rhinitis | Multivarablelogistic model | N |
|  | **Results of the analysis →** | Independent factors at baseline predicting remission were: milder eczema (adjusted odds ratio (aOR), 1.43; 95% confidence interval (95% CI) 1.16–1.77); later onset of eczema (aOR 1.40; 95% CI 1.08–1.80); non-flexural eczema (aOR 2.57; 95% CI 1.62–4.09); no food allergy (aOR 1.51; 95% CI 1.11–2.04), and rural living (aOR 1.48; 95% CI 1.07–2.05). Other factors were also associated with remission, such as having no or only one parent with a history of allergic disease. Factors related to the birth environment (birth order, sex, problems paying bills, or parental smoking) or family lifestyle (bedrooms with PVC flooring material, home construction, breastfeeding, antibiotic consumption and kindergarten attendance) were not associated with remission and neither was birth-weight. | | | | | | | | | | | |
| Yazganoglu (also in disease trajectory group) 2011 | Cross-sectional | 1996-2004 | Hos | Turkey | European Region | 321 | Median: 7; IQR: 12 (6 mo -21 y) | 175 (55) M / 146 (45) F | Turkish AD patients with onset before the age of 18 year (at infancy (1 month-2 years), at childhood (2-10 years), and at adolescence (10-18 years)) | 100% | Morphology: typical lichenified/exudative eczematous pattern or nummular, papular, prurigo-like, follicular, seborrheic dermatitis-like, mixed patterns, and erythroderma. Localization: Face/symmetrical cheek involvement, flexural sites (antecubital/popliteal/neck/wrist/ankle) with or without involvement of other parts, nonflexural involvement of the extremities, seborrheic areas (scalp, retroauricular region), anogenital/diaper area, and generalized involvement. Site of onset. | No formal statistical tests | N |
|  | **Results of the analysis →** | The main involved sites were flexures in 239 patients (74.5%), being most frequently antecubital/popliteal flexural areas in 217 patients (67.6%). Face n=189 (58.9%). Extremity n=174 (54.2%). Trunk n=151 (47.0%). Hand n=97 (30.2%; in 26.5% of infant, 31.6% of childhood and 41.3% of adolescent patients). Seborrheic areas (scalp/retroauricular) n=83 (25.9%). Foot n=36 (11.2%), Nipple n=17 (5.3%). Anogenital n=14 (4.4%; the majority being infants (64%)). Generalized n=17 (5.3%). Erythoderma n=2 (0.6%). 49.5% (n=159) of patients had nontypical localization of AD, the majority being infants or children who had flexural involvement rather than the typical cheek or extremity lesions. Lichenified/exudative eczematous pattern was the most frequent morphologic type (45.5%), followed by a mixed type (44.9%) comprising combinations of mainly lichenified/eczematous pattern with other pattens of dermatitis, mainly nummular pattern. A total of 175 patients (54.5%) had the nontypical morphologic variants such as nummular (21.2%), seborrheic dermatitis-like (21.2%), popular (18.7%), follicular (8.7%), and prurigo-like (4.6%) patterns mainly in combination with the lichenified/exsudative pattern. Among them, 32 patients (9.6%) had the following isolated morphologic variants: 17 patients (5.3%) had nummular pattern alone, whereas 7 patients (2.2%) had popular, 4 patients (1.2%) prurigo-like, and 3 patients (0.9%) follicular pattern alone. Regarding site of onset, flexural (mainly antecubital/popliteal) onset of AD was seen in the majority of cases (n = 130, 40.5%), followed by face (symmetrical cheek involvement) (n = 95, 29.6%), and extremity extensors (n = 33, 10.3%). | | | | | | | | | | | |

Articles in alphabetical order. Column methodological approach: presents the methodological approach for investigating associations, unless further specified (i.e. in case of data-driven approach to identify phenotypes). AD, atopic dermatitis; Hos, hospital-based; Pop, population-based; No., number of participants with (atopic) dermatitis; NR, not reported; SD, standard deviation; Y, yes; N, no; U, unclear. ICDRG: International Contact Dermatitis Research Group. TEWL, transepidermal water loss.
