# Supplementary material for: Classifying atopic dermatitis: a systematic review of phenotypes and associated characteristics
Source: J Eur Acad Dermatol Venereol. 2022 Feb 25;36(6):807–19. doi: 10.1111/jdv.18008 (PMC9307020; doi:10.1111/jdv.18008)
Supplement: Supplementary file 2 — Table S2. Qualitative outcomes by the JBI critical appraisal checklists. [file JDV-36-807-s003.zip › jdv18008-sup-0006-Table2a.docx]

**Supplementary Table 2a. Qualitative outcomes by appropriate JBI Critical Appraisal Checklist on phenotypes based on disease severity**

**Cross-sectional studies**

| Study | Year | Item 1 | Item 2 | Item 3 | Item 4 | Item 5 | Item 6 | Item 7 | Item 8 |
| --- | --- | --- | --- | --- | --- | --- | --- | --- | --- |
| Addor | 2012 | **Y** | **Y** | **Y** | **Y** | **N** | **N** | **Y** | **Y** |
| Arima | 2005 | **U** | **N** | **Y** | **U** | **N** | **N** | **U** | **Y** |
| Batmaz | 2018 | **Y** | **Y** | **Y** | **Y** | **N** | **N** | **Y** | **Y** |
| Benito | 2016 | **U** | **N** | **Y** | **N** | **U** | **N** | **Y** | **NA** |
| Bergallo | 2020 | **Y** | **N** | **Y** | **Y** | **Y** | **Y** | **U** | **Y** |
| Bradley | 2002 | **Y** | **Y** | **Y** | **U** | **U** | **U** | **Y** | **Y** |
| Brandwein | 2018 | **Y** | **N** | **Y** | **U** | **N** | **N** | **U** | **U** |
| Brown | 2008 | **Y** | **N** | **Y** | **Y** | **Y** | **Y** | **N** | **Y** |
| Brunner | 2018 | **Y** | **N** | **Y** | **U** | **Y** | **Y** | **U** | **Y** |
| Chan | 2018 | **Y** | **N** | **Y** | **U** | **Y** | **Y** | **U** | **Y** |
| Clausen | 2017 | **Y** | **Y** | **Y** | **Y** | **N** | **N** | **Y** | **Y** |
| Czarnowicki | 2015 | **U** | **N** | **Y** | **N** | **U** | **U** | **U** | **Y** |
| de Bruin-Weller | 2020 | **Y** | **Y** | **Y** | **N** | **N** | **N** | **Y** | **Y** |
| deOliveiraTitz | 2016 | **U** | **N** | **Y** | **U** | **N** | **N** | **U** | **Y** |
| Dworzak | 1999 | **U** | **N** | **Y** | **Y** | **Y** | **Y** | **U** | **Y** |
| Dyjack | 2018 | **U** | **N** | **Y** | **U** | **N** | **N** | **Y** | **Y** |
| Ekelund | 2008 | **Y** | **Y** | **Y** | **Y** | **Y** | **U** | **U** | **Y** |
| Ercan | 2013 | **Y** | **Y** | **Y** | **Y** | **Y** | **Y** | **Y** | **Y** |
| Flohr | 2010 | **Y** | **N** | **Y** | **Y** | **N** | **N** | **Y** | **Y** |
| Flohr | 2014 | **Y** | **N** | **Y** | **Y** | **Y** | **Y** | **Y** | **Y** |
| Foley | 2001 | **Y** | **Y** | **U** | **U** | **N** | **N** | **U** | **U** |
| Furue | 2012 | **Y** | **Y** | **Y** | **Y** | **N** | **N** | **Y** | **Y** |
| Galli | 2015 | **Y** | **Y** | **Y** | **Y** | **N** | **N** | **Y** | **Y** |
| Galli | 2020 | **Y** | **Y** | **Y** | **Y** | **Y** | **Y** | **Y** | **Y** |
| Gayret | 2019 | **Y** | **Y** | **Y** | **Y** | **N** | **N** | **Y** | **Y** |
| Holm | 2019 | **Y** | **Y** | **Y** | **Y** | **N** | **N** | **Y** | **Y** |
| Ibrahim | 2012 | **Y** | **N** | **Y** | **Y** | **N** | **N** | **Y** | **Y** |
| Jenerowicz | 2007 | **Y** | **N** | **Y** | **Y** | **O** | **O** | **U** | **Y** |
| Johnson | 1974 | **Y** | **N** | **Y** | **U** | **Y** | **Y** | **N** | **Y** |
| Jung | 2014 | **U** | **U** | **Y** | **Y** | **N** | **N** | **Y** | **Y** |
| Kaga | 2011 | **Y** | **N** | **Y** | **Y** | **N** | **N** | **U** | **Y** |
| Kayserova | 2012 | **Y** | **N** | **Y** | **N** | **N** | **N** | **Y** | **U** |
| Kezic | 2011 | **Y** | **Y** | **Y** | **Y** | **Y** | **Y** | **Y** | **Y** |
| Kou | 2014 | **Y** | **N** | **Y** | **Y** | **Y** | **Y** | **Y** | **Y** |
| Lacy | 2009 | **Y** | **N** | **Y** | **Y** | **Y** | **Y** | **U** | **Y** |
| Laske | 2004 | **Y** | **N** | **Y** | **U** | **N** | **N** | **Y** | **Y** |
| Lee | 2018 | **Y** | **N** | **Y** | **Y** | **N** | **N** | **Y** | **Y** |
| Liu | 2020 | **Y** | **N** | **Y** | **Y** | **N** | **N** | **Y** | **Y** |
| Lopes | 2016 | **Y** | **N** | **Y** | **Y** | **N** | **N** | **Y** | **Y** |
| Martel | 2016 | **Y** | **N** | **Y** | **Y** | **N** | **N** | **Y** | **Y** |
| Miadonna | 1985 | **Y** | **N** | **Y** | **U** | **N** | **N** | **Y** | **U** |
| Mittermann | 2016 | **Y** | **N** | **Y** | **Y** | **N** | **N** | **Y** | **Y** |
| Mocsai | 2014 | **U** | **N** | **Y** | **U** | **N** | **N** | **Y** | **Y** |
| Montero-Vilchez | 2021 | **Y** | **Y** | **Y** | **N** | **Y** | **Y** | **Y** | **Y** |
| Nousbeck | 2020 | **U** | **N** | **Y** | **U** | **N** | **N** | **U** | **Y** |
| Oh | 2009 | **Y** | **N** | **Y** | **Y** | **Y** | **Y** | **Y** | **Y** |
| Okano-Mitani | 1996 | **U** | **N** | **Y** | **Y** | **N** | **N** | **Y** | **Y** |
| Okawa | 2018 | **Y** | **N** | **Y** | **Y** | **N** | **N** | **Y** | **Y** |
| Ong | 2008 | **Y** | **N** | **Y** | **Y** | **Y** | **Y** | **Y** | **Y** |
| O'Regan | 2010 | **Y** | **N** | **Y** | **Y** | **Y** | **Y** | **Y** | **Y** |
| Pavel | 2021 | **Y** | **N** | **Y** | **U** | **N** | **N** | **U** | **Y** |
| Pigors | 2018 | **Y** | **N** | **Y** | **Y** | **N** | **N** | **Y** | **Y** |
| Rafatpanah | 2003 | **Y** | **N** | **Y** | **Y** | **Y** | **Y** | **Y** | **Y** |
| Sakurai | 2002 | **Y** | **Y** | **Y** | **Y** | **N** | **N** | **Y** | **Y** |
| Salpietro | 2011 | **Y** | **Y** | **Y** | **Y** | **N** | **N** | **Y** | **Y** |
| Sandilands | 2007 | **Y** | **N** | **Y** | **Y** | **N** | **N** | **Y** | **Y** |
| Savolainen | 1993 | **Y** | **Y** | **Y** | **Y** | **N** | **N** | **Y** | **Y** |
| SchultzLarsen | 1985 | **Y** | **N** | **Y** | **U** | **N** | **N** | **U** | **Y** |
| Seneviratne | 2006 | **Y** | **N** | **Y** | **Y** | **N** | **N** | **Y** | **Y** |
| Shen | 2018 | **Y** | **Y** | **Y** | **Y** | **Y** | **Y** | **Y** | **Y** |
| Silva | 2010 | **Y** | **Y** | **Y** | **Y** | **Y** | **Y** | **N** | **Y** |
| Simspon | 2018 | **Y** | **Y** | **Y** | **Y** | **N** | **N** | **Y** | **Y** |
| Sugawara | 2012 | **U** | **Y** | **Y** | **U** | **N** | **N** | **U** | **Y** |
| Takigawa | 1991 | **Y** | **N** | **Y** | **Y** | **N** | **N** | **Y** | **Y** |
| Thijs | 2017 | **Y** | **N** | **Y** | **Y** | **Y** | **Y** | **Y** | **Y** |
| Thompson | 1983 | **N** | **N** | **Y** | **U** | **N** | **N** | **U** | **Y** |
| Toncic | 2020 | **Y** | **Y** | **Y** | **Y** | **N** | **N** | **U** | **Y** |
| Uehara | 1989 | **U** | **N** | **Y** | **U** | **N** | **N** | **U** | **NA** |
| Ungar | 2020 | **Y** | **N** | **Y** | **U** | **Y** | **Y** | **Y** | **Y** |
| Valenzuela | 2020 | **Y** | **Y** | **Y** | **N** | **Y** | **Y** | **Y** | **Y** |
| Verzeaux | 2018 | **Y** | **N** | **Y** | **N** | **N** | **N** | **N** | **Y** |
| Wehrmann | 1990 | **U** | **N** | **Y** | **N** | **N** | **N** | **Y** | **Y** |
| Wehrmann | 1989 | **U** | **N** | **Y** | **Y** | **N** | **N** | **N** | **Y** |
| Wang | 2020 | **U** | **N** | **Y** | **U** | **N** | **N** | **U** | **Y** |
| Wei | 2019 | **Y** | **N** | **Y** | **N** | **N** | **N** | **Y** | **Y** |
| Winge | 2011 | **Y** | **N** | **Y** | **Y** | **N** | **N** | **Y** | **Y** |
| Yoshikawa | 2000 | **U** | **Y** | **Y** | **Y** | **N** | **N** | **U** | **Y** |
| Zhang | 2011 | **U** | **N** | **Y** | **U** | **N** | **N** | **U** | **Y** |
| Zheng | 2019 | **Y** | **N** | **Y** | **N** | **N** | **N** | **Y** | **Y** |

**Cohort studies**

| Study | Year | Item 1 | Item 2 | Item 3 | Item 4 | Item 5 | Item 6 | Item 7 | Item 8 | Item 9 | Item 10 | Item 11 |
| --- | --- | --- | --- | --- | --- | --- | --- | --- | --- | --- | --- | --- |
| Biagini Myers | 2020 | **Y** | **Y** | **Y** | **Y** | **Y** | **NA** | **Y** | **Y** | **U** | **U** | **Y** |
| Quah | 2015 | **Y** | **Y** | **Y** | **Y** | **Y** | **NA** | **Y** | **Y** | **Y** | **Y** | **Y** |
| Semic-Jusufagic | 2007 | **Y** | **Y** | **Y** | **Y** | **Y** | **NA** | **U** | **Y** | **Y** | **Y** | **Y** |
| Lammintausta | 1993 | **Y** | **Y** | **U** | **N** | **N** | **NA** | **U** | **Y** | **N** | **N** | **Y** |
| Lowe | 2020 | **Y** | **Y** | **Y** | **Y** | **Y** | **NA** | **Y** | **Y** | **N** | **N** | **Y** |
| McPherson | 2010 | **Y** | **Y** | **Y** | **N** | **N** | **NA** | **Y** | **NA** | **Y** | **NA** | **Y** |
| Roduit | 2012 | **Y** | **Y** | **Y** | **Y** | **Y** | **NA** | **Y** | **Y** | **Y** | **N** | **Y** |
| Sanchez | 2017 | **Y** | **Y** | **Y** | **Y** | **Y** | **NA** | **Y** | **Y** | **Y** | **Y** | **Y** |
| Schonmann | 2020 | **Y** | **Y** | **Y** | **Y** | **Y** | **NA** | **Y** | **Y** | **U** | **Y** | **Y** |
| Silverwood | 2018 | **Y** | **Y** | **Y** | **Y** | **Y** | **NA** | **Y** | **Y** | **Y** | **Y** | **Y** |

**Case-control studies**

| Study | Year | Item 1 | Item 2 | Item 3 | Item 4 | Item 5 | Item 6 | Item 7 | Item 8 | Item 9 | Item 10 |
| --- | --- | --- | --- | --- | --- | --- | --- | --- | --- | --- | --- |
| Hallau | 2016 | **U** | **N** | **Y** | **Y** | **Y** | **N** | **N** | **Y** | **Y** | **Y** |
| Torsney | 1966 | **Y** | **Y** | **Y** | **U** | **U** | **N** | **N** | **U** | **Y** | **NA** |
| Uysal | 2018 | **Y** | **Y** | **Y** | **Y** | **Y** | **N** | **N** | **U** | **Y** | **Y** |

Y, yes; N, no; U, unclear, O, other: not for the analyses of interest.

Joanna Briggs Institute Critical Appraisal Checklist for Analytical Cross Sectional Studies: Risk of bias domains: item 1: Were the criteria for inclusion in the sample clearly defined?; item 2: Were the study subjects and the setting described in detail?; item 3: Was the exposure measured in a valid and reliable way?; item 4: Were objective, standard criteria used for measurement of the condition?; item 5: Were confounding factors identified?; item 6: Were strategies to deal with confounding factors stated?; item 7: Were the outcomes measured in a valid and reliable way?; item 8: Was appropriate statistical analysis used?

Joanna Briggs Institute Critical Appraisal Checklist for Cohort Studies: Risk of bias domains: item 1: Were the two groups similar and recruited from the same population?; item 2: Were the exposures measured similarly to assign people to both exposed and unexposed groups?; item 3: Was the exposure measured in a valid and reliable way?; item 4: Were confounding factors identified?; item 5: Were strategies to deal with confounding factors stated?; item 6: Were the groups/participants free of the outcome at the start of the study (or at the moment of exposure)?; item 7: Were the outcomes measured in a valid and reliable way?; item 8: Was the follow up time reported and sufficient to be long enough for outcomes to occur?; item 9: Was follow up complete, and if not, were the reasons to loss to follow up described and explored?; item 10: Were strategies to address incomplete follow up utilized?; item 11: Was appropriate statistical analysis used?

Joanna Briggs Institute Critical Appraisal Checklist for Case Control Studies: Risk of bias domains: item 1: Were the groups comparable other than the presence of disease in cases or the absence of disease in controls?; item 2: Were cases and controls matched appropriately?; item 3: Were the same criteria used for identification of cases and controls?; item 4: Was exposure measured in a standard, valid and reliable way?; item 5: Was exposure measured in the same way for cases and controls?; item 6: Were confounding factors identified?; item 7: Were strategies to deal with confounding factors stated?; item 8: Were outcomes assessed in a standard, valid and reliable way for cases and controls; item 9: Was the exposure period of interest long enough to be meaningful?, item 10: Was appropriate statistical analysis used?
