# Supplementary material for: Classifying atopic dermatitis: a systematic review of phenotypes and associated characteristics
Source: J Eur Acad Dermatol Venereol. 2022 Feb 25;36(6):807–19. doi: 10.1111/jdv.18008 (PMC9307020; doi:10.1111/jdv.18008)
Supplement: Supplementary file 2 — Table S2. Qualitative outcomes by the JBI critical appraisal checklists. [file JDV-36-807-s003.zip › jdv18008-sup-0008-Table2c.docx]

**Supplementary Table 2c. Qualitative outcomes by appropriate JBI Critical Appraisal Checklist on phenotypes based on morphological features**

**Cross-sectional studies**

| Study | Year | Item 1 | Item 2 | Item 3 | Item 4 | Item 5 | Item 6 | Item 7 | Item 8 |
| --- | --- | --- | --- | --- | --- | --- | --- | --- | --- |
| Bremmer | 2008 | **Y** | **N** | **U** | **U** | **Y** | **Y** | **Y** | **Y** |
| Chen | 2011 | **Y** | **N** | **Y** | **Y** | **Y** | **Y** | **U** | **Y** |
| Cheng | 2016 | **Y** | **N** | **Y** | **Y** | **N** | **N** | **N** | **Y** |
| Cheng | 2012 | **Y** | **N** | **Y** | **Y** | **N** | **N** | **U** | **Y** |
| Ezzedine | 2012 | **Y** | **Y** | **N** | **Y** | **Y** | **Y** | **U** | **Y** |
| Flohr | 2014 | **Y** | **N** | **Y** | **Y** | **Y** | **Y** | **Y** | **Y** |
| Guglielmo | 2020 | **Y** | **Y** | **Y** | **U** | **N** | **N** | **U** | **Y** |
| Heede | 2017 | **Y** | **Y** | **U** | **N** | **N** | **N** | **U** | **Y** |
| Kaga | 2011 | **Y** | **N** | **Y** | **Y** | **N** | **N** | **U** | **Y** |
| Kou | 2014 | **Y** | **N** | **Y** | **Y** | **Y** | **Y** | **Y** | **Y** |
| Li | 2013 | **U** | **N** | **Y** | **U** | **N** | **N** | **U** | **U** |
| Li | 2019 | **Y** | **N** | **Y** | **Y** | **N** | **N** | **N** | **Y** |
| Luukkonen | 2017 | **Y** | **Y** | **Y** | **N** | **Y** | **Y** | **U** | **Y** |
| Okawa | 2018 | **Y** | **N** | **Y** | **Y** | **N** | **N** | **Y** | **Y** |
| Ono | 2018 | **Y** | **N** | **Y** | **Y** | **N** | **N** | **N** | **Y** |
| Silverberg | 2019 | **Y** | **N** | **Y** | **Y** | **N** | **N** | **Y** | **Y** |
| Takahashi | 2013 | **Y** | **N** | **Y** | **Y** | **N** | **N** | **N** | **Y** |
| Weidinger | 2006 | **U** | **N** | **Y** | **Y** | **Y** | **Y** | **U** | **Y** |
| Yamamoto-Hanada | 2020 | **Y** | **Y** | **Y** | **Y** | **N** | **N** | **Y** | **Y** |
| Yasuda-Sekiguchi | 2020 | **Y** | **Y** | **Y** | **Y** | **N** | **N** | **U** | **Y** |
| Zhong | 2016 | **Y** | **N** | **Y** | **Y** | **N** | **N** | **N** | **Y** |

**Cohort studies**

| Study | Year | Item 1 | Item 2 | Item 3 | Item 4 | Item 5 | Item 6 | Item 7 | Item 8 | Item 9 | Item 10 | Item 11 |
| --- | --- | --- | --- | --- | --- | --- | --- | --- | --- | --- | --- | --- |
| Lammintausta | 1993 | **Y** | **Y** | **U** | **N** | **N** | **NA** | **U** | **Y** | **N** | **N** | **Y** |

Y, yes; N, no; U, unclear, O, other: not for the analyses of interest.

Joanna Briggs Institute Critical Appraisal Checklist for Analytical Cross Sectional Studies: Risk of bias domains: item 1: Were the criteria for inclusion in the sample clearly defined?; item 2: Were the study subjects and the setting described in detail?; item 3: Was the exposure measured in a valid and reliable way?; item 4: Were objective, standard criteria used for measurement of the condition?; item 5: Were confounding factors identified?; item 6: Were strategies to deal with confounding factors stated?; item 7: Were the outcomes measured in a valid and reliable way?; item 8: Was appropriate statistical analysis used?

Joanna Briggs Institute Critical Appraisal Checklist for Cohort Studies: Risk of bias domains: item 1: Were the two groups similar and recruited from the same population?; item 2: Were the exposures measured similarly to assign people to both exposed and unexposed groups?; item 3: Was the exposure measured in a valid and reliable way?; item 4: Were confounding factors identified?; item 5: Were strategies to deal with confounding factors stated?; item 6: Were the groups/participants free of the outcome at the start of the study (or at the moment of exposure)?; item 7: Were the outcomes measured in a valid and reliable way?; item 8: Was the follow up time reported and sufficient to be long enough for outcomes to occur?; item 9: Was follow up complete, and if not, were the reasons to loss to follow up described and explored?; item 10: Were strategies to address incomplete follow up utilized?; item 11: Was appropriate statistical analysis used?
