# Supplementary material for: Classifying atopic dermatitis: a systematic review of phenotypes and associated characteristics
Source: J Eur Acad Dermatol Venereol. 2022 Feb 25;36(6):807–19. doi: 10.1111/jdv.18008 (PMC9307020; doi:10.1111/jdv.18008)
Supplement: Supplementary file 2 — Table S2. Qualitative outcomes by the JBI critical appraisal checklists. [file JDV-36-807-s003.zip › jdv18008-sup-0009-Table2d.docx]

**Supplementary Table 2d. Qualitative outcomes by appropriate JBI Critical Appraisal Checklist on phenotypes based on eczema herpeticum**

**Cross-sectional studies**

| Study | Year | Item 1 | Item 2 | Item 3 | Item 4 | Item 5 | Item 6 | Item 7 | Item 8 |
| --- | --- | --- | --- | --- | --- | --- | --- | --- | --- |
| Beck | 2009 | **Y** | **N** | **U** | **N** | **N** | **N** | **Y** | **Y** |
| Bin | 2014 | **Y** | **U** | **Y** | **U** | **Y** | **Y** | **Y** | **Y** |
| Boorgula | 2019 | **Y** | **N** | **Y** | **U** | **Y** | **Y** | **Y** | **Y** |
| Broccardo | 2011 | **Y** | **N** | **Y** | **Y** | **Y** | **Y** | **U** | **Y** |
| Gao | 2015 | **Y** | **N** | **Y** | **U** | **Y** | **Y** | **Y** | **Y** |
| Gao | 2010 | **Y** | **N** | **Y** | **N** | **Y** | **Y** | **Y** | **Y** |
| Gao | 2009 | **Y** | **N** | **Y** | **N** | **N** | **N** | **Y** | **Y** |
| Hinz | 2011 | **Y** | **Y** | **Y** | **Y** | **N** | **N** | **Y** | **Y** |
| Mathias | 2013 | **Y** | **N** | **Y** | **N** | **Y** | **Y** | **Y** | **Y** |
| Narla | 2018 | **Y** | **Y** | **Y** | **N** | **Y** | **Y** | **U** | **Y** |

**Case-control studies**

| Study | Year | Item 1 | Item 2 | Item 3 | Item 4 | Item 5 | Item 6 | Item 7 | Item 8 | Item 9 | Item 10 |
| --- | --- | --- | --- | --- | --- | --- | --- | --- | --- | --- | --- |
| Takahashi | 2014 | **Y** | **U** | **Y** | **Y** | **Y** | **N** | **N** | **Y** | **Y** | **Y** |

Y, yes; N, no; U, unclear.

Joanna Briggs Institute Critical Appraisal Checklist for Analytical Cross Sectional Studies: Risk of bias domains: item 1: Were the criteria for inclusion in the sample clearly defined?; item 2: Were the study subjects and the setting described in detail?; item 3: Was the exposure measured in a valid and reliable way?; item 4: Were objective, standard criteria used for measurement of the condition?; item 5: Were confounding factors identified?; item 6: Were strategies to deal with confounding factors stated?; item 7: Were the outcomes measured in a valid and reliable way?; item 8: Was appropriate statistical analysis used?

Joanna Briggs Institute Critical Appraisal Checklist for Case Control Studies: Risk of bias domains: item 1: Were the groups comparable other than the presence of disease in cases or the absence of disease in controls?; item 2: Were cases and controls matched appropriately?; item 3: Were the same criteria used for identification of cases and controls?; item 4: Was exposure measured in a standard, valid and reliable way?; item 5: Was exposure measured in the same way for cases and controls?; item 6: Were confounding factors identified?; item 7: Were strategies to deal with confounding factors stated?; item 8: Were outcomes assessed in a standard, valid and reliable way for cases and controls; item 9: Was the exposure period of interest long enough to be meaningful?, item 10: Was appropriate statistical analysis used?
