# Supplementary material for: Classifying atopic dermatitis: a systematic review of phenotypes and associated characteristics
Source: J Eur Acad Dermatol Venereol. 2022 Feb 25;36(6):807–19. doi: 10.1111/jdv.18008 (PMC9307020; doi:10.1111/jdv.18008)
Supplement: Supplementary file 3 — Appendix S1. Changes to the original protocol. [file JDV-36-807-s004.docx]

**Supplementary material 1. Changes to the original protocol**

1. The literature search was performed as described in the published protocol.* In addition, we applied the criteria for eligibility as described in the protocol while screening the papers. Because the term phenotype is used in numerous ways in the literature, we had to define phenotype for consistency and in the context of this systematic review we have defined phenotype as a subtype or subgroup of patients with atopic dermatitis (AD). In theory, subgroups of patients can be defined based on all kinds of features, including both clinical and non-clinical features. Consequently, our literature search has resulted in 675 records potentially eligible for inclusion after title and abstract screening. Based on this it was deemed not feasible to include all potential phenotypic groupings in this study. Therefore, we were forced to make choices on which phenotypes to focus, which resulted in focusing only on the most clinically relevant phenotypes.

After title and abstract screening but before full-text screening, data extraction and analysis, we predetermined the following phenotypic groupings to be of interest for this systematic review:

1) The AD phenotype is defined by disease severity (e.g., mild, moderate-to-severe, severe);

2) The AD phenotype is defined by disease trajectory (e.g., early-onset, late-onset);

3) The AD phenotype is defined by morphological features (i.e., based on findings at physical examination (e.g. flexural eczema)); and

4) The AD phenotype is defined by (history of) eczema herpeticum.

In these four phenotypic groupings the associated characteristics (e.g., FLG mutations) are subsequently investigated per phenotype. For papers that did not define the phenotype by morphological features (see under 3), but instead first determined the phenotype (e.g., based on FLG mutations) in order to describe morphological characteristics in these subgroups, we included as a fifth phenotype:

5) The study defines the AD phenotype based on a certain feature (e.g., FLG mutations) in order to investigate morphological characteristics in these phenotypes.

Although these phenotypic groupings were not yet specified in the published protocol, we further applied the protocol as described on these groupings.

1. Secondly, in the protocol we describe that we would assess quality within and between studies using a checklist modified from the Grading of Recommendations Assessment, Development and Evaluation (GRADE) tool for clinical trials or observational studies. However, GRADE is used to assess the quality of evidence, and risk of bias is one of the factors that determines the quality of evidence. Therefore, following discussions with author M.L., an international leading GRADE researcher, we decided to refrain from using GRADE for this purpose and opt for only assessing risk of bias using the critical appraisal checklists for analytical cross sectional studies, cohort studies and case control studies as appropriate from the Joanna Briggs Institute for assessing the risk of bias within studies. As for the assessment of the quality of evidence, we were unable to pool results and we did not use GRADE to assess the quality of evidence across studies, as anticipated in the protocol and mentioned in the limitation section of the systematic review manuscript.

*Reference: Mulick AR, Allen V, Williams HC, et al. Classifying atopic dermatitis: protocol for a systematic review of subtypes (phenotypes) and associated characteristics. BMJ Open. 2018;8(9):e023097.
