# Supplementary material for: Classifying atopic dermatitis: a systematic review of phenotypes and associated characteristics
Source: J Eur Acad Dermatol Venereol. 2022 Feb 25;36(6):807–19. doi: 10.1111/jdv.18008 (PMC9307020; doi:10.1111/jdv.18008)
Supplement: Supplementary file 4 — Appendix S2. Ovid MEDLINE search strategy. [file JDV-36-807-s002.docx]

**Supplementary material 2. Ovid MEDLINE search strategy**

1. exp PHENOTYPE/
2. classification.mp.
3. sub?type.mp.
4. phenotyp*.mp.
5. taxonomy.mp.
6. disease type*.mp.
7. disease typolog*.mp.
8. stratif*.mp.
9. strata.mp.
10. or/1-9
11. exp DERMATITIS, ATOPIC/
12. exp Eczema/
13. exp Neurodermatitis/
14. eczema*.mp.
15. atopic dermatitis.mp.
16. neurodermatitis.mp.
17. besnier* prurigo.mp.
18. or/11-17
19. 10 and 18
20. (clinical conference or editorial).pt.
21. 19 not 20
22. remove duplicates from 21
