# Supplementary material for: Classifying atopic dermatitis: a systematic review of phenotypes and associated characteristics
Source: J Eur Acad Dermatol Venereol. 2022 Feb 25;36(6):807–19. doi: 10.1111/jdv.18008 (PMC9307020; doi:10.1111/jdv.18008)
Supplement: Supplementary file 5 — Appendix S3. Summary of main results per phenotype category and per category of associated characteristics. [file JDV-36-807-s001.docx]

**Supplementary material 3. Summary of main results per phenotype category and per category of associated characteristics**

***Phenotypes based on disease severity***

Skin barrier function in disease severity phenotypes

Differences in skin barrier (including TEWL, hydration and pH) were described in patients with mild AD and moderate-to-severe AD, compared to controls (Jung 2014, Toncic 2020).^1, 2^ In mild, moderate and severe AD significant differences were found for corneometry (i.e. stratum corneum hydration, where lower mean values indicate higher severity) and TEWL (higher mean values indicate higher severity) (Addor 2012).^3^ Other studies also found an association between higher TEWL and more severe disease, compared to mild disease (Flohr 2010/2014, BiaginiMyers 2020).^4-6^ Significantly elevated TEWL values and decreased stratum corneum hydration values were also detected in mild AD compared to controls (Furue 2012).^7^ TEWL in non-lesional skin of severe and moderate AD has also been shown to differ compared to controls, in contrast to non-lesional skin in patients with mild and completely healed AD (Sakurai 2002).^8^ Further subgrouping of severe AD based on presence of FLG mutations did not yield differences with respect to TEWL (Mocsai).^9^ In patients with moderate/severe AD stratum corneum hydration and elasticity was lower, in contrast to temperature and erythema which were higher in patients with mild AD (Montero-Vilchez 2021).^10^

Natural Moisturizing Factor (NMF) components, including NMF components presumably derived from sweat, were decreased in mild and moderate-severe AD compared to controls (Sugawara 2012, Kezic 2011, Toncic 2020).^2, 11, 12^ NMF values were significantly lower in FLG-associated moderate-to-severe AD compared to non-FLG-associated AD, although TEWL values remained similar regardless of FLG status (O'Regan 2010, Kezic 2011) ^12, 13^ No significant difference was found for the presence of two ceramide subclasses between mild, moderate, and severe patients (Shen 2018).^14^ Mild-to-moderate AD skin revealed a significant decrease of the lipid-to-protein ratio and bound water, compared to controls (Verzeaux 2018).^15^

Serum blood cell types and markers in disease severity phenotypes

Batmaz et al^16^ found no significant differences between mild, moderate and severe AD and controls in neutrophil-lymphocyte ratios, platelet-lymphocyte ratios and mean platelet volume, as nonspecific indicators of systemic inflammation (Batmaz 2018). In another study, the mean platelet volume was higher and platelet distribution width was lower in severe versus mild AD (Gayret 2019).^17^ In subgroups with mild, moderate and severe AD, a higher frequency of eosinophils was significantly correlated with increasing AD severity (DeOliveiraTitz 2016, Holm 2019).^18, 19^ However, in another study there were no differences in mean eosinophil numbers or mean eosinophil percentage between mild-to-moderate and severe-to-very severe AD (Jenerowicz 2007).^20^ Activity of leukotriene A4 hydrolase (an inflammatory enzyme important in allergic disease) was significantly higher in preparations of peripheral blood cells from severe AD patients than in those from moderate and mild AD and normal controls (Okano-Mitani 1996).^21^

In patients with severe AD, circulating skin-associated T cells showed significant signs of subset expansion compared to controls (Dworzak 1999).^22^ Filaggrin null mutations were shown to be associated with higher frequencies of allergen-specific CD4+ T-helper 2 cell responses in patients with moderate-severe AD (Mcpherson 2010).^23^ In moderate AD a tendency to a decreased lymphocyte transformation and a reduction of the OKT 8+ cells with an increased OKT4/OKT8 ratio was found, compared to controls (SchultzLarsen 1985).^24^ An increased OKT4+/OKT8+ ratio was also detected in three children with particularly severe atopic eczema (Miadonna 1985).^25^ Higher frequencies of allergen-specific circulating CD4+ T cells producing TNF-a, IL-4-, IL-5- and IL-13, and lower frequencies of these cells producing IL-10 were found in individuals with severe AD compared with mild AD and controls (Seneviratne 2006).^26^ Patients with severe and moderate AD were also shown to have higher percentages of FcεRII+ peripheral blood mononuclear cells, compared to mild AD and controls (Takigawa 1991).^27^ Patients with moderate-to-severe AD and highly elevated IgE levels had lower percentages of Tε cells and T**y** cells than controls (Thompson 1983).^28^ Immunophenotypic characteristics of natural killer cells appeared to differ in patients with severe AD compared to healthy controls and patients with allergic rhinitis (Wehrmann 1990).^29^

A significantly impaired capacity to secrete IFN-gamma compared to controls was found in severe AD patients (Wehrmann 1989).^30^ Serum periostin levels were also found to be positively correlated with disease severity (Kou 2014).^31^ Serum levels of squamous cell carcinoma antigen (SCCA) 2 in mild, moderate, severe and very severe subgroups AD were significantly higher than those of healthy controls, although no differences were found between the AD subgroups (Okawa 2018).^32^ Significantly elevated serum thymus and activation-regulated chemokine (TARC) levels were detected in mild AD compared to controls (Furue 2012).^7^ There was no significant difference between mild, moderate and severe AD regarding oxidative stress parameters (Uysal 2018).^33^ Mean vitamin D concentrations in patients with moderate and severe chronic eczema were not statistically different from concentrations detected in patients with mild chronic eczema (Galli 2015).^34^ The frequencies of 6 common polymorphisms in the genes encoding the vitamin D synthesizing enzyme Cyp27b1 or the inactivating enzyme Cyp24a1 were found to be different between adult patients with severe AD compared to controls (Hallau 2016).^35^ In comparison to controls, differentially expressed and dysregulated miRNAs, including ones known to be involved in inflammation, were identified in PBMCs and plasma of infants with moderate-to-severe AD (Nousbreck 2020).^36^ Increased miR-155 and FOXP3 and RORγ responses have also been suggested to provide a link to immune dysregulation in AD (Bergallo 2020).^37^

Serum Ig levels and sensitization in disease severity phenotypes

Higher IgE levels are found in severe AD, in comparison to mild and moderate AD (Johnson 1974, Addor 2012, Uehara 1989, Yoshikawa 2000, Winge 2011, Laske 2004, Holm 2019). ^3, 18, 19, 38-42^ Highest IgE levels are found for patients with severe disease and personal history of atopic respiratory disease (Uehara 1989).^39^ Specific IgE reactivity to allergens was demonstrated to be higher in patients with severe AD compared to moderate AD (Mitterman 2016).^43^ Sensitization to aeroallergens was found more frequently in severe than in mild AD (Laske 2004).^42^ Conflicting results have been found for food allergen sensitization (Laske 2004, Flohr 2014).^5, 42^ Patients with severe AD were demonstrated to have higher specific IgE to Der p and Der f than milder AD phenotypes (Sanchez 2017, Winge 2011).^41, 44^ Polysensitization to house dust mite (HDM), fungus, dogs, birds and cockroaches, and maternal and paternal eczema were a risk factor for severe eczema (Sanchez 2017).^44^ Disease severity correlated with sensitization to C. Albicans measured by IgE levels and skin prick tests (Savolainen 1993).^45^ FLG mutant-type AD may have a higher risk of allergic sensitization compared with wild-type patients (Mocsai 2014).^9^ Using a data-driven approach (principal component analysis and cluster analysis), four distinct clusters of moderate-to-severe patients with AD were identified based on IgE levels and other serum mediators (Thijs 2017).^46^ Severe eczema at the age of 2 years was associated with the development of allergen sensitization at 5 years and was also more strongly associated with eczema at year 5 than those with less severe eczema. No significant association was found between eczema severity in the first 2 years of life and allergen sensitization at 2 years and wheeze and rhinitis at 5 years in this study (Quah 2015).^47^ Serum IgG levels were statistically significantly higher, whereas IgA levels were statistically significantly lower in Turkish children with severe AD compared to those with mild and moderate AD (Ercan 2013).^48^

Microbial colonization in disease severity phenotypes

Higher S. Aureus colonization prevalences were found in patients with moderate and severe disease than in those with mild disease (Clausen 2017).^49^ In addition, different Staphylococcus Aureus strain types were isolated from skin and nares from patients according to disease severity (Benito 2016).^50^ Specifically the bacterial composition of perioral skin was reported to vary with AD severity (Zheng 2019).^51^ Differences between moderate and severe disease were found in the presence of S. Aureus ribotypes on the skin (Brandwein 2018).^52^ Allergic sensitization to staphylococcal superantigens were found to be associated with moderate AD, compared to mild AD (Ong 2008).^53^ Sensitization to S. aureus–secreted enterotoxins also increased with increasing eczema severity (Semic-Jusufagic 2007).^54^ In contrast to Malassezzia species, the number of non-Malassezia yeast microbiota species decreased with increasing severity of AD (Zhang 2011).^55^ A difference for the extent of total Malassezia colonization was found in mild, moderate and severe head and neck AD in comparison to controls, with higher colonization in patients with higher severity, although no difference in species diversity related to severity was found (Kaga 2011).^56^ Lastly, a higher prevalence of Ascaris Lumbricoides infection was found in mild versus severe AD (Silva 2010).^57^ AD patients with predominant S. Aureus were found to have higher disease severity and lower microbiota diversity compared to patients without predominant S. Aureus group (Liu 2020).^58^

DNA mutations in disease severity phenotypes

The FLG mutations R501X and 2282del4 were more common in patients with severe eczema in a Swedish population (Ekelund 2008).^59^ The presence of FLG mutation p.Pro478Ser was associated with moderate and severe disease, in contrast with p.Arg501Terc and c.2282del4 (Lopes 2016).^60^ FLG mutations showed a strong association with moderate-to-severe childhood eczema compared to controls (Sandilands 2007).^61^ FLG mutations were also significantly associated with mild-to-moderate AD in childhood, in comparison to controls (Brown 2008: 52002).^62^ FLG was reported to harbor the highest number of mutations in severe atopic eczema (Pigors 2018).^63^ A significant difference between severity groups in small children was observed for FLG mutation carrier status (Holm 2019).^19^ SNPs in the promoter region of the interleukin (IL)-18 gene were more often associated with severe AD compared with mild and moderate phenotypes and compared with controls in a Egyptian population (Ibrahim 2012).^64^ The AGAC haplotype in the IL10 promoter region was significantly less frequent in patients with moderate/severe disease than in those with mild disease (Lacy 2009).^65^ Genotype and haplotype frequencies of SNPs of 13 selected cytokine/receptor genes were shown to be different between children with severe AD and controls (Kayserova 2012).^66^ Expression of the majority of genes associated with skin barrier formation was unchanged or upregulated in patients with mild AD compared to normal healthy skin (Martel 2016).^67^ In severe AD, a significantly increased representation of SNPs in genes encoding for microbial pattern recognition-related molecules by the innate immune system were found in comparison to patients with mild-to-moderate disease (Oh 2009).^68^ Severe AD in children was associated with polymorphisms of the GM-CSF gene, but not the TNF-α and IL-1β genes (Rafatpanah 2003).^69^ A SNP of the TLR2 R753Q gene was associated with severe AD phenotype, in comparison to mild-to-moderate AD (Salpietro 2011).^70^ Linkage was found between a severe phenotype and various other genomic DNA markers (Bradley 2002).^71^

Skin parameters in disease severity phenotypes

Transcriptomic analyses revealed differences in skin between paediatric patients with moderate-to-severe early-onset AD versus adult patients with longstanding AD (Brunner 2018).^72^ 1829 differentially expressed genes were identified in lesional skin and 662 differentially expressed genes in nonlesional skin of AD patients with early-onset moderate-to-severe AD, compared to healthy skin (Pavel 2021).^73^ Gene expression profiling of skin showed a type 2 inflammatory signature, positively correlated with disease severity in white adult patients with active mild, moderate or severe disease (Dyjack 2018).^74^ In comparison to mild AD, severe AD wasfound to be associated with decreased nonlesional FLG expression (Biagini Myers 2020).^6^ Severe AD was reported to be accompanied by expansion of skinhoming TH2/TC2 and TH22/TC22 subsets with lower TH1/TC1 frequencies, compared to controls (Czarnowicki 2015).^75^ The skin of moderate-to-severe AD patients of Han Chinese descent was reported to be characterized by TH17 upregulation and psoriasiform features (parakeratosis, increased neutrophils), in comparison to controls (Chan 2018).^76^ Differences in quantitative imaging parameters in skin were also found according to severity (Lee 2018).^77^ Lastly, differences were found in lipid components in moderate-to-severe AD, in comparison to healthy skin (Wang 2020).^78^

Personal and family history of allergy in disease severity phenotypes

Severity did not correlate with a personal history of asthma or hay fever or a family history of atopy (Laske 2004, Foley 2001).^42, 79^ In another study, the frequency of positive family history of allergy, past allergic history of hay fever, asthma, or hives, congenital anomalies and requirement of additional hospitalization or prolonged medical care for other medical conditions was higher in children hospitalized for severe AD, compared to controls (Torsney 1966).^80^ Parental history of asthma and eczema were associated with the “moderate-severe AD, high comorbidity” phenotype in preschool children, in contrast to the other phenotypes based on latent class analysis of severity and comorbidity (Galli 2020).^81^

Comorbidities in disease severity phenotypes

Patients with severe atopic eczema had an increase in the risk of stroke, myocardial infarction, unstable angina, atrial fibrillation, cardiovascular death and heart failure, compared to controls (Silverwood 2018).^82^ Severe AD was significantly associated with higher depression scores, compared to mild and moderate AD. No significant differences were found for scores measuring anxiety, personality, and upbringing experiences during childhood (Arima 2005).^83^ In another study, a new diagnosis of depression was reported to be associated with increasing severity, compared to no atopic eczema. This dose-reponse relation was less apparent for anxiety (Schonmann 2020).^84^ Moderate/severe patients reported greater severity and duration of itch, more pain, more sleep problems, higher prevalence of anxiety and depression, and greater health-related quality-of-life impairment, relative to mild AD (Simpson 2018).^85^ Compared with moderate and mild, patients with severe AD are reported to have more comorbidities, higher itch and pain severity, worse sleep, reduced quality of life and higher levels of anxiety and depression (de Bruin-Weller 2020).^86^ Asthma was also reported to be increasingly associated with mild, moderate and severe AD, respectively, in adults (Holm 2019).^19^ Fracture risk was increased with increasing eczema severity, with the strongest association found for spinal fractures in people with severe eczema, compared to controls (Lowe 2020).^87^

Morphology in disease severity phenotypes

With increasing severity (minimal, mild, moderate, severe), more sites of the body of children with AD in Australia were likely to be affected (Foley 2001).^79^ In moderate-severe AD patients, facial dermatitis was seen in 63% and hand dermatitis in 54%, flexural dermatitis in 50% and in 52% dermatitis occurred on the body (Lammintausta 1993).^88^ Hand eczema and flexural eczema were increasingly associated with mild, moderate and severe AD, respectively (Holm 2019).^19^

Other characteristics in disease severity phenotypes

Aortic and carotid vascular inflammation was greater in patients with very severe and moderate-to-severe AD, compared to controls (Ungar 2020).^89^ Lower protease levels were found in gingival fluid of patients with moderate-to-severe AD, when compared to controls (Valenzuela 2020).^90^ In addition to patient-reported outcome measurements indicating disease severity, more flares, working impairment and absenteeism was found in severe AD versus moderate AD (Wei 2019).^91^ Increasing AD severity was associated with worse self-rated health and quality of life in children, and with male sex in adults (Holm 2019).^19^ Early day-care attendance and exposure to molds were associated with the “moderate-severe AD, high comorbidity” phenotype in preschool children, in contrast to the other phenotypes based on latent class analysis of severity and comorbidity, and the “moderate-severe AD” phenotypes were also associated with the highest burden in terms of medication use and limitations in daily activities (Galli 2020).^81^

***Phenotypes based on disease trajectories***

Genetics in disease trajectory phenotypes

A study reported that early- and late-onset AD had different genetic backgrounds regarding skin barrier function genes (SNPs associated with early-onset AD) and immune response regulation genes (SNPs associated with late-onset AD) (Dezman 2017).^92^ FLG mutations (R501X, R2247X, S3247X and 2282del4) were more common in children with early-onset eczema (Flohr 2013, Greisenegger 2010, Luukkonen 2017).^4, 93, 94^ FLG null mutations R501X, 2282del4, and R2447X were also specifically associated with the phenotype of childhood onset AD persisting into adulthood, in contrast to rarer variants S3247X, 3702delG and 3673delC (Barker 2007, Brown 2008 49523).^95, 96^ In another study, the FLG mutation 2282del4 was associated with early-onset AD, however, no association was found for R501x, and no S3247X or R2447X mutations were detected at all (Ezzedine 2012).^97^ Children with early transient, late transient and persistent eczema (based on latent class analysis) were more likely to have a FLG mutation or additional risk alleles, compared to children without eczema (Hu 2019).^98^ Compared to no early onset of AD and no FLG mutations, early onset of AD and FLG mutations were associated with more severe disease and higher serum total IgE levels (Holm 2019).^19^ One study found that the IL-6R 358Ala allele predisposed to a persistent form of AD (Esparza-Gordillo 2013).^99^ No statistically significant difference was found when comparing frequencies polymorphisms in the coding region of SPINK5 between patients with an onset before or after the age of 2 (Zhao 2012).^100^ Linkage was found for an early-onset phenotype and various other genomic DNA markers (Bradley 2002).^71^ A high non-FLG genetic risk score was reported to be associated with childhood-onset atopic eczema in another study (Abuabara 2019).^101^ A thymic stromal lymphopoietin variant was found to be less prevalent in those with pediatric-onset AD than those without AD and to be associated with remission (Lou 2019).^102^

Comorbidities in disease trajectory phenotypes

Hypertension was more frequently present in adult-onset AD in comparison to persistent (onset < 18 years of age) AD (Megna 2017).^103^ An association was found between early-onset disease and food allergy (Roduit 2017).^104^ Infants with eczema diagnosed within the first 3 months and who required prescription topical corticosteroid treatments had the highest risk of food allergy (Martin 2015).^105^ The risk for the development of food allergy at 3 years tended to differ in subgroups based on onset of eczema in the first year: infants with onset of eczema within the first 1–2 months after birth had the highest risk, followed by the subgroup with onset of eczema between 3-4 months (Shoda 2016).^106^ The risk of developing asthma and allergic rhinitis was increased in a data-driven identified early-persistent phenotype (based on latent class analysis), with a family history of allergies being a risk factor for this phenotype, and the late-onset phenotype was only associated with increased risk of allergic rhinitis (Roduit 2017).^104^ Another study found that early eczema was associated with increased risk of asthma and allergic rhinitis at the age of 7 years specifically. Those with persistent early eczema had a higher risk for asthma and allergic rhinitis, followed by intermittent early eczema, late-onset eczema and lastly transient early eczema (Shen 2013).^107^ Compared to controls, early transient and persistent eczema (based on latent class analysis) were reported to be associated with parental history of eczema, allergy, asthma and persistent wheezing (Hu 2019).^98^ Patients with AD onset at ages 3–7 or 8–17 were demonstrated to have significantly lower rates of seasonal allergies and asthma than those with onset before age 2 (Wan 2017).^108^ Percentages of children suffering from asthma at the age of 6 were higher in infants with early-onset AD and multiple sensitizations, and infants with early-onset AD and familial history of asthma, compared to infants with early-onset AD and low sensitization, using a data-driven approach for identifying these subgroups by cluster analysis (Amat 2015).^109^ An association between childhood-onset AD was found for increased personal history of asthma, hay fever, and food allergy and family history of AD, asthma and food allergy, versus adult-onset AD (Silverberg 2018).^110^ In another study comparing adult-onset with childhood-onset disease, those with adult-onset disease were also less likely to have a history of asthma (Abuabara 2019).^101^ Early transient eczema was reported to be associated with more aggressive behaviour symptoms and all phenotypes, based on a latent class growth model, were associated with internalizing problems and attention problems (Hu 2020).^111^ All these phenotypes were also associated with asthma, food and inhalant allergic sensitization, and physician-diagnosed inhalant allergy, but not lung function measures, at age 10 years, in separate study (Hu 2020).^112^

Allergic sensitization in disease trajectory phenotypes

High allergen-specific IgE levels were reported to be associated with childhood-onset atopic eczema, and not with adult-onset disease (Abuabara 2019).^101^ Early-onset AD was also shown to be associated with an increased risk of developing allergic sensitization (Loo 2015).^113^ Another study reported that patients with early-onset-persistent AD had an increased risk of allergic rhinitis development and sensitization to inhalant allergens, and that patients with early-onset-early-resolving AD did not have an increased risk (Wang 2020).^114^ In the early-onset AD phenotype, multiple sensitization to food allergens conveyed a higher risk of sensitization to inhaled allergens than single sensitization (Just 2014).^115^ Early onset of eczema by 2 years was shown to increase the risk of eczema at year 5 and this risk was even higher in the presence of allergen sensitization and wheeze. Early onset of eczema at 2 years was associated with an increased risk of rhinitis and house dust mite allergen sensitization at 5 years of age (Quah 2015).^47^ Highest differences in number of sensitizations and total immunoglobulin E serum levels were observed between the following data-driven phenotypes, based on logistic or linear regression: patients with early-onset AD and a chronic persisting course until adulthood and patients with a late-onset AD, i.e. after the 20th year of life (Garmhausen 2013).^116^ In another study, children with persistent AD had higher sensitization to common allergens at age 9 years and a higher proportion of parental history of AD (Yamamoto-Hanada 2019).^117^ Extrinsic adult-onset AD seemed to differ from intrinsic adult-onset AD in Thai patients regarding morphological features (Kulthanan 2011).^118^

Morphology in disease trajectory phenotypes

Adult-onset AD was associated with a lower probability of flexural dermatitis and/or higher probability of hand and/or head and neck dermatitis and higher rates of nummular eczema lesions (Silverberg 2018).^110^ A lichenified/exudative eczematous pattern was the most frequent morphologic type in patients with an onset before 18 years, with the flexures as the main involved sites (Yazganoglu).^119^ When comparing childhood-onset and adult-onset AD, lichenified/ exudative flexural dermatitis was reported to be more common in childhood-onset AD, in contrast to nummular eczema-like and PN-like patterns, which were more common in adult-onset AD (Nettis 2020).^120^ No differences were found in persistent versus adult-onset AD regarding morphology of lesions in another study: erythemato-desquamative pattern was the most common clinical presentation in both groups, followed by lichenified pattern. Also, no statistically significant differences were found regarding AD lesion localization between persistent and adult-onset AD groups (Megna 2017).^103^ Skin lesions were found to predominate on the face and neck in AD patients of 45 years or older with AD since childhood, compared to those with adult-onset AD, while no differences were found for other characteristics (Dezoteux 2019).^121^ Adolescent-onset head and neck dermatitis was reported to be correlated with a past history of classic AD and exclusive head and neck involvement and adult-onset head and neck dermatitis with concomitant widespread AD (Guglielmo 2020).^122^

Immunological differences in skin and blood in disease trajectory phenotypes

Skin of new-onset paediatric AD patients was different from that of adult AD. Although excess TH2 activation characterizes both, TH9 and TH17 were highly activated at disease initiation (Esaki 2016).^123^ As described earlier, transcriptomic differences in skin were revealed between paediatric patients with early-onset AD versus adult patients with longstanding AD (Brunner 2018).^72^ Also, compared to healthy skin, 1829 differentially expressed genes were identified in lesional skin and 662 differentially expressed genes in nonlesional AD of patients with early-onset moderate-to-severe AD (Pavel 2021).^73^

Children with early-onset AD with high eosinophil and food sensitization had the highest Scoring for Atopic Dermatitis (SCORAD) and lesional TEWL values, compared to children with early-onset, non-allergic AD, children with early-onset AD with high C-reactive protein and children with middle-onset AD (mean age: 8.69 months) with inhalant sensitization, based on a data-driven analysis by using cluster analysis (Seo 2018).^124^ TEWL was also higher in children with early-onset eczema compared with unaffected infants (Flohr 2010).^4^

Differences in the number of some but not all lymphocyte subsets were found between children with and without occurrence of AD during the first year of life (Hagendorens 2004).^125^ There was an association between eczema phenotypes based on disease trajectory from birth to 8 years and serum-soluble IL-5 receptor alpha levels, with children with late-onset eczema having significantly higher s-IL-5Ra levels compared to other groups (Semic-Jusufagic 2010).^126^ Various biomarkers were found to differ in the skin and plasma between controls and infants at first presentation of AD (McAleer 2019).^127^

Besides the data-driven studies that were already mentioned, two additional studies used a data-driven approach to identify phenotypes. Based on latent class analysis, six AD phenotypes were identified in two birth cohorts. The most prevalent class was early-onset-early resolving AD, which was associated with male sex. Early-onset-persistent and early-onset-late-resolving classes were most strongly associated with an AD-genetic risk score as well as personal and parental history of atopic disease. Mid-onset-resolving AD was not associated with FLG mutations, but strongly associated with asthma (Paternoster 2018).^128^ Separately, four current AD phenotypes in school children were identified by using latent class analysis in a Korean study, including an AD phenotype that was characterized by early onset of AD with its persistence, increased serum IL-13 levels, high atopy, and increased blood eosinophils (Lee 2016).^129^

Other characteristics in disease trajectory phenotypes

Persistent AD was associated with significantly higher severity and lower mean age compared adult-onset AD (Megna 2017).^103^ There were no differences between boys and girls in the prevalence of early-onset eczema (Mohrenschlager 2006).^130^Adult-onset AD was associated with birthplace outside the United States, when compared with childhood-onset AD in an American study (Silverberg 2018).^110^ Maternal allergic history was associated with an increased risk of developing early-onset AD before 6 months. Maternal allergic history and attendance at a daycare centre increased the odds of AD development between 6 and 12 months. Risk factors for late-onset AD from 12 months were the consumption of probiotics between the age of 9 and 12 months and antibiotic treatment in the first 6 months of life (Loo 2015).^113^ A study reported that early-life exposure to phthalates is positively associated with early-onset eczema and later-onset eczema (Soomro 2018).^131^ The microbial composition and diversity of the gut microbiome differs in patients with onset of eczema over the first 1.5-2.5 years compared to controls (Wang 2008, West 2015, Yap 2014).^132-134^ In addition, while no relation was found for S. Aureus colonization in early-onset eczema, compared to controls, differences were reported in genes from gut S. aureus strains from infants with early-onset eczema, compared with strains from controls (Nowrouzian 2019).^135^ A negative association has been reported between AD with onset during the first year of life and respectively, prenatal contact with farm animals, introduction of yoghurt and shop milk and the diversity of introduction of complementary food in the first year of life (Roduit 2012).^136^ Rural living and absence of food allergy and parental history of allergic disease were found to be associated with patients with disease onset and remission in childhood, compared to patients without remission (Von Kobyletzki 2014).^137^ Farm exposures were associated with a reduced incidence of early-onset AD (Steiman 2020).^138^ Exposure to air pollutant with a particulate matter ranging from 0.1 mm to 2.5 mm during the first trimester of pregnancy was found to be associated with early-onset persistent AD and not with early-onset transient and late-onset AD (Yang 2020).^139^ Patients with early-onset of AD had worse disease control and disease persistence, compared to patients with mid-onset and late-onset disease (Wan 2019).^140^ When compared with subjects whose eczema started in childhood, those with adult-onset disease were more likely to be women, of lower childhood socioeconomic group and to be smokers in adulthood (Abuabara 2019).^101^ Compared to controls, early transient and persistent eczema in children (based on latent class analysis) were most common in first-borns; early transient eczema was most common in male children only; and late transient and persistent eczema were more often of Asian ethnicity (Hu 2019).^98^ Female sex and black and multi/other race, but not Hispanic ethnicity or any household income groups, were associated with persistent AD across all 3 ages (McKenzie 2019).^141^

***Phenotypes defined by morphological features, with subsequent investigation of associated characteristics***

Genetics in morphological phenotypes

FLG mutations were significantly associated with palmar hyperlinearity in patients with AD (Weidinger 2006, Luukonen 2017, Chen 2011).^94, 142, 143^ For keratosis pilaris (KP), conflicting results were found (Luukonen 2017, Chen 2011).^94, 143^ In a Chinese study one out of eight AD susceptibility SNPs (A allele of rs6780220) investigated were demonstrated to be preferentially lower in patients with KP than those without KP and controls (Cheng 2016).^144^ In AD patients with coexisting IV; asthma, allergic rhinoconjunctivitis and earlier AD onset were more common than in patients without IV (Bremmer 2008).^145^ Lower SCORAD scores and higher global clinical IV severity scores were also found to be associated with the AD IV phenotype (Ezzedine 2012).^97^ Significant associations were found between FLG mutation c.3321delA and IV, palmar hyperlinearity and higher global clinical dry skin scores (Zhong 2016).^146^ The percentage of FLG mutations was shown to be significantly higher in patients with isolated IV compared to patients with AD-associated IV. However, all patients with either AD or IV showed lower FLG mRNA expression compared with controls (Li 2013).^147^ FLG mutations were also associated with IV, with palmar hyperlinearity and with KP in specifically Chinese Han patients, in comparison to patients without these morphological features. No differences in FLG mutations were found for AD with and without dyshydrosis, cheilitis, and infra-auricular and retroauricular fissuring in this study (Cheng 2012).^148^ Glucose concentration in sweat was significantly higher and GLUT2 mRNA expression was significantly lower in sweat glands from patients with AD who had eczema/exudative papules, compared with those who had chronic dermatitis/lichenification and healthy subjects (Ono 2018).^149^ AD subjects with lichenoid eczema and either prurigo or papules over the cubital fossa also showed below-average sweating, compared to patients with no eczematous change (Takahashi 2013).^150^ Single nucleotide variations in genes associated with the innate immune pathway were enriched in a subgroup of AD patients with face and neck involvement (Yasuda-Sekiguchi 2020).^151^ Aryl hydrocarbon receptor single-nucleotide polymorphisms may predict a dry skin phenotype in patients with AD (Li 2019).^152^

Serum markers and other characteristics in morphological phenotypes

Patients with erythroderma-type AD, followed by widespread-type AD, had significantly higher levels of periostin compared with other phenotypes (Kou 2014).^31^ AD patients with erythroderma type showed significantly higher levels of SCCA2 compared to the other phenotypes, followed by the widespread type. However, AD patients whose lesions were not distributed systemically, such as those with limb type, had lower levels of SCCA2 (Okawa 2018).^32^ Regarding food sensitization risk, no significant difference was found for AD phenotype (no AD versus flexural versus non-flexural versus both flexural and non-flexural) after adjusting for FLG, sex and AD severity (Flohr 2014).^5^ A significant difference was found in head and neck AD of all severities for the extent of total Malassezia colonisation in comparison to controls (Kaga).^56^ Authors reported a higher prevalence of FLG mutations in those with AD and hand eczema, compared to patients with AD only and hand eczema only. Patients with only hand eczema were significantly older and had higher prevalence of positive patch test reactions and occupational dermatitis, followed by patients with both hand eczema and AD and patients with AD only (Heede 2017).^153^ In addition, hand dermatitis in the context of AD correlated with occupational exposure (Lammintausta 1993).^88^ Plasma levels of the chemokine CCL22/MDC were found to be significantly higher in children with visible flexural dermatitis than in those without visible flexural dermatitis (Yamamoto-Hanada 2020).^154^ Lastly, having numerous sites affected by AD was associated with lower quality of life (Silverberg 2019).^155^

***Phenotypes based on history of eczema herpeticum***

Patients with a history of eczema herpeticum were more likely to have a history of food allergy, asthma, sensitization to common allergens, more severe disease, AD onset before five years of age, seropositive results for herpes simplex virus and cutaneous infections with S. aureus or molluscum contagiosum virus (Beck 2009).^156^ In a second study, males were more frequently affected by eczema herpeticum than females. In this study, AD patients with history of eczema herpeticum also displayed higher total IgE serum levels, higher sensitization profiles, higher frequency of concomitant physician diagnosed asthma and allergic rhinitis and stronger severity, compared to patients without eczema herpeticum (Hinz 2011).^157^ Authors reported that AD patients with history of eczema herpeticum have identifiable defects in cell-mediated immunity that reduce their ability to control viral infections (Mathias 2013).^158^ PBMCs from patients with a history of eczema herpeticum were reported to have distinct changes to the transcriptome when compared with those from patients without a history of eczema herpeticum after HSV-1 stimulation, which the authors state implicates defects that may affect an ability to control HSV-1 infections (Bin 2014).^159^ Tregs expanded at the initial stage of HSV infection may hamper the anti–HSV-1–specific immune responses required for clearance of HSV and thereby are thought to diminish viral control (Takahashi 2014).^160^ The R501X mutation in the gene encoding FLG was reported to confer a risk for eczema herpeticum in both European and African ancestry American populations (Gao 2009).^161^ In an European American population, an association was found between AD with a history of eczema herpeticum and two thymic stromal lymphopoietin gene polymorphisms, in comparison to patients without a history of eczema herpeticum (Gao 2010).^162^ Genetic variants in the gene encoding IFNGR1 (interferon pathway gene) are implicated in susceptibility to the phenotype of AD patients with a history of eczema herpeticum in both the European American and African American population (Gao 2015).^163^ Various methylation changes have been reported to be associated with eczema herpeticum, compared with controls (Boorgula 2019).^164^ Proteins related to the skin barrier and generation of NMF were found to be expressed at lower levels in lesional versus nonlesional sites of patients with AD with and without history of eczema herpeticum. No differences were found between patients with and without history of eczema herpeticum (Broccardo 2011).^165^ Patients hospitalized with AD and eczema without EH had significantly higher odds of cellulitis, erysipelas, acute sinusitis, and pyelonephritis, but not of fungal infection, compared to patients without AD and eczema or EH. However, patients with EH had even higher odds of cellulitis (Narla 2018).^166^

***Phenotypes defined by any feature, with subsequent investigation of associated morphological characteristics***

Phenotypes based on age, with investigation of associated morphological characteristics

Of all morphological characteristics studied, xerosis and facial erythema were found more frequently in 2-year-old children with AD, compared to controls (Bohme 2000).^167^ In the same population, the majority of children had eczema on the legs, followed by the trunk, the arms, the hands and lastly the head (Bohme 2001).^168^ In another study, the only manifestation that was found in a majority of patients with infantile AD was facial dermatitis (Guo 2019).^169^ In a broader population, flexures were found to be affected in 72.5% of children with AD, following by the face in 60.1%, trunk in 38.2%, limbs in 37.6%, diaper area in 7.2% and all other sites in 4.9%. Only 5.8% of children with AD did not have face or flexural involvement (Foley 2001).^79^ In a study comparing age groups, the frequency of some clinical features showed differences, with specifically genital dermatitis and papular-lichenoid variant being more common in infants; atopic feet, prurigo-like, nummular pattern and erythroderma in preschool and school-aged children; and eyelid eczema and nipple dermatitis in adolescents. No significant differences were found for follicular pattern, infra- auricular fissures, cheilitis, retro-auricular fissures, fingertip eczema and infranasal fissures (Julian Gonzalez 2012).^170^ In another study, infants and adults showed involvements in their head and neck area the most, whereas in patients in their childhood and adolescence, the upper extremities were the most involved (Chu 2017).^171^ In one study, almost all investigated morphological features were more likely to occur in South Korean AD patients, compared to controls, with several features being of diagnostic importance only in adolescent-adult AD or in childhood AD (Lee 2000).^172^

Disease trajectory phenotypes, with investigation of associated morphological characteristics

No differences were found for persistent versus adult-onset AD regarding morphology of lesions: erythemato-desquamative pattern was the most common clinical presentation in both groups, followed by lichenified pattern. Also, no statistically significant differences were found regarding AD lesion localization between persistent and adult-onset AD (Megna 2017).^103^ Highest differences in the predilection of skin lesions were observed between patients with early-onset AD and a chronic persisting course until adulthood and patients with a late-onset AD, i.e. after the 20th year of life (Garmhausen 2013).^116^ As described earlier, adult-onset AD versus childhood-onset AD was more likely to present with lower probability of flexural dermatitis and/or higher probability of hand and/or head and neck dermatitis and higher rates of nummular eczema lesions (Silverberg 2018).^110^ Also, a lichenified/exudative eczematous pattern was the most frequent morphologic type in patients with an onset before 18 years. The main involved sites were the flexures. Half of the patients were found to have a nontypical localization of AD (Yazganoglu 2011).^119^ As also described earlier, lichenified/ exudative flexural dermatitis was reported to be more common in childhood-onset AD, in contrast to nummular eczema-like and PN-like patterns, which were more common in adult-onset AD (Nettis 2020).^120^ Non-flexural eczema was found to be an independent predictor of remission, being associated with patients with disease onset and remission in childhood, compared to patients without remission (Von Kobyletzki 2014).^137^ Skin lesions predominated on the face and neck in AD patients of 45 years or older with AD since childhood, compared to those with adult-onset AD (Dezoteux 2019).^121^ Earlier we already described that adolescent-onset head and neck dermatitis was reported to be correlated with a past history of classic AD and exclusive head and neck involvement and adult-onset head and neck dermatitis with concomitant widespread AD (Guglielmo 2020).^122^

Phenotypes based on IgE levels, with investigation of associated morphological characteristics

Extrinsic AD seemed to differ from intrinsic AD in morphological features, including distribution (Kulthanan 2011).^118^ A higher prevalence of lesions affecting only the hands was observed among patients with low total IgE, compared to patients with high total IgE, whereas lesions affecting the head and neck were least common within the subgroup with low total IgE, compared to the subgroup of non-atopic eczema with low total IgE levels and the subgroup of AE with high total IgE levels (Reefer 2007).^173^ The Dennie-Morgan fold was more present in atopiform patients (defined as AD patients with negative skin prick and Phadiatop test) compared with ‘true AD’ patients (defined as AD patients who had tested positive for allergen-specific IgE levels). Palmar hyperlinearity, KP, pityriasis alba, and nonspecific hand or foot eczema were less frequently observed in atopiform dermatitis patients compared with ‘true AD’ patients. For many other features no differences were found (Brenninkmeijer 2008).^174^ Stratification of elderly patients based on IgE levels was reported not to yield statistical differences for skin manifestations, apart from a lower incidence of IV in IgE-allergic AE as compared with indeterminate-allergic AE and non-IgE-allergic AE (Tanei 2015).^175^ Marked differences in the clinical morphology of lesions (including distribution) was found in subgroups based on Dermatophagoides pteronyssinus patch test results and mite-specific IgE (Imayama 1992).^176^

Genetic phenotypes, with investigation of associated morphological characteristics

The FLG c.3321delA allele frequency distribution was significantly associated with concomitant skin xerosis, palmar hyperlinearity, white dermatographism, IV, KP and orbital darkening (Meng 2014).^177^ FLG mutations in general were significantly associated with palmar hyperlinearity, and palmar hyperlinearity was also reported to be present in the majority of patients with AD and FLG mutations (On 2017).^178^ FLG mutations were also associated with widespread dermatitis, involvement of the palm and back of the hands, the flexor and extensor extremities, the feet and the cheeks, compared to wild-type (Carson 2012).^179^ In addition, FLG mutations increased the prevalence of foot and persistent hand dermatitis in patients with AD, in comparison to controls. No statistical differences were found for occasional hand dermatitis, face dermatitis, axillae dermatitis and abdomen, chest, or back dermatitis (Heede 2015).^180^ Another study showed that a combined presence of AD and FLG null mutation status has a significantly higher prevalence of hand eczema, compared with subjects with normal FLG status and absence of AD (Thyssen 2010).^181^ No significant associations were found between any FLG single nucleotide variations and AD-associated minor clinical features (Kim 2017).^182^

Morphological phenotypes, with investigation of associated morphological characteristics

In AD patients with coexisting IV; palmar hyperlinearity and KP were more common than in patients without IV (Bremmer 2008).^145^ No associations were found between AD patients with IV compared to without IV for various characteristics including scalp desquamation, KP, xerosis, palmar hyperlinearity and scale on legs only in univariate analyses (Ezzedine 2012).^97^ KP, palmar hyperlinearity or ichthyosis were found more often in AD patients with a history of eczema herpeticum compared to patients without prior eczema herpeticum(Beck 2009).^156^ Patients with SNPs in the Aryl hydrocarbon receptor gene were more likely to have severe dry skin scores (xerosis, IV, palmar hyperlinearity and KP) (Li 2018).^183^ Acetylating status (rapid versus slow) was shown to correlate to face/neck and limb dermatitis (Majeed 2011).^184^ More facial dermatitis was found in patients with AD compared to patients with AD and hand eczema, or hand eczema only (Heede 2017).^153^ As reported earlier, in moderate-severe AD patients, facial dermatitis was seen in 63% and hand dermatitis in 54%, flexural dermatitis in 50% and in 52% dermatitis occurred on the body (Lammintausta 1993).^88^ As also reported earlier, adolescent-onset head and neck dermatitis was reported to be correlated with a past history of classic AD and exclusive head and neck involvement and adult-onset head and neck dermatitis with concomitant widespread AD (Guglielmo 2020).^122^

Other phenotypes, with investigation of associated morphological characteristics

As described earlier, hand eczema and flexural eczema were reported to be increasingly associated with mild, moderate and severe AD, respectively (Holm 2019).^19^ Between AD patients with and without concurrent allergic contact dermatitis, no differences were found in the presence of prurigo nodularis and nummular eczema (Lee 2018).^185^ Lesional distribution of AD was reported to be associated with phenotypes based on the presence of clinically suspected bacterial infection (Li 2020).^186^

**References**

1. Jung M, Choi J, Lee SA, Kim H, Hwang J, Choi EH. Pyrrolidone carboxylic acid levels or caspase-14 expression in the corneocytes of lesional skin correlates with clinical severity, skin barrier function and lesional inflammation in atopic dermatitis. Journal of Dermatological Science. 2014;76(3):231-9.

2. Toncic RJ, Kezic S, Jakasa I, Hadzavdic SL, Balic A, Petkovic M*, et al.* Filaggrin loss-of-function mutations and levels of filaggrin degradation products in adult patients with atopic dermatitis in Croatia. Journal of the European Academy of Dermatology and Venereology. 2020;34(8):1789-94.

3. Addor FAS, Takaoka R, Rivitti EA, Aoki V. Atopic dermatitis: correlation between non-damaged skin barrier function and disease activity. International Journal of Dermatology. 2012;51(6):672-6.

4. Flohr C, England K, Radulovic S, McLean WH, Campbel LE, Barker J*, et al.* Filaggrin loss-of-function mutations are associated with early-onset eczema, eczema severity and transepidermal water loss at 3 months of age. British Journal of Dermatology. 2010;163(6):1333-6.

5. Flohr C, Perkin M, Logan K, Marrs T, Radulovic S, Campbell LE*, et al.* Atopic dermatitis and disease severity are the main risk factors for food sensitization in exclusively breastfed infants. Journal of Investigative Dermatology. 2014;134(2):345-50.

6. Biagini Myers JM, Sherenian MG, Baatyrbek Kyzy A, Alarcon R, An A, Flege Z*, et al.* Events in Normal Skin Promote Early-Life Atopic Dermatitis-The MPAACH Cohort. Journal of Allergy and Clinical Immunology: In Practice. 2020;8(7):2285-93.e6.

7. Furue M, Matsumoto T, Yamamoto T, Takeuchi S, Esaki H, Chiba T*, et al.* Correlation between serum thymus and activation-regulated chemokine levels and stratum corneum barrier function in healthy individuals and patients with mild atopic dermatitis. Journal of Dermatological Science. 2012;66(1):60-3.

8. Sakurai K, Sugiura H, Matsumoto M, Uehara M. Occurrence of patchy parakeratosis in normal-appearing skin in patients with active atopic dermatitis and in patients with healed atopic dermatitis: A cause of impaired barrier function of the atopic skin. Journal of Dermatological Science. 2002;30(1):37-42.

9. Mocsai G, Gaspar K, Nagy G, Irinyi B, Kapitany A, Biro T*, et al.* Severe skin inflammation and filaggrin mutation similarly alter the skin barrier in patients with atopic dermatitis. British Journal of Dermatology. 2014;170(3):617-24.

10. Montero-Vilchez T, Segura-Fernandez-Nogueras MV, Perez-Rodriguez I, Soler-Gongora M, Martinez-Lopez A, Fernandez-Gonzalez A*, et al.* Skin Barrier Function in Psoriasis and Atopic Dermatitis: Transepidermal Water Loss and Temperature as Useful Tools to Assess Disease Severity. Journal of Clinical Medicine. 2021;10(2).

11. Sugawara T, Kikuchi K, Tagami H, Aiba S, Sakai S. Decreased lactate and potassium levels in natural moisturizing factor from the stratum corneum of mild atopic dermatitis patients are involved with the reduced hydration state. Journal of Dermatological Science. 2012;66(2):154-9.

12. Kezic S, O'Regan GM, Yau N, Sandilands A, Chen H, Campbell LE*, et al.* Levels of filaggrin degradation products are influenced by both filaggrin genotype and atopic dermatitis severity. Allergy. 2011;66(7):934-40.

13. O'Regan GM, Kemperman PM, Sandilands A, Chen H, Campbell LE, Kroboth K*, et al.* Raman profiles of the stratum corneum define 3 filaggrin genotype-determined atopic dermatitis endophenotypes. Journal of Allergy & Clinical Immunology. 2010;126(3):574-80.e1.

14. Shen CP, Zhao MT, Jia ZX, Zhang JL, Jiao L, Ma L. Skin Ceramide Profile in Children With Atopic Dermatitis. Dermatitis. 2018;29(4):219-22.

15. Verzeaux L, Vyumvuhore R, Boudier D, Le Guillou M, Bordes S, Essendoubi M*, et al.* Atopic skin: In vivo Raman identification of global molecular signature, a comparative study with healthy skin. Experimental Dermatology. 2018;27(4):403-8.

16. Batmaz SB. Simple Markers for Systemic Inflammation in Pediatric Atopic Dermatitis Patients. Indian Journal of Dermatology. 2018;63(4):305-10.

17. Gayret OB, Nacaroglu HT, Erol M, Sener A. Neutrophil-Lymphocyte ratio and the platelet parameters as biomarkers of atopic dermatitis severity in children. Iranian Red Crescent Medical Journal. 2019;21(7).

18. de Oliveira Titz T, Orfali RL, de Lollo C, Dos Santos VG, da Silva Duarte AJ, Sato MN*, et al.* Impaired CD23 and CD62L expression and tissue inhibitors of metalloproteinases secretion by eosinophils in adults with atopic dermatitis. Journal of the European Academy of Dermatology & Venereology. 2016;30(12):2072-6.

19. Holm JG, Agner T, Clausen ML, Thomsen SF. Determinants of disease severity among patients with atopic dermatitis: association with components of the atopic march. Archives of Dermatological Research. 2019;311(3):173-82.

20. Jenerowicz D, Czarnecka-Operacz M, Silny W. Peripheral blood eosinophilia in atopic dermatitis. Acta Dermatovenerologica Alpina, Panonica et Adriatica. 2007;16(2):47-52.

21. Okano-Mitani H, Ikai K, Imamura S. Leukotriene A<inf>4</inf> hydrolase in peripheral leukocytes of patients with atopic dermatitis. Archives of Dermatological Research. 1996;288(4):168-72.

22. Dworzak MN, Froschl G, Printz D, Fleischer C, Potschger U, Fritsch G*, et al.* Skin-associated lymphocytes in the peripheral blood of patients with atopic dermatitis: Signs of subset expansion and stimulation. Journal of Allergy and Clinical Immunology. 1999;103(5):901-6.

23. McPherson T, Sherman VJ, Aslam A, Crack L, Chan H, Lloyd-Lavery A*, et al.* Filaggrin null mutations associate with increased frequencies of allergen-specific CD4+ T-helper 2 cells in patients with atopic eczema. British Journal of Dermatology. 2010;163(3):544-9.

24. Schultz Larsen F, Grunnet N. Lymphocyte transformation tests and subpopulations of lymphocytes in a population-based material of atopic dermatitis in twins. Scandinavian Journal of Immunology. 1985;21(4):375-81.

25. Miadonna A, Tedeschi A, Leggieri E, Cottini M, Menni S, Froldi M*, et al.* Characterization of T cell subsets in patients with atopic dermatitis using OKT monoclonal antibodies. Annals of Allergy. 1985;54(4):321-4.

26. Seneviratne SL, Jones L, Bailey AS, Black AP, Ogg GS. Severe atopic dermatitis is associated with a reduced frequency of IL-10 producing allergen-specific CD4+ T cells. Clinical & Experimental Dermatology. 2006;31(5):689-94.

27. Takigawa M, Tamamori T, Horiguchi D, Sakamoto T, Yamada M, Yoshioka A*, et al.* Fc epsilon receptor II/CD23-positive lymphocytes in atopic dermatitis. I. The proportion of Fc epsilon RII+ lymphocytes correlates with the extent of skin lesion. Clinical & Experimental Immunology. 1991;84(2):275-82.

28. Thompson LF, Mellon MH, Zeiger RS, Spiegelberg HL. Characterization with monoclonal antibodies of T lymphocytes bearing Fc receptors for IgE (T epsilon cells) and IgG (T gamma cells) in atopic patients. Journal of Immunology. 1983;131(6):2772-6.

29. Wehrmann W, Reinhold U, Kukel S, Franke N, Uerlich M, Kreysel HW. Selective alterations in natural killer cell subsets in patients with atopic dermatitis. International Archives of Allergy and Applied Immunology. 1990;92(3):318-22.

30. Wehrmann W, Reinhold U, Pawelec G, Wernet P, Kreysel HW. In vitro generation of IFN-gamma in relationship to in vivo concentration of IgE and IgG subclasses and Fc epsilon Rl/CD23 positive circulating lymphocytes in patients with severe atopic dermatitis (AD). Acta Dermato-Venereologica Supplementum. 1989;144:127-30.

31. Kou K, Okawa T, Yamaguchi Y, Ono J, Inoue Y, Kohno M*, et al.* Periostin levels correlate with disease severity and chronicity in patients with atopic dermatitis. British Journal of Dermatology. 2014;171(2):283-91.

32. Okawa T, Yamaguchi Y, Kou K, Ono J, Azuma Y, Komitsu N*, et al.* Serum levels of squamous cell carcinoma antigens 1 and 2 reflect disease severity and clinical type of atopic dermatitis in adult patients. Allergology International. 2018;67(1):124-30.

33. Uysal P, Avcil S, Neselioglu S, Bicer C, Catal F. Association of oxidative stress and dynamic thiol-disulphide homeostasis with atopic dermatitis severity and chronicity in children: a prospective study. Clinical & Experimental Dermatology. 2018;43(2):124-30.

34. Galli E, Rocchi L, Carello R, Giampietro PG, Panei P, Meglio P. Serum Vitamin D levels and Vitamin D supplementation do not correlate with the severity of chronic eczema in children. European Annals of Allergy & Clinical Immunology. 2015;47(2):41-7.

35. Hallau J, Hamann L, Schumann RR, Worm M, Heine G. A Promoter Polymorphism of the Vitamin D Metabolism Gene Cyp24a1 is Associated with Severe Atopic Dermatitis in Adults. Acta Dermato-Venereologica. 2016;96(2):169-72.

36. Nousbeck J, McAleer MA, Hurault G, Kenny E, Harte K, Kezic S*, et al.* MicroRNA analysis of childhood atopic dermatitis reveals a role for miR-451a. British Journal of Dermatology. 2020.

37. Bergallo M, Accorinti M, Galliano I, Coppo P, Montanari P, Quaglino P*, et al.* Expression of miRNA155, FOXP3 and RORgamma, in children with moderate and severe atopic dermatitis. Giornale Italiano di Dermatologia e Venereologia. 2020;155(2):168-72.

38. Johnson EE, Irons JS, Patterson R, Roberts M. Serum IgE concentration in atopic dermatitis. Relationship to severity of disease and presence of atopic respiratory disease. Journal of Allergy & Clinical Immunology. 1974;54(2):94-9.

39. Uehara M. Family background of respiratory atopy: a factor of serum IgE elevation in atopic dermatitis. Acta Dermato-Venereologica Supplementum. 1989;144:78-82.

40. Yoshikawa K, Ishii M, Chanoki M, Okano M, Sugai T, Abe Y*, et al.* Relationship of the severity of atopic dermatitis with nonspecific IgE, serum LDH, eosinophil counts in patients given the anti-allergic agent oxatomide. [Japanese]. Skin Research. 2000;42(2):276-84.

41. Winge MC, Bilcha KD, Lieden A, Shibeshi D, Sandilands A, Wahlgren CF*, et al.* Novel filaggrin mutation but no other loss-of-function variants found in Ethiopian patients with atopic dermatitis. British Journal of Dermatology. 2011;165(5):1074-80.

42. Laske N, Niggemann B. Does the severity of atopic dermatitis correlate with serum IgE levels? Pediatric Allergy & Immunology. 2004;15(1):86-8.

43. Mittermann I, Wikberg G, Johansson C, Lupinek C, Lundeberg L, Crameri R*, et al.* IgE Sensitization Profiles Differ between Adult Patients with Severe and Moderate Atopic Dermatitis. PLoS ONE [Electronic Resource]. 2016;11(5):e0156077.

44. Sanchez J, Sanchez A, Cardona R. Particular characteristics of atopic eczema in tropical environments. The Tropical Environment Control for Chronic Eczema and Molecular Assessment (TECCEMA) cohort study. Anais Brasileiros de Dermatologia. 2017;92(2):177-83.

45. Savolainen J, Lammintausta K, Kalimo K, Viander M. Candida albicans and atopic dermatitis. Clinical & Experimental Allergy. 1993;23(4):332-9.

46. Thijs JL, Strickland I, Bruijnzeel-Koomen CAFM, Nierkens S, Giovannone B, Csomor E*, et al.* Moving toward endotypes in atopic dermatitis: Identification of patient clusters based on serum biomarker analysis. Journal of Allergy and Clinical Immunology. 2017;140(3):730-7.

47. Quah PL, Loo EX, Lee GN, Kuo IC, Gerez I, Llanora GV*, et al.* Clinical phenotype and allergen sensitization in the first 2 years as predictors of atopic disorders at age 5 years. World Allergy Organization Journal. 2015;8(1):33.

48. Ercan H, Ispir T, Kirac D, Baris S, Ozen A, Oztezcan S*, et al.* Predictors of atopic dermatitis phenotypes and severity: roles of serum immunoglobulins and filaggrin gene mutation R501X. Allergologia et Immunopathologia. 2013;41(2):86-93.

49. Clausen ML, Edslev SM, Andersen PS, Clemmensen K, Krogfelt KA, Agner T. Staphylococcus aureus colonization in atopic eczema and its association with filaggrin gene mutations. British Journal of Dermatology. 2017;177(5):1394-400.

50. Benito D, Aspiroz C, Gilaberte Y, Sanmartin R, Hernandez-Martin A, Alonso M*, et al.* Genetic lineages and antimicrobial resistance genotypes in Staphylococcus aureus from children with atopic dermatitis: detection of clonal complexes CC1, CC97 and CC398. Journal of Chemotherapy. 2016;28(5):359-66.

51. Zheng Y, Wang Q, Ma L, Chen Y, Gao Y, Zhang G*, et al.* Alterations in the skin microbiome are associated with disease severity and treatment in the perioral zone of the skin of infants with atopic dermatitis. European Journal of Clinical Microbiology & Infectious Diseases. 2019;38(9):1677-85.

52. Brandwein M, Fuks G, Israel A, Nejman D, Straussman R, Hodak E*, et al.* Identification of a unique Staphylococcus aureus ribosomal signature in severe atopic dermatitis. British Journal of Dermatology. 2018;179(5):1222-4.

53. Ong PY, Patel M, Ferdman RM, Dunaway T, Church JA. Association of staphylococcal superantigen-specific immunoglobulin e with mild and moderate atopic dermatitis. Journal of Pediatrics. 2008;153(6):803-6.

54. Semic-Jusufagic A, Bachert C, Gevaert P, Holtappels G, Lowe L, Woodcock A*, et al.* Staphylococcus aureus sensitization and allergic disease in early childhood: population-based birth cohort study. Journal of Allergy & Clinical Immunology. 2007;119(4):930-6.

55. Zhang E, Tanaka T, Tajima M, Tsuboi R, Nishikawa A, Sugita T. Characterization of the skin fungal microbiota in patients with atopic dermatitis and in healthy subjects. Microbiology & Immunology. 2011;55(9):625-32.

56. Kaga M, Sugita T, Nishikawa A, Wada Y, Hiruma M, Ikeda S. Molecular analysis of the cutaneous Malassezia microbiota from the skin of patients with atopic dermatitis of different severities. Mycoses. 2011;54(4):e24-8.

57. Silva MT, Souza VM, Bragagnoli G, Pereira TG, Malagueno E. Atopic dermatitis and ascariasis in children aged 2 to 10 years. Jornal de Pediatria. 2010;86(1):53-8.

58. Liu Y, Wang S, Dai W, Liang Y, Shen C, Jiao L*, et al.* Distinct Skin Microbiota Imbalance and Responses to Clinical Treatment in Children With Atopic Dermatitis. Frontiers in Cellular and Infection Microbiology. 2020;10 (no pagination).

59. Ekelund E, Lieden A, Link J, Lee SP, D'Amato M, Palmer CN*, et al.* Loss-of-function variants of the filaggrin gene are associated with atopic eczema and associated phenotypes in Swedish families. Acta Dermato-Venereologica. 2008;88(1):15-9.

60. Lopes C, Rocha L, Sokhatska O, Soares J, Tavaria F, Correia O*, et al.* Filaggrin Polymorphism Pro478Ser Is Associated With the Severity of Atopic Dermatitis and Colonization by Staphylococcal aureus. Journal of Investigational Allergology & Clinical Immunology. 2016;26(1):70-2.

61. Sandilands A, Terron-Kwiatkowski A, Hull PR, O'Regan GM, Clayton TH, Watson RM*, et al.* Comprehensive analysis of the gene encoding filaggrin uncovers prevalent and rare mutations in ichthyosis vulgaris and atopic eczema. Nature Genetics. 2007;39(5):650-4.

62. Brown SJ, Relton CL, Liao H, Zhao Y, Sandilands A, Wilson IJ*, et al.* Filaggrin null mutations and childhood atopic eczema: a population-based case-control study. Journal of Allergy & Clinical Immunology. 2008;121(4):940-46.e3.

63. Pigors M, Common JEA, Wong X, Malik S, Scott CA, Tabarra N*, et al.* Exome Sequencing and Rare Variant Analysis Reveals Multiple Filaggrin Mutations in Bangladeshi Families with Atopic Eczema and Additional Risk Genes. Journal of Investigative Dermatology. 2018;138(12):2674-7.

64. Ibrahim GH, ElTabbakh MT, Gomaa AH, Mohamed EA. Interleukin-18 gene polymorphisms in Egyptian patients with allergic diseases. American Journal of Rhinology & Allergy. 2012;26(5):385-9.

65. Lacy K, Archer C, Wood N, Bidwell J. Association between a common IL10 distal promoter haplotype and IgE production in individuals with atopic dermatitis. International Journal of Immunogenetics. 2009;36(4):213-6.

66. Kayserova J, Sismova K, Zentsova-Jaresova I, Katina S, Vernerova E, Polouckova A*, et al.* A prospective study in children with a severe form of atopic dermatitis: clinical outcome in relation to cytokine gene polymorphisms. Journal of Investigational Allergology & Clinical Immunology. 2012;22(2):92-101.

67. Martel BC, Litman T, Hald A, Norsgaard H, Lovato P, Dyring-Andersen B*, et al.* Distinct molecular signatures of mild extrinsic and intrinsic atopic dermatitis. Experimental Dermatology. 2016;25(6):453-9.

68. Oh DY, Schumann RR, Hamann L, Neumann K, Worm M, Heine G. Association of the toll-like receptor 2 A-16934T promoter polymorphism with severe atopic dermatitis. Allergy. 2009;64(11):1608-15.

69. Rafatpanah H, Bennett E, Pravica V, McCoy MJ, David TJ, Hutchinson IV*, et al.* Association between novel GM-CSF gene polymorphisms and the frequency and severity of atopic dermatitis. Journal of Allergy & Clinical Immunology. 2003;112(3):593-8.

70. Salpietro C, Rigoli L, Miraglia Del Giudice M, Cuppari C, Di Bella C, Salpietro A*, et al.* TLR2 and TLR4 gene polymorphisms and atopic dermatitis in Italian children: a multicenter study. International Journal of Immunopathology & Pharmacology. 2011;24(4 Suppl):33-40.

71. Bradley M, Soderhall C, Luthman H, Wahlgren CF, Kockum I, Nordenskjold M. Susceptibility loci for atopic dermatitis on chromosomes 3, 13, 15, 17 and 18 in a Swedish population. Human Molecular Genetics. 2002;11(13):1539-48.

72. Brunner PM, Israel A, Zhang N, Leonard A, Wen HC, Huynh T*, et al.* Early-onset pediatric atopic dermatitis is characterized by T(H)2/T(H)17/T(H)22-centered inflammation and lipid alterations. Journal of Allergy and Clinical Immunology. 2018;141(6):2094-106.

73. Pavel AB, Renert-Yuval Y, Wu JN, Del Duca E, Diaz A, Lefferdink R*, et al.* Tape strips from early-onset pediatric atopic dermatitis highlight disease abnormalities in nonlesional skin. Allergy. 2021;76(1):314-25.

74. Dyjack N, Goleva E, Rios C, Kim BE, Bin L, Taylor P*, et al.* Minimally invasive skin tape strip RNA sequencing identifies novel characteristics of the type 2-high atopic dermatitis disease endotype. Journal of Allergy & Clinical Immunology. 2018;141(4):1298-309.

75. Czarnowicki T, Gonzalez J, Shemer A, Malajian D, Xu H, Zheng X*, et al.* Severe atopic dermatitis is characterized by selective expansion of circulating TH2/TC2 and TH22/TC22, but not TH17/TC17, cells within the skin-homing T-cell population. Journal of Allergy & Clinical Immunology. 2015;136(1):104-15.e7.

76. Chan TC, Sanyal RD, Pavel AB, Glickman J, Zheng X, Xu H*, et al.* Atopic dermatitis in Chinese patients shows T<inf>H</inf>2/T<inf>H</inf>17 skewing with psoriasiform features. Journal of Allergy and Clinical Immunology. 2018;142(3):1013-7.

77. Lee JH, Shih YT, Wei ML, Sun CK, Chiang BL. Classification of Established Atopic Dermatitis in Children with the In Vivo Imaging Methods. Journal of Biophotonics. 2018:e201800148.

78. Wang HC, Cui L, Jia Y, Gao Y, Zhang GL, He CF. Application of lipidomics to reveal differences of facial skin surface lipids between atopic dermatitis and healthy infants. Journal of Cosmetic Dermatology. 2020;19(6):1528-34.

79. Foley P, Zuo YQ, Plunkett A, Marks R. The frequency of common skin conditions in preschool-age children in Australia - Atopic dermatitis. Archives of Dermatology. 2001;137(3):293-300.

80. Torsney P, Blumstein GI. Atopic dermatitis. observations in natural history and prognosis. Journal of Allergy. 1966;38(1):41-5.

81. Galli E, Maiello N, Cipriani F, La Grutta S, Fasola S, Carello R*, et al.* Atopic dermatitis phenotypes in preschool and school-age children: A latent class analysis. Journal of Investigational Allergology and Clinical Immunology. 2020;30(2):108-16.

82. Silverwood RJ, Forbes HJ, Abuabara K, Ascott A, Schmidt M, Schmidt SAJ*, et al.* Severe and predominantly active atopic eczema in adulthood and long term risk of cardiovascular disease: population based cohort study. BMJ (Clinical research ed). 2018;361:k1786.

83. Arima M, Shimizu Y, Sowa J, Narita T, Nishi I, Iwata N*, et al.* Psychosomatic analysis of atopic dermatitis using a psychological test. Journal of Dermatology. 2005;32(3):160-8.

84. Schonmann Y, Mansfield KE, Hayes JF, Abuabara K, Roberts A, Smeeth L*, et al.* Atopic Eczema in Adulthood and Risk of Depression and Anxiety: A Population-Based Cohort Study. Journal of Allergy and Clinical Immunology: In Practice. 2020;8(1):248-57.e16.

85. Simpson EL, Guttman-Yassky E, Margolis DJ, Feldman SR, Qureshi A, Hata T*, et al.* Association of Inadequately Controlled Disease and Disease Severity With Patient-Reported Disease Burden in Adults With Atopic Dermatitis. JAMA Dermatology. 2018;154(8):903-12.

86. de Bruin-Weller M, Gadkari A, Auziere S, Simpson EL, Puig L, Barbarot S*, et al.* The patient-reported disease burden in adults with atopic dermatitis: a cross-sectional study in Europe and Canada. Journal of the European Academy of Dermatology and Venereology. 2020;34(5):1026-36.

87. Lowe KE, Mansfield KE, Delmestri A, Smeeth L, Roberts A, Abuabara K*, et al.* Atopic eczema and fracture risk in adults: A population-based cohort study. Journal of Allergy & Clinical Immunology. 2020;145(2):563-71.e8.

88. Lammintausta K, Kalimo K. Does a patient's occupation influence the course of atopic dermatitis? Acta Dermato-Venereologica. 1993;73(2):119-22.

89. Ungar B, Pavel AB, Robson PM, Kaufman A, Pruzan A, Brunner P*, et al.* A Preliminary 18F-FDG-PET/MRI Study Shows Increased Vascular Inflammation in Moderate-to-Severe Atopic Dermatitis. Journal of Allergy and Clinical Immunology: In Practice. 2020;8(10):3500-6.

90. Valenzuela F, Fernandez J, Aroca M, Jimenez C, Albers D, Hernandez M*, et al.* Gingival Crevicular Fluid Zinc- and Aspartyl-Binding Protease Profile of Individuals with Moderate/Severe Atopic Dermatitis. Biomolecules. 2020;10(12).

91. Wei W, Ghorayeb E, Andria M, Walker V, Schnitzer J, Kennedy M*, et al.* A real-world study evaluating adeQUacy of Existing Systemic Treatments for patients with moderate-to-severe Atopic Dermatitis (QUEST-AD): Baseline treatment patterns and unmet needs assessment. Annals of Allergy, Asthma and Immunology. 2019;123(4):381-8.e2.

92. Dezman K, Korosec P, Rupnik H, Rijavec M. SPINK5 is associated with early-onset and CHI3L1 with late-onset atopic dermatitis. International Journal of Immunogenetics. 2017;44(5):212-8.

93. Greisenegger EK, Novak N, Maintz L, Bieber T, Zimprich F, Haubenberger D*, et al.* Analysis of four prevalent filaggrin mutations (R501X, 2282del4, R2447X and S3247X) in Austrian and German patients with atopic dermatitis. Journal of the European Academy of Dermatology and Venereology. 2010;24(5):607-10.

94. Luukkonen TM, Kiiski V, Ahola M, Mandelin J, Virtanen H, Poyhonen M*, et al.* The Value of FLG Null Mutations in Predicting Treatment Response in Atopic Dermatitis: An Observational Study in Finnish Patients. Acta Dermato-Venereologica. 2017;97(4):456-63.

95. Barker JN, Palmer CN, Zhao Y, Liao H, Hull PR, Lee SP*, et al.* Null mutations in the filaggrin gene (FLG) determine major susceptibility to early-onset atopic dermatitis that persists into adulthood. Journal of Investigative Dermatology. 2007;127(3):564-7.

96. Brown SJ, Sandilands A, Zhao Y, Liao H, Relton CL, Meggitt SJ*, et al.* Prevalent and low-frequency null mutations in the filaggrin gene are associated with early-onset and persistent atopic eczema. Journal of Investigative Dermatology. 2008;128(6):1591-4.

97. Ezzedine K, Droitcourt C, Ged C, Diallo A, Hubiche T, de Verneuil H*, et al.* Usefulness of a global clinical ichthyosis vulgaris scoring system for predicting common FLG null mutations in an adult caucasian population. British Journal of Dermatology. 2012;167(5):1165-9.

98. Hu C, Duijts L, Erler NS, Elbert NJ, Piketty C, Bourdes V*, et al.* Most associations of early-life environmental exposures and genetic risk factors poorly differentiate between eczema phenotypes: the Generation R Study. British Journal of Dermatology. 2019;181(6):1190-7.

99. Esparza-Gordillo J, Schaarschmidt H, Liang L, Cookson W, Bauerfeind A, Lee-Kirsch MA*, et al.* A functional IL-6 receptor (IL6R) variant is a risk factor for persistent atopic dermatitis. Journal of Allergy and Clinical Immunology. 2013;132(2):371-7.

100. Zhao LP, Di Z, Zhang L, Wang L, Ma L, Lv Y*, et al.* Association of SPINK5 gene polymorphisms with atopic dermatitis in Northeast China. Journal of the European Academy of Dermatology & Venereology. 2012;26(5):572-7.

101. Abuabara K, Ye M, McCulloch CE, Sullivan A, Margolis DJ, Strachan DP*, et al.* Clinical onset of atopic eczema: Results from 2 nationally representative British birth cohorts followed through midlife. J Allergy Clin Immunol. 2019;144(3):710-9.

102. Lou C, Mitra N, Wubbenhorst B, D'Andrea K, Hoffstad O, Kim BS*, et al.* Association between fine mapping thymic stromal lymphopoietin and atopic dermatitis onset and persistence. Annals of Allergy, Asthma, & Immunology. 2019;123(6):595-601.e1.

103. Megna M, Patruno C, Balato A, Rongioletti F, Stingeni L, Balato N*, et al.* An Italian multicentre study on adult atopic dermatitis: persistent versus adult-onset disease. Archives of Dermatological Research. 2017;309(6):443-52.

104. Roduit C, Frei R, Depner M, Karvonen AM, Renz H, Braun-Fahrlander C*, et al.* Phenotypes of Atopic Dermatitis Depending on the Timing of Onset and Progression in Childhood. JAMA Pediatrics. 2017;171(7):655-62.

105. Martin PE, Eckert JK, Koplin JJ, Lowe AJ, Gurrin LC, Dharmage SC*, et al.* Which infants with eczema are at risk of food allergy? Results from a population-based cohort. Clinical & Experimental Allergy. 2015;45(1):255-64.

106. Shoda T, Futamura M, Yang L, Yamamoto-Hanada K, Narita M, Saito H*, et al.* Timing of eczema onset and risk of food allergy at 3 years of age: A hospital-based prospective birth cohort study. Journal of Dermatological Science. 2016;84(2):144-8.

107. Shen CY, Lin MC, Lin HK, Lin CH, Fu LS, Fu YC. The natural course of eczema from birth to age 7 years and the association with asthma and allergic rhinitis: a population-based birth cohort study. Allergy & Asthma Proceedings. 2013;34(1):78-83.

108. Wan J, Mitra N, Hoffstad OJ, Gelfand JM, Yan AC, Margolis DJ. Variations in risk of asthma and seasonal allergies between early- and late-onset pediatric atopic dermatitis: A cohort study. Journal of the American Academy of Dermatology. 2017;77(4):634-40.

109. Amat F, Saint-Pierre P, Bourrat E, Nemni A, Couderc R, Boutmy-Deslandes E*, et al.* Early-onset atopic dermatitis in children: which are the phenotypes at risk of asthma? Results from the ORCA cohort. PLoS ONE [Electronic Resource]. 2015;10(6):e0131369.

110. Silverberg JI, Vakharia PP, Chopra R, Sacotte R, Patel N, Immaneni S*, et al.* Phenotypical Differences of Childhood- and Adult-Onset Atopic Dermatitis. The Journal of Allergy & Clinical Immunology in Practice. 2018;6(4):1306-12.

111. Hu C, Nijsten T, Pasmans SGMA, de Jongste JC, Jansen PW, Duijts L. Associations of eczema phenotypes with emotional and behavioural problems from birth until school age. The Generation R Study. British Journal of Dermatology. 2020;183(2):311-20.

112. Hu C, Nijsten T, Van Meel ER, Erler NS, Piketty C, De Jong NW*, et al.* Eczema phenotypes and risk of allergic and respiratory conditions in school age children. Clinical and Translational Allergy. 2020;10(1).

113. Loo EX, Shek LP, Goh A, Teoh OH, Chan YH, Soh SE*, et al.* Atopic Dermatitis in Early Life: Evidence for at Least Three Phenotypes? Results from the GUSTO Study. International Archives of Allergy & Immunology. 2015;166(4):273-9.

114. Wang LC, Chiang BL. Early-onset-early-resolving atopic dermatitis does not increase the risk of development of allergic diseases at 3 Years old. Journal of the Formosan Medical Association. 2020;119(12):1854-61.

115. Just J, Deslandes-Boutmy E, Amat F, Desseaux K, Nemni A, Bourrat E*, et al.* Natural history of allergic sensitization in infants with early-onset atopic dermatitis: results from ORCA Study. Pediatric Allergy & Immunology. 2014;25(7):668-73.

116. Garmhausen D, Hagemann T, Bieber T, Dimitriou I, Fimmers R, Diepgen T*, et al.* Characterization of different courses of atopic dermatitis in adolescent and adult patients. Allergy. 2013;68(4):498-506.

117. Yamamoto-Hanada K, Yang L, Saito-Abe M, Sato M, Inuzuka Y, Toyokuni K*, et al.* Four phenotypes of atopic dermatitis in Japanese children: A general population birth cohort study. Allergology International. 2019;68(4):521-3.

118. Kulthanan K, Boochangkool K, Tuchinda P, Chularojanamontri L. Clinical features of the extrinsic and intrinsic types of adult-onset atopic dermatitis. Asia Pacific Allergy. 2011;1(2):80-6.

119. Yazganoglu KD, Ozkaya E. Non-typical morphology and localization in Turkish atopic dermatitis patients with onset before the age of 18 years. Indian Journal of Dermatology, Venereology & Leprology. 2011;77(1):23-7.

120. Nettis E, Ortoncelli M, Pellacani G, Foti C, Di Leo E, Patruno C*, et al.* A multicenter study on the prevalence of clinical patterns and clinical phenotypes in adult atopic dermatitis. Journal of Investigational Allergology and Clinical Immunology. 2020;30(6):448-72.

121. Dezoteux F, Astrid B, Chuffard M, Drumez E, Azib S, Staumont-Salle D. Atopic dermatitis in elderly adults. European Journal of Dermatology. 2019;29(4):371-4.

122. Guglielmo A, Sechi A, Patrizi A, Gurioli C, Neri I. Head and neck dermatitis, a subtype of atopic dermatitis induced by Malassezia spp: Clinical aspects and treatment outcomes in adolescent and adult patients. Pediatric Dermatology. 2020.

123. Esaki H, Brunner PM, Renert-Yuval Y, Czarnowicki T, Huynh T, Tran G*, et al.* Early-onset pediatric atopic dermatitis is T(H)2 but also T(H)17 polarized in skin. Journal of Allergy and Clinical Immunology. 2016;138(6):1639-51.

124. Seo E, Yoon J, Jung S, Lee J, Lee BH, Yu J. Phenotypes of atopic dermatitis identified by cluster analysis in early childhood. Journal of Dermatology. 2018;06:06.

125. Hagendorens MM, Ebo DG, Bridts CH, Van de Water L, De Clerck LS, Stevens WJ. Prenatal exposure to house dust mite allergen (Der p 1), cord blood T cell phenotype and cytokine production and atopic dermatitis during the first year of life. Pediatric Allergy & Immunology. 2004;15(4):308-15.

126. Semic-Jusufagic A, Gevaert P, Bachert C, Murray C, Simpson A, Custovic A. Increased serum-soluble interleukin-5 receptor alpha level precedes the development of eczema in children. Pediatric Allergy & Immunology. 2010;21(7):1052-8.

127. McAleer MA, Jakasa I, Hurault G, Sarvari P, McLean WHI, Tanaka RJ*, et al.* Systemic and stratum corneum biomarkers of severity in infant atopic dermatitis include markers of innate and T helper cell-related immunity and angiogenesis. British Journal of Dermatology. 2019;180(3):586-96.

128. Paternoster L, Savenije OEM, Heron J, Evans DM, Vonk JM, Brunekreef B*, et al.* Identification of atopic dermatitis subgroups in children from 2 longitudinal birth cohorts. Journal of Allergy & Clinical Immunology. 2018;141(3):964-71.

129. Lee E, Lee SH, Kwon JW, Kim YH, Cho HJ, Yang SI*, et al.* Atopic dermatitis phenotype with early onset and high serum IL-13 is linked to the new development of bronchial hyperresponsiveness in school children. Allergy. 2016;71(5):692-700.

130. Mohrenschlager M, Schafer T, Huss-Marp J, Eberlein-Konig B, Weidinger S, Ring J*, et al.* The course of eczema in children aged 5-7 years and its relation to atopy: differences between boys and girls. British Journal of Dermatology. 2006;154(3):505-13.

131. Soomro MH, Baiz N, Philippat C, Vernet C, Siroux V, Nichole Maesano C*, et al.* Prenatal Exposure to Phthalates and the Development of Eczema Phenotypes in Male Children: Results from the EDEN Mother-Child Cohort Study. Environmental Health Perspectives. 2018;126(2):027002.

132. West CE, Ryden P, Lundin D, Engstrand L, Tulic MK, Prescott SL. Gut microbiome and innate immune response patterns in IgE-associated eczema. Clinical & Experimental Allergy. 2015;45(9):1419-29.

133. Yap GC, Loo EX, Aw M, Lu Q, Shek LP, Lee BW. Molecular analysis of infant fecal microbiota in an Asian at-risk cohort-correlates with infant and childhood eczema. BMC Research Notes. 2014;7:166.

134. Wang M, Karlsson C, Olsson C, Adlerberth I, Wold AE, Strachan DP*, et al.* Reduced diversity in the early fecal microbiota of infants with atopic eczema. Journal of Allergy & Clinical Immunology. 2008;121(1):129-34.

135. Nowrouzian FL, Ljung A, Nilsson S, Hesselmar B, Adlerberth I, Wold AE. Neonatal gut colonization by Staphylococcus aureus strains with certain adhesins and superantigens is negatively associated with subsequent development of atopic eczema. British Journal of Dermatology. 2019;180(6):1481-8.

136. Roduit C, Frei R, Loss G, Büchele G, Weber J, Depner M*, et al.* Development of atopic dermatitis according to age of onset and association with early-life exposures. J Allergy Clin Immunol. 2012;130(1):130-6.e5.

137. von Kobyletzki LB, Bornehag CG, Breeze E, Larsson M, Lindström CB, Svensson Å. Factors associated with remission of eczema in children: a population-based follow-up study. Acta Derm Venereol. 2014;94(2):179-84.

138. Steiman CA, Evans MD, Lee KE, Lasarev MR, Gangnon RE, Olson BF*, et al.* Patterns of farm exposure are associated with reduced incidence of atopic dermatitis in early life. Journal of Allergy and Clinical Immunology. 2020;146(6):1379-86.e6.

139. Yang SI, Lee SH, Lee SY, Kim HC, Kim HB, Kim JH*, et al.* Prenatal PM2.5 exposure and vitamin D-associated early persistent atopic dermatitis via placental methylation. Annals of Allergy, Asthma, & Immunology. 2020;125(6):665-73.e1.

140. Wan J, Mitra N, Hoffstad OJ, Yan AC, Margolis DJ. Longitudinal atopic dermatitis control and persistence vary with timing of disease onset in children: A cohort study. Journal of the American Academy of Dermatology. 2019;81(6):1292-9.

141. McKenzie C, Silverberg JI. The prevalence and persistence of atopic dermatitis in urban United States children. Annals of Allergy, Asthma, & Immunology. 2019;123(2):173-8.e1.

142. Weidinger S, Illig T, Baurecht H, Irvine AD, Rodriguez E, Diaz-Lacava A*, et al.* Loss-of-function variations within the filaggrin gene predispose for atopic dermatitis with allergic sensitizations. Journal of Allergy and Clinical Immunology. 2006;118(1):214-9.

143. Chen H, Common JEA, Haines RL, Balakrishnan A, Brown SJ, Goh CSM*, et al.* Wide spectrum of filaggrin-null mutations in atopic dermatitis highlights differences between Singaporean Chinese and European populations. British Journal of Dermatology. 2011;165(1):106-14.

144. Cheng F, Zhao JH, Tang XF, Cheng H, Sheng YJ, Jiang XY*, et al.* Association of the chromosome 11p13.5 variant and atopic dermatitis with a family history of atopy in the Chinese Han population. Asian Pacific Journal of Allergy & Immunology. 2016;34(2):109-14.

145. Bremmer SE, Hanifin JM, Simpson EL. Clinical detection of icthyosis vulgaris in an atopic dermatits clinic: Implications for allergic respiratory disease and prognosis. Journal of the American Academy of Dermatology. 2008;59(1):72-8.

146. Zhong WL, Wu X, Yu B, Zhang J, Zhang W, Xu N*, et al.* Filaggrin Gene Mutation c.3321delA is Associated with Dry Phenotypes of Atopic Dermatitis in the Chinese Han Population. Chinese Medical Journal. 2016;129(12):1498-500.

147. Li M, Cheng R, Shi M, Liu J, Zhang G, Liu Q*, et al.* Analyses of FLG mutation frequency and filaggrin expression in isolated ichthyosis vulgaris (IV) and atopic dermatitis-associated IV. British Journal of Dermatology. 2013;168(6):1335-8.

148. Cheng R, Li M, Zhang H, Guo Y, Chen X, Tao J*, et al.* Common FLG mutation K4671X not associated with atopic dermatitis in Han Chinese in a family association study. PLoS ONE [Electronic Resource]. 2012;7(11):e49158.

149. Ono E, Murota H, Mori Y, Yoshioka Y, Nomura Y, Munetsugu T*, et al.* Sweat glucose and GLUT2 expression in atopic dermatitis: Implication for clinical manifestation and treatment. PLoS ONE [Electronic Resource]. 2018;13(4):e0195960.

150. Takahashi A, Murota H, Matsui S, Kijima A, Kitaba S, Lee JB*, et al.* Decreased Sudomotor Function is Involved in the Formation of Atopic Eczema in the Cubital Fossa. Allergology International. 2013;62(4):473-8.

151. Yasuda-Sekiguchi F, Shiohama A, Fukushima A, Obata S, Mochimaru N, Honda A*, et al.* Single nucleotide variations in genes associated with innate immunity are enriched in Japanese adult cases of face and neck type atopic dermatitis. Journal of Dermatological Science. 2020.

152. Li ZZ, Zhong WL, Hu H, Chen XF, Zhang W, Huang HY*, et al.* Aryl hydrocarbon receptor polymorphisms are associated with dry skin phenotypes in Chinese patients with atopic dermatitis. Clinical & Experimental Dermatology. 2019;44(6):613-9.

153. Heede NG, Thyssen JP, Thuesen BH, Linneberg A, Szecsi PB, Stender S*, et al.* Health-related quality of life in adult dermatitis patients stratified by filaggrin genotype. Contact Dermatitis. 2017;76(3):167-77.

154. Yamamoto-Hanada K, Kawakami E, Saito-Abe M, Sato M, Mitsubuchi H, Oda M*, et al.* Exploratory analysis of plasma cytokine/chemokine levels in 6-year-old children from a birth cohort study. Cytokine. 2020;130 (no pagination).

155. Silverberg JI, Margolis DJ, Boguniewicz M, Fonacier L, Grayson MH, Ong PY*, et al.* Distribution of atopic dermatitis lesions in United States adults. Journal of the European Academy of Dermatology & Venereology. 2019;33(7):1341-8.

156. Beck LA, Boguniewicz M, Hata T, Schneider LC, Hanifin J, Gallo R*, et al.* Phenotype of atopic dermatitis subjects with a history of eczema herpeticum. Journal of Allergy & Clinical Immunology. 2009;124(2):260-9, 9.e1-7.

157. Hinz T, Zaccaro D, Byron M, Brendes K, Krieg T, Novak N*, et al.* Atopic dermo-respiratory syndrome is a correlate of eczema herpeticum. Allergy. 2011;66(7):925-33.

158. Mathias RA, Weinberg A, Boguniewicz M, Zaccaro DJ, Armstrong B, Schneider LC*, et al.* Atopic dermatitis complicated by eczema herpeticum is associated with HLA B7 and reduced interferon-gamma-producing CD8+ T cells. British Journal of Dermatology. 2013;169(3):700-3.

159. Bin L, Edwards MG, Heiser R, Streib JE, Richers B, Hall CF*, et al.* Identification of novel gene signatures in patients with atopic dermatitis complicated by eczema herpeticum. Journal of Allergy & Clinical Immunology. 2014;134(4):848-55.

160. Takahashi R, Sato Y, Kurata M, Yamazaki Y, Kimishima M, Shiohara T. Pathological role of regulatory T cells in the initiation and maintenance of eczema herpeticum lesions. Journal of Immunology. 2014;192(3):969-78.

161. Gao PS, Rafaels NM, Hand T, Murray T, Boguniewicz M, Hata T*, et al.* Filaggrin mutations that confer risk of atopic dermatitis confer greater risk for eczema herpeticum. J Allergy Clin Immunol. 2009;124(3):507-13, 13.e1-7.

162. Gao PS, Rafaels NM, Mu D, Hand T, Murray T, Boguniewicz M*, et al.* Genetic variants in thymic stromal lymphopoietin are associated with atopic dermatitis and eczema herpeticum. Journal of Allergy and Clinical Immunology. 2010;125(6):1403-7.e4.

163. Gao L, Bin L, Rafaels NM, Huang L, Potee J, Ruczinski I*, et al.* Targeted deep sequencing identifies rare loss-of-function variants in IFNGR1 for risk of atopic dermatitis complicated by eczema herpeticum. Journal of Allergy & Clinical Immunology. 2015;136(6):1591-600.

164. Boorgula MP, Taub MA, Rafaels N, Daya M, Campbell M, Chavan S*, et al.* Replicated methylation changes associated with eczema herpeticum and allergic response. Clinical Epigenetics. 2019;11(1):122.

165. Broccardo CJ, Mahaffey S, Schwarz J, Wruck L, David G, Schlievert PM*, et al.* Comparative proteomic profiling of patients with atopic dermatitis based on history of eczema herpeticum infection and Staphylococcus aureus colonization. Journal of Allergy and Clinical Immunology. 2011;127(1):186-U300.

166. Narla S, Silverberg JI. Association between atopic dermatitis and serious cutaneous, multiorgan and systemic infections in US adults. Annals of Allergy, Asthma, & Immunology. 2018;120(1):66-72.e11.

167. Bohme M, Svensson A, Kull I, Wahlgren CF. Hanifin's and Rajka's minor criteria for atopic dermatitis: which do 2-year-olds exhibit? Journal of the American Academy of Dermatology. 2000;43(5 Pt 1):785-92.

168. Bohme M, Svensson A, Kull I, Nordvall SL, Wahlgren CF. Clinical features of atopic dermatitis at two years of age: a prospective, population-based case-control study. Acta Dermato-Venereologica. 2001;81(3):193-7.

169. Guo Y, Zhang H, Liu Q, Wei F, Tang J, Li P*, et al.* Phenotypic analysis of atopic dermatitis in children aged 1-12 months: elaboration of novel diagnostic criteria for infants in China and estimation of prevalence. Journal of the European Academy of Dermatology & Venereology. 2019;33(8):1569-76.

170. Julian-Gonzalez RE, Orozco-Covarrubias L, Duran-McKinster C, Palacios-Lopez C, Ruiz-Maldonado R, Saez-de-Ocariz M. Less common clinical manifestations of atopic dermatitis: prevalence by age. Pediatric Dermatology. 2012;29(5):580-3.

171. Chu H, Shin JU, Park CO, Lee H, Lee J, Lee KH. Clinical diversity of atopic dermatitis: A review of 5,000 patients at a single institute. Allergy, Asthma and Immunology Research. 2017;9(2):158-68.

172. Lee HJ, Cho SH, Ha SJ, Ahn WK, Park YM, Byun DG*, et al.* Minor cutaneous features of atopic dermatitis in South Korea. International Journal of Dermatology. 2000;39(5):337-42.

173. Reefer AJ, Satinover SM, Wilson BB, Woodfolk JA. The relevance of microbial allergens to the IgE antibody repertoire in atopic and nonatopic eczema. Journal of Allergy & Clinical Immunology. 2007;120(1):156-63.

174. Brenninkmeijer EE, Spuls PI, Legierse CM, Lindeboom R, Smitt JH, Bos JD. Clinical differences between atopic and atopiform dermatitis. Journal of the American Academy of Dermatology. 2008;58(3):407-14.

175. Tanei R. Clinical Characteristics, Treatments, and Prognosis of Atopic Eczema in the Elderly. Journal of Clinical Medicine. 2015;4(5):979-97.

176. Imayama S, Hashizume T, Miyahara H, Tanahashi T, Takeishi M, Kubota Y*, et al.* Combination of patch test and IgE for dust mite antigens differentiates 130 patients with atopic dermatitis into four groups. Journal of the American Academy of Dermatology. 1992;27(4):531-8.

177. Meng L, Wang L, Tang H, Tang X, Jiang X, Zhao J*, et al.* Filaggrin gene mutation c.3321delA is associated with various clinical features of atopic dermatitis in the Chinese Han population. PLoS ONE [Electronic Resource]. 2014;9(5):e98235.

178. On HR, Lee SE, Kim SE, Hong WJ, Kim HJ, Nomura T*, et al.* Filaggrin Mutation in Korean Patients with Atopic Dermatitis. Yonsei Medical Journal. 2017;58(2):395-400.

179. Carson CG, Rasmussen MA, Thyssen JP, Menne T, Bisgaard H. Clinical Presentation of Atopic Dermatitis by Filaggrin Gene Mutation Status during the First 7 Years of Life in a Prospective Cohort Study. PLoS ONE. 2012;7 (11) (no pagination)(e48678).

180. Heede NG, Thyssen JP, Thuesen BH, Linneberg A, Johansen JD. Anatomical patterns of dermatitis in adult filaggrin mutation carriers. Journal of the American Academy of Dermatology. 2015;72(3):440-8.

181. Thyssen JP, Carlsen BC, Menne T, Linneberg A, Nielsen NH, Meldgaard M*, et al.* Filaggrin null mutations increase the risk and persistence of hand eczema in subjects with atopic dermatitis: results from a general population study. British Journal of Dermatology. 2010;163(1):115-20.

182. Kim M, Yoo J, Kim J, Park J, Han E, Jang W*, et al.* Association of FLG single nucleotide variations with clinical phenotypes of atopic dermatitis. PLoS ONE [Electronic Resource]. 2017;12(12):e0190077.

183. Li ZZ, Zhong WL, Hu H, Chen XF, Zhang W, Huang HY*, et al.* Aryl hydrocarbon receptor polymorphisms are associated with dry skin phenotypes in Chinese patients with atopic dermatitis. Clinical & Experimental Dermatology. 2018;29:29.

184. Majeed Al-Razzuqi RA, Al-Jeboori AA, Al-Waiz MM. Acetylation phenotype variation in pediatric patients with atopic dermatitis. Indian Journal of Dermatology. 2011;56(2):150-2.

185. Lee S, Wang HY, Kim E, Hwang HJ, Choi E, Lee H*, et al.* Clinical characteristics and genetic variation in atopic dermatitis patients with and without allergic contact dermatitis. European Journal of Dermatology. 2018;28(5):637-43.

186. Li Y, Xu W, Li LF. Risk Factors in Outpatients with Dermatitis and Eczema in Tertiary Hospitals of China Who Have Clinically Suspected Bacterial Infection. Biomed Research International. 2020;2020.
